# Biological basis of extensive pleiotropy between blood traits and cancer risk

**DOI:** 10.1186/s13073-024-01294-8

**Published:** 2024-02-02

**Authors:** Miguel Angel Pardo-Cea, Xavier Farré, Anna Esteve, Joanna Palade, Roderic Espín, Francesca Mateo, Eric Alsop, Marc Alorda, Natalia Blay, Alexandra Baiges, Arzoo Shabbir, Francesc Comellas, Antonio Gómez, Montserrat Arnan, Alex Teulé, Monica Salinas, Laura Berrocal, Joan Brunet, Paula Rofes, Conxi Lázaro, Miquel Conesa, Juan Jose Rojas, Lars Velten, Wojciech Fendler, Urszula Smyczynska, Dipanjan Chowdhury, Yong Zeng, Housheng Hansen He, Rong Li, Kendall Van Keuren-Jensen, Rafael de Cid, Miquel Angel Pujana

**Affiliations:** 1 ProCURE, Catalan Institute of Oncology, Oncobell, Bellvitge Institute for Biomedical Research (IDIBELL), L’Hospitalet del Llobregat, 08908 Barcelona, Catalonia Spain; 2 Genomes for Life – GCAT Lab Group, Institut Germans Trias i Pujol (IGTP), Badalona, 08916 Barcelona, Catalonia Spain; 3 Badalona Applied Research Group in Oncology (B-ARGO), Catalan Institute of Oncology, Institut Germans Trias i Pujol (IGTP), Badalona, 08916 Barcelona, Catalonia Spain; 4 Cancer and Cell Biology, Translational Genomics Research Institute (TGen), Arizona, Phoenix, AZ 85004 USA; 5 Department of Mathematics, Technical University of Catalonia, Castelldefels, 08860 Barcelona, Catalonia Spain; 6 Department of Biosciences, Faculty of Sciences and Technology (FCT), University of Vic - Central University of Catalonia (UVic-UCC), Vic, 08500 Barcelona, Catalonia Spain; 7 Department of Hematology, Catalan Institute of Oncology, Oncobell, Bellvitge Institute for Biomedical Research (IDIBELL), L’Hospitalet del Llobregat, 08908 Barcelona, Catalonia Spain; 8 Hereditary Cancer Program, Catalan Institute of Oncology, Oncobell, Bellvitge Institute for Biomedical Research (IDIBELL), L’Hospitalet del Llobregat, 08908 Barcelona, Catalonia Spain; 9 OncoGir, Catalan Institute of Oncology, Girona Biomedical Research Institute (IDIBGI), 17190 Salt, Catalonia Spain; 10 Biomedical Research Network Centre in Cancer (CIBERONC), Instituto de Salud Carlos III, 28222 Madrid, Spain; 11 Department of Pathology and Experimental Therapies, University of Barcelona (UB), Oncobell, Bellvitge Institute for Biomedical Research (IDIBELL), L’Hospitalet del Llobregat, 08908 Barcelona, Catalonia Spain; 12 Centre for Genomic Regulation (CRG), The Barcelona Institute of Science and Technology (BIST), 08003 Barcelona, Spain; 13 University Pompeu Fabra (UPF), 08002 Barcelona, Spain; 14 Department of Biostatistics and Translational Medicine, Medical University of Lodz, 92-215 Lodz, Poland; 15 Department of Radiation Oncology, Dana-Farber Cancer Institute, Boston, MA 02115 USA; 16 Center for BRCA and Related Genes, Dana-Farber Cancer Institute, Boston, MA 02115 USA; 17 Harvard Medical School, Boston, MA 02115 USA; 18 Princess Margaret Cancer Center, University Health Network, Toronto, ON M5G 2C4 Canada; 19 Department of Medical Biophysics, University of Toronto, Toronto, ON M5G 1L7 Canada; 20 Department of Biochemistry and Molecular Medicine, School of Medicine and Health Sciences, The George Washington University, Washington, DC 20052 USA; 21 Biomedical Research Network Centre in Respiratory Diseases (CIBERES), Instituto de Salud Carlos III, 28222 Madrid, Spain

**Keywords:** Blood trait, Cancer, Eosinophil, Hematopoiesis, Myeloid, Pleiotropy, Telomere, Y-RNA

## Abstract

**Background:**

The immune system has a central role in preventing carcinogenesis. Alteration of systemic immune cell levels may increase cancer risk. However, the extent to which common genetic variation influences blood traits and cancer risk remains largely undetermined. Here, we identify pleiotropic variants and predict their underlying molecular and cellular alterations.

**Methods:**

Multivariate Cox regression was used to evaluate associations between blood traits and cancer diagnosis in cases in the UK Biobank. Shared genetic variants were identified from the summary statistics of the genome-wide association studies of 27 blood traits and 27 cancer types and subtypes, applying the conditional/conjunctional false-discovery rate approach. Analysis of genomic positions, expression quantitative trait loci, enhancers, regulatory marks, functionally defined gene sets, and bulk- and single-cell expression profiles predicted the biological impact of pleiotropic variants. Plasma small RNAs were sequenced to assess association with cancer diagnosis.

**Results:**

The study identified 4093 common genetic variants, involving 1248 gene loci, that contributed to blood–cancer pleiotropism. Genomic hotspots of pleiotropism include chromosomal regions 5p15-*TERT* and 6p21-*HLA*. Genes whose products are involved in regulating telomere length are found to be enriched in pleiotropic variants. Pleiotropic gene candidates are frequently linked to transcriptional programs that regulate hematopoiesis and define progenitor cell states of immune system development. Perturbation of the myeloid lineage is indicated by pleiotropic associations with defined master regulators and cell alterations. Eosinophil count is inversely associated with cancer risk. A high frequency of pleiotropic associations is also centered on the regulation of small noncoding Y-RNAs. Predicted pleiotropic Y-RNAs show specific regulatory marks and are overabundant in the normal tissue and blood of cancer patients. Analysis of plasma small RNAs in women who developed breast cancer indicates there is an overabundance of Y-RNA preceding neoplasm diagnosis.

**Conclusions:**

This study reveals extensive pleiotropism between blood traits and cancer risk. Pleiotropism is linked to factors and processes involved in hematopoietic development and immune system function, including components of the major histocompatibility complexes, and regulators of telomere length and myeloid lineage. Deregulation of Y-RNAs is also associated with pleiotropism. Overexpression of these elements might indicate increased cancer risk.

**Supplementary Information:**

The online version contains supplementary material available at 10.1186/s13073-024-01294-8.

## Background

Cancer cells have evolved multiple mechanisms to avoid their recognition and elimination by the immune system [1]. Cancer immune evasion can be achieved by modulating antigen presentation, promoting immune tolerance, and/or recruiting immunosuppressive cell types, among several complementary strategies [2]. While these mechanisms are well-established in cancer progression, analogous tactics may endorse cancer initiation [3]. Evidence from mouse models with defined alterations of immune system factors [4–10], and epidemiological data from immunodeficient conditions [11, 12], indicate that immune surveillance substantially contributes to eliminating malignant cells at early stages. Characterization of premalignant lesions in mouse and human tissue also reveals meaningful changes in immune system factors and cell populations [13–16]. Indeed, a substantial proportion of genetic variants associated with cancer risk converges on immune system-related genes, pathways, and/or cell phenotypes [17–20]. However, we do not yet fully understand which systemic immune cell alterations markedly influence cancer risk [21, 22].

Naïve and educated immune cells circulate through the blood from one tissue to another, functioning to protect against harmful internal and external factors. However, there is substantial interindividual variation in the normality of blood traits. This variability is largely determined by inherited genetic factors [23, 24]. More than 7000 genetic loci have been associated with differences in blood traits among individuals in the general population, and several of the corresponding loci are linked to Mendelian blood disorders and the risk of a range of immune-related conditions [25]. Analysis of a subset of rare genetic variants associated with blood traits identified pleiotropic loci for the risk of breast and skin cancer [25]. However, despite the key role of the immune system in preventing carcinogenesis, the impact of common genetic variation on blood trait–cancer pleiotropism remains relatively undetermined.

To examine the basis of blood trait–cancer pleiotropism, we analyzed the results of the genome-wide association studies (GWASs) of 27 blood traits [24, 25] and 27 cancer types, including breast cancer subtypes [26]. The results reveal extensive pleiotropy, identifying thousands of genetic variants that influence one or more blood trait, as well as one or more of the common cancer types and/or subtypes. Pleiotropism is thought to be caused by the perturbation of telomere length control, and alteration of immune system processes, in which master regulators and transcriptional programs of hematopoiesis are of particular relevance. The pleiotropic loci are also found to be enriched in the presence of functional and derived Y-RNA sequences, whose overexpression is associated with cancer status [27, 28] and that might indicate a relatively high risk of cancer.

## Methods

### Blood trait–cancer diagnosis association study

The UK Biobank (UKBB: https://www.ukbiobank.ac.uk/) is a large prospective cohort study for research into the causes of human disease. Full details of the UKBB have been described previously [29]. Briefly, it includes approximately half a million individuals, aged 40–69 years, recruited between 2006 and 2010 in the UK. Baseline sociodemographic, medical history, lifestyle exposures, and physical information, and blood samples were collected at the time of recruitment. Cancer diagnoses were obtained by linkage to electronic medical records, and national cancer and death registries. Data from 503,317 individuals were obtained following approval of project application #61744. To analyze the associations, and following the original study [24], we excluded individuals who showed (1) a discrepancy between self-reported sex and inferred genetic sex (*n* = 373); (2) heterozygosity outlier (*n* = 968); (3) chromosome aneuploidy (*n* = 651); (4) no information about genetic principal components (*n* = 14,242); (5) a cancer diagnosis before blood test (*n* = 28,795); (6) no information from the blood test (*n* = 23,153); (7)) a discrepancy between the dates of the health care record and of the blood test (*n* = 1); (8) an outlier measure (> 3 times the interquartile range) for the leukocyte (*n* = 1,124) or platelet (*n* = 871) count; and (9) a C-reactive protein (CRP) value > 10 mg/L (*n* = 19,475). The outlier threshold applied to the leukocyte and platelet counts was based on a previous study of prostate cancer risk [30] and aimed to exclude individuals with probable chronic inflammation and thrombocytosis, respectively. These pathological processes could have confounded the study conclusions as they have been associated with cancer development and progression [31–34]. Similarly, individuals with a CRP measure > 10 mg/L were excluded because this threshold constitutes clinical evidence of an acute infection or inflammatory reaction [35, 36], which could also confound the conclusions concerning cancer risk. Data from 32 individuals who withdrew from the UKBB project were also discarded. In total, 170,512 men and 198,331 women were included in the study. The cancer types were based on the International Classification of Diseases – 10th Edition (ICD-10) code for malignant cancer (ICD-10 Chapter C) [37]. Benign neoplasms (ICD-10-CM D10-D49) were not considered. The main outcome of the study was defined as a first diagnosis of cancer after the date of recruitment or a cancer-related death. Similarly, secondary outcomes of the study were considered for the most common cancer types: breast, colon, lung, and prostate. Peripheral blood samples of the UKBB participants were typically taken at the time of enrollment [29]. Values of all blood traits were log_2_-transformed for the analysis. Multivariate Cox proportional hazards models were used to assess the association between blood traits and cancer diagnosis by considering a descriptive model-building strategy. The follow-up time was defined as being from the date of enrollment to the date of cancer diagnosis, death, loss to follow-up, or administrative censoring (March 31st 2016 for England and Wales, and November 30th 2015 for Scotland), whichever occurred first. We estimated HRs and 95% confidence intervals (CIs) associated with the risk of cancer diagnosis for a doubling of the value of each log-transformed blood trait. Models for the main outcome (all-cancer diagnosis), as well as separate lung and colon cancer diagnosis outcomes, were stratified by sex, alcohol consumption (non-drinker, drinker, unknown), the number of self-reported comorbidities (0, 1, 2, 3–5, > 5), and region of recruitment (England, Wales, Scotland), and adjusted by age at enrollment, body mass index (BMI), smoking status (non-smoker, smoker, unknown), highest level of educational qualifications (preparatory school, high school, college, other, unknown), the Townsend deprivation index (grouped into quintiles), and the top 40 genetic principal components [24]. To account for departures from the proportional hazards assumption more accurately, we used penalized splines for age at enrollment and BMI. Multicollinearity was assessed using the variance inflation factor. To consider the potential influence of an underlying cancer on blood traits levels, we conducted separate analyses for cancer diagnoses after 1 year and within 1 year following enrollment. Analyses were performed in R v 4.1.2 (R Core Team, 2020) using the *survival* and *survminer* packages.

### GWAS data processing

The GWAS summary statistics of blood traits and cancer risk studies were obtained from the corresponding data sources, detailed in Additional file 1: Table S1. The study did not require individual data. For each of the variant-summary statistics, the following quality controls were applied, removing cases of single-nucleotide polymorphisms (SNPs) without a reference identifier (rs ID); duplication; poor imputation (information score < 0.9); value of minor allele frequency (MAF) ≤ 0.01; strand-ambiguous alleles; and/or allele sample sizes five standard deviations or more away from the mean.

### Shared genetic architecture analysis

The heritability of all phenotypes and genetic correlations were estimated by the linkage disequilibrium (LD) score regression method [38], restricted to HapMap3 SNPs. The pleiotropy-informed conditional false-discovery rate approach [39] was employed to detect shared genetic factors, using pleio-false discovery rate (pleioFDR) software (https://github.com/precimed/pleiofdr/) and computing conjFDR statistics. The conjFDR is given as the maximum value between the conditional FDRs (condFDR) of two given conditions. The method is not affected by the direction of the allele effects [40, 41]. To ensure the results were comparable, we analyzed a common set of 5,264,785 SNPs, from which all summary statistics were derived. Shared genetic variants were defined by conjFDR < 0.05. We performed LD clumping to define independently significant SNPs (PLINK software, p1 = 0.05, LD threshold *r*^*2*^ = 0.6, and physical distance threshold for clumping 1000 kb) and lead SNPs (PLINK software, p1 = 0.05, *r*^*2*^ = 0.1, and distance 1000 kb). Genomic risk loci were found by merging lead SNPs if they were closer than 250 kb. Candidate SNPs were mapped to independently significant SNPs using this clumping strategy. Stratified Q-Q plots were obtained using pleioFDR to visualize shared genetic architecture. In these representations, the probabilities of the primary phenotype were plotted against the null distribution. In the same plots, SNP subsets of the primary phenotype were represented as being conditioned by the significance of the association with the secondary phenotype (*p* < 0.1, 0.01, and 0.001). The genomic inflation factor (lambda) for each of the thresholds was computed to establish the existence of pleiotropy in the stratified Q-Q plots.

### Genetic data and functional associations

Positional information about genetic elements was obtained from ENSEMBL BioMart [42] version 2.52.0, genome build GRCh37/hg19. This resource was used to assign the identified pleiotropic variants to defined gene loci. The variants linked to the genes previously associated with leukocyte telomere length were identified using the original study annotations [43–45] and not considering other types of data. Functional annotations (GO terms and Reactome pathways) of positional protein-coding genes were analyzed using the *gost* tool of gprofiler2 [46], with default parameters and using the FDR approach for multiple-test correction. The *cis* eQTL data from blood and immortalized lymphocytes were obtained from the GTEx project [47]. The pleiotropic variants in specific loci were examined for eQTLs of the corresponding positional gene, and the resulting pleiotropic/eQTL proportion compared with the frequency of eQTLs identified in sets of 200 randomly selected variants with defined MAF (European > 0.05), using different LD thresholds (five random sets; average *r*^*2*^ = 0.10, 0.25, 0.50, 0.75, or 0.90) in 1000 random protein-coding gene loci. These genes were randomly selected from among those detected (defined as RNA-seq transcripts per million (TPM) > 1) in all immune major cell types [48]. The MAF information was obtained from the 1000 Genomes Project (ftp.1000genomes.ebi.ac.uk.) [49]. The SNPs were assigned to the nearest gene locus (± 100 kb) using ENSEMBL BioMart [42] 2.52.0 (GRCh37/hg19), and LD was estimated using LDlinkR software [50]. A two-proportion *Z*-test was done to assess the enrichment of eQTLs in sets of pleiotropic variants of defined gene loci relative to randomly selected variants/genes. The enhancer data from immune cell types were obtained from the FANTOM Consortium [51] (predefined enhancer data; https://enhancer.binf.ku.dk/presets/). Fisher’s exact test and the FDR approach were used to assess the proportion of pleiotropic variants identified in immune cell enhancers, relative to the proportions in adipose and brain data from the same study [51]. The list of mammalian phenotypes (MPs) and the corresponding mouse genes and human orthologs linked to immune system alterations was obtained from The Mammalian Phenotype Browser (keywords: “inflammation”, “inflammatory”, and “immune”; MP:0005387) [52]. Myeloid-related gene sets were also obtained from this source [52]. The hypergeometric test was applied to assess the degree of overlap of pleiotropic gene candidates (positional) among all genes annotated with the given term, and considering all protein-coding human orthologs as background. The Locus Overlap Analysis (LOLA; R version 1.28.0) [53] was applied for enrichment assessment of regulatory features (default reference database) in defined genomic intervals centered in the TSSs of pleiotropic *RNYs* and the results compared with equivalent intervals of non-pleiotropic *RNYs*. The RNA repeat genome annotations were obtained from RepeatMasker (hg19, version 2020-02-20).

### Phylogenetic analysis

Human *RNY*-related sequences were downloaded from BioMart (version 3.17), FASTA files compiled using *readDNAStringSet* in Biostrings (version 3.17), and sequences aligned using *msa* and ClustalW [54], and stored as.DNAbin and DNAStringSet (version 5.7) in APE [55]. The *msaplot* function in *ggtree* [56], *ggplot2* [57], and *dist.dna* in APE [55] were used to construct and visualize the phylogenetic tree. The pairwise sequence distance was computed using the K80 model [58]. The phylogenetic tree was estimated using the *nj* function implemented in APE [55].

### Gene expression data

Data from The Cancer Genome Atlas (TCGA) were obtained via the Genomic Data Commons Data Portal (https://portal.gdc.cancer.gov) and gene expression information corresponded to FPKM-UQ values. The expression signature scores were computed using the single-sample GSEA (ssGSEA) algorithm calculated with GSVA software [59] (version 1.42.0). Analysis and visualization were carried out using the *ggplot2* [57] (version 3.3.5), *complexHeatmap* [60] (2.10.0), *circlize* [61] (version 0.4.13), and R *base* graphs (version 4.1.2) packages. To estimate the expression correlations empirically, 1000 sets of randomly selected ncRNA genes with the same length as the pleiotropy *RNY* set were selected, computed in ssGSEA, and analyzed to establish any association with age at diagnosis (TCGA clinical data annotation). The sRNA-seq data of plasma and the clinical and individual information from the corresponding healthy donors and cancer patients were downloaded from the exRNA Atlas [62]: Gene Expression Omnibus reference GSE71008 [28]. The difference in the levels of expression between the *RNY* signatures was examined using the Mann–Whitney test.

### Cell-free plasma small-RNA library preparation, sequencing, and analysis

The genetic and clinical data of the two sample sets analyzed are detailed in Additional file 1: Tables S2 and S3. Plasma small RNAs were isolated using the Plasma/Serum Circulating and Exosomal RNA Purification Mini Kit (51000, Norgen Biotek) and washed and concentrated using the RNeasy MinElute Cleanup Kit (74204, QIAGEN). For plasma collected in heparin tubes (used in the prospective study), the RNA samples were further purified using a heparinase-based protocol [63]. RNA concentration was measured using the Quant-it™ RiboGreen RNA Assay Kit and RiboGreen RNA Reagent (R11490, Thermo Fisher Scientific). Perkin Elmer’s NEXTFLEX Small RNA-seq v3 kit (NOVA-5132-06) was used to prepare the small RNA libraries, with slight modifications to the manufacturer’s protocol: up to 5 ng of total RNA was denatured at 70°C, and subjected to 3′ ligation using 0.5x diluted adenylated adapter for 2 h at 25°C. NEXTFLEX Cleanup Beads were used to remove excess adapter. The adapter inactivation step was skipped, and 5′ ligation was carried out with 0.5x diluted adenylated adapter. After cDNA synthesis and another bead cleanup, samples were PCR-amplified with UDI primers for 18 cycles. Finally, libraries were size-selected by gel electrophoresis. Samples were separated on 6% polyacrylamide gels, stained with SYBRgold, and bands of interest were excised, minced, and incubated in water overnight, with constant agitation. Gel-extracted libraries were treated with a DNA Clean and Concentrate kit (D4014, Zymo) following the manufacturer’s instructions. Library size and concentration were determined with an Agilent 2100 Bioanalyzer, using a High Sensitivity DNA kit. Libraries were then pooled equimolarly, and the pool was quantified with KAPA SYBR FAST Universal qPCR Kit (KK4824) and loaded at 3.8 pM with 5% PhiX spike-in. Sequencing was done with Illumina’s NovaSeq 6000 apparatus, using v1.5 SP 100 cycle reagents with XP workflow. Sequencing data were demultiplexed using Illumina’s *bcl2fastq* software to generate *fastq* files for each sample. Samples were analyzed with the exceRpt small RNA pipeline [64] using the option to trim 4 bp from the 5′ and 3′ ends of the sequencing data, as specified by PerkinElmer.

## Results

### Blood traits associated with cancer diagnosis

**Fig. 1:**
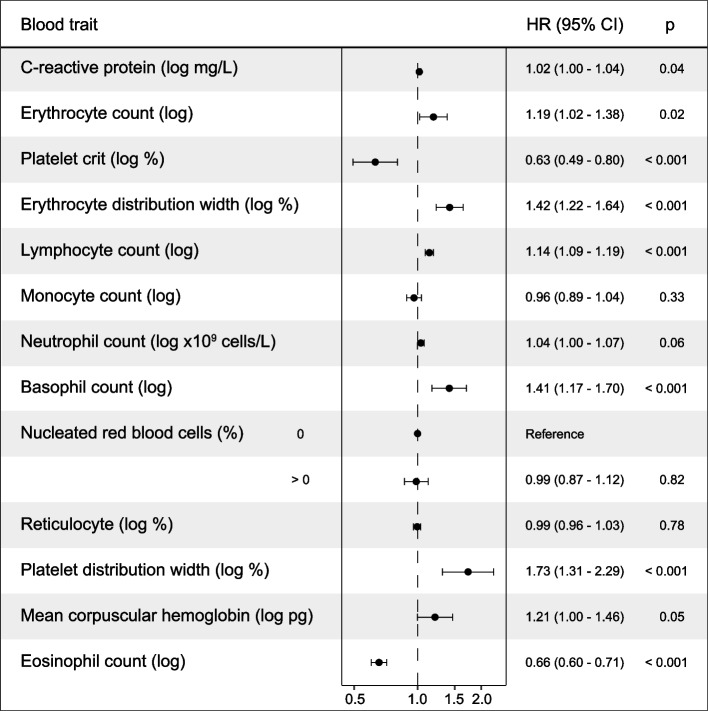
Study of association of blood traits with cancer diagnosis. Forest plot showing the associations between blood traits and cancer diagnosis in the UK Biobank (*n* = 364,791). The trait units, HR, 95% CI, and significance (*p*) of the multivariate Cox proportional model are indicated. The dataset was filtered, blood traits log_2_-transformed, and regression models stratified and adjusted as described in the “Methods”

Systemic alteration of specific immune cell types may enable cancer development [65]. We analyzed the association between blood traits and cancer diagnosis in the prospective cohort of the UKBB [24, 25]. After data filtering and quality control (Methods), the normalized blood trait measures of 364,791 individuals were examined for associations with cancer diagnosis using a Cox proportional hazard model that included individual and biological covariates. To prevent confounding effects from hidden tumors, the analysis was limited to individuals with a first cancer diagnosed >12 months after a basal blood test, and without considering benign neoplasms. As in previous studies [66], the C-reactive protein (CRP) was found to be associated with increased risk of cancer, although with a marginal effect: hazard ratio (HR) = 1.02, 95% CI 1.00–1.04, *p* = 0.035 (Fig. 1 and Additional file 1: Table S4). Individuals with an indication of an acute inflammatory condition (CRP > 10 mg/L) were excluded from the analysis. Then, five blood traits were found to be significantly associated with increased risk of cancer: counts of lymphocytes (HR = 1.14, 95% CI 1.09–1.19, *p* < 0.001), erythrocytes (HR = 1.19, 95% CI 1.02–1.38, *p* = 0.025), and basophils (HR = 1.41, 95% CI 1.17–1.70, p < 0.001), and the distribution widths of erythrocytes (HR = 1.42, 95% CI 1.22–1.64, *p* < 0.001) and platelets (PDW: HR = 1.73, 95% CI 1.31–2.29, *p* < 0.001). In turn, two blood traits were found to be significantly associated with reduced risk: eosinophil count (HR = 0.66, 95% CI 0.60–0.71, *p* < 0.001) and platelet crit (PC: HR = 0.63, 95% CI 0.49–0.80, *p* < 0.001; Fig. 1 and Additional file 1: Table S4). The contrary effects of PDW and PC were consistent with a predictable negative correlation of these measures, and the association between platelet activation—inferred from the high PDW—and increased cancer risk might be akin to the role of this feature in tumor growth and invasion [67]. A subsequent sensitivity analysis of diagnoses within the first year after the basal blood test showed a greater effect of CRP (HR = 1.15, CI 1.10–1.20, *p* < 0.001), and predictable cancer associations with conditions analogous to anemia, indicated by cancer-risk associations with low erythrocyte count (HR = 0.54, 95% CI 0.36–0.81, *p* = 0.003) and low mean corpuscular hemoglobin concentration (HR = 0.38, 95% CI 0.23–0.63, *p* < 0.001; Additional file 1: Table S5; and Additional file 2: Fig. S1).

Analysis of cancer diagnosis >12 months after the blood test and stratified by sex showed similar results to those from the complete cohort, except for indications of a higher cancer risk linked to high neutrophil counts in women, and a lower cancer risk linked to low monocyte counts in men (Additional file 1: Tables S6 and S7). Stratified analyses for the most common cancer types (breast, colon, lung, and prostate; Additional file 1: Table S8) showed greater heterogeneity in the predicted effects of the blood traits, except for eosinophil counts, which were found to be significantly associated with a lower risk of the four cancer types (Additional file 1: Tables S9-S12). An inverse relationship between eosinophils and colorectal cancer incidence had been previously noted [68], and analogous trends towards a protective association were suggested for prostate and lung cancer risk [30, 69]. The data suggest that interindividual differences in systemic immune cell levels influence cancer risk; however, the genetic factors and biological processes underlying pleiotropism are mostly unknown.

### Lack of global genetic correlation between blood traits and cancer risk

Host and exposome factors can alter the function of the immune system and thereby influence cancer risk [70]. Since blood traits are strongly determined by common genetic variation [24, 25], we examined the shared genetic basis of blood traits and cancer risk. We analyzed the GWAS results of 27 blood traits [24, 25] and of the risk of 27 cancer types and subtypes (subtypes of breast cancer; Additional file 1: Tables S1). After data processing and quality control analyses of the summary statistics, genetic correlations were computed using the HapMap3 [71] catalog of SNPs. Consistent with the original UKBB study [24], approximately 50% (177/351) of the pairwise comparisons of blood traits showed significant genetic correlations (FDR-adjusted *p* < 0.05; Additional file 2: Fig. S2a). By contrast, few significant genetic correlations were identified in the cancer-risk analyses, and these were only detected among the overall and subtype-specific breast cancer studies, and for the breast-colon, breast-cervix, and colon-rectum comparisons (Additional file 2: Fig. S2b). Two GWASs were included for the analysis of breast cancer: BC#1 refers to the results from the Breast Cancer Association Consortium (BCAC) [72], including subtype analyses [26]; and BC#2 refers to the results from the UKBB [73] (Additional file 1: Tables S1). Next, analysis of the genetic correlation between blood traits and cancer risk did not reveal any significant associations (FDR-adjusted). A few nominally significant correlations were indicated, including lung cancer with white blood cell (leukocyte) counts (Additional file 1: Table S13; and Additional file 2: Fig. S2c), which was consistent with an independent observation in the UKBB [69]. Therefore, the genetics of blood traits and cancer risk are not globally correlated in the same direction when considering > 5 million variants, although pleiotropic signals might exist at specific loci.

### Identification of blood trait–cancer pleiotropic variants

**Fig. 2:**
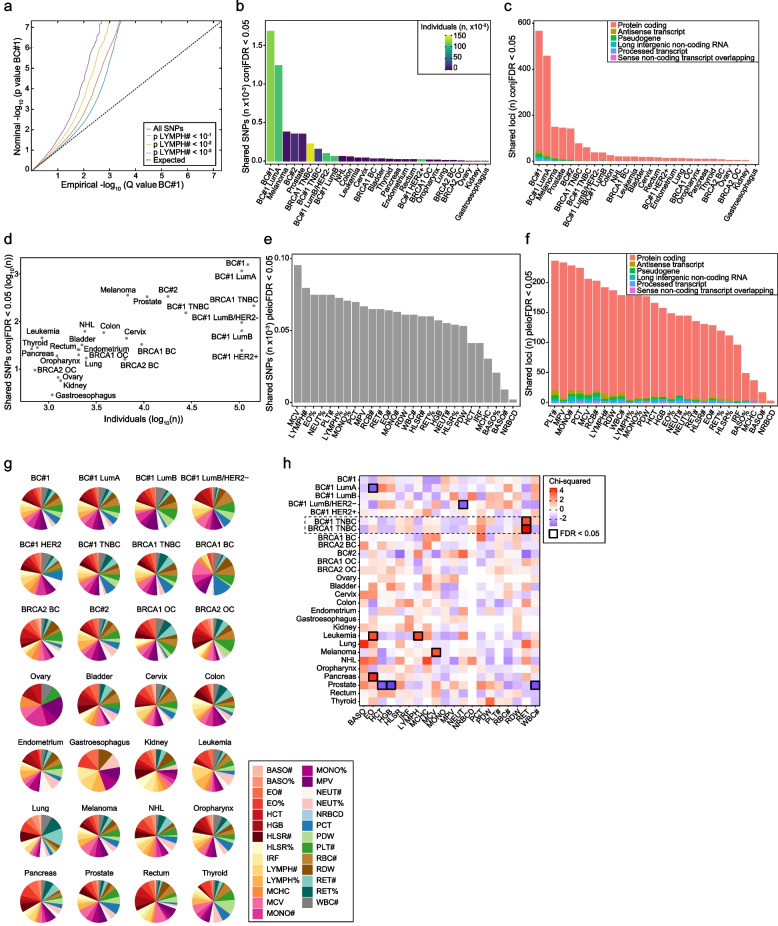
Shared genetics of blood traits and cancer risk. **a** Stratified Q-Q plot for breast cancer risk (BC#1) as a function of the significance of SNP associations with LYMPH#, as indicated in the inset. The dotted line indicates no association. **b** Histogram depicting the number of variants (*n* ×10^−3^; conjFDR < 0.05) shared between cancer risk and blood traits. The colored bar indicates the number of individuals originally included in each cancer GWAS, as denoted in the inset. **c** Histogram depicting the distribution of classes of genetic elements (denoted in the inset) across the identified pleiotropic loci and cancer studies. **d** Plot depicting the relationship between the number (*X*-axis; log_10_) of individuals in each GWAS analyzed and the number of identified pleiotropic variants (conjFDR < 0.05; log_10_). **e** Histogram depicting the number of variants (*n* ×10^−3^; conjFDR < 0.05) shared by blood traits and cancer risk. **f** Histogram depicting the distribution of classes of genetic elements (denoted in the inset) across the identified pleiotropic loci and blood traits. **g** Pie charts showing the contribution of each blood trait to each cancer risk study based on the number of shared variants. Color-coded blood trait acronyms are depicted in the inset. **h** Heatmap showing the overrepresentation and underrepresentation of shared blood-trait variants for each cancer study. The significant associations (FDR-adjusted *p* < 0.05) are indicated by black-bordered squares

To identify the genetic factors shared by blood traits and cancers, we examined Q-Q plots stratified by SNP significance and conditioned for the corresponding blood trait or cancer type. Each cancer type showed evidence of deviation from expectation for an association with one or more blood traits (Additional file 2: Fig. S3). To evaluate deviation from expectation, genomic inflation scores were computed. Evidence of shared genetics (lambda > 1) was obtained in 400 blood trait–cancer risk comparisons (Additional file 1: Table S14). An example of the evidence for shared genetics, the comparison between BC#1 and “lymphocyte count” (LYMPH#) at three SNP significance thresholds (LYMPH# *p* < 10^−1^, 10^−2^, and 10^−3^) and for all SNPs, is shown in Fig. 2a.

Next, the condFDR/conjFDR method [39, 74] was used to leverage and identify genetic associations between blood traits and cancer risk. With a conjFDR < 0.05, 4093 pleiotropic variants were identified, ranging from 3 to 1689, associated with gastroesophageal cancer and BC#1, respectively (Fig. 2b and Additional file 1: Table S15). Analyses of breast and prostate cancer included the data solely for females and males, respectively. The causal gene for a genetic association is often the closest gene to the specific variant [75, 76]. Next, mapping the variants to genetic elements using BioMart annotations [42] identified a range from 0 (gastroesophageal cancer) to 560 (BC#1) protein-coding genes, and relatively minor contributions from other elements (Fig. 2c). As expected, the larger cancer studies revealed more pleiotropic associations, with the exception of HER2-positive breast cancer, which yielded only 26 variants; in contrast, the melanoma and prostate studies showed comparatively more pleiotropic associations (385 and 356 variants, respectively) (Fig. 2b,d and Additional file 1: Table S15).

From the perspective of blood traits, mean corpuscular volume (MCV) and platelet count (PLT#) showed the greatest number of shared genetic variants and pleiotropic gene candidates (i.e., genes mapped to pleiotropic variants), respectively, while nucleated red blood cells showed the weakest evidence of pleiotropy (Fig. 2e,f and Additional file 1: Table S15). Despite these profiles, all blood traits were linked to cancer risk to some extent (Fig. 2g). Subsequent grouping of blood traits by immune cell type identified specific overrepresentation and underrepresentation (FDR < 0.05) of shared variants with cancer risk. For instance, a significant enrichment of shared variants was found between reticulocytes and triple-negative breast cancer (TNBC) (Fig. 2h). Therefore, it may be concluded that broad perturbations of blood cells might influence cancer risk, although the specific processes remain to be determined.

### Pleiotropism is partially linked to telomere length control

**Fig. 3:**
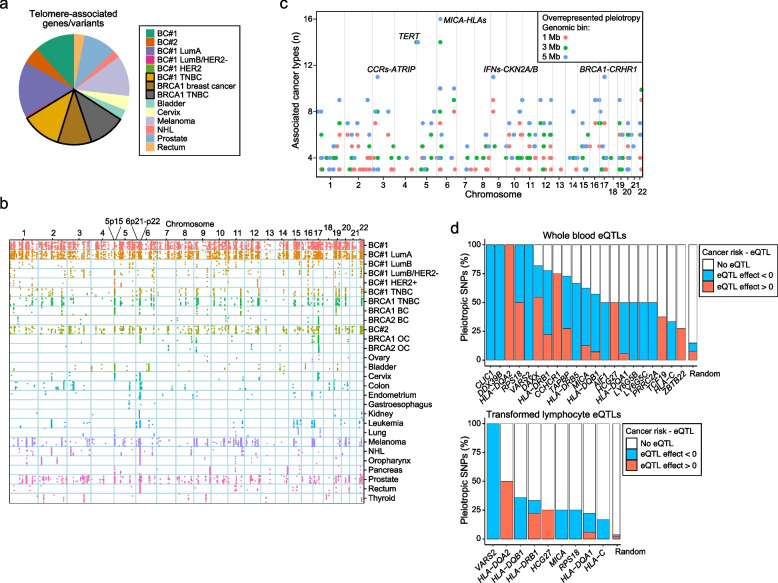
Link of pleiotropism with telomere length regulation and genomic hotspots. **a** Pie charts showing the contribution of pleotropic variants in telomere length-associated gene loci across the cancer studies. The proportion of variants associated with breast cancer caused by *BRCA1* pathological variants and/or TNBC is denoted by solid triangles, as indicated in the inset. **b** Genomic diagram showing the relative position of the pleiotropic variants (dots) across human chromosomes 1–22 (*X*-axis) and cancer-risk studies (*Y*-axis). **c** Graph showing the identified pleiotropic hotspots across human chromosomes 1–22. Results are shown for the regions including associations with > 2 cancer types and corresponding to genomic bins of 1, 3, and 5 Mb, as indicated in the inset. The hotspots including > 10 cancer trait associations are denoted by candidate gene names. **d** Histograms showing the percentage of the 6p21-p22 pleiotropic variants identified as cis-eQTL in whole blood (left panel) or immortalized lymphocytes (right panel) of the corresponding 6p21-p22 genes (*X*-axis). The direction of the eQTL effect is defined by the slope color (inset). The indicated genes showed significant enrichment (FDR-adjusted *p* < 0.05) of pleiotropy-eQTL correspondences relative to equivalent randomly chosen variants in 1000 gene loci expressed in all major immune cell types

A previous study of pan-cancer pleiotropy—not considering blood traits, but including a meta-analysis of cancer GWAS UKBB results—identified 85 leading variants that influenced two or more cancer types in the same direction [73]. Our blood trait–cancer pleiotropy study identified nine variants in this set (Additional file 1: Table S16), which represents a highly significant overlap if an equivalent genome coverage is assumed: identifying nine pleiotropic variants among sets of 85 variants against a background of approximately 5 million variants has a significance of *p*_hypergeometric_ = 1 × 10^−19^. The nine pleiotropic variants were found to be associated with 17 blood traits and nine cancer types. The corresponding gene candidates included the telomerase RNA component (*TERC*), which had previously been shown to be associated with leukocyte telomere length [43] and the risk of diverse cancer types [77]. Following on from this observation, we identified a significant overlap of 20 genes that were linked to leukocyte telomere length [45] and that mapped to the 4093 pleiotropic variants (total pleiotropic gene candidates *n* = 1228; *p*_hypergeometric_ = 0.001). In addition to the *TERC*, the pleiotropic gene set included the telomerase reverse transcriptase (*TERT*) and the regulator of telomere elongation helicase 1 (*RTEL1*; Additional file 1: Table S17). Next, analysis of the proportion of pleiotropic variants linked to genes associated with leukocyte telomere length revealed an enrichment in breast cancer caused by pathological variants of *BRCA1* and TNBC (32% of variants), followed by luminal A breast cancer (LumA; 16%) and melanoma (12%; Fig. 3a). Intriguingly, luminal progenitors, the cells of origin of *BRCA1*-associated breast tumors [78], are particularly sensitive to telomere dysfunction [79]. Therefore, more than 4000 variants concurrently influence one or more blood trait and cancer risk, and regulation of telomere length in immune and/or epithelial cells might underlie this pleiotropism.

### Hotspots of blood trait–cancer pleiotropism are present in the TERT and HLA regions

Examining the location of pleiotropic variants throughout the genome indicated regions with a relatively high frequency of associations (Fig. 3b). Analysis of the representation of pleiotropic associations relative to all examined variants in genomic bins of 1, 3 and 5 megabases (Mb) identified 81–159 regions with a significant pleiotropy enrichment (chi-squared test FDR-adjusted *p* < 0.05; Fig. 3c and Additional file 1: Table S18). The genomic bins comprising associations with > 10 cancer types corresponded to the chromosomes 3p21, 5p15, 6p21-p22, 9p21, and 17q21, which, among other genes, encompass CC-motif chemokine receptors, *TERT*, human leukocyte antigens, interferons, and corticotropin-releasing hormone receptor 1, respectively (Fig. 3d).

The chromosome region with the greatest number of cancer associations (*n* = 16) corresponded to 6p21-p22 (chromosome bin from 30 to 35 Mb; Additional file 1: Table S18). To assess the regulatory impact of the pleiotropic variants identified in this hotspot, we analyzed the correspondence with expression quantitative trait locus (eQTL) identified in whole blood and transformed lymphocytes [47], and compared the observed eQTL frequencies with those of randomly selected genetic variants (European MAF > 0.01) across different LD thresholds: *r*^*2*^ < 0.2, 0.2–0.8, and > 0.8) from 1000 randomly chosen genes that were substantially expressed (TPM > 1) in all major immune cell types [48]. Thus, pleiotropic variants in 21 genes of chromosome 6p21-p22 were frequently found to be eQTLs in blood cells and/or lymphocytes (FDR < 0.05; Fig. 3d). Alteration of the regulation of some of these genes might therefore determine blood-cancer pleiotropism. The candidates include five *HLAs* and the major histocompatibility complex (MHC) class I polypeptide-related sequence A (*MICA*) genes.

### Pleiotropic factors are frequent regulators of hematopoiesis and myeloid lineage

**Fig. 4:**
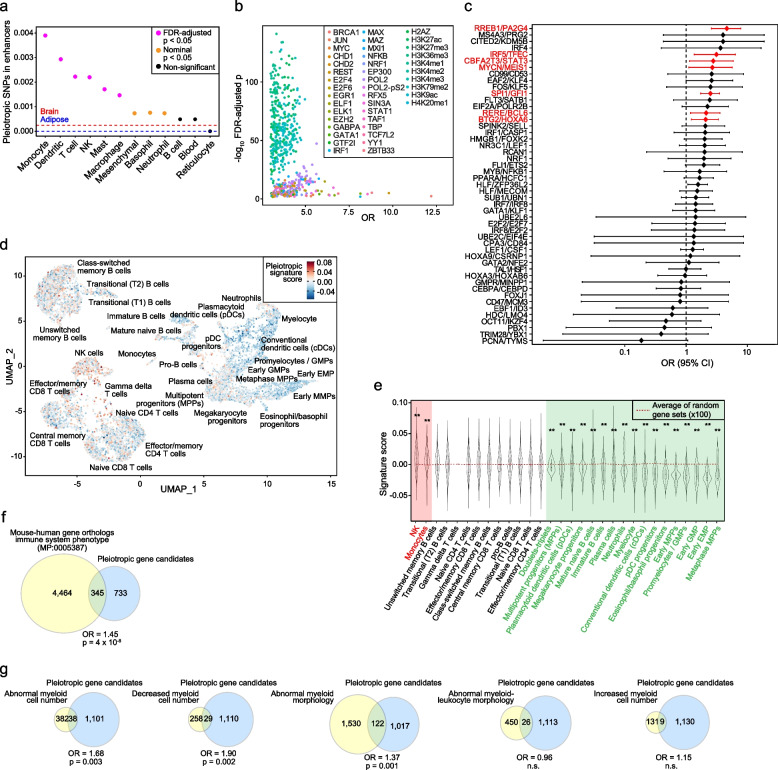
Link between pleiotropic gene candidates and hematopoiesis. **a** Graph showing the proportion of pleiotropic variants (all cancers included) mapped in enhancers from immune cell types and blood (*X*-axis). The pink dots indicate significant overlap, as indicated in the inset. The variant-enhancer overlap proportions in brain and adipose tissue are indicated by red and blue horizontal dashed lines, respectively. **b** Graph showing the overrepresented (−log_10_ FDR-adjusted *p*) genomic regulatory features (binding of transcription factors and defined histone marks, denoted in the inset) in the genomic sequences centered (± 10 base pairs) on the identified pleiotropic variants (*n* = 4,093). **c** Forest plot showing the OR and 95% CI of the overlap between the pleiotropic gene set and hematopoiesis gene modules, depicted by the corresponding master regulators (*Y*-axis). Red bars indicate significant overlap. **d** Uniform Manifold Approximation and Projection (UMAP) of the pleiotropic gene signature expression (score indicated in inset) in the bone marrow single-cell RNA sequencing profiles. Cell clusters are annotated. **e** Violin plot showing the distribution of the pleiotropic signature expression score in each bone marrow cell type (*X*-axis). The horizontal line corresponds to the average score of 100 random equivalent gene sets. The asterisks indicate a significant expression difference in the pleiotropic gene signature relative to equivalent random gene sets (**p_empirical_ < 0.01). **f** Venn diagram showing the overlap between mouse gene orthologs that, when mutated, cause immune system alterations (MP:0005387; “immune system phenotype”) and the pleiotropic gene set (all cancers included). The OR and significance (*p*_hypergeometric_) are indicated. **g** Venn diagrams showing the overlap between mouse gene orthologs linked to myeloid cell alterations (phenotypes are indicated) and the pleiotropic gene set (all cancers included). The OR and significance (*p*_hypergeometric_) value are indicated; n.s., not significant

Telomere dysfunction alters hematopoiesis [80]. To assess the connection between pleiotropy and immune cell regulation further, we analyzed the genomic location of the pleiotropic variants in relation to enhancers identified in immune cell types and whole blood, and compared the results with those of enhancers from predicted unrelated tissue origins (adipose and brain) [81]. In six of the 12 (50%) immune cell types analyzed, the proportion of pleiotropic variants mapped to defined enhancers was significantly higher than expected, with the highest pleiotropic enrichment for enhancers in monocytes (FDR-adjusted *p* < 0.05; Fig. 4a). Next, we analyzed the occurrence of DNAse I hypersensitivity and transcription factor binding sites, and epigenetic marks [53, 82, 83], in the genomic regions encompassing the positions of the identified pleiotropic variants ± 10 base pairs, and compared the observed frequency of regulatory features with that of equivalent regions in 100,000 randomly chosen variants (European MAF > 0.05). Several transcription factors were found to be overrepresented in the pleiotropic set, including some of those involved in hematopoiesis (EGR1, GATA1, and IRF1; Fig. 4b and Additional file 1: Table S19). The regulatory features with the greatest overrepresentation in the pleiotropic variants were the binding of RNA polymerase II (POL2) and the tri-methylation of the fourth lysine residue of histone H3 (H3K4me3), which marks transcription start sites of active genes (Fig. 4b and Additional file 1: Table S19).

We further evaluated the pleiotropic connection with master regulators of hematopoiesis. Considering the 62 curated regulators identified in the literature (Additional file 1: Table S20), 18 gene loci (29%) were found to be identified with pleiotropic variants, a significantly higher proportion than expected, given the proportion among all protein coding genes: OR = 5.0; p_hypergeometric_ = 9 × 10^−9^. The occurrence of the candidate pleiotropic genes in the gene expression modules that portray a hematopoiesis cell hierarchy [84] was then examined. This analysis revealed a significant overlap of the pleiotropic gene set with seven modules (FDR-adjusted *p*_hypergeometric_ values < 0.05; Fig. 4c and Additional file 1: Table S21), including a module regulated by the canonical myeloid lineage factor SPI1, also known as PU.1 [85].

Next, we analyzed the profile of the pleiotropic gene set in the cell states of the hematopoietic system [86]. The signature of the pleiotropic gene set was found to be underexpressed in several progenitor cell states (Fig. 4d). Comparison of the pleiotropic signature against 100 equivalent randomly chosen gene sets (random genes among those expressing TPM > 1 in all major immune cell types [48]) confirmed significant underexpression in progenitor cell populations (Fig. 4e). The pleiotropic gene set appeared to be particularly strongly underexpressed in myeloid progenitor cell populations, including granulocyte–monocyte progenitors (GMPs), erythro-myeloid progenitors (EMP), and multipotent progenitors (MPPs) (Fig. 4e). Indeed, the pleiotropic gene set was found to have an overrepresentation of regulators of myeloid leukemia [87]: *DOT1L*, *EP300*, *FLI1*, *GSE1*, and *MED24* (OR = 7.1; *p*_hypergeometric_ = 4 × 10^−4^). In addition, there was an overrepresentation (OR = 3.7; *p*_hypergeometric_ = 5 × 10^−4^) of genes that have been associated with clonal hematopoiesis through germline variation [88]. These included *ATM*, *CHEK2*, *LY75*, *PARP1*, *TERT*, *TET2*, *THADA*, *TP53*, and *ZNF318*.

Following on from the indication that perturbed hematopoiesis is linked to blood trait–cancer pleiotropism, the pleiotropic gene set was found to have an overrepresentation of mouse orthologs that cause immune system alterations when mutated or altered by allelic variants [89] (Mammalian Phenotype ontology code MP:0005387; Fig. 4f). A detailed analysis of the five ontology terms corresponding to myeloid cell alterations revealed three of them to be significantly overrepresented in the pleiotropic gene set: “decreased myeloid cell number”, “abnormal myeloid cell number,” and “abnormal myeloid cell morphology” (Fig. 4g). Therefore, the genes predicted to influence blood trait–cancer pleiotropism are frequently associated with regulating hematopoiesis and progenitor cell states, leading to potential alterations of the myeloid lineage.

### High frequency of pleiotropic variants in loci containing Y-RNA-related sequences

**Fig. 5:**
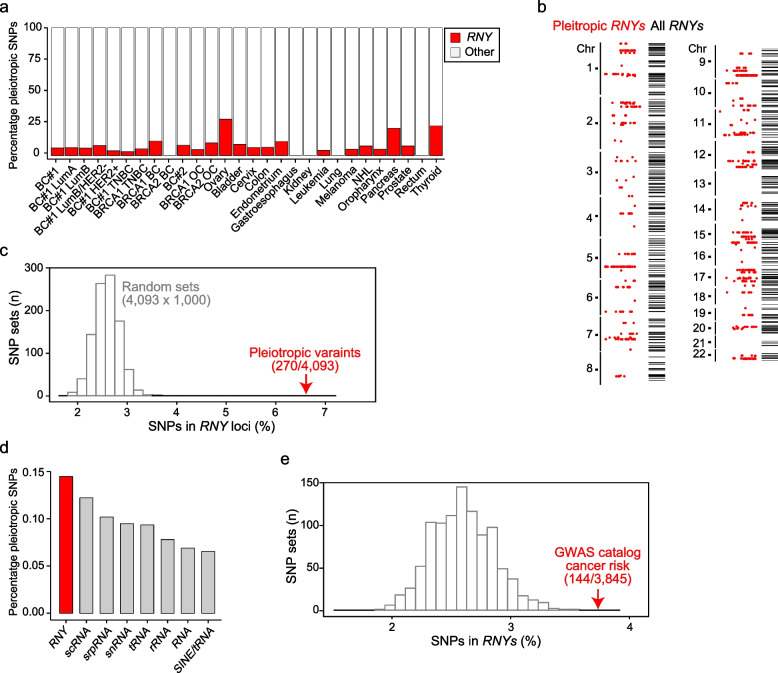
High frequency of pleiotropic variants in *RNY*-containing loci. **a** Histogram showing the relative contribution of pleiotropic variants (%; *Y*-axis) in *RNY*-containing loci (± 50 kb centered on each variant) across cancer studies (*X*-axis). **b** Genomic distribution of pleiotropic variants in *RNY*-containing loci (red dots) and all *RNY*-containing loci (horizontal bars) from chromosome (chr) 1 to 22. **c** Graph showing the percentage of variants (SNPs) mapped to *RNYs* (± 50 kb) in 1000 random sets of 8155 SNPs (European MAF > 0.01 and *r*^*2*^ < 0.8) and the observed percentage in the blood trait–cancer pleiotropy set (6.6%; 270/4,093). **d** Histogram showing the distribution of identified RNA repeat elements across the pleiotropic loci (4093 variants; ± 50 kb). The families of repeat elements are indicated (*X*-axis). **e** Graph showing the percentage of variants (SNPs) mapped to *RNYs* (± 50 kb) in 1000 random sets of 3847 SNPs (no filter criteria) and the observed percentage in the GWAS catalog of cancer risk variants (3.7%; 144/3,845)

The human genome has four functional Y-RNAs (*RNY1*, *2*, *3*, and *5*), which are a class of small noncoding RNAs that bind and regulate Ro60 [90–92], a protein involved in the cell’s response to stress and one identified as an autoantigen in autoimmune diseases [93]. Detailed examination of the pleiotropic loci identified numerous *RNY* genes, pseudogenes, and derived sequences (total *n* = 118) mapped in a region ± 50 kb from the pleiotropic variants across the cancer studies, with the exception of three settings: breast cancer caused by pathological variants in *BRCA2*, and gastroesophageal and kidney cancers (Fig. 5a). The *RNY*-containing loci were identified by mapping 270 pleiotropic variants (6.6% of the total 4,093 variants). They included *RNY1* and *RNY3*, four *RNY4* pseudogenes, and 112 miscellaneous Y-RNA sequences (Additional file 1: Table S22). There was no difference in the genomic distribution of the *RNY*-containing pleotropic loci relative to all human *RNY*-derived sequences (Kolmogorov–Smirnov test *p* > 0.05; Fig. 5b). Then, the percentage of pleiotropic variants linked to *RNY* sequences was significantly higher than the expectation based on 1000 sets of 4093 randomly chosen variants—European MAF > 0.01 and *r*^*2*^ < 0.8 in any pair— and considering 767 *RNY* sequences annotated in the human genome, from chromosome 1 to 22, for which an average 2.8% of random variants mapped to *RNY* loci (*p*_empirical_ < 0.001; Fig. 5c). Indeed, among the established families of small noncoding RNAs, *RNY* sequences showed the closest concordance with pleiotropic loci (Fig. 5d).

Two breast cancer associations were previously predicted to target *RNY*-derived transcripts [18], and we identified these variants as being pleiotropic: rs12962334 in chromosome 18q11, which potentially targets Y-RNA ENSG00000223023; and rs1061657 in chromosome 12q24, which potentially targets Y-RNA ENSG00000199220. In addition, the study of pan-cancer pleiotropism [73] identified a potential pleiotropic *RNY* transcript in chromosome 2q14, ENSG00000201006. To assess the link between cancer risk and *RNY* sequences further, we analyzed the catalog of GWAS results [94]. Of the 3847 variants associated with cancer risk and mapped between chromosomes 1 to 22, 142 (3.7%) were found in the vicinity of an *RNY* sequence (± 50 kb; Additional file 1: Table S23). Notably, this percentage was significantly higher than expected from a consideration of 1000 sets of 3847 randomly chosen variants (dbSNP build 154; *p*_empirical_ < 0.001; Fig. 5e). We conclude that an excess of blood trait–cancer pleiotropic variants is located near *RNY* sequences, including functional *RNYs*, pseudogenes, and derived sequences.

### Pleiotropic RNYs show specific regulatory features and relative overexpression

**Fig. 6:**
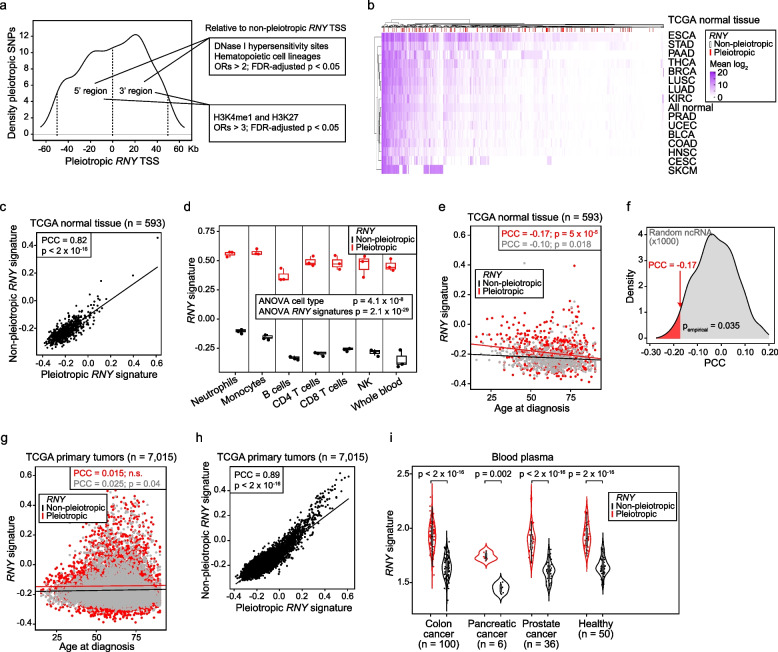
Regulatory features and relative overexpression of pleiotropic *RNYs*. **a** Density distribution of the pleiotropic SNPs identified nearby (± 50 kb) *RNY* TSSs. The 5′ and 3′ 50-kb regions are delimited by vertical dashed lines. Genomic regulatory features found to be significantly enriched in each region are denoted in boxes. **b** Unsupervised hierarchical clustering of the average expression level of each pleiotropic and non-pleiotropic *RNY* transcript (as depicted in the inset) across normal tissue from TCGA (study acronyms are depicted on the Y-axis). **c** Scatter plot of the expression correlation between the pleiotropic and non-pleiotropic *RNY* signatures across normal tissue from TCGA. The PCC and corresponding significance (*p*) are indicated. **d** Box plots showing of the pleiotropic and non-pleiotropic *RNY* signature scores across primary immune cell populations isolated from whole blood. The two-way ANOVA comparisons and significance (*p*) are indicated. **e** Scatter plot of the correlation (PCC and *p* are indicated) between the pleiotropic or non-pleiotropic *RNY* expression signatures and age at diagnosis of cancer, using the corresponding normal tissue TCGA data. **f** Density distribution of the PCCs between equivalent random sets of microRNAs and age at diagnosis of cancer, using the normal tissue TCGA data (*n* = 593). The observed PCC for the pleiotropic *RNY* expression signature is indicated by an arrow, and the significant PCC tail and *p*_empirical_ threshold are denoted. **g** Scatter plot of the correlation (PCC and *p* are indicated) between the pleiotropic or non-pleiotropic *RNY* signatures and age at diagnosis of cancer, using primary tumor TCGA data. **h** Scatter plot of the expression correlation between the pleiotropic and non-pleiotropic *RNY* signatures across TCGA primary tumors. The PCC and corresponding significance (*p*) are indicated. **i** Violin plot of the expression level of the pleiotropic and non-pleiotropic *RNY* signatures in blood plasma from cancer patients and healthy individuals, as indicated on the *X*-axis. Significance of the Wilcoxon rank test comparing the two signatures in each setting is shown

The pleiotropic variants identified in *RNY*-containing loci were found to be relatively highly concentrated around the corresponding transcription start sites (TSSs) and 3′ regions (Fig. 6a). Only one pleiotropic variant (rs10193900) mapped within a transcribed *RNY*: the *RNY1*-derived sequence, ENSG00000201160 (Additional file 2: Fig. S4). To further determine the functionality of the pleiotropic *RNYs*, we analyzed the occurrence of DNAse I hypersensitivity sites and epigenetic marks [53, 82, 83] in the regions encompassing the corresponding TSSs ± 50 kb and compared the observed frequency of regulatory elements with equivalent regions in the non-pleiotropic *RNY* loci (*n* = 698). The 5′ and 3′ regions of the pleiotropic *RNYs* were found to be significantly enriched in DNase I hypersensitivity sites identified in several cell lineages [82], including hematopoietic: ORs > 2; FDR-adjusted *p* < 0.05 (Fig. 6a and Additional file 1: Table S24). Both regions were also found to be significantly enriched in the enhancer-linked histone marks H3K4me1 and H3K27ac [83], observed in >1 assays (ORs > 3; FDR-adjusted *p* < 0.05) (Fig. 6a and Additional file 1: Table S24).

Consistent with marks of active transcription and enhancers, the average expression value of the pleiotropic *RNY*s in normal tissue was found to be higher than that of non-pleiotropic *RNYs*, established from the data from 15 studies included in TCGA [95] (tissue samples *n* = 593; Wilcoxon rank-sum *p* = 0.014; Fig. 6b). This difference in expression was detected despite the positive correlation between the pleiotropic and non-pleiotropic *RNY* transcript sets (hereafter “signatures”): Pearson’s correlation coefficient (PCC) = 0.82, *p* < 2 × 10^−16^ (Fig. 6c). Then, analysis of the *RNY* signatures in blood cell populations of neutrophils, monocytes, B, CD4 T, CD8 T, and natural killer cells [96] corroborated the overexpression of the pleiotropic set, and further indicated higher levels of this signature in myeloid relative to lymphoid cell types (2-tailed *t*-test *p* = 0.0003; Fig. 6d).

Analysis of the *RNY* signatures in normal tissue of TCGA showed a negative correlation with age at diagnosis for both, although it was stronger for the pleiotropic set: PCC = −0.17 *vs.* −0.10; *p* = 5 × 10^−5^ and 0.018, respectively (Fig. 6e). An analogous analysis using 1000 signatures of equivalent randomly selected sets of microRNAs in TCGA indicated that the negative correlation between age at diagnosis and the pleiotropic *RNY* signature was significant (*p*_empirical_ = 0.035; Fig. 6f). Multivariate logistic regression including patient sex, cancer type and subtype, and tumor stage (matched with the normal tissue analyzed) confirmed the negative correlation between the pleiotropic *RNY* signature and age at diagnosis: *β* = −0.10, *p* = 0.025. The analysis stratified by TCGA study was limited by the sample sizes, but reached nominal significance for the pleiotropic *RNY* signature in normal breast and esophageal tissue (*n* = 112 and 12, respectively; the non-pleiotropic *RNY* signature was also found to be significantly correlated in esophageal tissue; Additional file 2: Fig. S5). By contrast, the *RNY* association with age at diagnosis was not observed in the expression profiles of primary tumors (Fig. 6g), regardless of the high positive correlation between the two *RNY* signatures (PCC = 0.89, *p* < 2 × 10^−16^; Fig. 6h).

Products derived from processing *RNY* transcripts are highly abundant in body fluids and their relative overexpression has been noted in the plasma of cancer patients [27, 28, 97–100]. A large fraction of circulating *RNY* products might be derived from the *RNY4* pseudogenes [101], but phylogenetic analysis did not detect an association between *RNY4*-derived sequences and pleiotropic identification in *RNYs* (Additional file 2: Fig. S6). Subsequent examination of public plasma RNA profiles of healthy individuals and cancer patients [28] confirmed the significant overexpression of the pleiotropic *RNY* signature relative to the non-pleiotropic set (Fig. 6i). Therefore, blood trait–cancer pleiotropic variants are frequently located relatively close to *RNY* sequences, which are differentially regulated, and tend to be overexpressed in normal tissue and blood plasma of cancer patients.

### Pleotropic RNYs linked to loci influencing systemic lupus erythematosus

**Fig. 7:**
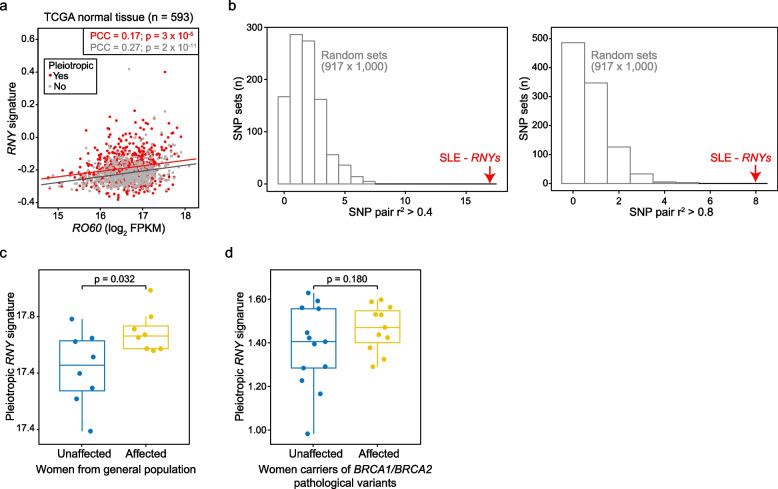
Pleiotropic *RNYs* are linked to SLE risk and plasma *RNYs* are relatively abundant preceding breast cancer diagnosis. **a** Scatter plot of the correlation of the levels of expression between *RO60* and the pleiotropic or non-pleiotropic *RNY* signatures in TCGA normal tissue. The PCCs and *p* values are indicated. **b** Graphs showing the number of variants (SNPs) identified as pleiotropic in *RNYs* (± 50 kb) and correlated (European *r*^*2*^ > 0.4, left panel; *r*^*2*^ > 0.8, right panel) with SLE GWAS catalog variants, and compared with the results of equivalent 1000 random variant sets (European MAF > 0.01). **c** Box plot showing overexpression of the pleiotropic *RNY* signature in plasma of women who developed sporadic breast cancer (< 12 months after blood test) relative to matched controls who did not develop any neoplasm. The significance (*p*) of the Wilcoxon rank test is shown. **d** Box plot showing overexpression of the pleiotropic *RNY* signature in plasma of women carriers of pathological variants of *BRCA1* and *BRCA2* who developed breast cancer (< 12 months after blood test) relative to matched controls who did not develop any neoplasm. The significance (*p*) of the Wilcoxon rank test is shown

Ro60 controls the quality of noncoding RNAs [102, 103] and Ro60 loss causes anomalous activation of inflammatory pathways [104–106]. Ro60 binding to *RNY1* and *RNY3* is necessary to sustain a normal Ro60 level in cells, and these functional *RNYs* also influence Ro60’s subcellular location and interactions [92]. In turn, Ro60 loss is correlated with reduced levels of functional *RNY* expression [104]. Similarly, we found that the expression profiles of the pleiotropic and non-pleiotropic *RNY* signatures were positively correlated with *RO60* expression in TCGA normal tissue: PCC = 0.17 and 0.27; *p* = 3 × 10^−5^ and 2 × 10^−11^, respectively (Fig. 7a).

Ro60 was originally identified as a soluble antigen targeted by autoantibodies from patients with autoimmune rheumatic diseases; systemic lupus erythematosus (SLE) and Sjögren’s syndrome [107, 108]. SLE patients have increased risk of several cancer types [109]. Next, we analyzed the GWAS catalog of SLE risk variants (*n* = 917) in search of a link to pleiotropic variants in *RNY* loci. Seventeen and eight pleiotropic variants in *RNY* TSSs ± 50 kb were found to be linked to SLE risk variants when using two thresholds (European *r*^*2*^ > 0.4 and > 0.8, respectively), and these figures of correlated genetic elements were found to be greater than expected from 1000 sets of 917 randomly selected variants (European MAF > 0.01; Fig. 7b and Additional file 1: Table S25). None of the pleiotropic variants was found to be linked to variants of risk for Sjögren’s syndrome (*n* = 48).

### Overabundance of plasma RNY transcripts preceding breast cancer diagnosis

Since the overexpression of *RNYs* might be associated with an increased risk of cancer, we analyzed the levels of *RNY* transcripts in plasma collected from women before they developed breast cancer and compared the results with those of matched women who remained unaffected. Using small RNA-sequencing (sRNA-seq), two independent breast cancer sets were analyzed: a set of women carriers of pathogenic variants in *BRCA1* and *BRCA2*, and diagnosed with breast cancer as a first neoplasm within 12 months of their blood test (*n* = 11), or who provided a blood sample at a similar age and remained unaffected (*n* = 13; Additional file 1: Table S2); and a set from a long-term prospective study [110], comprising eight sporadic breast cancer cases (diagnosed within 12 months of the blood test) and eight controls matched for individual and epidemiological variables (Additional file 1: Table S3).

Unsupervised hierarchical clustering of individual *RNY* expression profiles did not distinguish women by their cancer-affected or cancer-unaffected status (Additional file 2: Fig. S7). However, computing the signature score of the pleiotropic *RNYs* showed significant overexpression in the plasma of the sporadic cases relative to unaffected women (Wilcoxon rank test *p* = 0.032; Fig. 7c). A similar, though not significant, difference was observed when comparing affected and unaffected women carriers of pathogenic variants in *BRCA1* and *BRCA2* (Fig. 7d). Consistent with the high correlation of levels of expression between *RNY* signatures (Fig. 6c,h), analysis of the non-pleiotropic *RNY*s showed similar differences in both sets (Additional file 2: Fig. S8). By contrast, the expression of four miRNAs known to be abundant in extracellular vesicles and/or lipoprotein particles of plasma (miR-16-5p, miR-21-5p, and miR-122-5p, miR-150-5p) was not significantly different in either set (Additional file 2: Fig. S9). These data suggest that overexpression of *RNY* sequences is associated with an increased risk of breast cancer.

## Discussion

This study identifies 4093 pleiotropic variants influencing blood traits and cancer risk in populations of European origin. A substantial proportion of blood-cancer pleiotropism is connected to immune-related molecules and regulators of telomere length in immune and/or epithelial cells. Expanding on these observations, the predicted pleiotropic genes converge on regulatory features, gene expression profiles, and master regulators of hematopoiesis, in which factors that control myeloid lineage appear to be of greater relevance. The data provide evidence that disrupted immune surveillance increases the risk of cancer [111–113]. However, additional studies, including Mendelian randomization [114] to assess causality of the identified genetic factors, and functional assays of defined gene candidates, are required to determine the mechanisms of pleiotropism accurately.

Myeloid lineage may be of major relevance to blood trait–cancer pleiotropism, as indicated by the identification of key master regulators, their transcriptional programs and associated progenitor cell states. A recent study showed that breast tumor cells can distantly remodel the cellular cross-talks in the bone marrow niche to increase myelopoiesis [115]. Our study identifies the pleiotropic candidate SPI1/PU.1, which is necessary for normal myeloid and lymphoid development [116, 117], as controlling progenitor fate, but it is specifically required for the maturation of myeloid progenitors [118]. The pleiotropic variant rs71475909 was found to be associated with breast cancer risk and eosinophil counts, and this variant is in LD with a splicing QTL of *SPI1* in blood cells [119]. In addition, SPI1 and another proposed pleiotropic factor, ZFPM1/FOG1 (which is linked to *BRCA1*-associated breast cancer and eosinophil counts, among other blood traits), are involved in the lineage commitment of eosinophils [120, 121]. It is of particular note that the systemic increase and tissue activation of eosinophils are associated with beneficial responses to immunotherapy in breast cancer [122], non-small cell lung cancer [123, 124], melanoma [125–127], and renal cell carcinoma [128]. In turn, high levels of circulating immunoglobulin E (IgE), and conditions of allergy and atopy may be protective of specific tumor types [129], whereas IgE immunodeficiency may increase cancer risk [130]. Thus, identified pleiotropic factors may influence cancer risk by determining myeloid lineage and the ultimate differentiation of cells, including that of eosinophils. The inferred protective effect of eosinophil counts for common cancer types in the UKBB supports this hypothesis.

Alteration of hematopoiesis and myeloid differentiation influencing blood trait–cancer pleiotropism might in turn be associated with the phenomenon of “clonal hematopoiesis”: i.e., clonal expansion of hematopoietic stem cells and their progeny due to acquired somatic mutations in driver genes, frequently linked to myeloid malignancies [131, 132]. This phenomenon causes immune dysregulation, inflammatory disease, and increased risk of hematological and solid cancers, among other consequences [133–135]. Pathological variants of genes functionally linked to the regulation of telomere length have been associated with sporadic and familial clonal hematopoiesis [88, 136]. Mendelian randomization analyses have indicated causality linking relative long telomere length to increased cancer risk [137, 138]. Further studies including clonal hematopoiesis as an additional trait are required to determine the interplay between perturbed hematopoiesis and cancer risk.

The overexpression of functional *RNYs* and of their processed fragments may induce inflammatory responses directly and/or indirectly from their interaction with Ro60 [105, 106, 139]. The plasma ratios of *RNY* subtypes are altered upon systemic inflammation [140], and *RNY*-derived sequences can activate macrophages [139]. The identification of an excess of pleiotropic signals in *RNY*-containing loci might indicate that deregulated expression of these sequences influences cancer risk by altering the levels of immune cell types and/or inflammatory signals. According to the hypothesis, the pleiotropic variants identify *RNY* transcripts that tend to be overexpressed in normal and cancer tissue, and in plasma samples of cancer patients. Analysis of plasma *RNYs* in women prior to breast cancer development supports the link between *RNY* overexpression and increased risk, although our sample sets were of limited size. Larger studies across a range of cancer settings are needed to confirm the cancer-predictive capacity of *RNY* in body fluids. Future studies and attempts to assess applicability would also benefit from developing an informative *RNY* panel in which the corresponding transcripts are analyzed by a cost-effective method [141].

## Conclusions

The study draws further attention to the relevance of the influence of systemic immune cell alterations on cancer development. The analysis reveals extensive blood–cancer pleiotropy and predicts that alteration of hematopoietic development and immune cell function principally underlies this connection. Myeloid lineage bias may be particularly relevant for blood-cancer pleiotropism. In addition, the study shows that overexpression of Y-RNAs potentially contributes to pleiotropism and might predict cancer initiation, but that larger retrospective and prospective studies across the full spectrum of settings are warranted to assess these indications. The biological factors identified here suggest opportunities for better estimating cancer risk and for developing targeted prevention approaches.

### Supplementary Information

**Additional file 1: Table S1.** Blood traits, cancer types and GWAS data sources. **Table S2.** Plasma samples of women carriers of pathogenic variants in *BRCA1/2*, affected or unaffected by breast cancer after blood test (< 12 months) and used for circulating sRNA-seq. **Table S3.** Plasma samples of sporadic women affected or unaffected by breast cancer after blood test (< 12 months) and used for sRNA-seq. **Table S4.** Multivariate Cox regression analysis of cancer diagnosis in UKBB (all cancers; >12 months from basal blood test). **Table S5.** Multivariate Cox regression analysis of cancer diagnosis in UKBB (all cancers; within 12 months from basal blood test). **Table S6.** Multivariate Cox regression analysis of cancer diagnosis in women of the UKBB (all cancers; >12 months from basal blood test). **Table S7.** Multivariate Cox regression analysis of cancer diagnosis in men of the UKBB (all cancers; >12 months from basal blood test). **Table S8.** Patient and incident cases included in the analyses. **Table S9.** Multivariate Cox regression analysis of breast cancer diagnosis in UKBB (>12 months from basal blood test). **Table S10.** Multivariate Cox regression analysis of colon cancer diagnosis in UKBB (>12 months from basal blood test). **Table S11.** Multivariate Cox regression analysis of lung cancer diagnosis in UKBB (>12 months from basal blood test). **Table S12.** Multivariate Cox regression analysis of prostate cancer diagnosis in UKBB (>12 months from basal blood test). **Table S13.** Heritability and genetic correlations between blood cell traits and cancer risk. **Table S14.** Genomic inflation (lambda factor) analysis for the comparisons between cancer risk and blood trait GWAS results. **Table S15.** Pleiotropy leading SNPs linking blood traits and cancer risk. **Table S16.** Pan-cancer pleiotropic SNPs (Rashkin et al., 2020) identified in the blood-cancer pleiotropy study (conjFDR < 0.05). **Table S17.** Pleiotropic gene candidates previously associated with leukocyte telomere length (Codd et al., 2021). **Table S18.** Genomic hotspots (1, 3, or 5 Mb) with significant enrichment in pleiotropic variants and linked to > 2 cancer traits. **Table S19.** Regulatory marks enriched in the blood-cancer pleiotropic variants (DNAse I hypersensitivity (sheffield_dnase), transcription factor binding sites (encode_tfbs), and epigenetic marks (oadmap_epigenomics) data). **Table S20.** Master regulators of hematopoiesis. **Table S21.** Pleiotropic gene candidates identified in the hematopoiesis-related gene modules (Velten et al., 2017). **Table S22.** Pleiotropic variants linked to *RNY*-containing loci. **Table S23.** GWAS-catalog cancer risk associations linked to *RNY*-containing loci (chromosomes 1-22). **Table S24.** Regulatory marks enriched in the 5' and 3' TSS regions of the pleiotropic *RNY* relative to non-pleiotropic *RNY* loci. **Table S25.** SLE risk variants (GWAS) correlated with blood-cancer pleiotropic variants in *RNY*-containing loci.

**Additional file 2: Fig. S1.** Blood trait associations with cancer diagnosis in the first year. **Fig. S2.** Genetic correlations among blood traits and cancer risk. **Fig. S3.** Q-Q plots for the genetic comparisons between blood traits and cancer risk. **Fig. S4.** Pleiotropic variant in a *RNY*-transcribed sequence. **Fig. S5.** *RNY* signatures and age of diagnosis of cancer types in TCGA. **Fig. S6.** Phylogenetic analysis of *RNY* sequences from the human genome. **Fig. S7.** The individual profiles of *RNYs* in plasma do not predict breast cancer. **Fig. S8.** General *RNY* overabundance in plasma is associated with breast cancer development. **Fig. S9.** Absence of association between levels of miRNAs known to be abundant in human plasma and breast cancer development.

**Additional file 3.**

## Data Availability

The sRNA-seq data generated in this study have been deposited in the Gene Expression Omnibus (GEO) database [142] under accession number GSE239907 (https://www.ncbi.nlm.nih.gov/geo/query/acc.cgi?acc=GSE239907) [143]. The individual UKBB [144] protected data were obtained upon application request and approval: project 61744 (https://www.ukbiobank.ac.uk/enable-your-research/approved-research/study-of-white-blood-cell-counts-in-relation-to-cancer-risk) [145]. The sources of the summary statistics of the GWASs are denoted in Additional file 1: Table S1. Validation analyses were performed using publicly deposited data: GTEx Portal [47], Open Access Datasets (https://www.gtexportal.org/home/downloads/adult-gtex/bulk_tissue_expression) [146]; FANTOM5 Human Enhancers [51] (https://enhancer.binf.ku.dk/human_enhancers/presets) [147]; gene expression of immune cell states [86], BioStudies accession S-EPMC8642243 (https://www.ebi.ac.uk/biostudies/europepmc/studies/S-EPMC8642243) [148]; Mammalian Phenotype Browser [52], immune system phenotypes (https://www.informatics.jax.org/vocab/mp_ontology/MP:0005387) [149]; GWAS Catalog [94] (https://www.ebi.ac.uk/gwas/api/search/downloads/full) [150]; TCGA [95] data, Genomics Data Commons Portal (https://portal.gdc.cancer.gov/) [151]; RNA-seq data of blood immune cell populations [96], GEO [142] accession GSE60424 (https://www.ncbi.nlm.nih.gov/geo/query/acc.cgi?acc=GSE60424) [152]; and plasma extracellular RNA profiles [28], GEO [142] accession GSE71008 (https://www.ncbi.nlm.nih.gov/geo/query/acc.cgi?acc=GSE71008) [153]. All original code has been deposited at GitHub (https://github.com/pujana-lab/PleiotropyBloodCancer) [154] and is publicly available.

## Abbreviations

BC: Breast cancer
BCAC: Breast cancer association consortium
BMI: Body mass index
CI: Confidence interval
condFDR: Conditional false discovery rate
conjFDR: Conjunctional false discovery rate
CRP: C-reactive protein
eQTL: Expression quantitative trait locus
FDR: False discovery rate
GWAS: Genome-wide association study
HLA: Human leukocyte antigen
HR: Hazard ratio
ICD-10: International classification of diseases – 10^th^ edition
LD: Linkage disequilibrium
MAF: Minor allele frequency
MP: Mammalian phenotype
NHL: Non-Hodgkin’s lymphoma
OR: Odds ratio
PC: Platelet crit
PCC: Pearson’s correlation coefficient
PDW: Platelet distribution width
pleioFDR: Pleiotropy false discovery rate
SLE: Systemic lupus erythematosus
SNP: Single-nucleotide polymorphism
sRNA-seq: Small RNA-sequencing
ssGSEA: Single-sample gene set enrichment analysis
TCGA: The cancer genome atlas
TNBC: Triple-negative breast cancer
TPM: Transcripts per million
TSS: Transcription start site
UKBB: UK Biobank
