# Supplementary material for: Biological basis of extensive pleiotropy between blood traits and cancer risk
Source: Genome Med. 2024 Feb 2;16:21. doi: 10.1186/s13073-024-01294-8 (PMC10837955; doi:10.1186/s13073-024-01294-8)

**Biological basis of extensive pleiotropy between blood traits and cancer risk**

# Additional file 2 - Supplementary figures

**Fig. S1. Blood trait associations with cancer diagnosis in the first year.**

Forest plot showing the associations between blood traits and cancer diagnosis within the first year after basal blood test in the UK Biobank. The trait units, HR, 95% CI, and significance (p) of the multivariate Cox proportional model are indicated. The dataset was filtered, values of blood traits log_2_-transformed, and regression models stratified and adjusted as described in the Methods.


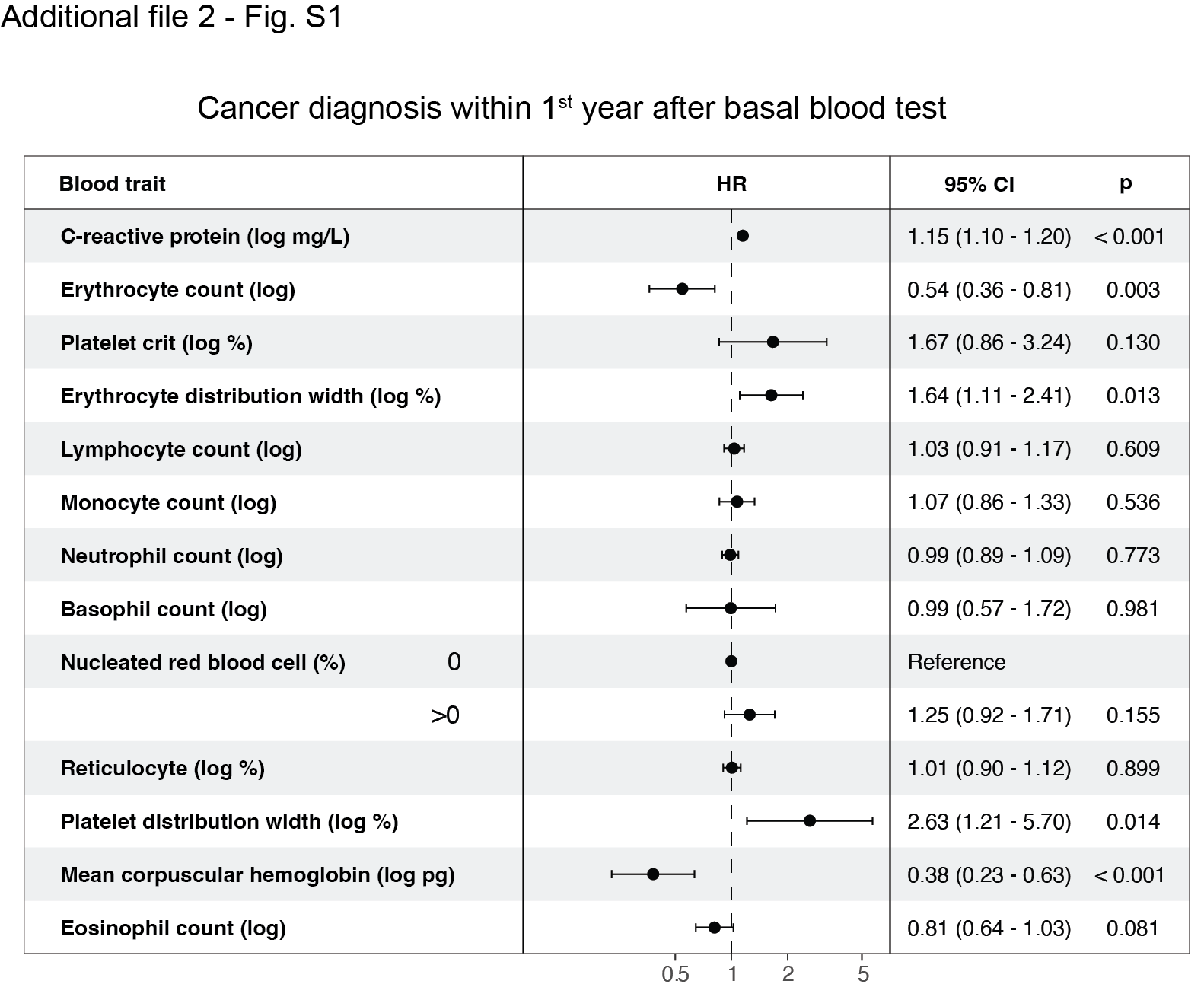


**Fig. S2. Genetic correlations among blood traits and cancer risk.**

**a** Heatmap of the genetic correlations among 27 blood traits. Asterisks indicate significant correlations (FDR-adjusted p < 0.05, as depicted in the inset).

**b** Heatmap of the genetic correlations among 28 cancer risk studies. Asterisks indicate significant correlations (FDR-adjusted p < 0.05, as depicted in the inset).

**c** Heatmap of the genetic correlations between the blood traits and cancer risk. Asterisks indicate nominally significant correlations, as depicted in the inset.


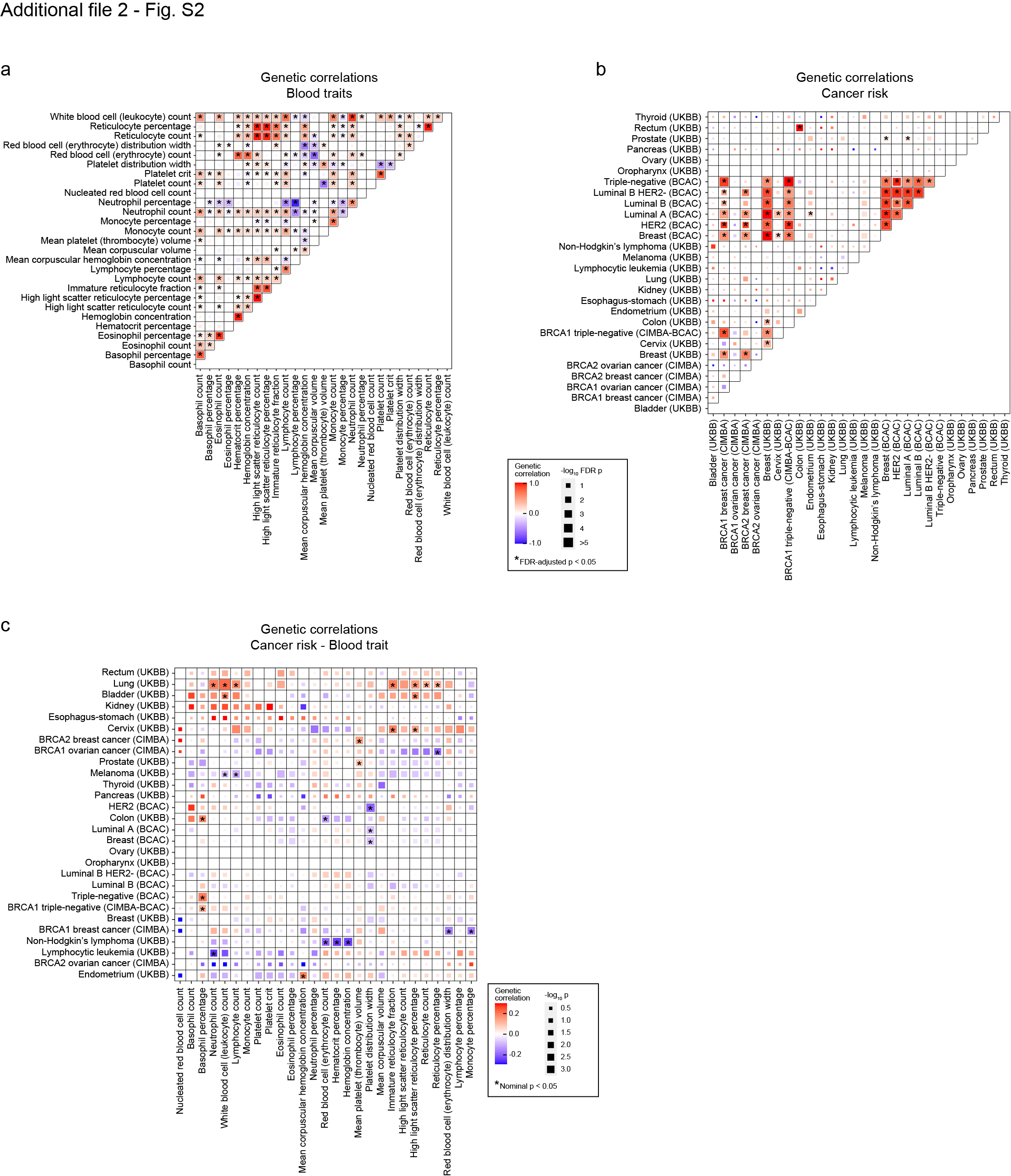


**Fig. S3 (see attached PDF file). Q-Q plots for the genetic comparisons between blood traits and cancer risk.** Conditional Q-Q plots of theoretical versus empirical -log_10_ p values, corrected for genomic inflation, and as a function of the statistical significance of association, as depicted in the inset. Dotted line indicates the theoretical line in the case of no association.

**Fig. S4. Pleiotropic variant in a *RNY*-transcribed sequence.** Sequence alignment between ENSG00000201160 and the *RNY1* sequence. Asterisks indicate concordant nucleotides. The rs10193900 allele difference is shown.


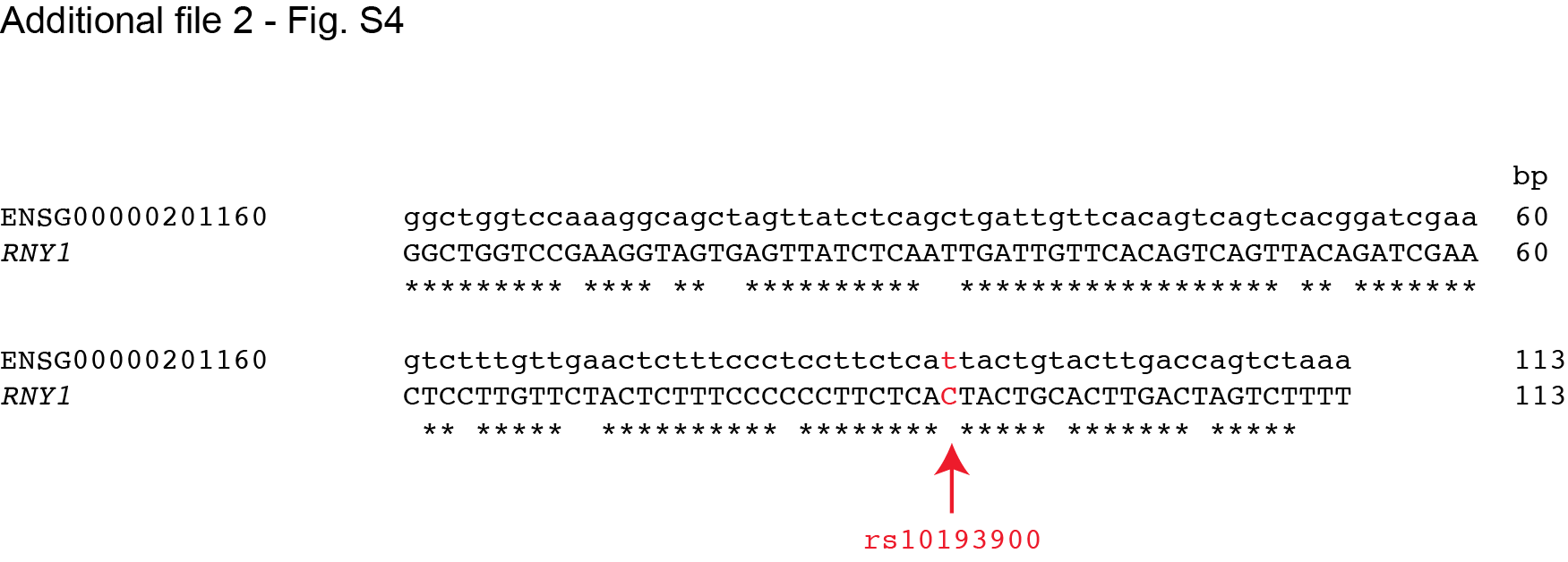


**Fig. S5. *RNY* signatures and age of diagnosis of cancer types in TCGA.** Scatter plots of the correlations (PCCs and p values are indicated) between the pleiotropic or non-pleiotropic *RNY* signatures and age at diagnosis of each TCGA cancer type with available normal tissue data (the cancer study acronyms and number of tissue samples in each setting are indicated).

**
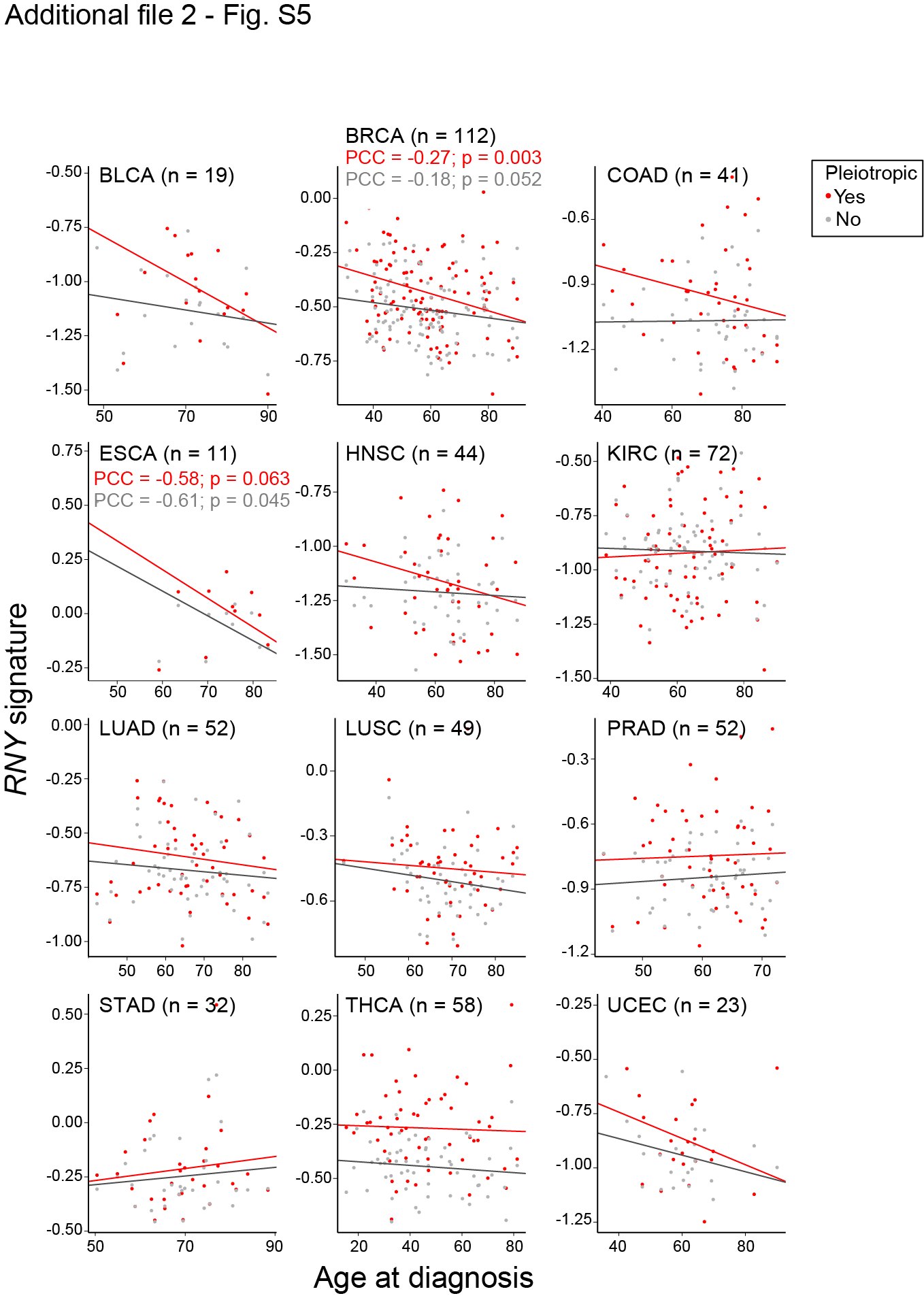
**

**Fig. S6. Phylogenetic analysis of *RNY* sequences from the human genome.** Phylogenetic tree generated from the sequence alignment of all *RNY* loci. The pleiotropy-based category and *RNY4* are indicated.


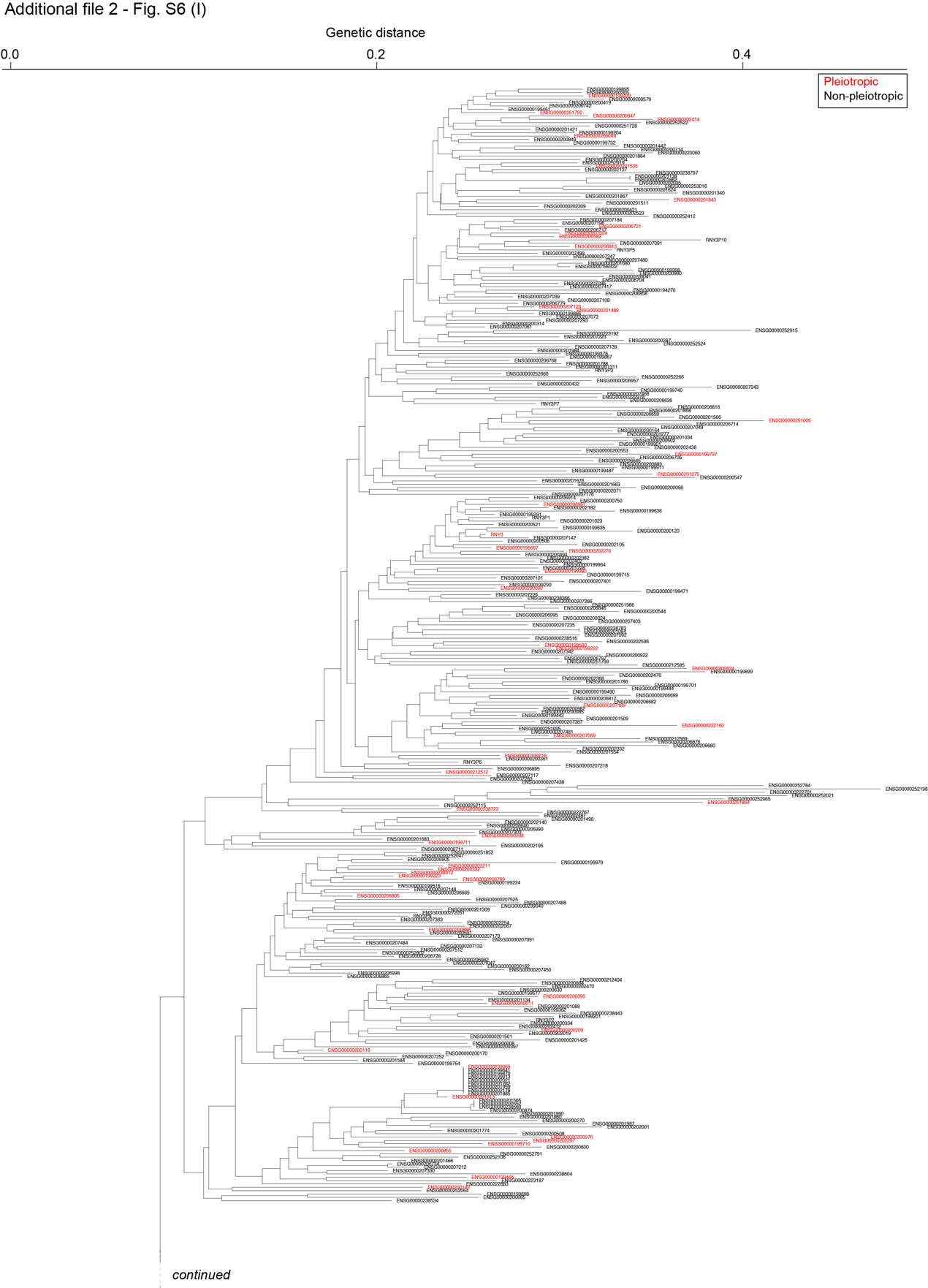


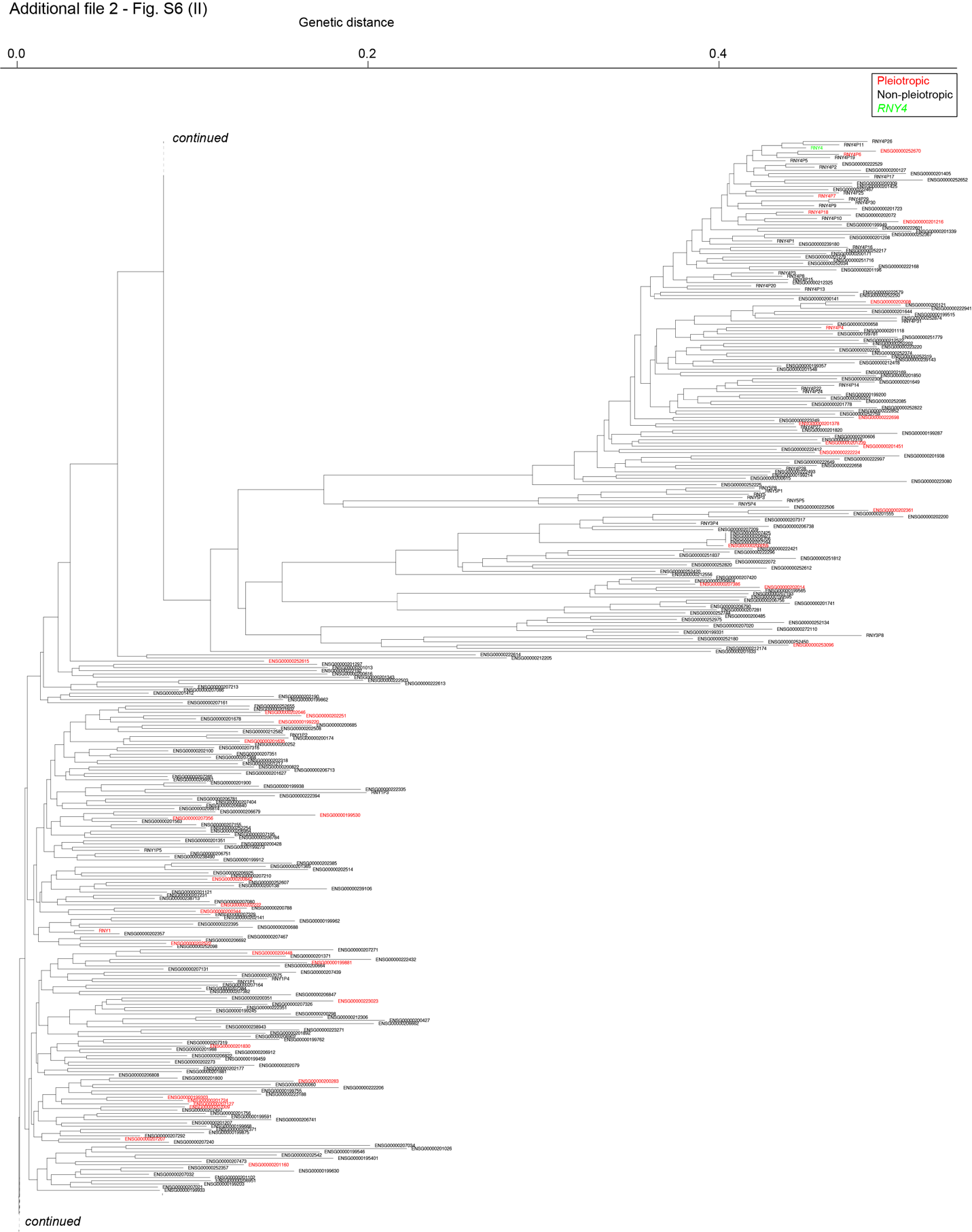


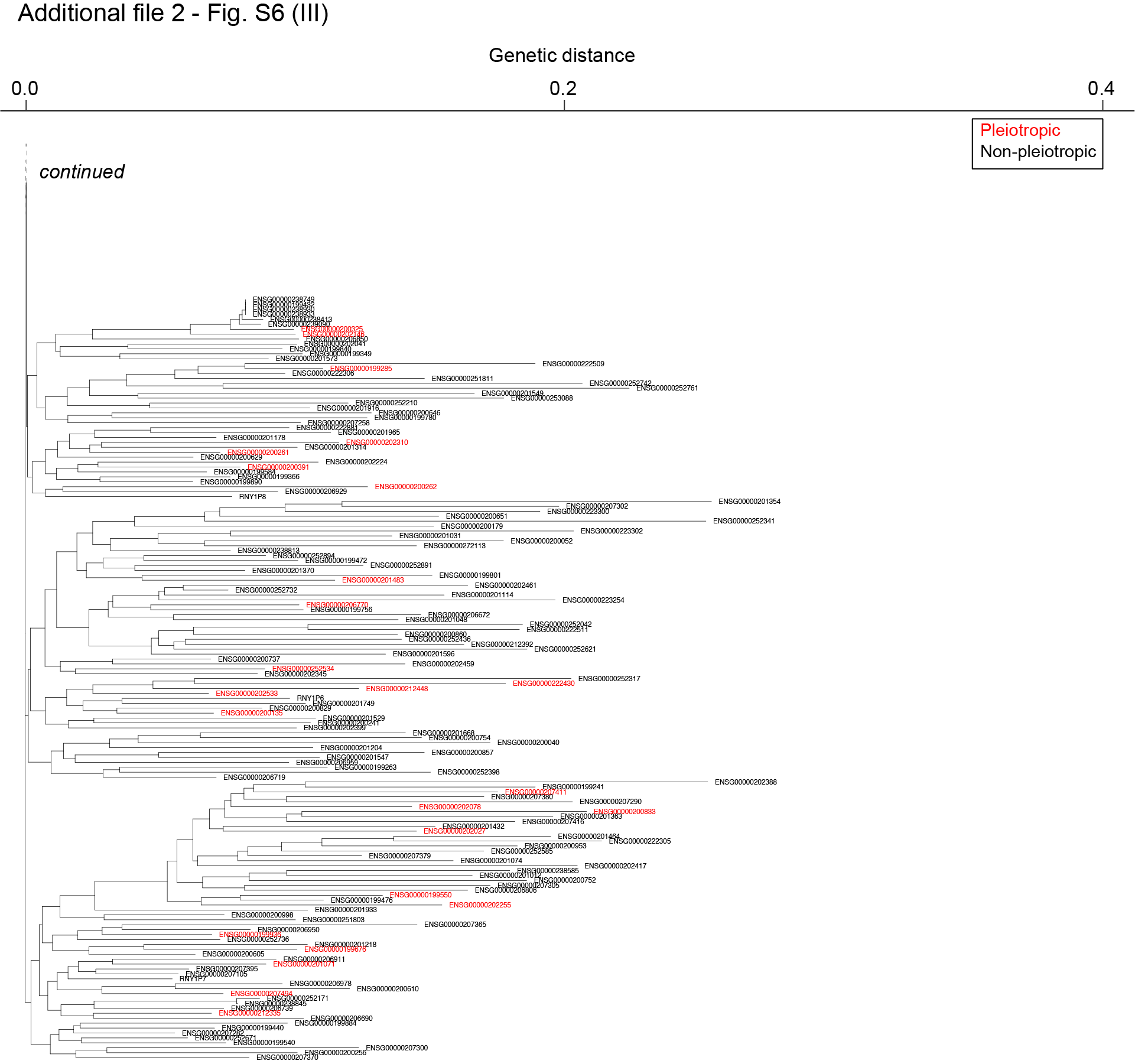


**Fig. S7. The individual profiles of *RNYs* in plasma do not predict breast cancer.** Heatmaps of unsupervised clustering of the pleiotropic and non-pleiotropic (indicated in the top bar) *RNY* profiles in the two sample sets analyzed (top, women carriers of *BRCA1* and *BRCA2* pathological variants; and bottom, sporadic-prospective study). The subsequent cancer status of each sample is indicated.


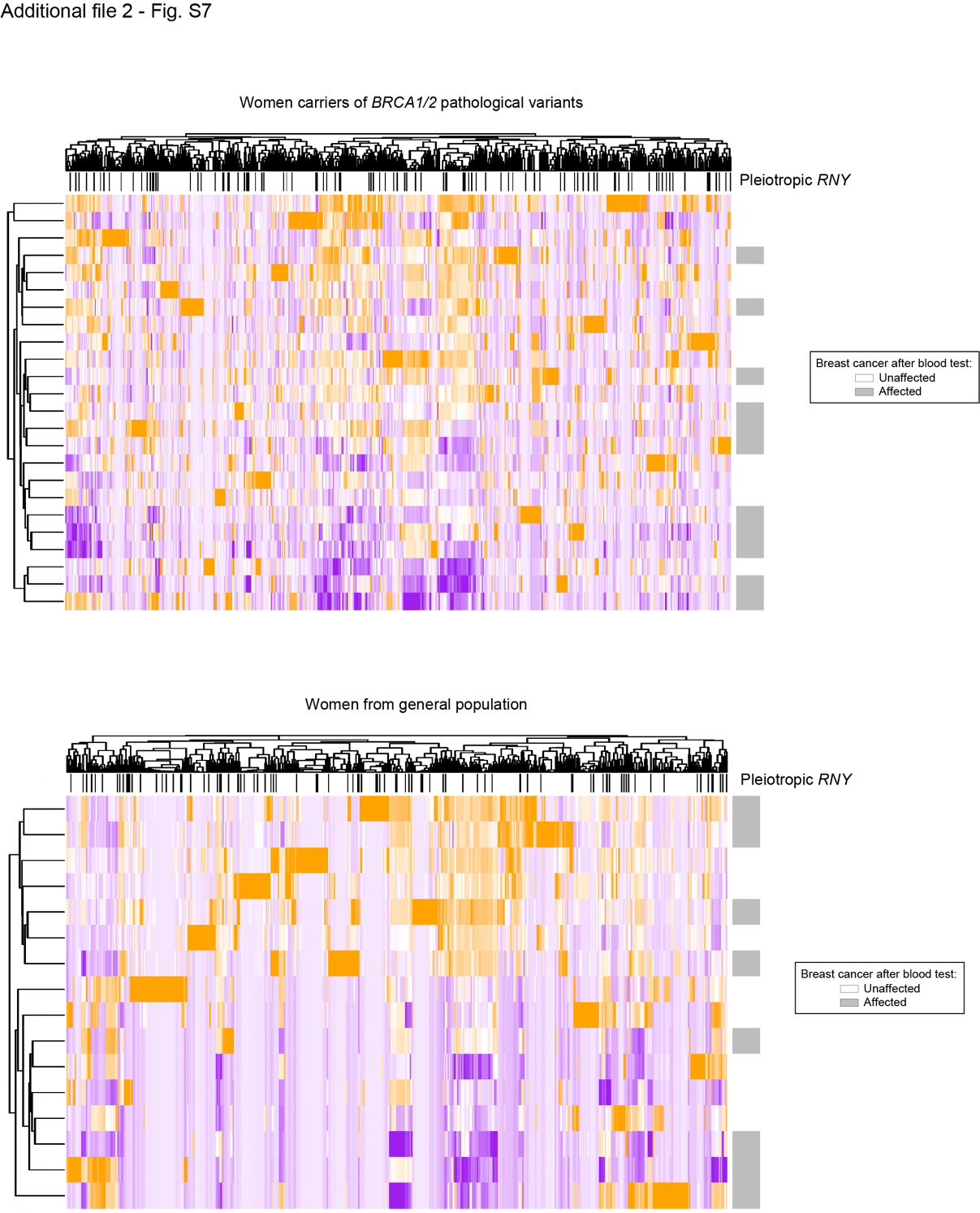


**Fig. S8. General *RNY* overabundance in plasma is associated with breast cancer development.** Box plots showing overexpression of the *RNY* signatures considering all *RNY*-derived transcripts from the human genome (left panels) or non-pleiotropic *RNYs* (right panels) in plasma of women from the prospective study (top panels) and carriers of pathological variants of *BRCA1* and *BRCA2* (bottom panels). The significance (p) of the Wilcoxon rank tests is shown.


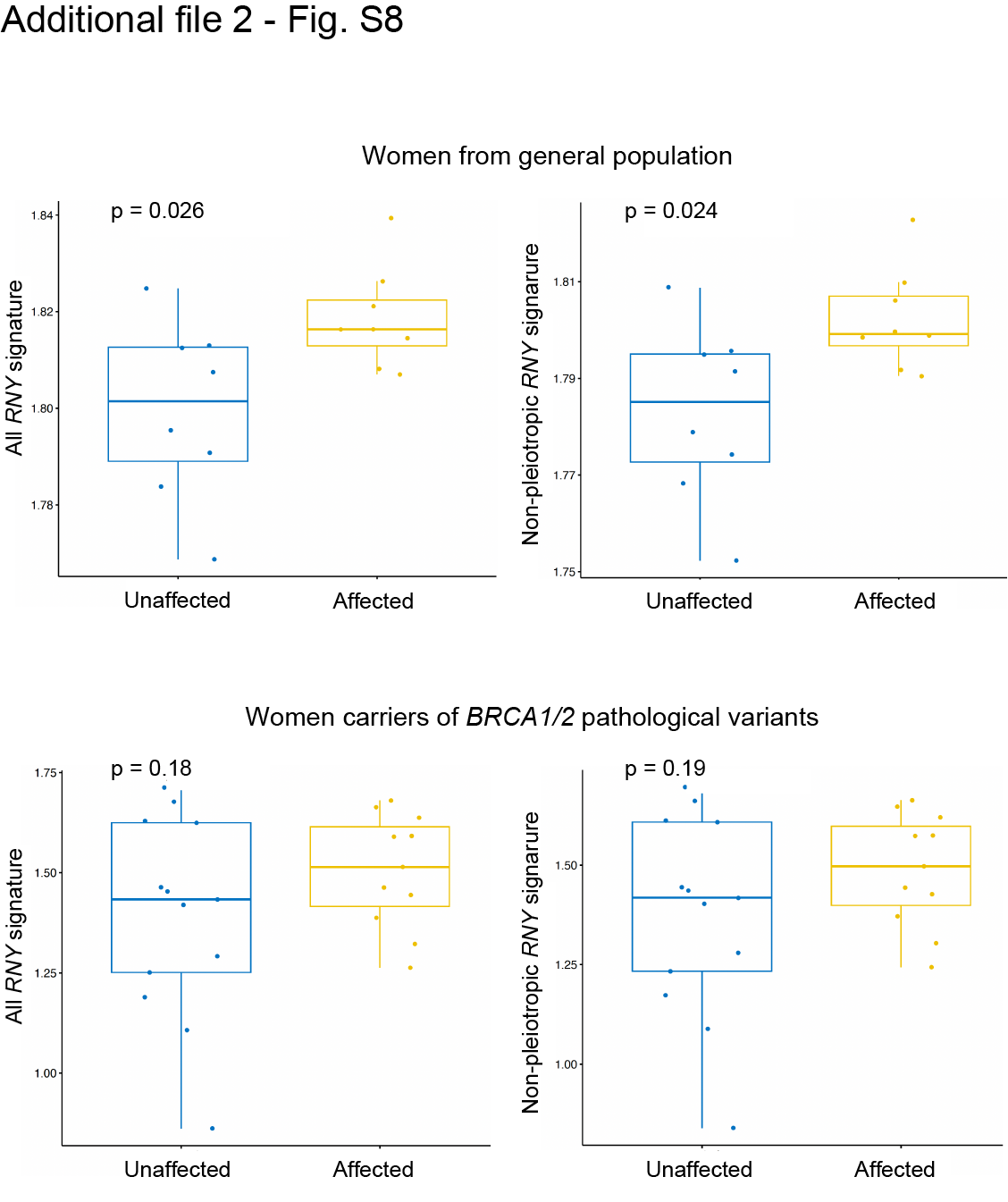


**Fig. S9. Absence of association between levels of miRNAs known to be abundant in human plasma and breast cancer development.** The results of four miRNAs in the two sample sets are shown. The significance (p) of the Wilcoxon rank tests is shown.


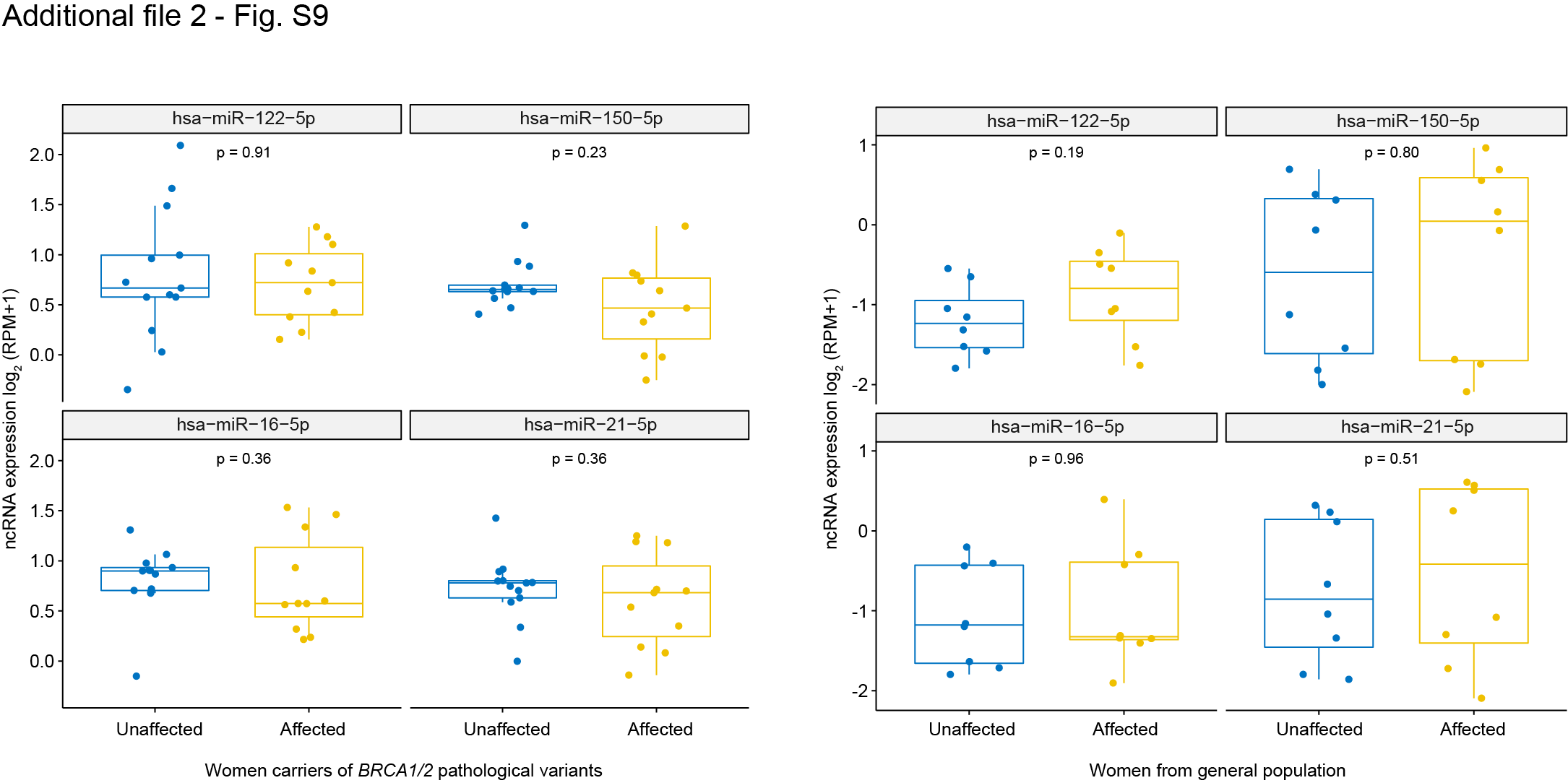

Supplement: Supplementary file 2 — Additional file 2: Fig. S1. Blood trait associations with cancer diagnosis in the first year. Fig. S2. Genetic correlations among blood traits and cancer risk. Fig. S3. Q-Q plots for the genetic comparisons between blood traits and cancer risk. Fig. S4. Pleiotropic variant in a RNY-transcribed sequence. Fig. S5. RNY signatures and age of diagnosis of cancer types in TCGA. Fig. S6. Phylogenetic analysis of RNY sequences from the human genome. Fig. S7. The individual profiles of RNYs in plasma do not predict breast cancer. Fig. S8. General RNY overabundance in plasma is associated with breast cancer development. Fig. S9. Absence of association between levels of miRNAs known to be abundant in human plasma and breast cancer development. [file 13073_2024_1294_MOESM2_ESM.docx]
