# Supplementary material for: Biological basis of extensive pleiotropy between blood traits and cancer risk
Source: Genome Med. 2024 Feb 2;16:21. doi: 10.1186/s13073-024-01294-8 (PMC10837955; doi:10.1186/s13073-024-01294-8)
Supplement: Supplementary file 3 — Additional file 3. [file 13073_2024_1294_MOESM3_ESM.pdf]

Additional file 2 - Fig. S3

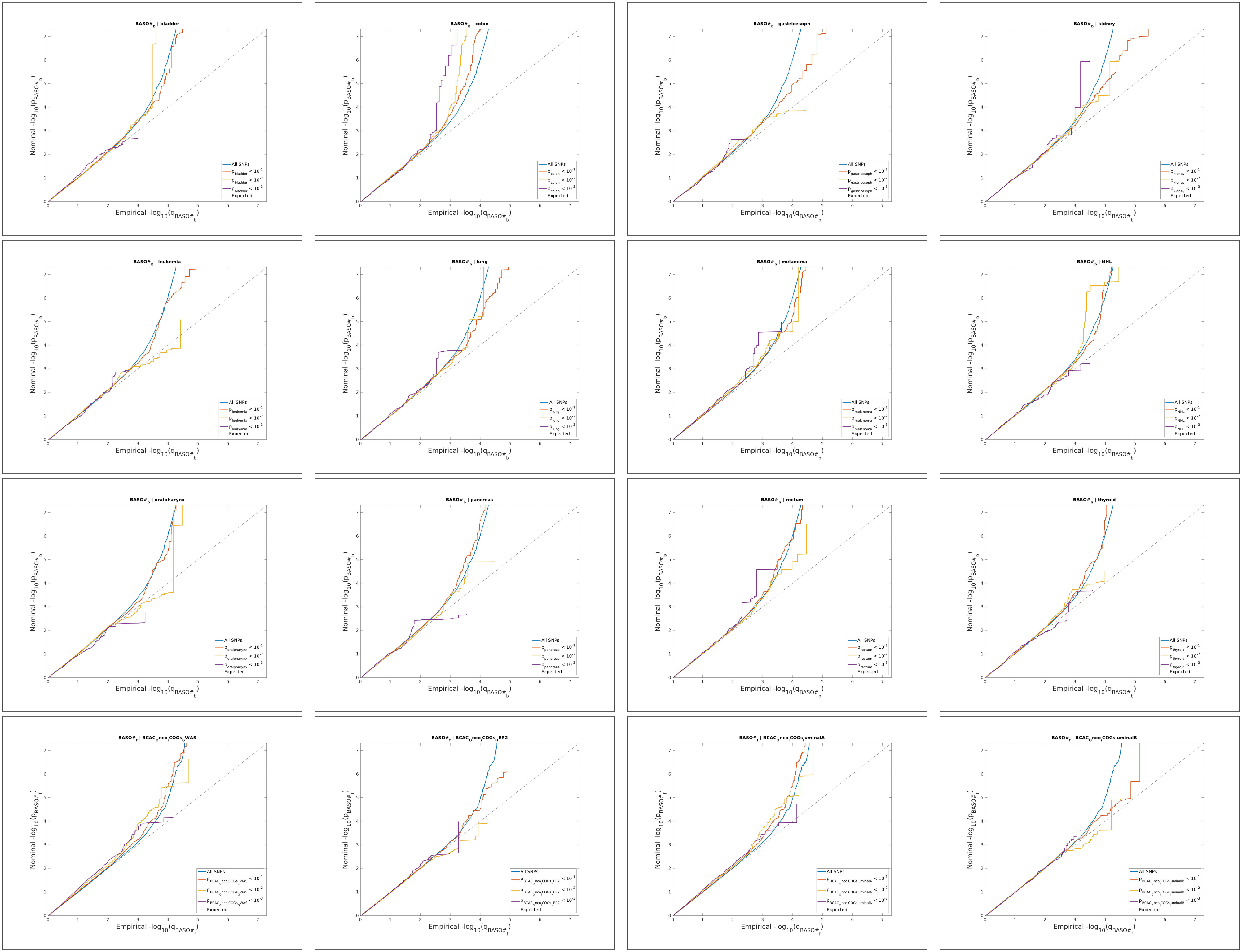

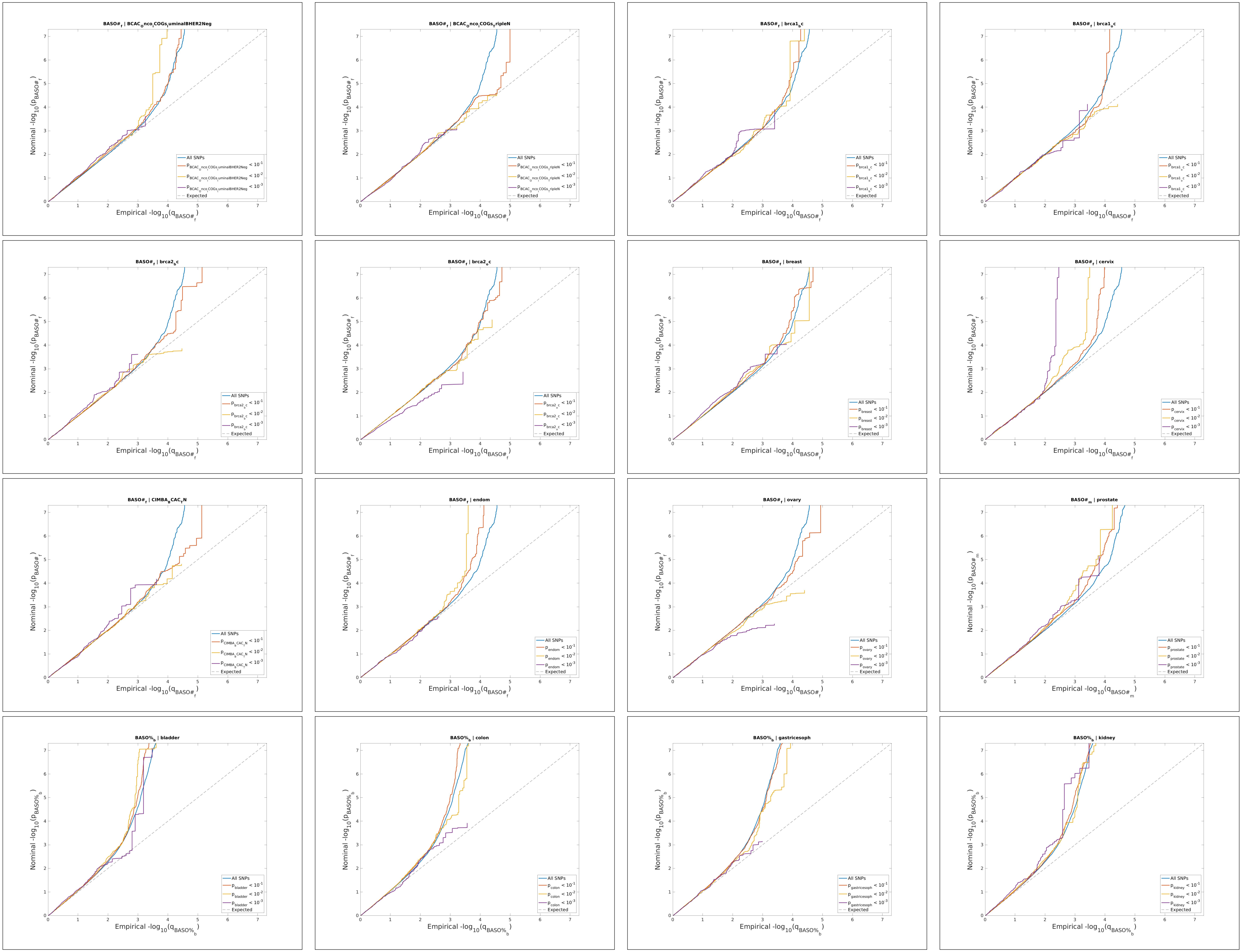

BASO#f | brca1bc
BASO#f | brca1oc
BASO#f | brca2bc
BASO#f | brca2oc
BASO#f | breast
BASO#f | cervix
BASO#f | endom
BASO#f | ovary
BASO#m | prostate
BASO%b | bladder
BASO%b | colon
BASO%b | gastricesoph
BASO%b | kidney
All SNPs
Expected
Nominal -log10(pBASO#f)
Empirical -log10(qBASO#f)

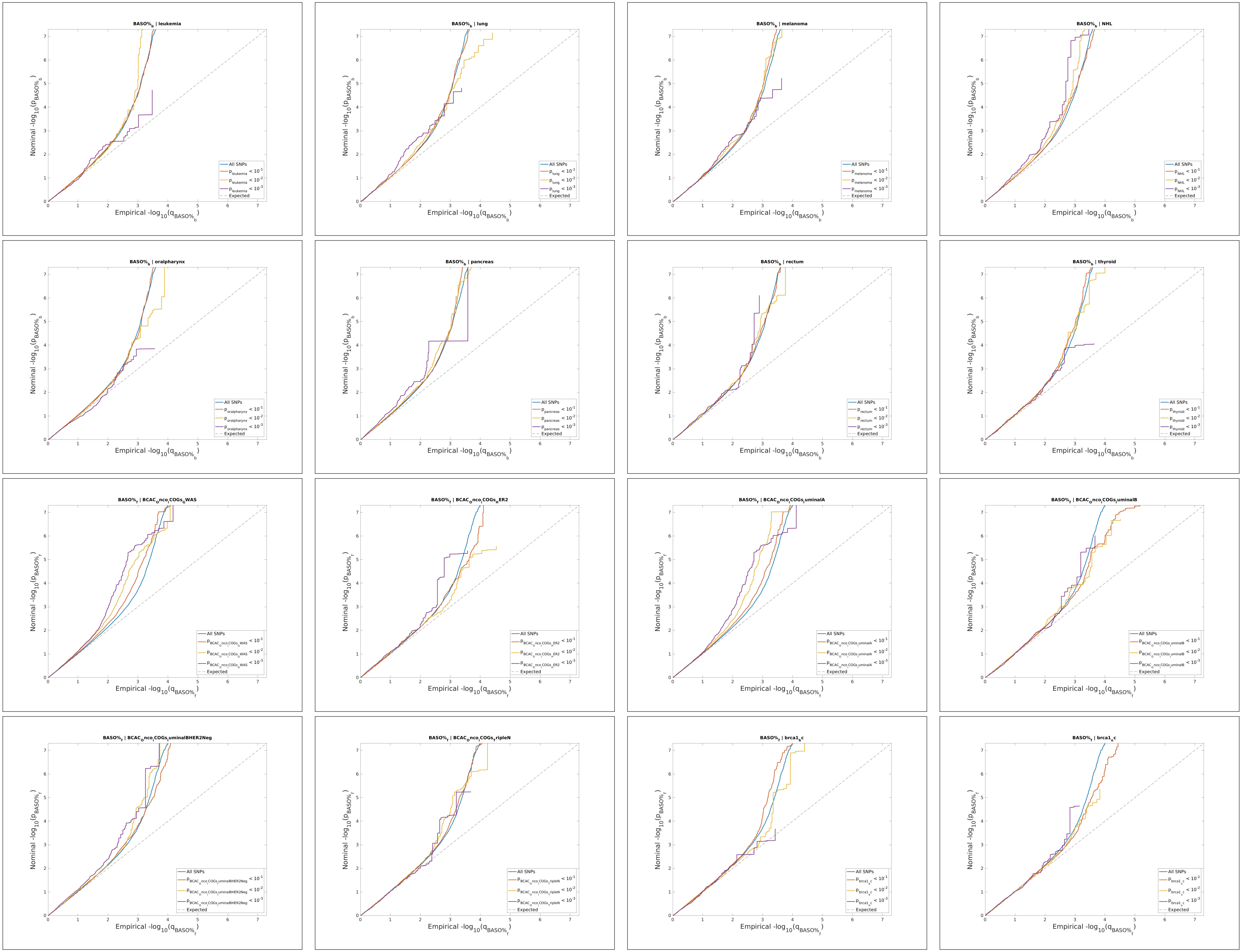
BASO%b | leukemia
BASO%b | lung
BASO%b | melanoma
BASO%b | NHL
BASO%b | oralpharynx
BASO%b | pancreas
BASO%b | rectum
BASO%b | thyroid
BASO%f | BCAConcoCOGsGWAS
BASO%f | BCAConcoCOGsHER2
BASO%f | BCAConcoCOGsLuminalA
BASO%f | BCAConcoCOGsLuminalB
BASO%f | BCAConcoCOGsLuminalBHER2Neg
BASO%f | BCAConcoCOGsTripleN
BASO%f | brca1bc
BASO%f | brca1oc

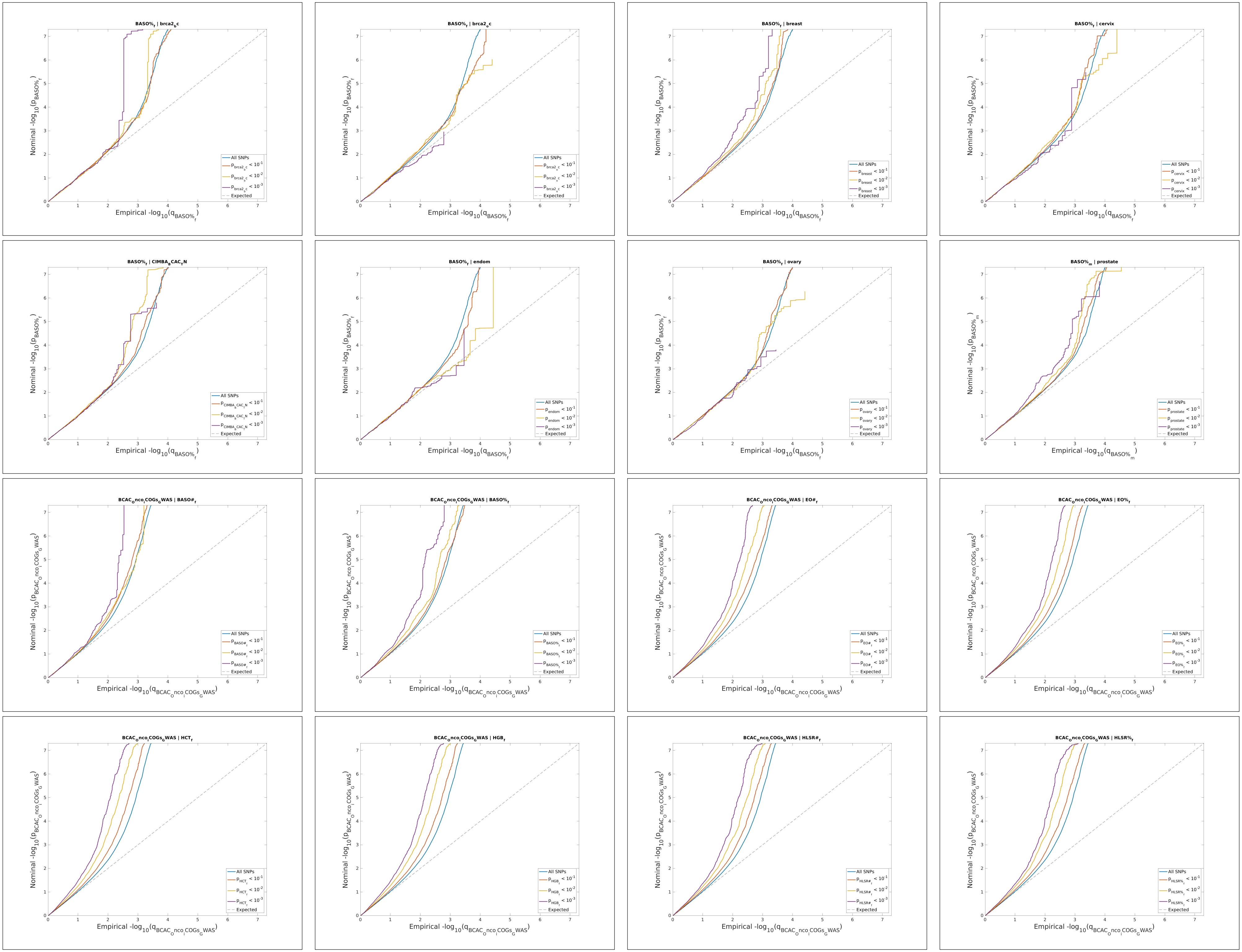

BASO%f | brca2bc
BASO%f | brca2oc
BASO%f | breast
BASO%f | cervix
BASO%f | CIMBABCACTN
BASO%f | endom
BASO%f | ovary
BASO%m | prostate
BCAConcoCOGsGWAS | BASO#f
BCAConcoCOGsGWAS | BASO%f
BCAConcoCOGsGWAS | EO#f
BCAConcoCOGsGWAS | EO%f
BCAConcoCOGsGWAS | HCTf
BCAConcoCOGsGWAS | HGBf
BCAConcoCOGsGWAS | HLSR#f
BCAConcoCOGsGWAS | HLSR%f
Nominal -log10(p)
Empirical -log10(q)
All SNPs
Expected

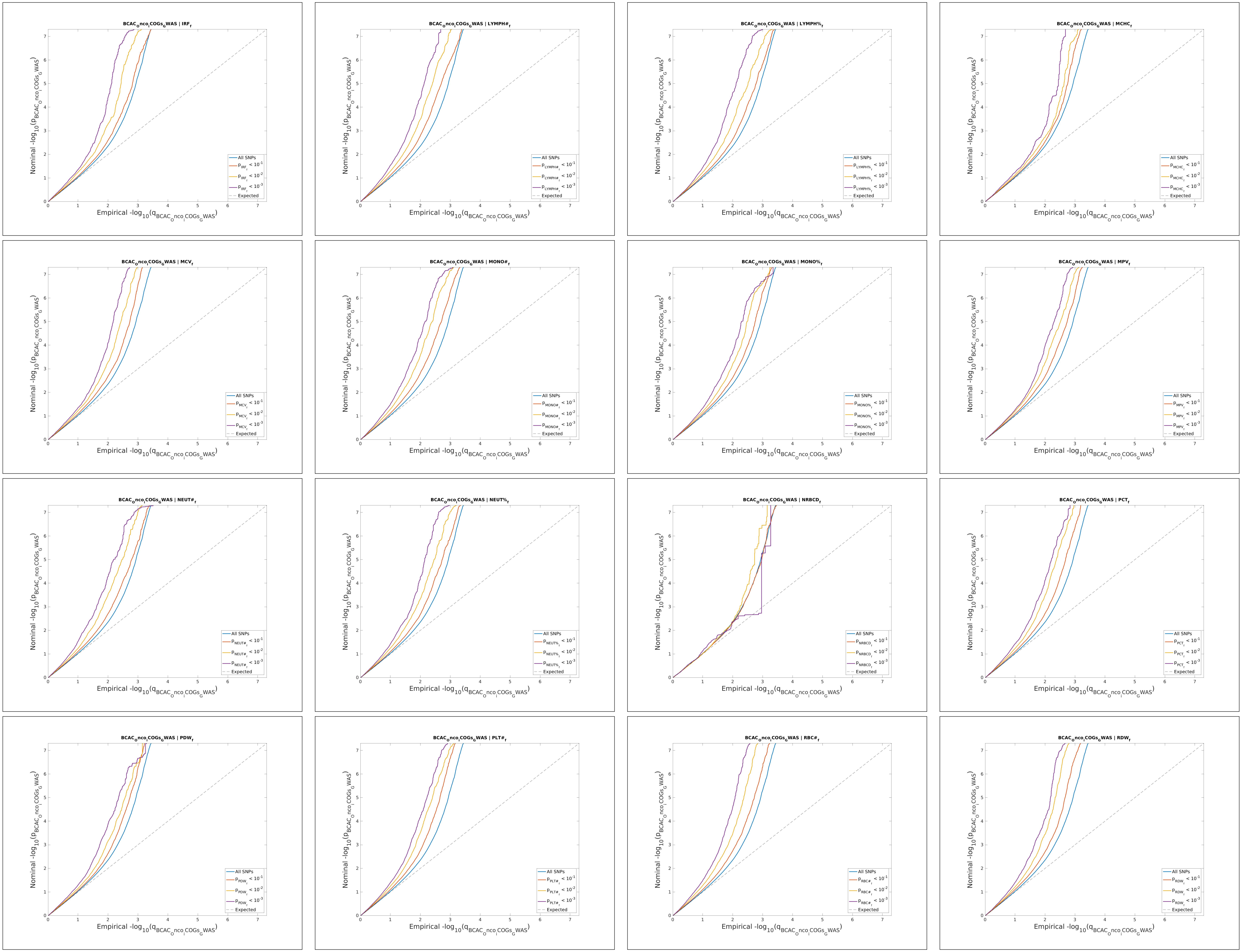
BCAC_Onco_iCOGs_GWAS | IRF_f
BCAC_Onco_iCOGs_GWAS | LYMPH#_f
BCAC_Onco_iCOGs_GWAS | LYMPH%_f
BCAC_Onco_iCOGs_GWAS | MCHC_f
BCAC_Onco_iCOGs_GWAS | MCV_f
BCAC_Onco_iCOGs_GWAS | MONO#_f
BCAC_Onco_iCOGs_GWAS | MONO%_f
BCAC_Onco_iCOGs_GWAS | MPV_f
BCAC_Onco_iCOGs_GWAS | NEUT#_f
BCAC_Onco_iCOGs_GWAS | NEUT%_f
BCAC_Onco_iCOGs_GWAS | NRBCD_f
BCAC_Onco_iCOGs_GWAS | PCT_f
BCAC_Onco_iCOGs_GWAS | PDW_f
BCAC_Onco_iCOGs_GWAS | PLT#_f
BCAC_Onco_iCOGs_GWAS | RBC#_f
BCAC_Onco_iCOGs_GWAS | RDW_f
Nominal -log10(p BCAC_Onco_iCOGs_GWAS)
Empirical -log10(q BCAC_Onco_iCOGs_GWAS)
All SNPs
< 10^-1
< 10^-2
< 10^-3
Expected

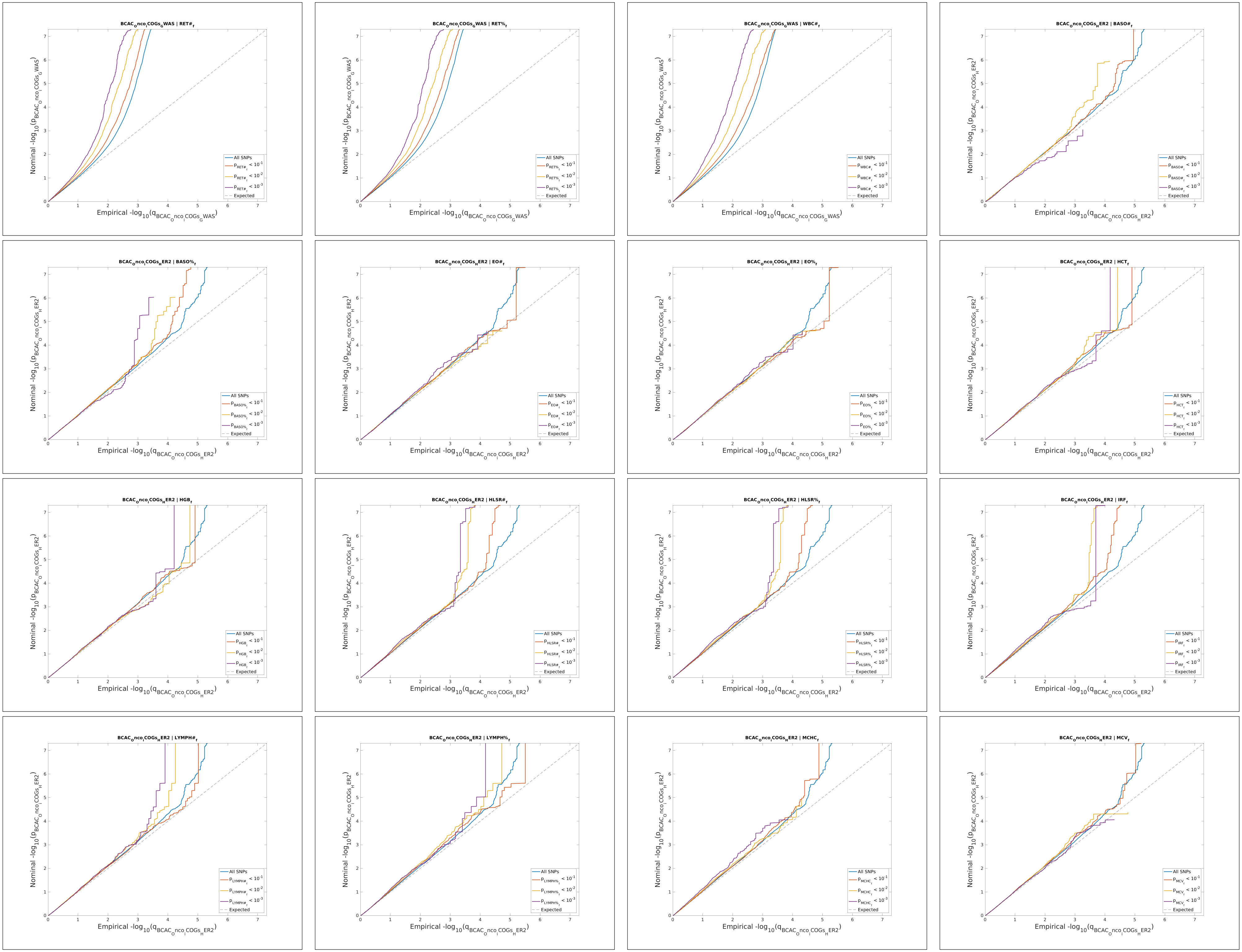

BCACOncoiCOGsGWAS | RET#f
BCACOncoiCOGsGWAS | RET%f
BCACOncoiCOGsGWAS | WBC#f
BCACOncoiCOGsHER2 | BASO#f
BCACOncoiCOGsHER2 | BASO%f
BCACOncoiCOGsHER2 | EO#f
BCACOncoiCOGsHER2 | EO%f
BCACOncoiCOGsHER2 | HCTf
BCACOncoiCOGsHER2 | HGBf
BCACOncoiCOGsHER2 | HLSR#f
BCACOncoiCOGsHER2 | HLSR%f
BCACOncoiCOGsHER2 | IRFf
BCACOncoiCOGsHER2 | LYMPH#f
BCACOncoiCOGsHER2 | LYMPH%f
BCACOncoiCOGsHER2 | MCHCf
BCACOncoiCOGsHER2 | MCVf
All SNPs
Expected

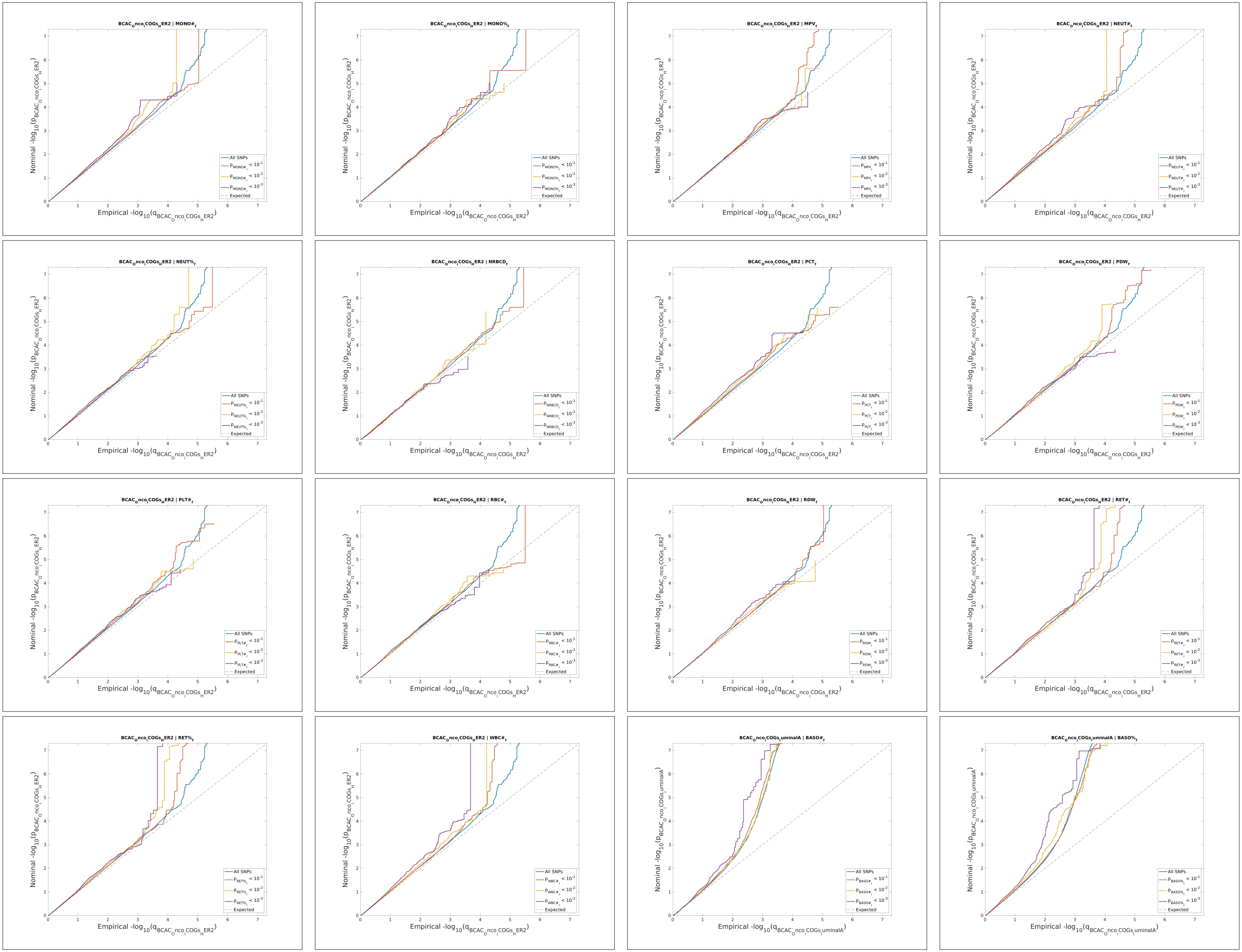

BCAC_OncoiCOGs_HER2 | MONO#_f
BCAC_OncoiCOGs_HER2 | MONO%_f
BCAC_OncoiCOGs_HER2 | MPV_f
BCAC_OncoiCOGs_HER2 | NEUT#_f
BCAC_OncoiCOGs_HER2 | NEUT%_f
BCAC_OncoiCOGs_HER2 | NRBCD_f
BCAC_OncoiCOGs_HER2 | PCT_f
BCAC_OncoiCOGs_HER2 | PDW_f
BCAC_OncoiCOGs_HER2 | PLT#_f
BCAC_OncoiCOGs_HER2 | RBC#_f
BCAC_OncoiCOGs_HER2 | RDW_f
BCAC_OncoiCOGs_HER2 | RET#_f
BCAC_OncoiCOGs_HER2 | RET%_f
BCAC_OncoiCOGs_HER2 | WBC#_f
BCAC_OncoiCOGs_LuminalA | BASO#_f
BCAC_OncoiCOGs_LuminalA | BASO%_f
All SNPs
Expected
Nominal -log10(p)
Empirical -log10(q)

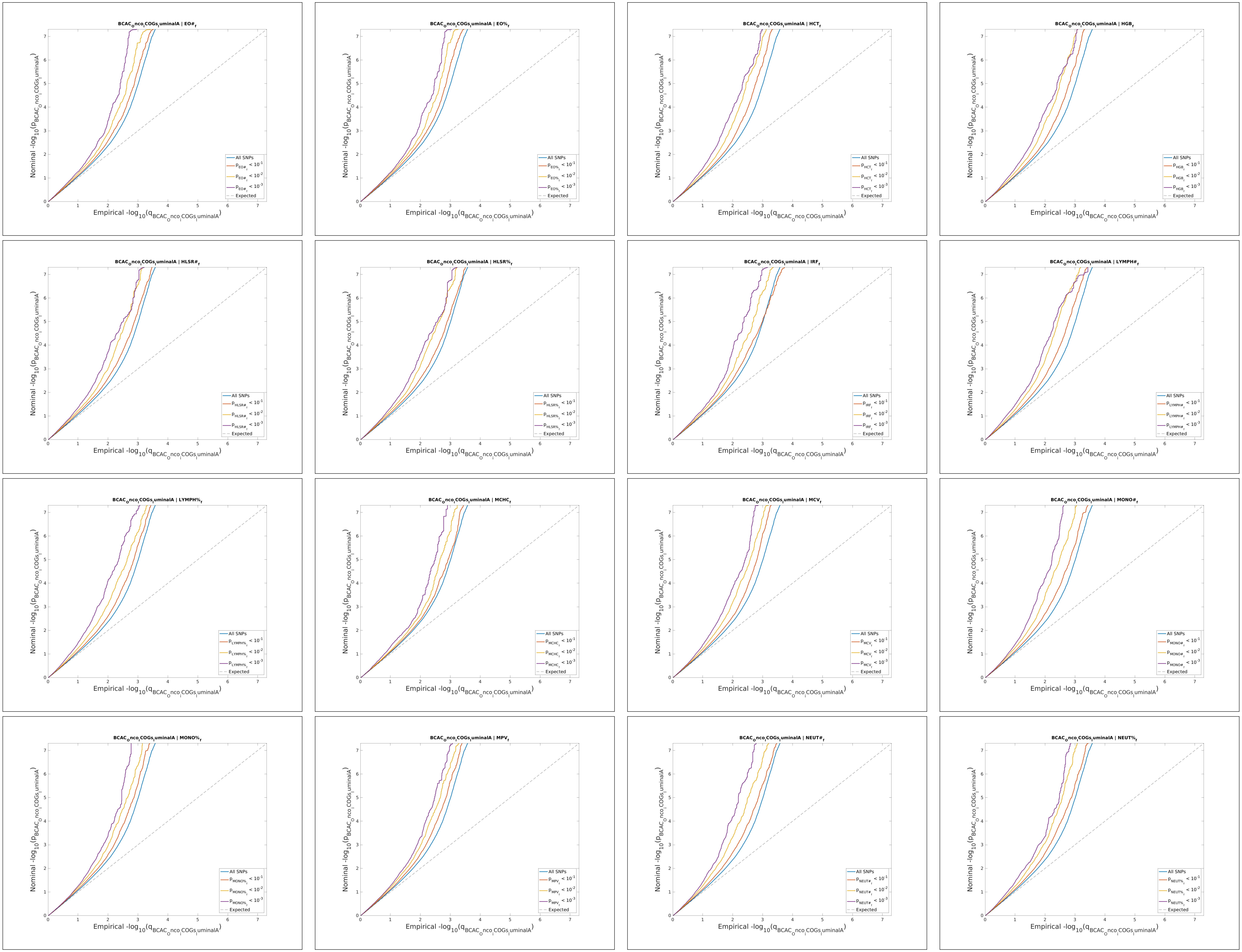
BCAConcoCOGsuminalA | EO#f
BCAConcoCOGsuminalA | EO%f
BCAConcoCOGsuminalA | HCTf
BCAConcoCOGsuminalA | HGBf
BCAConcoCOGsuminalA | HLSR#f
BCAConcoCOGsuminalA | HLSR%f
BCAConcoCOGsuminalA | IRFf
BCAConcoCOGsuminalA | LYMPH#f
BCAConcoCOGsuminalA | LYMPH%f
BCAConcoCOGsuminalA | MCHCf
BCAConcoCOGsuminalA | MCVf
BCAConcoCOGsuminalA | MONO#f
BCAConcoCOGsuminalA | MONO%f
BCAConcoCOGsuminalA | MPVf
BCAConcoCOGsuminalA | NEUT#f
BCAConcoCOGsuminalA | NEUT%f
All SNPs
Expected

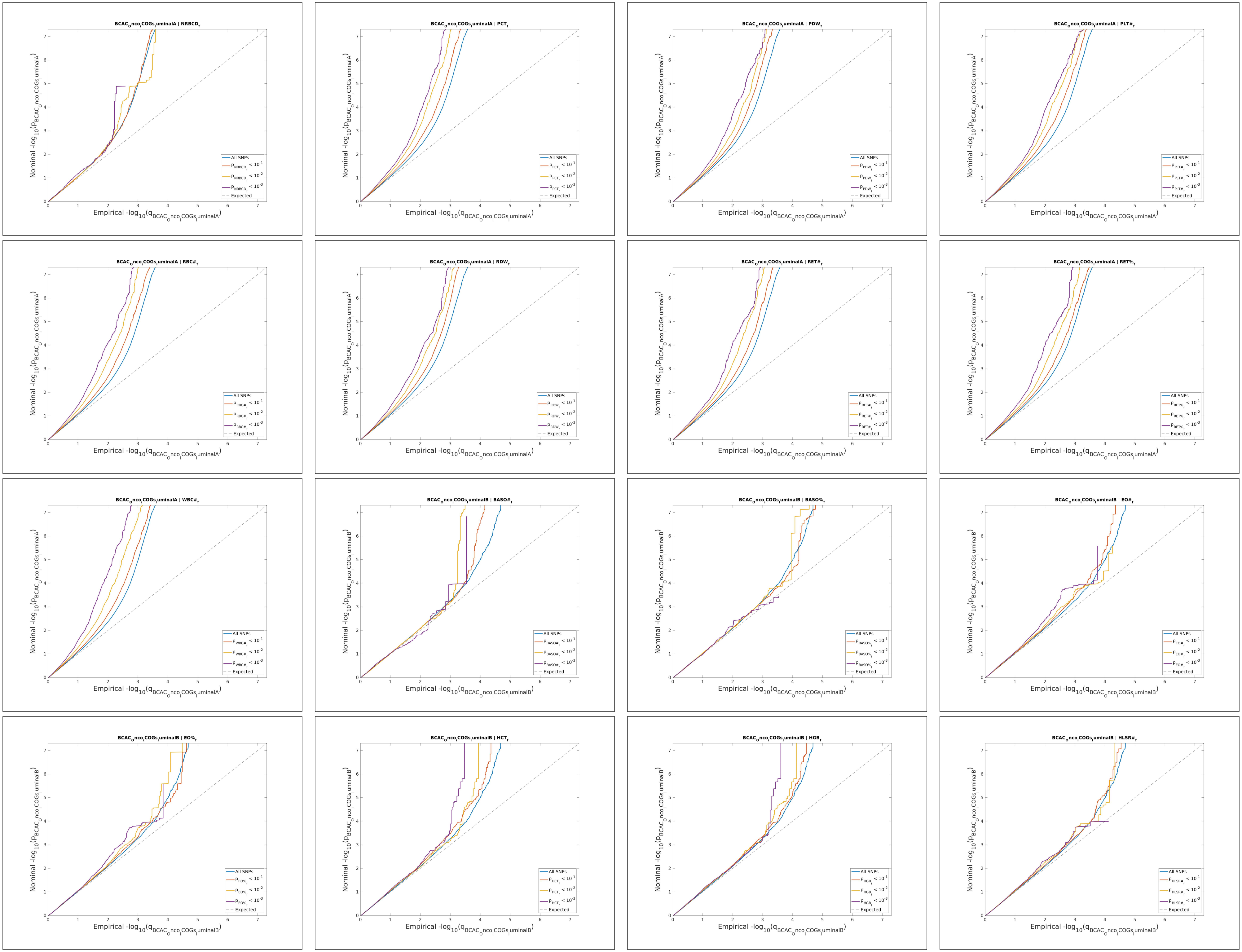

BCAConcoiCOGsluminalA | NRBCDf
BCAConcoiCOGsluminalA | PCTf
BCAConcoiCOGsluminalA | PDWf
BCAConcoiCOGsluminalA | PLT#f
BCAConcoiCOGsluminalA | RBC#f
BCAConcoiCOGsluminalA | RDWf
BCAConcoiCOGsluminalA | RET#f
BCAConcoiCOGsluminalA | RET%f
BCAConcoiCOGsluminalA | WBC#f
BCAConcoiCOGsluminalB | BASO#f
BCAConcoiCOGsluminalB | BASO%f
BCAConcoiCOGsluminalB | EO#f
BCAConcoiCOGsluminalB | EO%f
BCAConcoiCOGsluminalB | HCTf
BCAConcoiCOGsluminalB | HGBf
BCAConcoiCOGsluminalB | HLSR#f
All SNPs
Expected

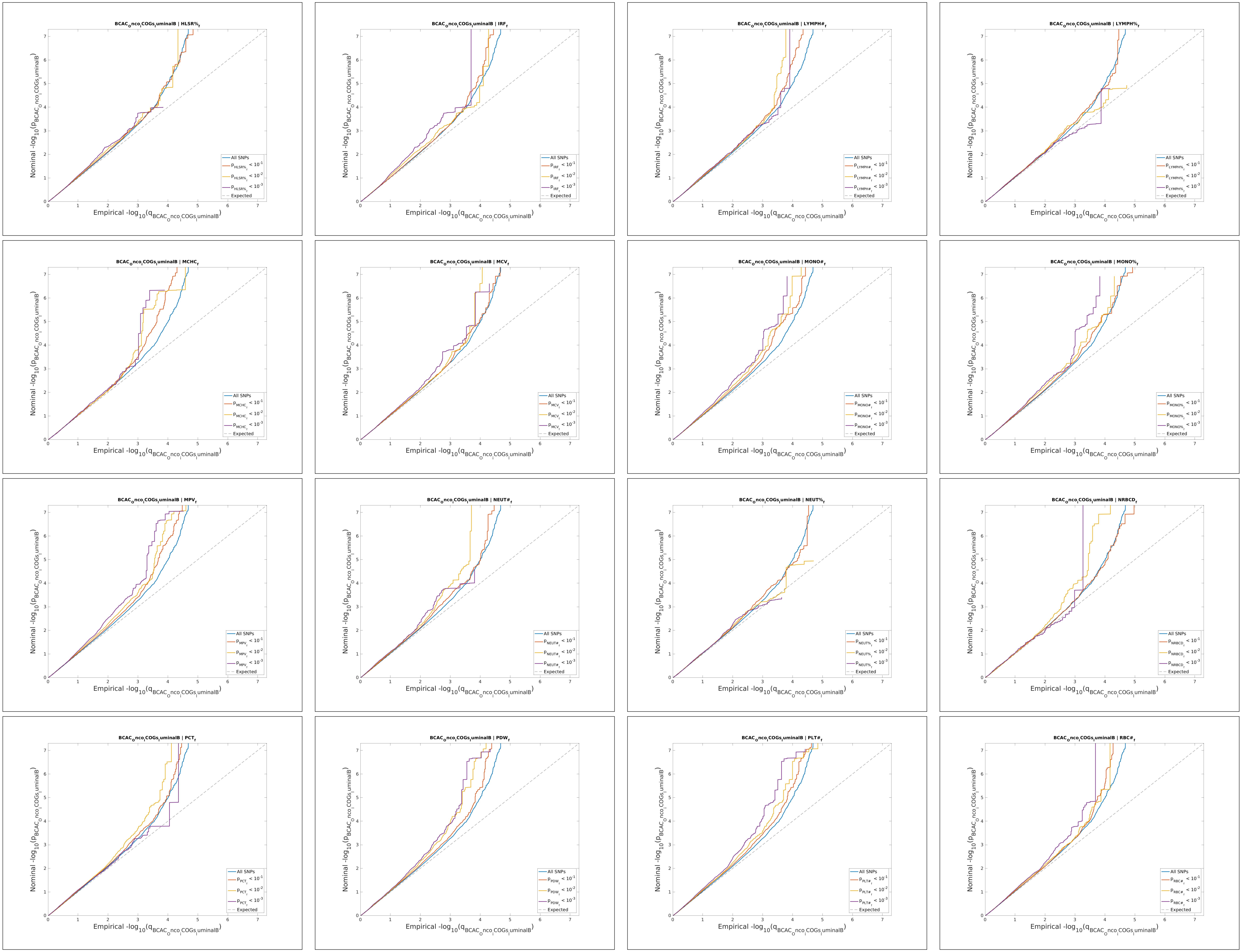

BCAC_onco_COGs_uminalB | HLSR%_f
BCAC_onco_COGs_uminalB | IRF_f
BCAC_onco_COGs_uminalB | LYMPH#_f
BCAC_onco_COGs_uminalB | LYMPH%_f
BCAC_onco_COGs_uminalB | MCHC_f
BCAC_onco_COGs_uminalB | MCV_f
BCAC_onco_COGs_uminalB | MONO#_f
BCAC_onco_COGs_uminalB | MONO%_f
BCAC_onco_COGs_uminalB | MPV_f
BCAC_onco_COGs_uminalB | NEUT#_f
BCAC_onco_COGs_uminalB | NEUT%_f
BCAC_onco_COGs_uminalB | NRBCD_f
BCAC_onco_COGs_uminalB | PCT_f
BCAC_onco_COGs_uminalB | PDW_f
BCAC_onco_COGs_uminalB | PLT#_f
BCAC_onco_COGs_uminalB | RBC#_f
Nominal -log10(p BCAC_onco_COGs_uminalB)
Empirical -log10(q BCAC_onco_COGs_uminalB)
All SNPs
Expected

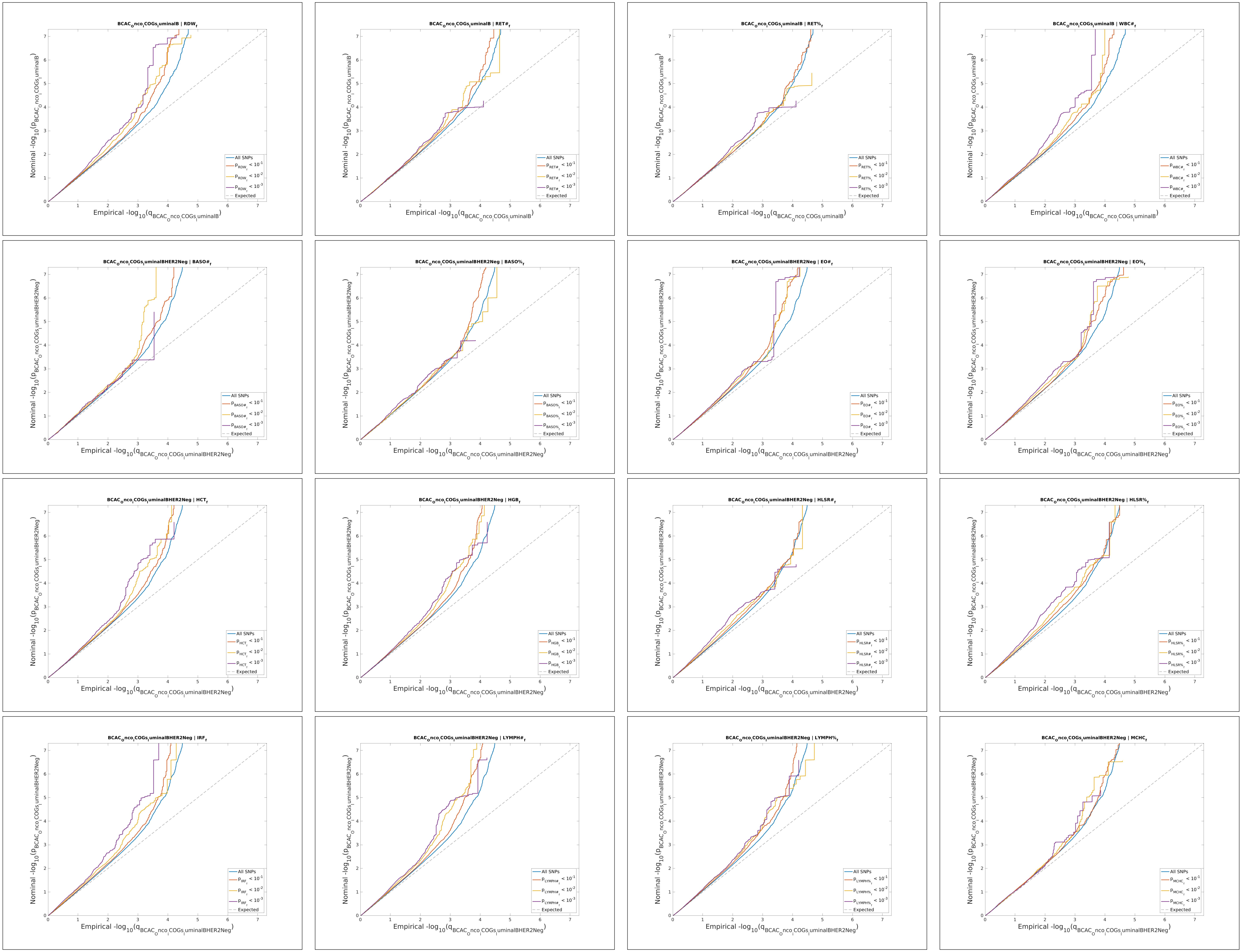

BCAC_onco_iCOGs_luminalB | RDW_f
BCAC_onco_iCOGs_luminalB | RET#_f
BCAC_onco_iCOGs_luminalB | RET%_f
BCAC_onco_iCOGs_luminalB | WBC#_f
BCAC_onco_iCOGs_luminalBHER2Neg | BASO#_f
BCAC_onco_iCOGs_luminalBHER2Neg | BASO%_f
BCAC_onco_iCOGs_luminalBHER2Neg | EO#_f
BCAC_onco_iCOGs_luminalBHER2Neg | EO%_f
BCAC_onco_iCOGs_luminalBHER2Neg | HCT_f
BCAC_onco_iCOGs_luminalBHER2Neg | HGB_f
BCAC_onco_iCOGs_luminalBHER2Neg | HLSR#_f
BCAC_onco_iCOGs_luminalBHER2Neg | HLSR%_f
BCAC_onco_iCOGs_luminalBHER2Neg | IRF_f
BCAC_onco_iCOGs_luminalBHER2Neg | LYMPH#_f
BCAC_onco_iCOGs_luminalBHER2Neg | LYMPH%_f
BCAC_onco_iCOGs_luminalBHER2Neg | MCHC_f

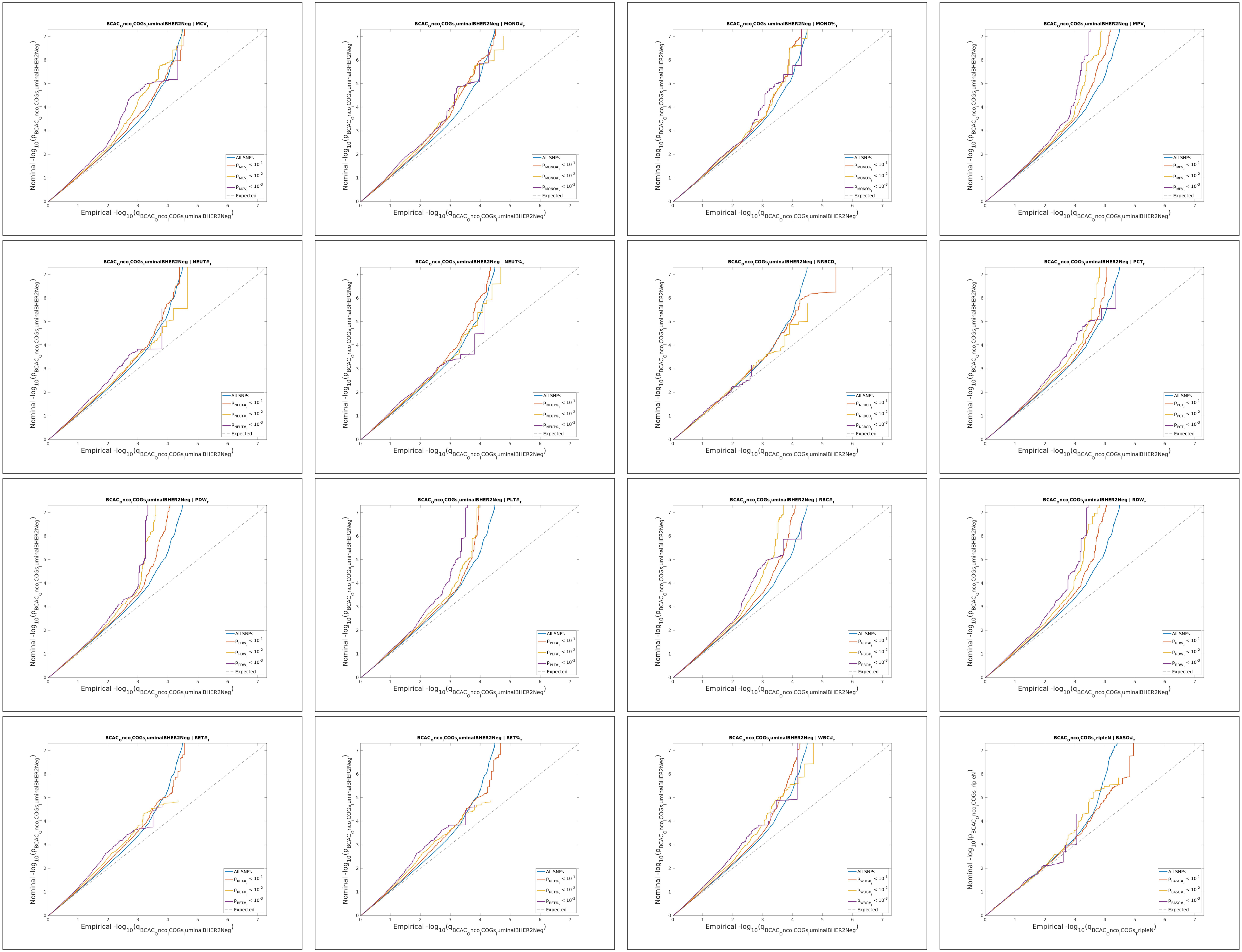

BCAConcoiCOGsluminalBHER2Neg | MCVf
BCAConcoiCOGsluminalBHER2Neg | MONO#f
BCAConcoiCOGsluminalBHER2Neg | MONO%f
BCAConcoiCOGsluminalBHER2Neg | MPVf
BCAConcoiCOGsluminalBHER2Neg | NEUT#f
BCAConcoiCOGsluminalBHER2Neg | NEUT%f
BCAConcoiCOGsluminalBHER2Neg | NRBCDf
BCAConcoiCOGsluminalBHER2Neg | PCTf
BCAConcoiCOGsluminalBHER2Neg | PDWf
BCAConcoiCOGsluminalBHER2Neg | PLT#f
BCAConcoiCOGsluminalBHER2Neg | RBC#f
BCAConcoiCOGsluminalBHER2Neg | RDWf
BCAConcoiCOGsluminalBHER2Neg | RET#f
BCAConcoiCOGsluminalBHER2Neg | RET%f
BCAConcoiCOGsluminalBHER2Neg | WBC#f
BCAConcoCOGsTripleN | BASO#f
Nominal -log10(pBCAConcoiCOGsluminalBHER2Neg)
Empirical -log10(qBCAConcoiCOGsluminalBHER2Neg)
All SNPs
Expected

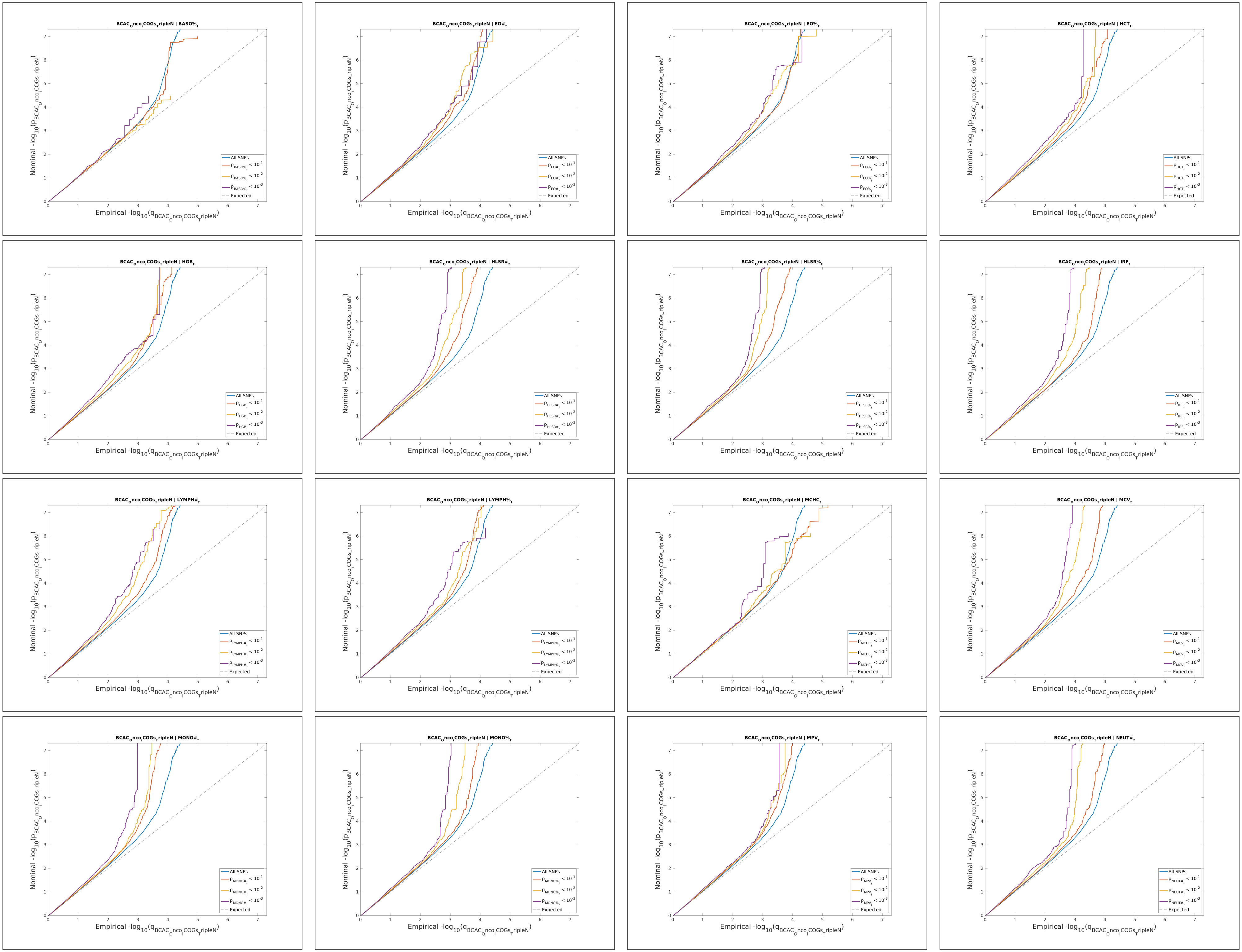
BCAConcoiCOGsTripleN | BASO%f
BCAConcoiCOGsTripleN | EO#f
BCAConcoiCOGsTripleN | EO%f
BCAConcoiCOGsTripleN | HCTf
BCAConcoiCOGsTripleN | HGBf
BCAConcoiCOGsTripleN | HLSR#f
BCAConcoiCOGsTripleN | HLSR%f
BCAConcoiCOGsTripleN | IRFf
BCAConcoiCOGsTripleN | LYMPH#f
BCAConcoiCOGsTripleN | LYMPH%f
BCAConcoiCOGsTripleN | MCHCf
BCAConcoiCOGsTripleN | MCVf
BCAConcoiCOGsTripleN | MONO#f
BCAConcoiCOGsTripleN | MONO%f
BCAConcoiCOGsTripleN | MPVf
BCAConcoiCOGsTripleN | NEUT#f
Nominal -log10(pBCAConcoiCOGsTripleN)
Empirical -log10(qBCAConcoiCOGsTripleN)
All SNPs
Expected

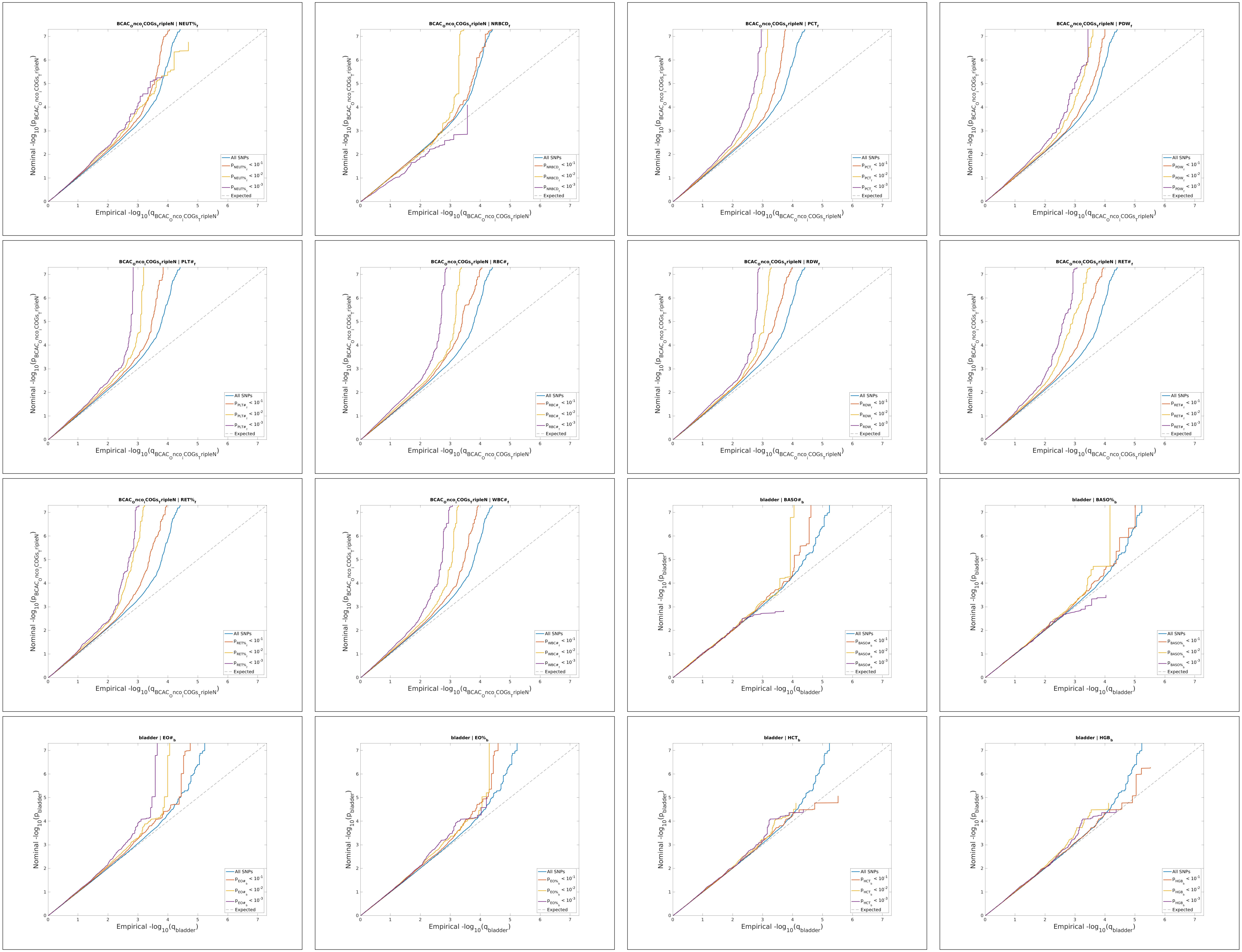
BCAConcoiCOGsTripleN | NEUT%f
BCAConcoiCOGsTripleN | NRBCDf
BCAConcoiCOGsTripleN | PCTf
BCAConcoiCOGsTripleN | PDWf
BCAConcoiCOGsTripleN | PLT#f
BCAConcoiCOGsTripleN | RBC#f
BCAConcoiCOGsTripleN | RDWf
BCAConcoiCOGsTripleN | RET#f
BCAConcoiCOGsTripleN | RET%f
BCAConcoiCOGsTripleN | WBC#f
bladder | BASO#b
bladder | BASO%b
bladder | EO#b
bladder | EO%b
bladder | HCTb
bladder | HGBb
Nominal -log10(p)
Empirical -log10(q)
All SNPs
Expected

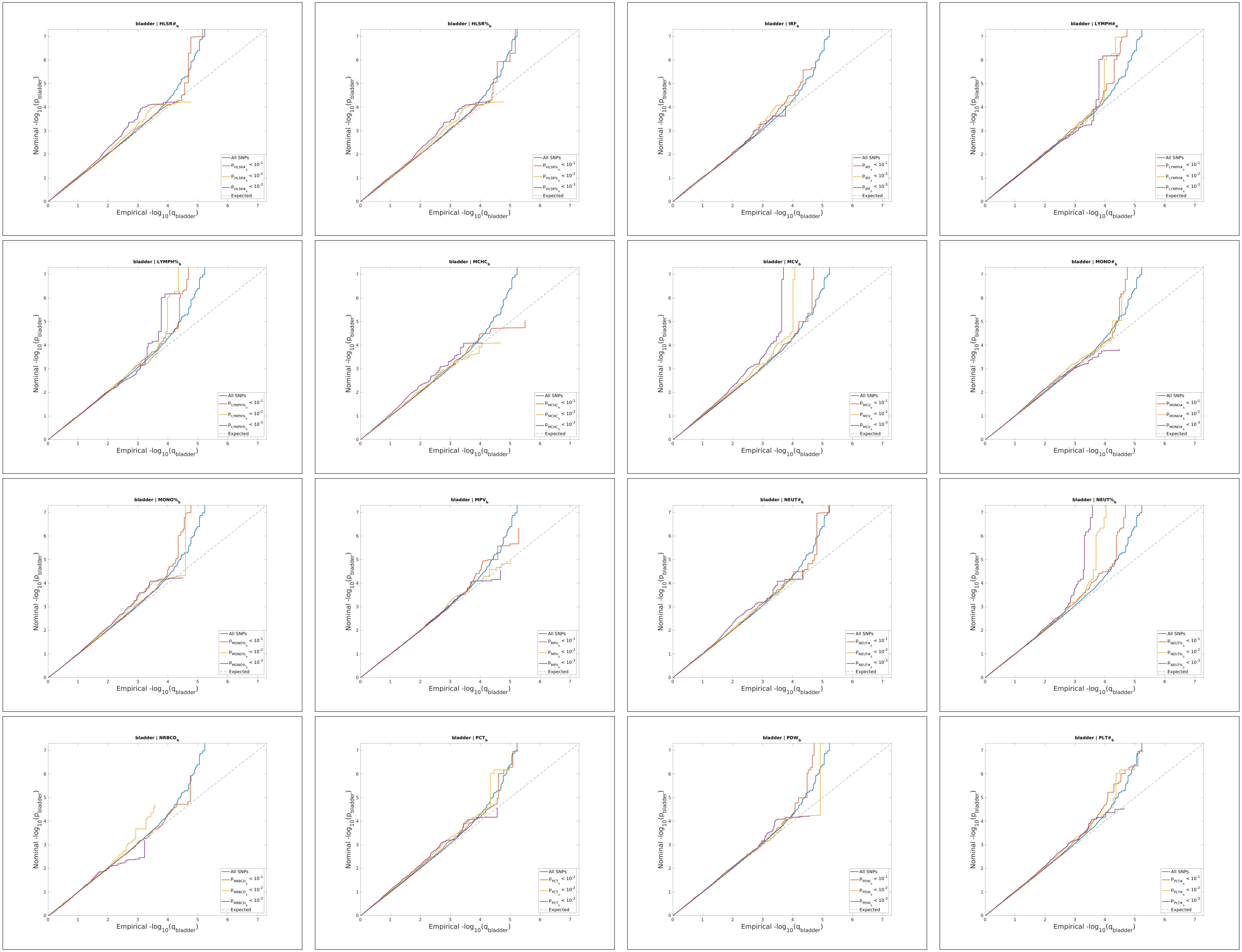
bladder | HLSR#b
bladder | HLSR%b
bladder | IRFb
bladder | LYMPH#b
bladder | LYMPH%b
bladder | MCHCb
bladder | MCVb
bladder | MONO#b
bladder | MONO%b
bladder | MPVb
bladder | NEUT#b
bladder | NEUT%b
bladder | NRBCDb
bladder | PCTb
bladder | PDWb
bladder | PLT#b
Nominal -log10(pbladder)
Empirical -log10(qbladder)
All SNPs
Expected

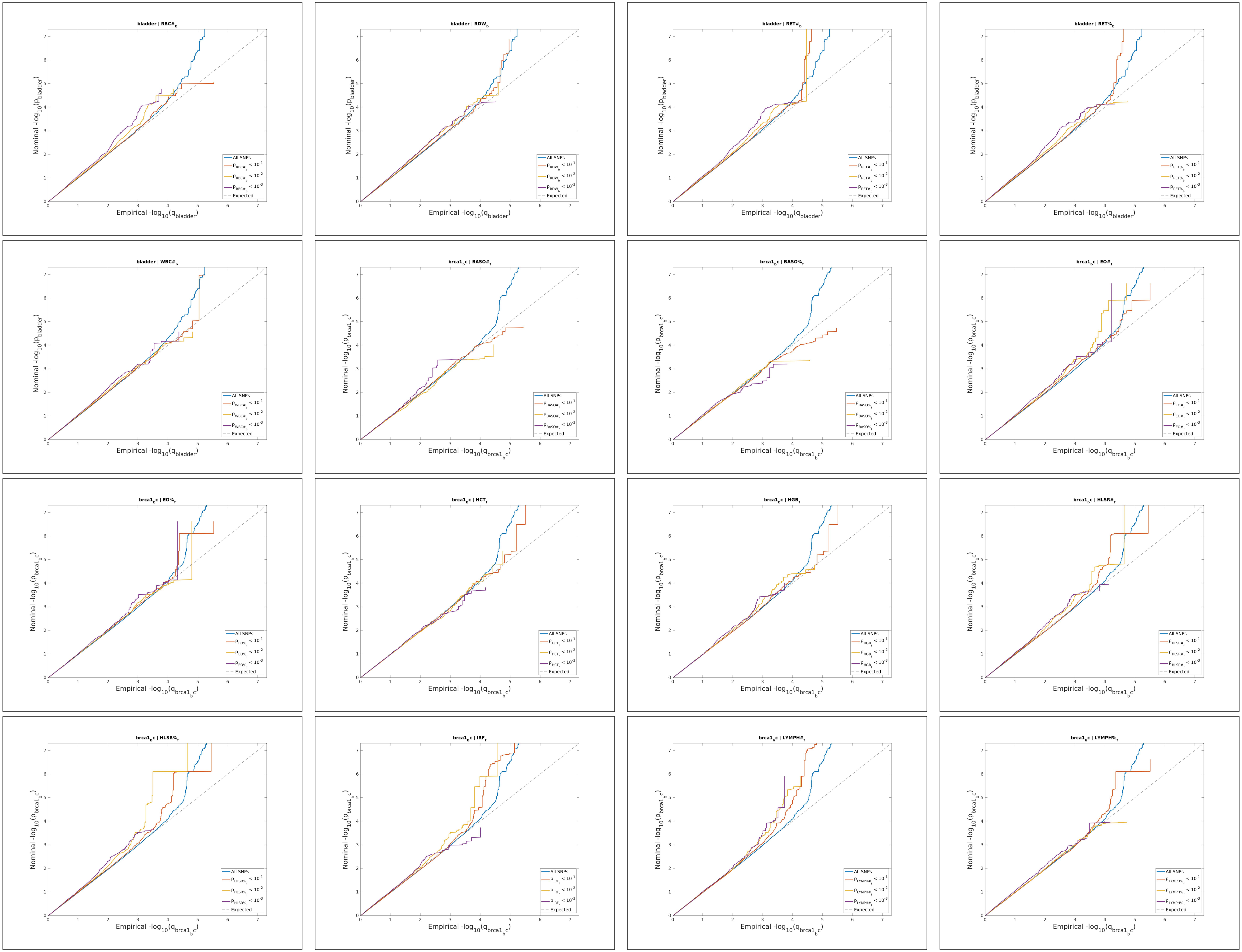

bladder | $RBC\#_b$
bladder | RDW_b
bladder | $RET\#_b$
bladder | $RET\%_b$
bladder | $WBC\#_b$
$brca1_bc$ | $BASO\#_f$
$brca1_bc$ | $BASO\%_f$
$brca1_bc$ | $EO\#_f$
$brca1_bc$ | $EO\%_f$
$brca1_bc$ | HCT_f
$brca1_bc$ | HGB_f
$brca1_bc$ | $HLSR\#_f$
$brca1_bc$ | $HLSR\%_f$
$brca1_bc$ | IRF_f
$brca1_bc$ | $LYMPH\#_f$
$brca1_bc$ | $LYMPH\%_f$
Nominal $-\log_{10}(p_{bladder})$
Empirical $-\log_{10}(q_{bladder})$
Nominal $-\log_{10}(p_{brca1_bc})$
Empirical $-\log_{10}(q_{brca1_bc})$
All SNPs
$p < 10^{-1}$
$p < 10^{-2}$
$p < 10^{-3}$
Expected

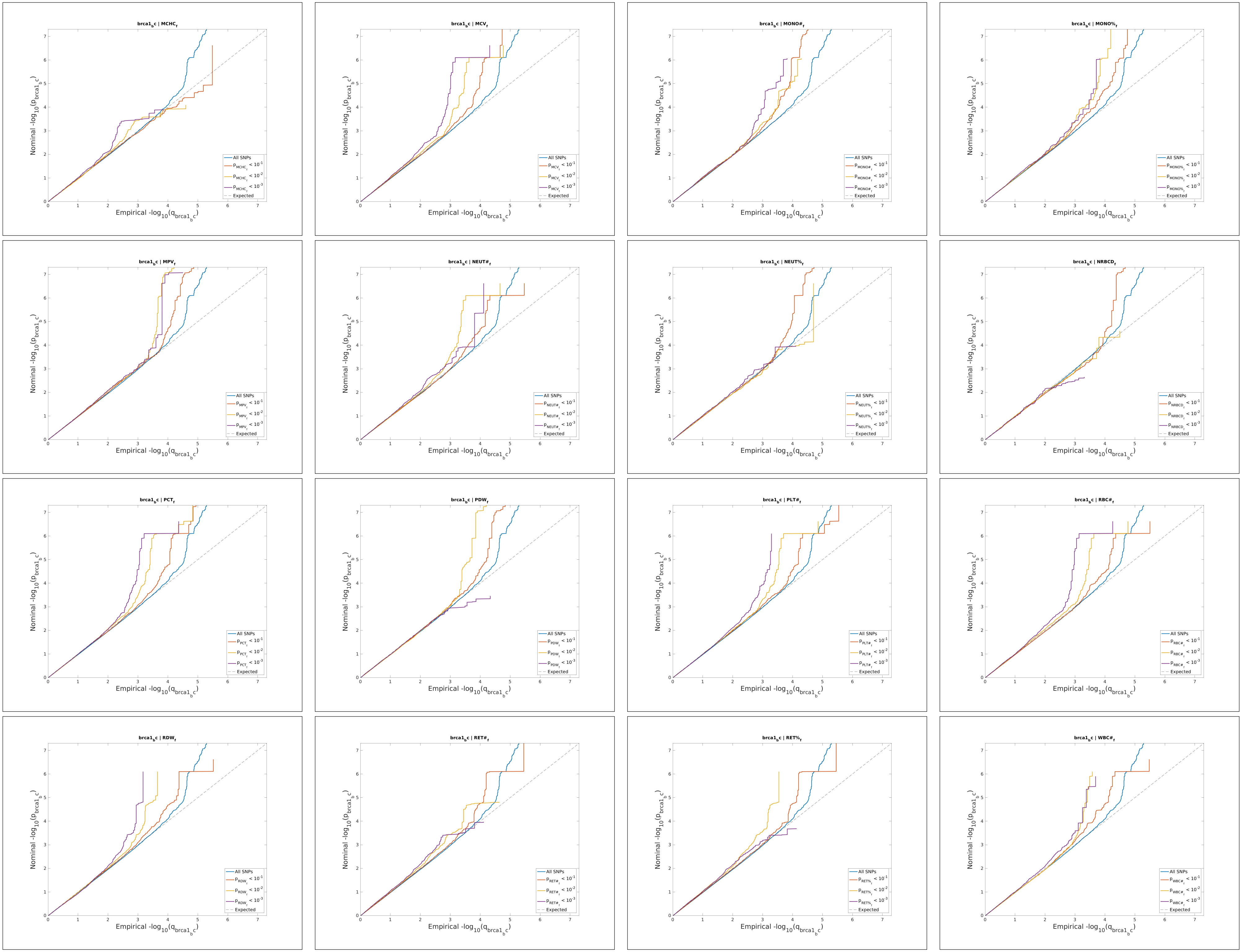
brca1bc | MCHCf
brca1bc | MCVf
brca1bc | MONO#f
brca1bc | MONO%f
brca1bc | MPVf
brca1bc | NEUT#f
brca1bc | NEUT%f
brca1bc | NRBCDf
brca1bc | PCTf
brca1bc | PDWf
brca1bc | PLT#f
brca1bc | RBC#f
brca1bc | RDWf
brca1bc | RET#f
brca1bc | RET%f
brca1bc | WBC#f
Nominal -log10(pbrca1bc)
Empirical -log10(qbrca1bc)
All SNPs
Expected

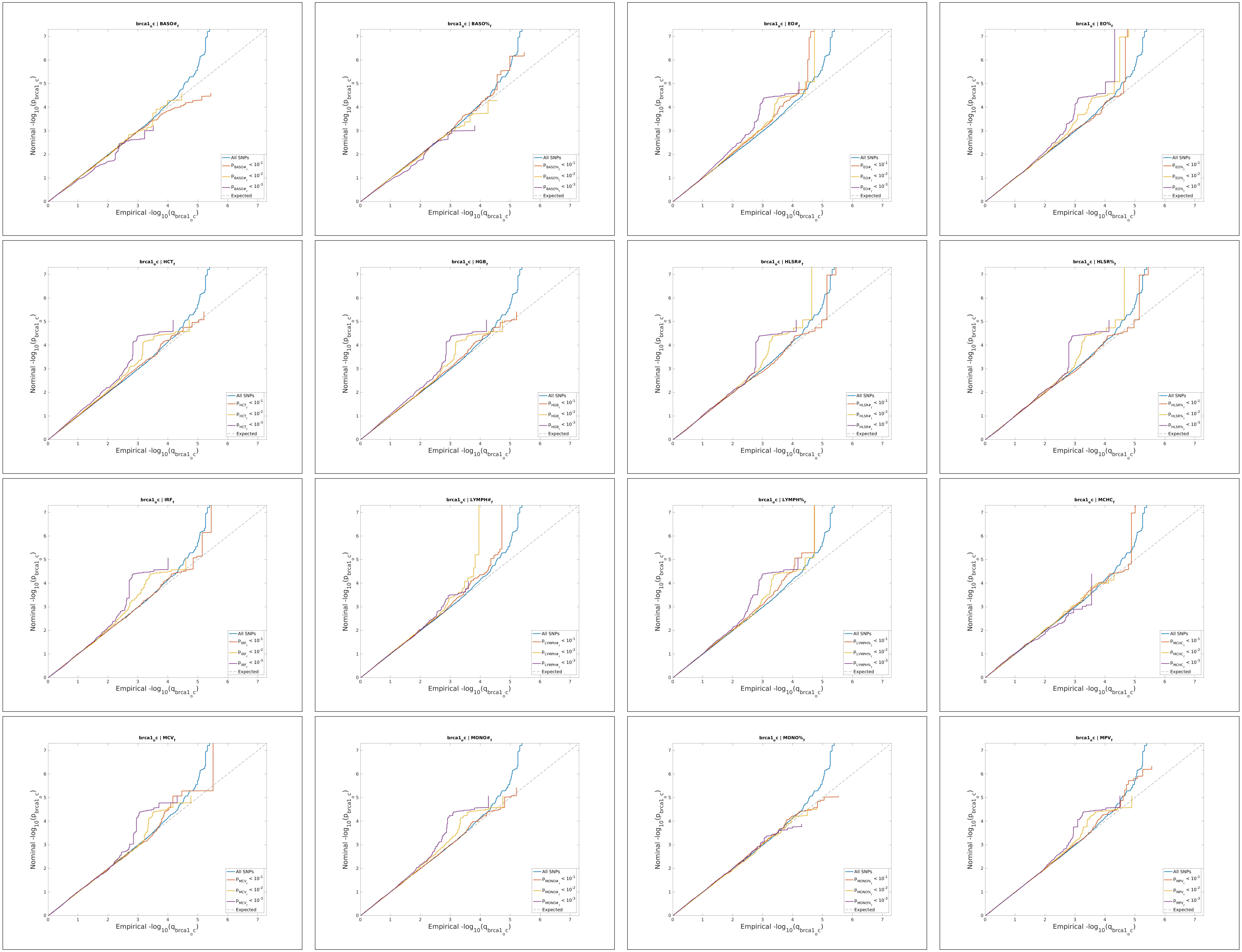
brca1_oc | BASO#_f
brca1_oc | BASO%_f
brca1_oc | EO#_f
brca1_oc | EO%_f
brca1_oc | HCT_f
brca1_oc | HGB_f
brca1_oc | HLSR#_f
brca1_oc | HLSR%_f
brca1_oc | IRF_f
brca1_oc | LYMPH#_f
brca1_oc | LYMPH%_f
brca1_oc | MCHC_f
brca1_oc | MCV_f
brca1_oc | MONO#_f
brca1_oc | MONO%_f
brca1_oc | MPV_f

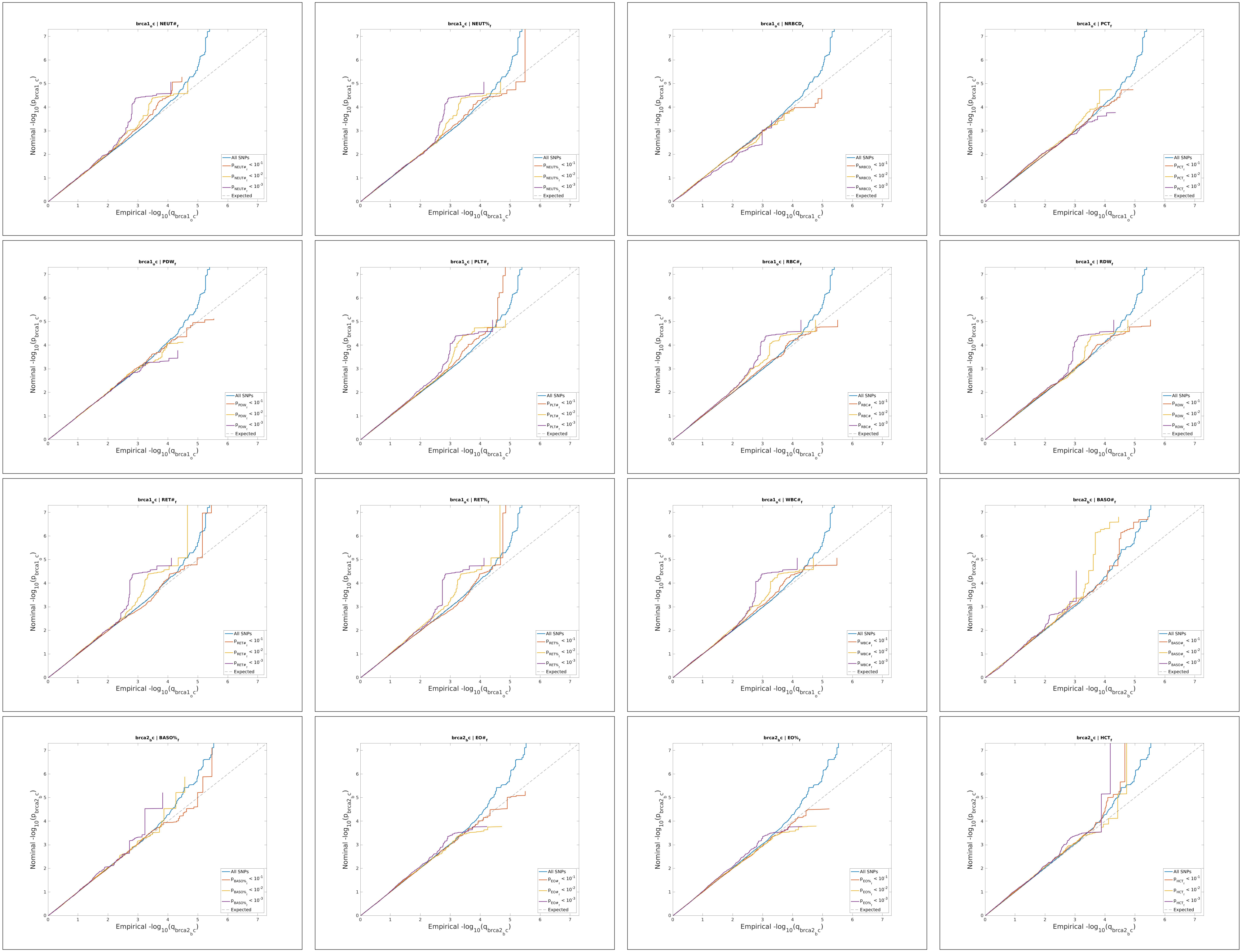

brca1_oc | NEUT#_f
brca1_oc | NEUT%_f
brca1_oc | NRBCD_f
brca1_oc | PCT_f
brca1_oc | PDW_f
brca1_oc | PLT#_f
brca1_oc | RBC#_f
brca1_oc | RDW_f
brca1_oc | RET#_f
brca1_oc | RET%_f
brca1_oc | WBC#_f
brca2_bc | BASO#_f
brca2_bc | BASO%_f
brca2_bc | EO#_f
brca2_bc | EO%_f
brca2_bc | HCT_f
Nominal -log10(p)
Empirical -log10(q)
All SNPs
Expected

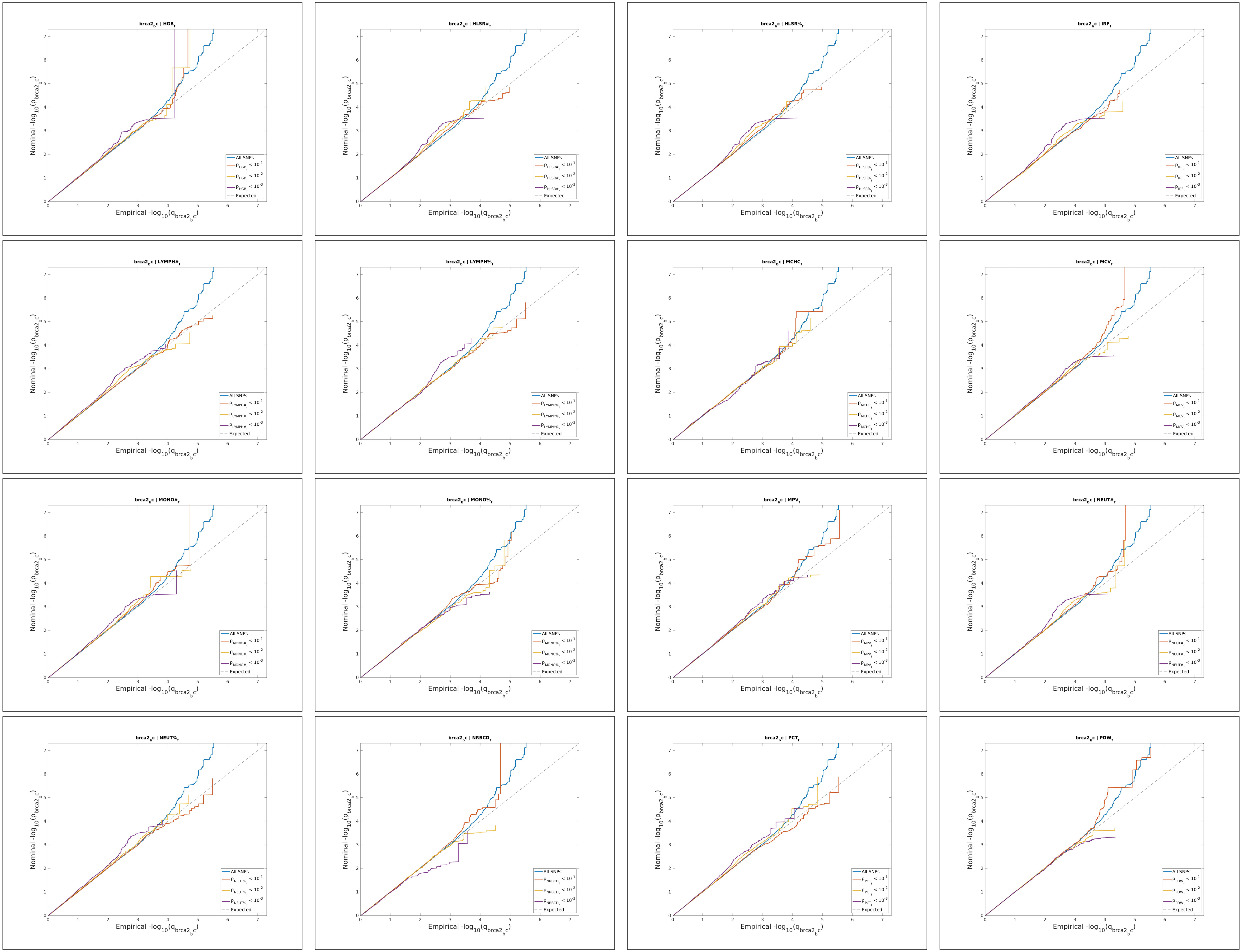
brca2_bc | HGB_f
brca2_bc | HLSR#_f
brca2_bc | HLSR%_f
brca2_bc | IRF_f
brca2_bc | LYMPH#_f
brca2_bc | LYMPH%_f
brca2_bc | MCHC_f
brca2_bc | MCV_f
brca2_bc | MONO#_f
brca2_bc | MONO%_f
brca2_bc | MPV_f
brca2_bc | NEUT#_f
brca2_bc | NEUT%_f
brca2_bc | NRBCD_f
brca2_bc | PCT_f
brca2_bc | PDW_f
Nominal -log10(p brca2_bc)
Empirical -log10(q brca2_bc)
All SNPs
Expected

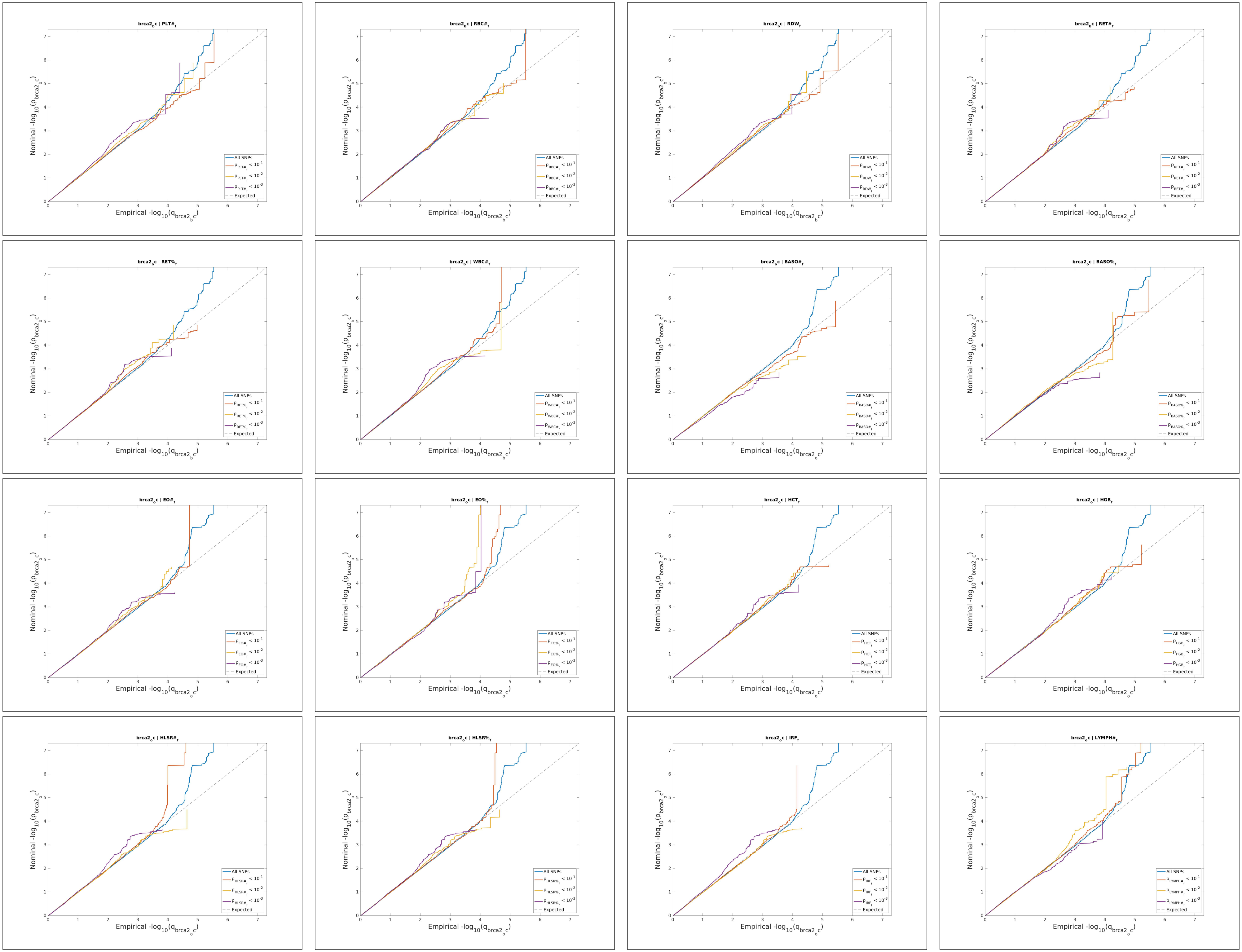

brca2bc | PLT#f
brca2bc | RBC#f
brca2bc | RDWf
brca2bc | RET#f
brca2bc | RET%f
brca2bc | WBC#f
brca2oc | BASO#f
brca2oc | BASO%f
brca2oc | EO#f
brca2oc | EO%f
brca2oc | HCTf
brca2oc | HGBf
brca2oc | HLSR#f
brca2oc | HLSR%f
brca2oc | IRFf
brca2oc | LYMPH#f
Nominal -log10(pbrca2bc)
Empirical -log10(qbrca2bc)
Nominal -log10(pbrca2oc)
Empirical -log10(qbrca2oc)
All SNPs
Expected

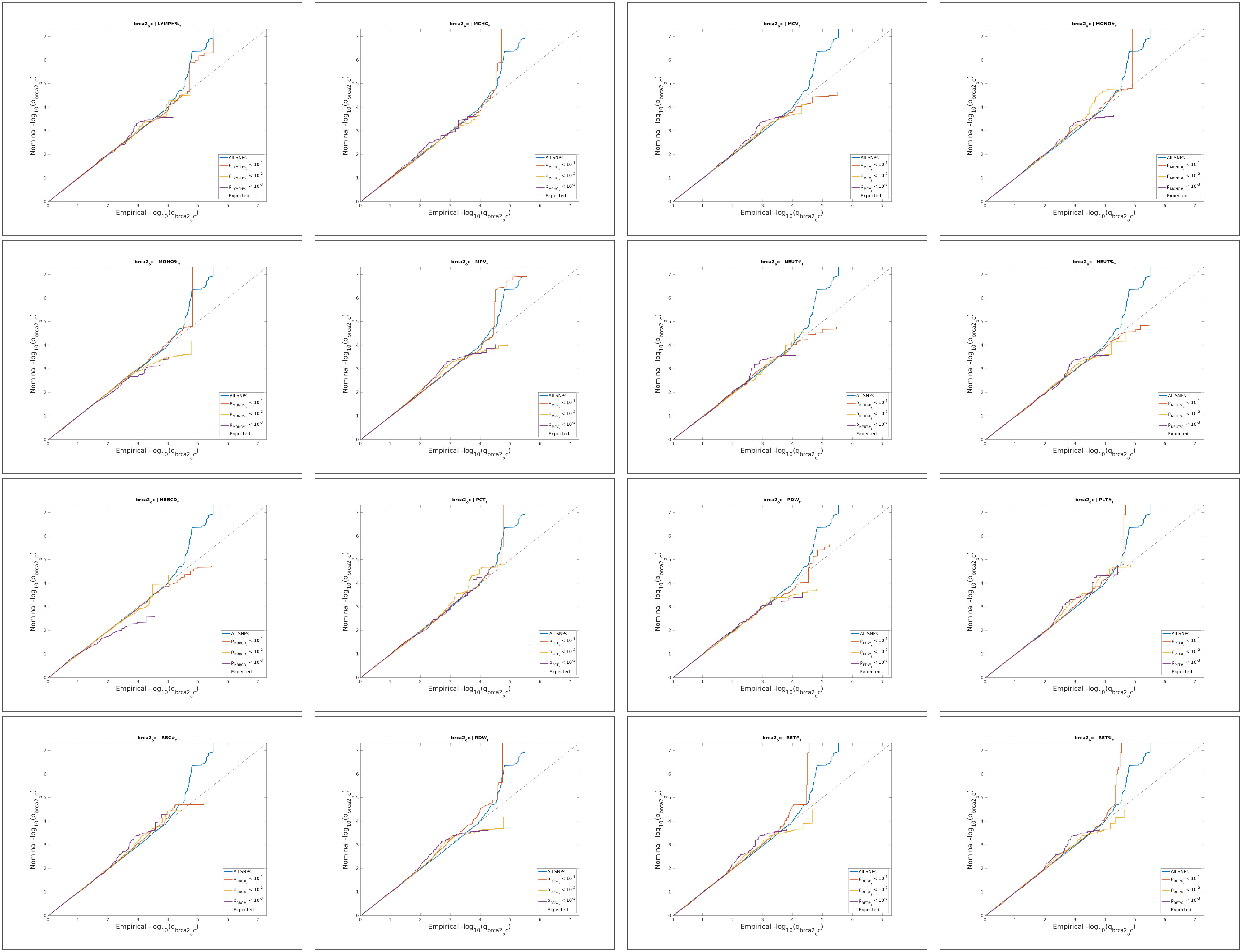
brca2oc | LYMPH%f
brca2oc | MCHCf
brca2oc | MCVf
brca2oc | MONO#f
brca2oc | MONO%f
brca2oc | MPVf
brca2oc | NEUT#f
brca2oc | NEUT%f
brca2oc | NRBCDf
brca2oc | PCTf
brca2oc | PDWf
brca2oc | PLT#f
brca2oc | RBC#f
brca2oc | RDWf
brca2oc | RET#f
brca2oc | RET%f
Nominal -log10(pbrca2oc)
Empirical -log10(qbrca2oc)
All SNPs
Expected

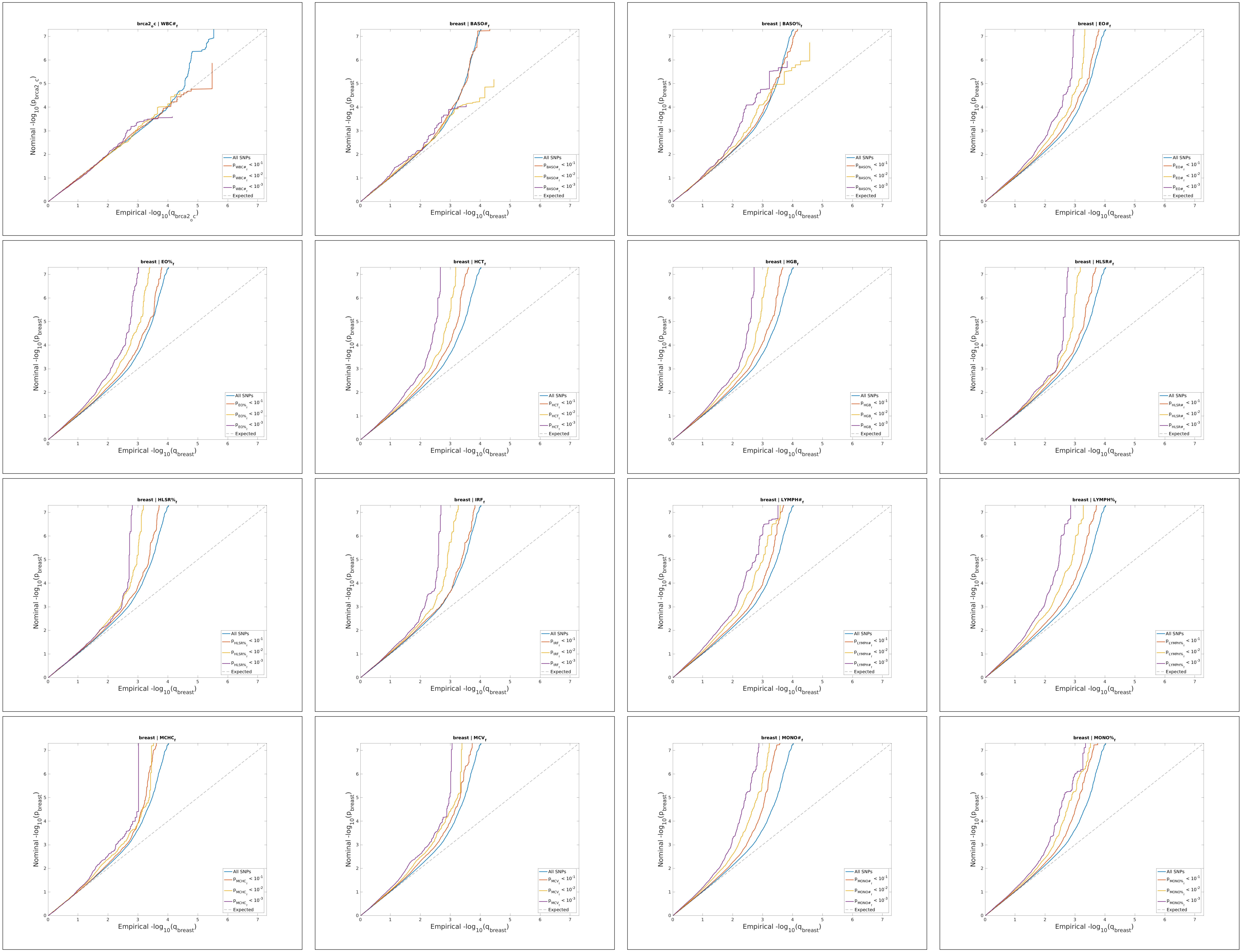

brca2_c | WBC#_f
breast | BASO#_f
breast | BASO%_f
breast | EO#_f
breast | EO%_f
breast | HCT_f
breast | HGB_f
breast | HLSR#_f
breast | HLSR%_f
breast | IRF_f
breast | LYMPH#_f
breast | LYMPH%_f
breast | MCHC_f
breast | MCV_f
breast | MONO#_f
breast | MONO%_f
Nominal -log10(p_brca2_c)
Empirical -log10(q_brca2_c)
Nominal -log10(p_breast)
Empirical -log10(q_breast)
All SNPs
< 10^-1
< 10^-2
< 10^-3
Expected

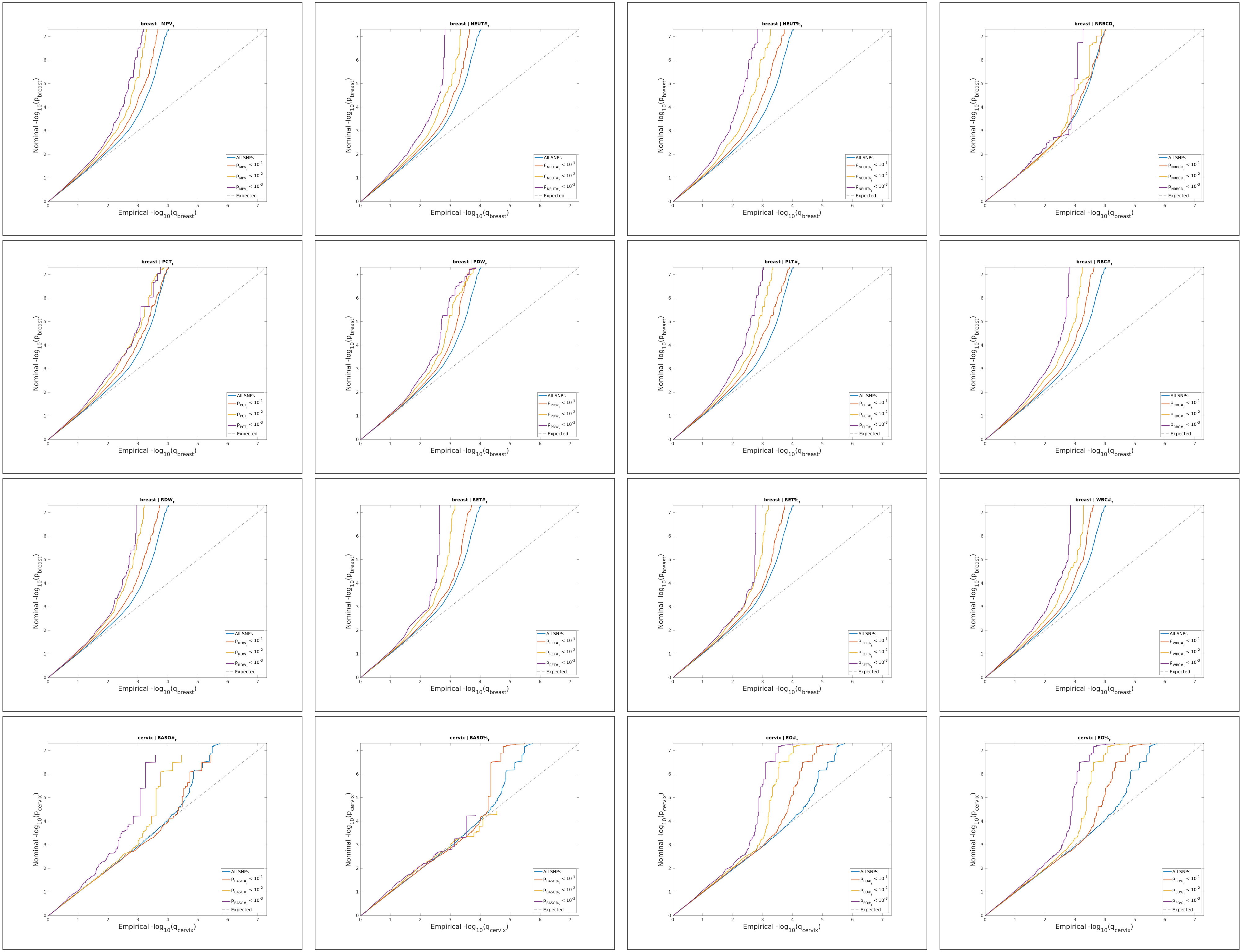
breast | MPVf
breast | NEUT#f
breast | NEUT%f
breast | NRBCDf
breast | PCTf
breast | PDWf
breast | PLT#f
breast | RBC#f
breast | RDWf
breast | RET#f
breast | RET%f
breast | WBC#f
cervix | BASO#f
cervix | BASO%f
cervix | EO#f
cervix | EO%f
Nominal -log10(pbreast)
Empirical -log10(qbreast)
Nominal -log10(pcervix)
Empirical -log10(qcervix)
All SNPs
Expected

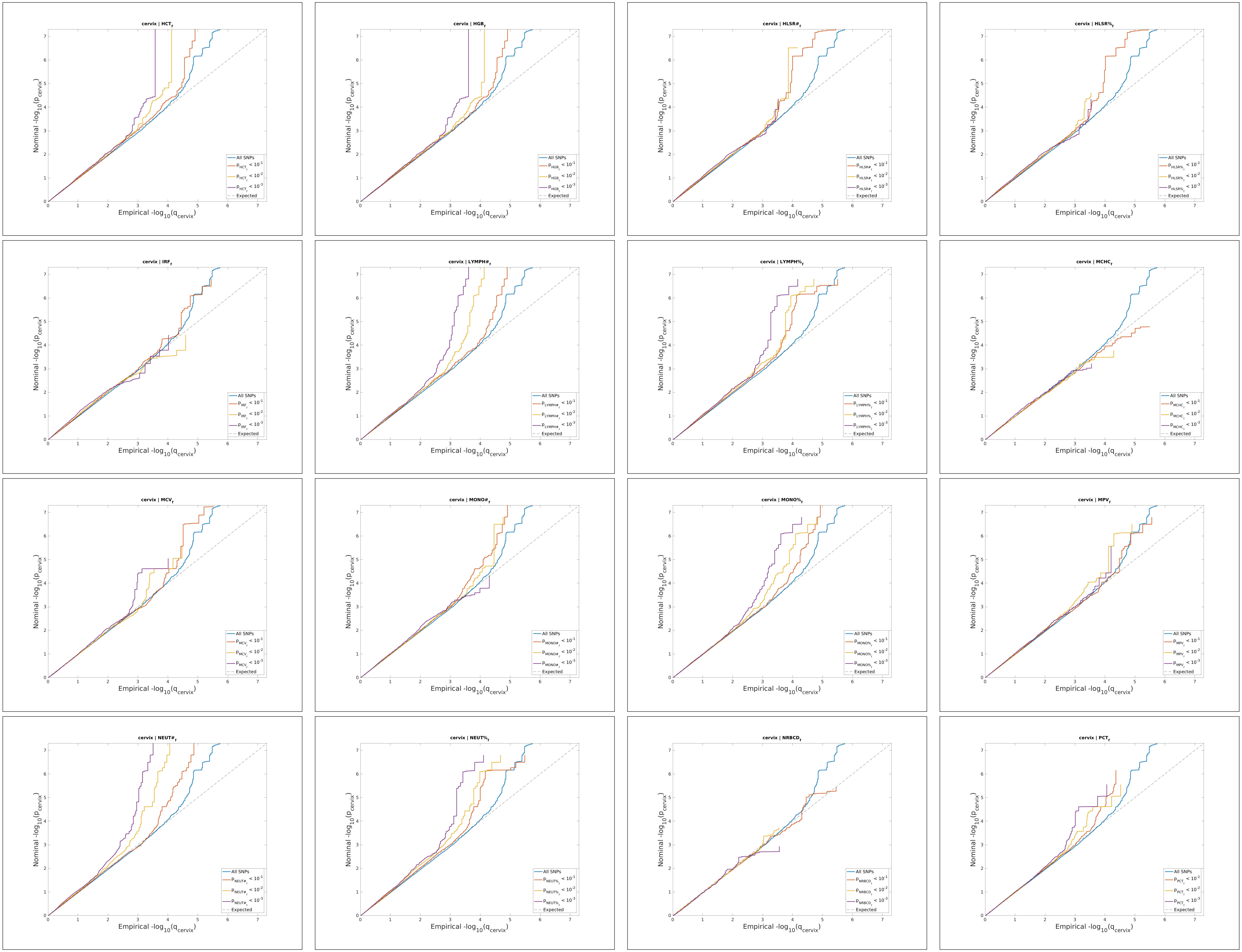

cervix | HCT_f
All SNPs
p_HCT_f < 10^-1
p_HCT_f < 10^-2
p_HCT_f < 10^-3
Expected
cervix | HGB_f
All SNPs
p_HGB_f < 10^-1
p_HGB_f < 10^-2
p_HGB_f < 10^-3
Expected
cervix | HLSR#_f
All SNPs
p_HLSR#_f < 10^-1
p_HLSR#_f < 10^-2
p_HLSR#_f < 10^-3
Expected
cervix | HLSR%_f
All SNPs
p_HLSR%_f < 10^-1
p_HLSR%_f < 10^-2
p_HLSR%_f < 10^-3
Expected
cervix | IRF_f
All SNPs
p_IRF_f < 10^-1
p_IRF_f < 10^-2
p_IRF_f < 10^-3
Expected
cervix | LYMPH#_f
All SNPs
p_LYMPH#_f < 10^-1
p_LYMPH#_f < 10^-2
p_LYMPH#_f < 10^-3
Expected
cervix | LYMPH%_f
All SNPs
p_LYMPH%_f < 10^-1
p_LYMPH%_f < 10^-2
p_LYMPH%_f < 10^-3
Expected
cervix | MCHC_f
All SNPs
p_MCHC_f < 10^-1
p_MCHC_f < 10^-2
p_MCHC_f < 10^-3
Expected
cervix | MCV_f
All SNPs
p_MCV_f < 10^-1
p_MCV_f < 10^-2
p_MCV_f < 10^-3
Expected
cervix | MONO#_f
All SNPs
p_MONO#_f < 10^-1
p_MONO#_f < 10^-2
p_MONO#_f < 10^-3
Expected
cervix | MONO%_f
All SNPs
p_MONO%_f < 10^-1
p_MONO%_f < 10^-2
p_MONO%_f < 10^-3
Expected
cervix | MPV_f
All SNPs
p_MPV_f < 10^-1
p_MPV_f < 10^-2
p_MPV_f < 10^-3
Expected
cervix | NEUT#_f
All SNPs
p_NEUT#_f < 10^-1
p_NEUT#_f < 10^-2
p_NEUT#_f < 10^-3
Expected
cervix | NEUT%_f
All SNPs
p_NEUT%_f < 10^-1
p_NEUT%_f < 10^-2
p_NEUT%_f < 10^-3
Expected
cervix | NRBCD_f
All SNPs
p_NRBCD_f < 10^-1
p_NRBCD_f < 10^-2
p_NRBCD_f < 10^-3
Expected
cervix | PCT_f
All SNPs
p_PCT_f < 10^-1
p_PCT_f < 10^-2
p_PCT_f < 10^-3
Expected
Nominal -log10(p_cervix)
Empirical -log10(q_cervix)

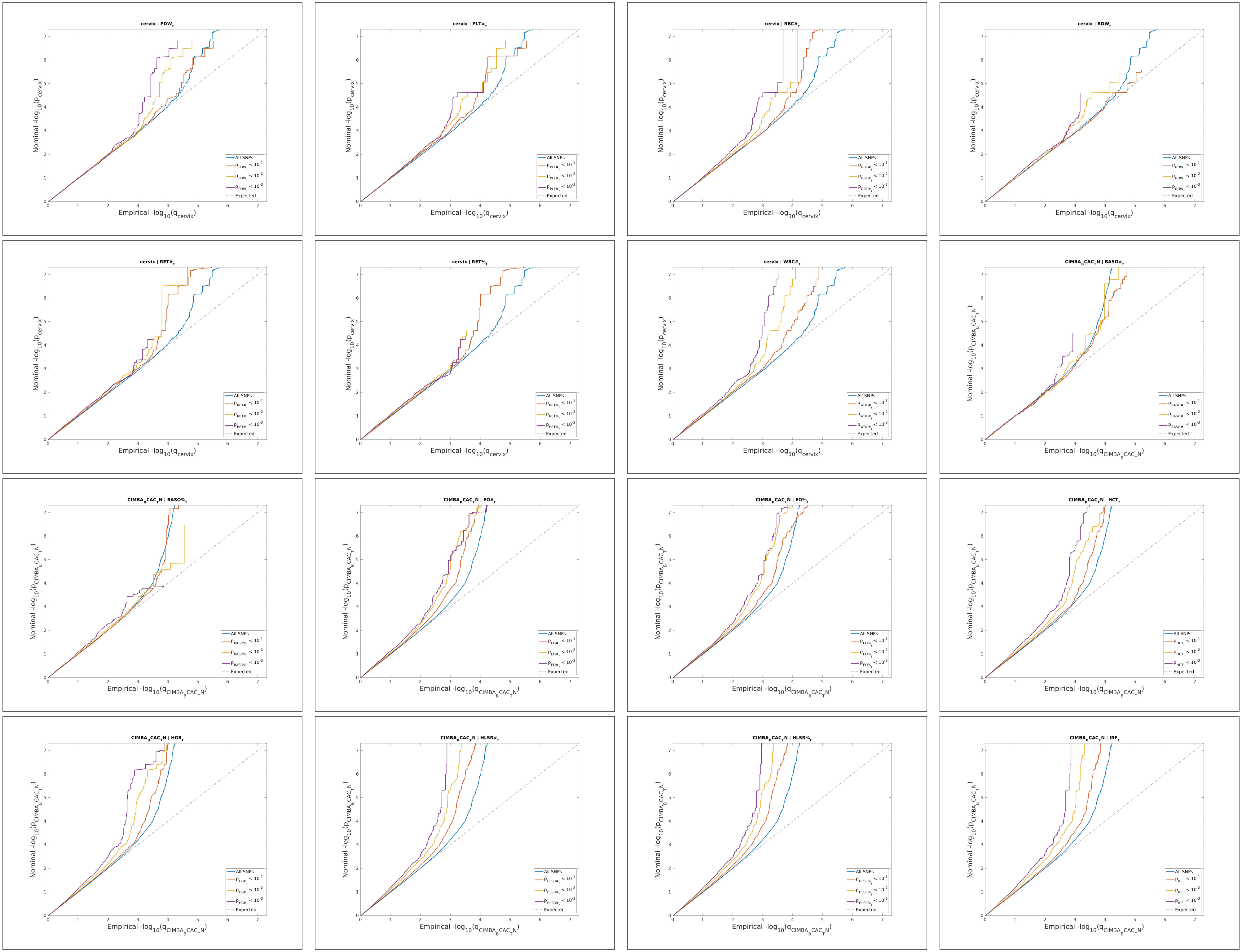

cervix | PDW_f
cervix | PLT#_f
cervix | RBC#_f
cervix | RDW_f
cervix | RET#_f
cervix | RET%_f
cervix | WBC#_f
CIMBA_BCAC_TN | BASO#_f
CIMBA_BCAC_TN | BASO%_f
CIMBA_BCAC_TN | EO#_f
CIMBA_BCAC_TN | EO%_f
CIMBA_BCAC_TN | HCT_f
CIMBA_BCAC_TN | HGB_f
CIMBA_BCAC_TN | HLSR#_f
CIMBA_BCAC_TN | HLSR%_f
CIMBA_BCAC_TN | IRF_f
Nominal -log10(p_cervix)
Empirical -log10(q_cervix)
Nominal -log10(p_CIMBA_BCAC_TN)
Empirical -log10(q_CIMBA_BCAC_TN)
All SNPs
p < 10^-1
p < 10^-2
p < 10^-3
Expected

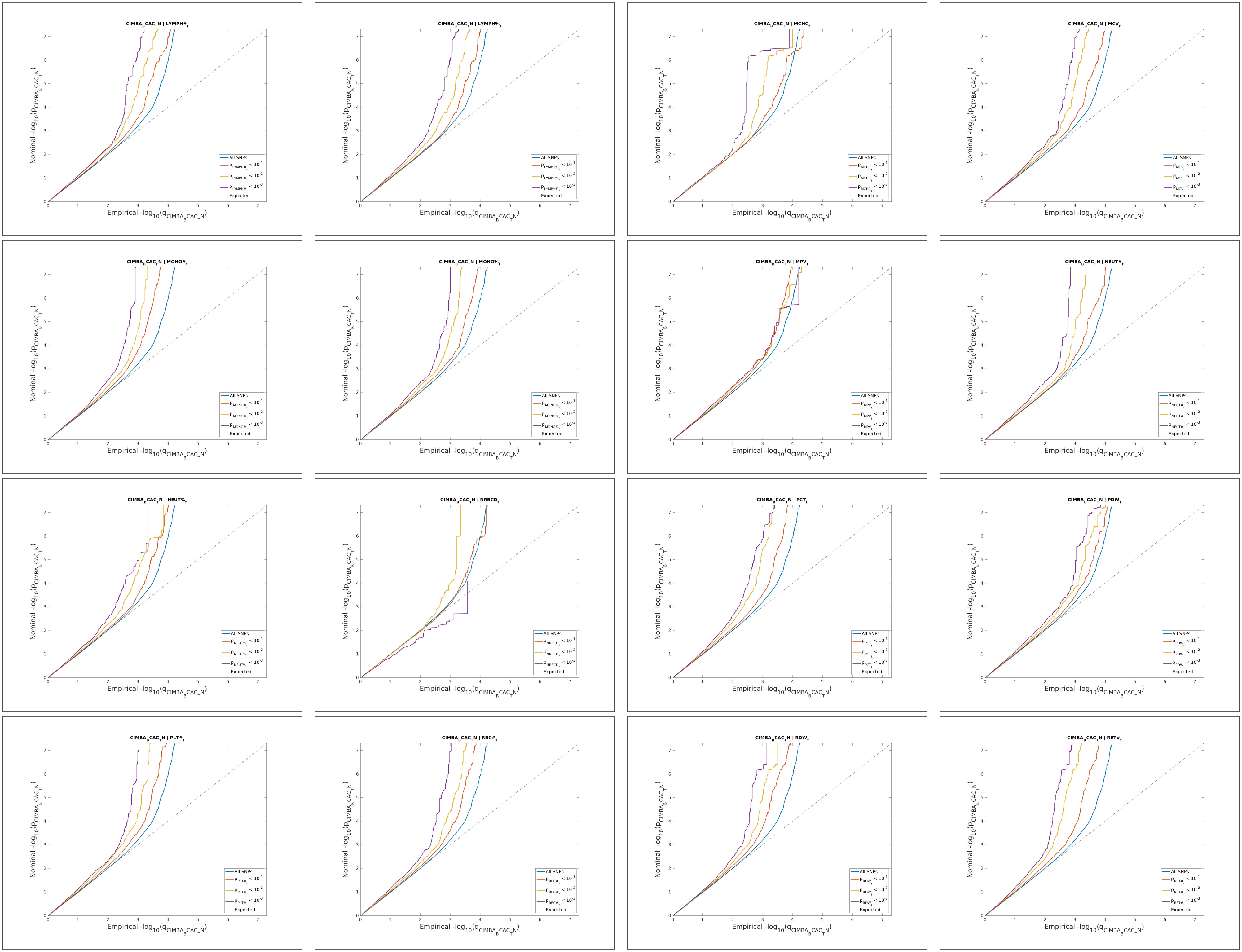

CIMBA_BCAC_TN | LYMPH#_f
CIMBA_BCAC_TN | LYMPH%_f
CIMBA_BCAC_TN | MCHC_f
CIMBA_BCAC_TN | MCV_f
CIMBA_BCAC_TN | MONO#_f
CIMBA_BCAC_TN | MONO%_f
CIMBA_BCAC_TN | MPV_f
CIMBA_BCAC_TN | NEUT#_f
CIMBA_BCAC_TN | NEUT%_f
CIMBA_BCAC_TN | NRBCD_f
CIMBA_BCAC_TN | PCT_f
CIMBA_BCAC_TN | PDW_f
CIMBA_BCAC_TN | PLT#_f
CIMBA_BCAC_TN | RBC#_f
CIMBA_BCAC_TN | RDW_f
CIMBA_BCAC_TN | RET#_f
Nominal -log10(p CIMBA_BCAC_TN)
Empirical -log10(q CIMBA_BCAC_TN)
All SNPs
p < 10^-1
p < 10^-2
p < 10^-3
Expected

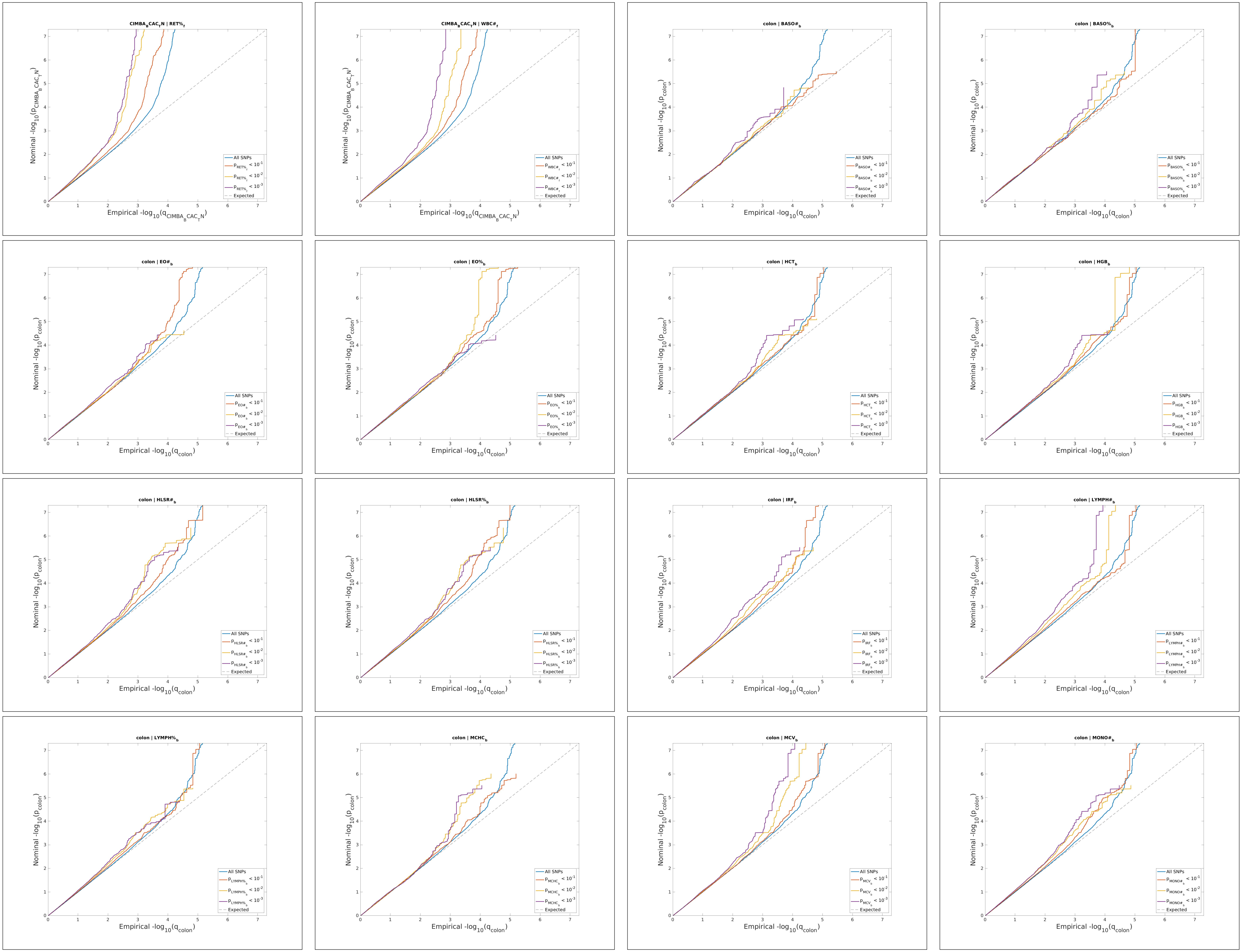
CIMBA_B CAC_T N | RET%_f
CIMBA_B CAC_T N | WBC#_f
colon | BASO#_b
colon | BASO%_b
colon | EO#_b
colon | EO%_b
colon | HCT_b
colon | HGB_b
colon | HLSR#_b
colon | HLSR%_b
colon | IRF_b
colon | LYMPH#_b
colon | LYMPH%_b
colon | MCHC_b
colon | MCV_b
colon | MONO#_b
Nominal -log10(p)
Empirical -log10(q)
All SNPs
Expected

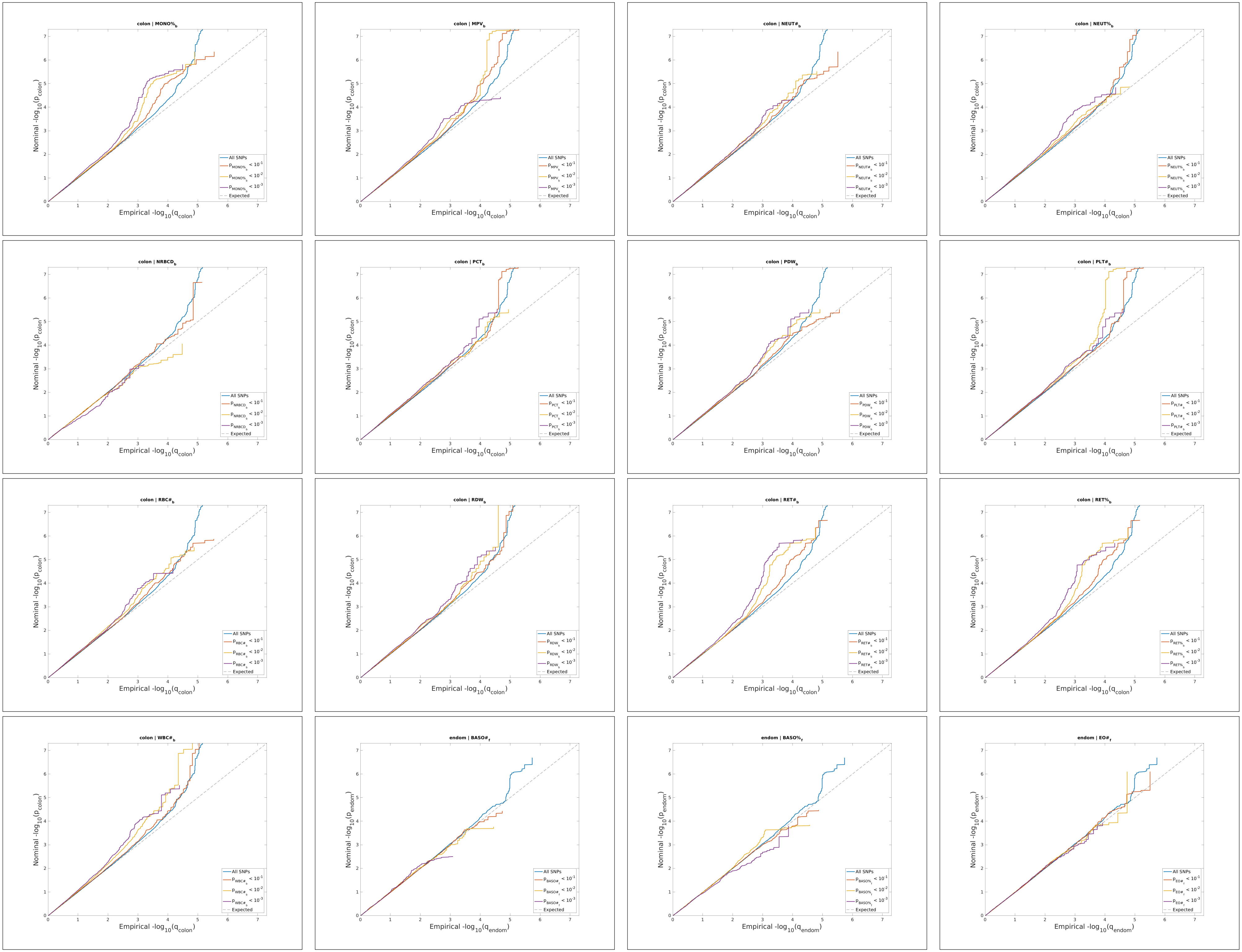

colon | MONO%b
colon | MPVb
colon | NEUT#b
colon | NEUT%b
colon | NRBCDb
colon | PCTb
colon | PDWb
colon | PLT#b
colon | RBC#b
colon | RDWb
colon | RET#b
colon | RET%b
colon | WBC#b
endom | BASO#f
endom | BASO%f
endom | EO#f
Nominal -log10(pcolon)
Empirical -log10(qcolon)
Nominal -log10(pendom)
Empirical -log10(qendom)
All SNPs
Expected

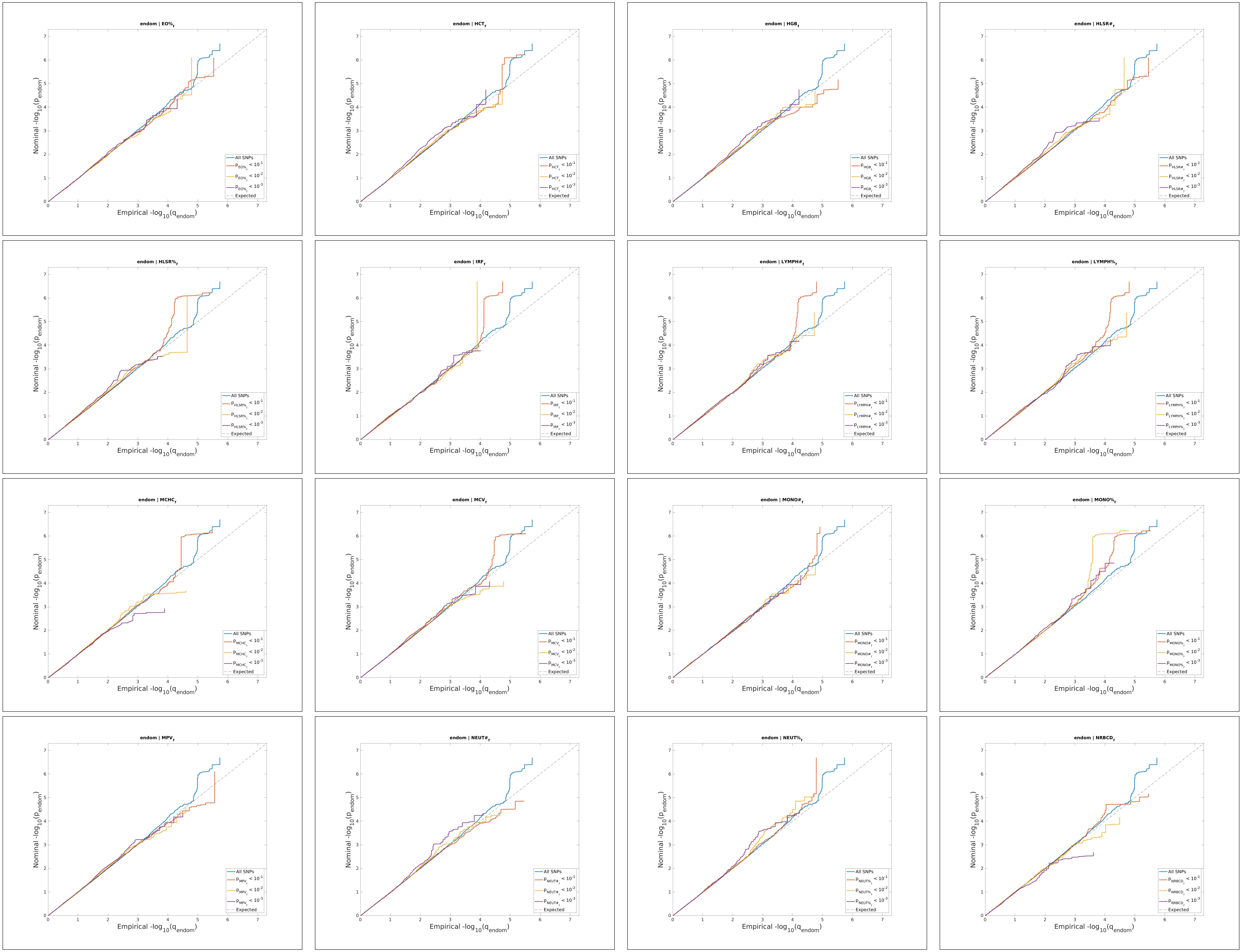
endom | EO%f
endom | HCTf
endom | HGBf
endom | HLSR#f
endom | HLSR%f
endom | IRFf
endom | LYMPH#f
endom | LYMPH%f
endom | MCHCf
endom | MCVf
endom | MONO#f
endom | MONO%f
endom | MPVf
endom | NEUT#f
endom | NEUT%f
endom | NRBCDf
Nominal -log10(pendom)
Empirical -log10(qendom)
All SNPs
< 10-1
< 10-2
< 10-3
Expected

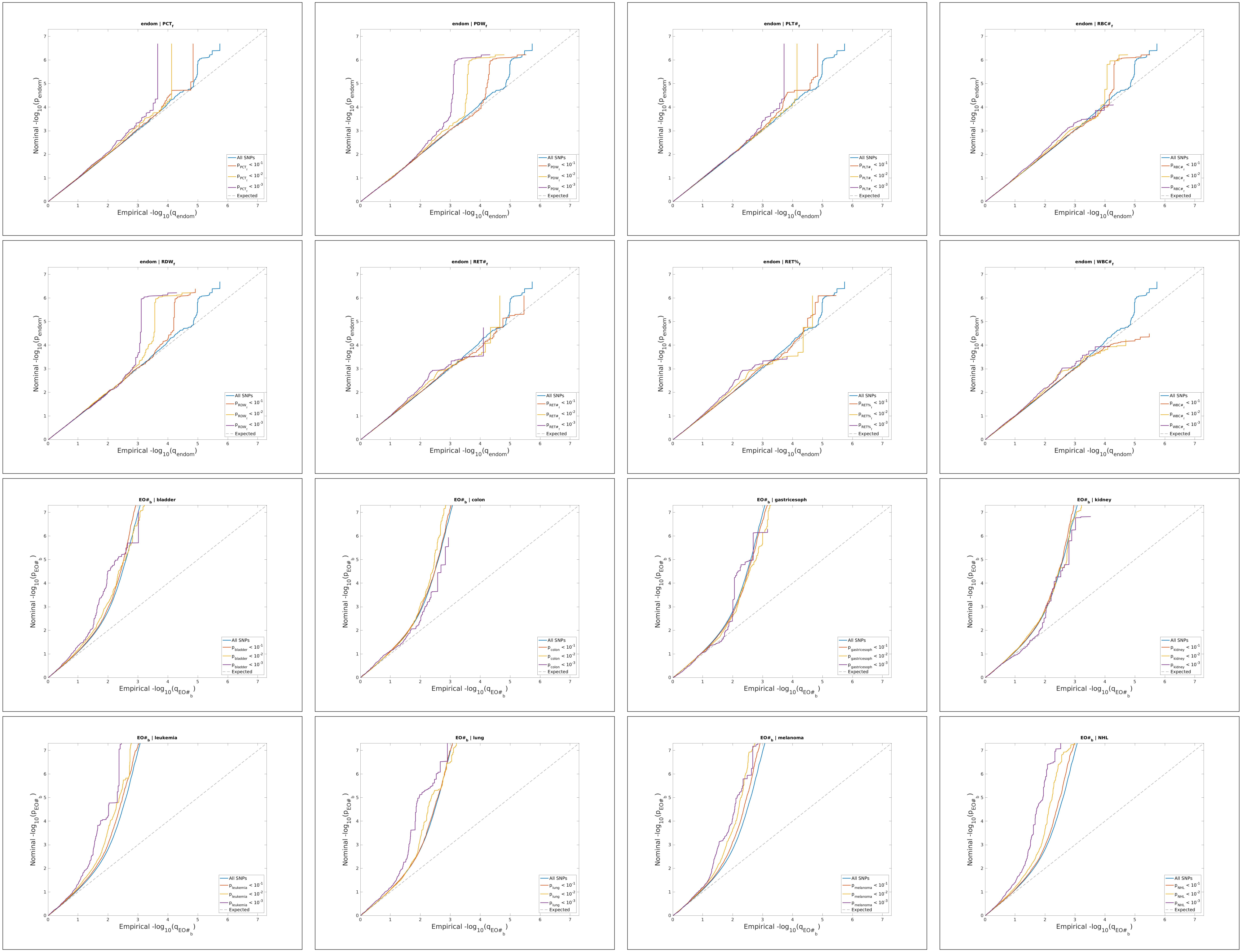
endom | PCT_f
endom | PDW_f
endom | $PLT\#_f$
endom | $RBC\#_f$
endom | RDW_f
endom | $RET\#_f$
endom | $RET\%_f$
endom | $WBC\#_f$
$EO\#_b$ | bladder
$EO\#_b$ | colon
$EO\#_b$ | gastricesoph
$EO\#_b$ | kidney
$EO\#_b$ | leukemia
$EO\#_b$ | lung
$EO\#_b$ | melanoma
$EO\#_b$ | NHL
Nominal $-\log_{10}(p_{endom})$
Empirical $-\log_{10}(q_{endom})$
Nominal $-\log_{10}(p_{EO\#_b})$
Empirical $-\log_{10}(q_{EO\#_b})$
All SNPs
$p < 10^{-1}$
$p < 10^{-2}$
$p < 10^{-3}$
Expected

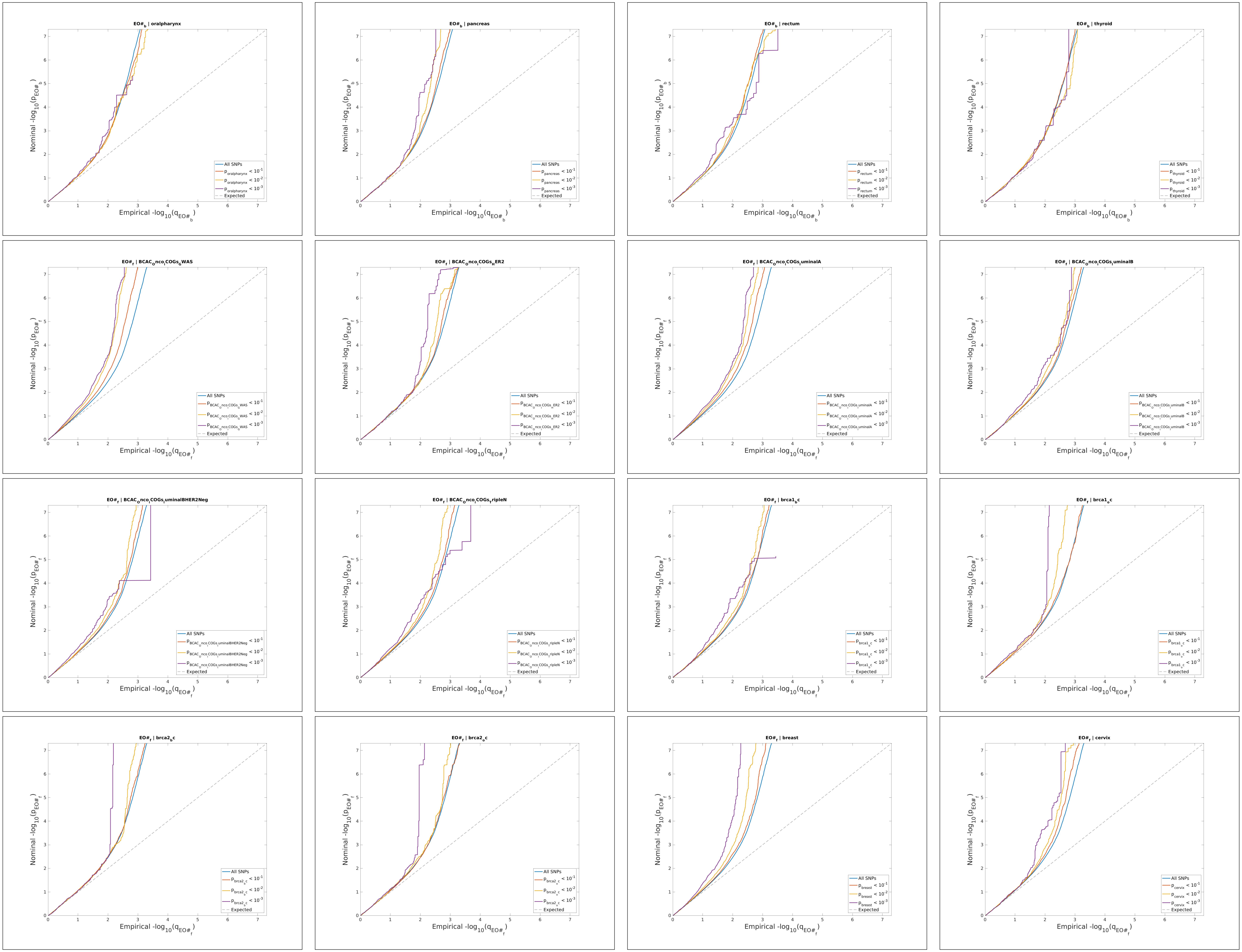

EO#b | oralpharynx
EO#b | pancreas
EO#b | rectum
EO#b | thyroid
EO#f | BCAC_Onco_iCOGs_GWAS
EO#f | BCAC_Onco_iCOGs_HER2
EO#f | BCAC_Onco_iCOGs_LuminalA
EO#f | BCAC_Onco_iCOGs_LuminalB
EO#f | BCAC_Onco_iCOGs_LuminalBHER2Neg
EO#f | BCAC_Onco_iCOGs_TripleN
EO#f | brca1_bc
EO#f | brca1_oc
EO#f | brca2_bc
EO#f | brca2_oc
EO#f | breast
EO#f | cervix
All SNPs
Expected

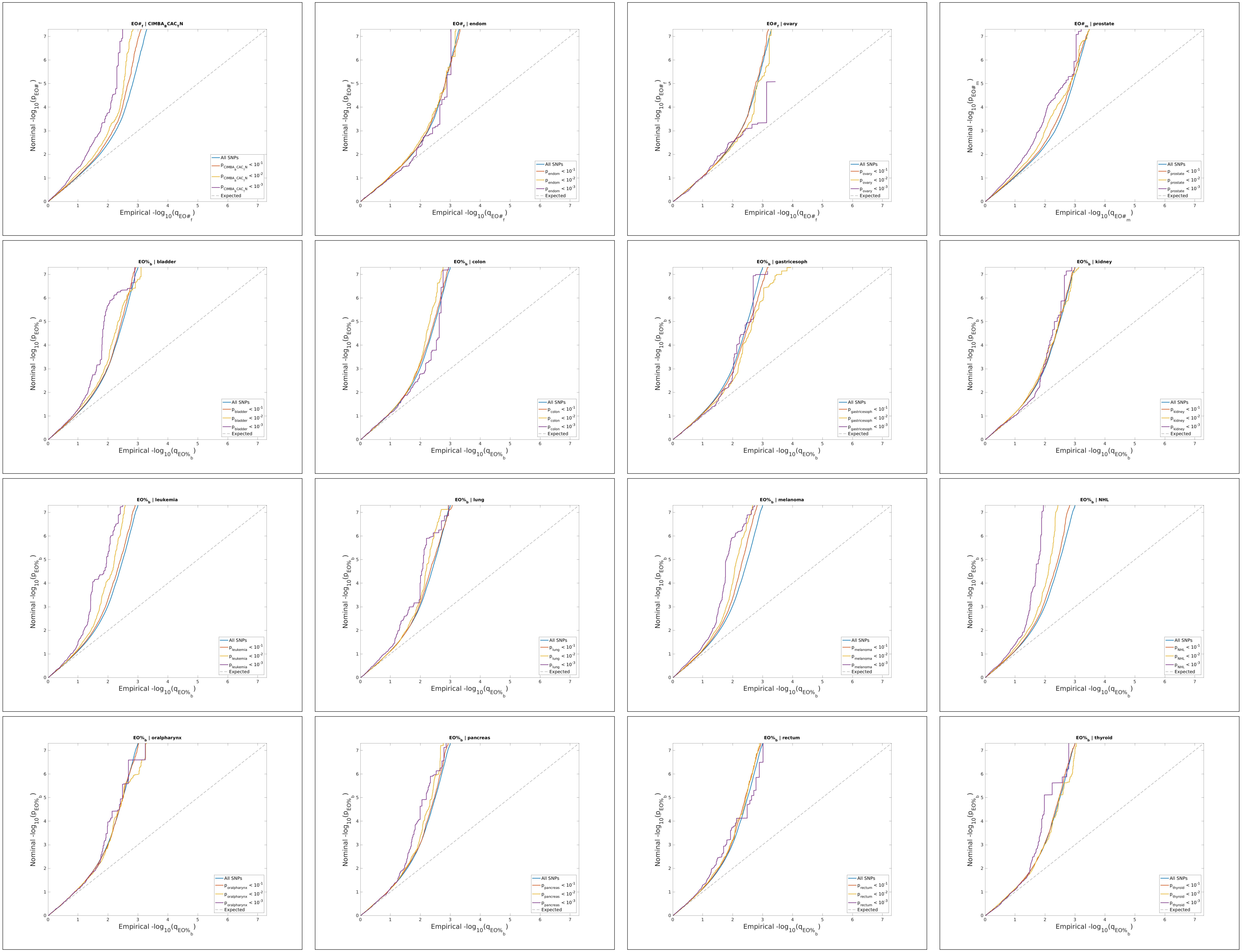
EO#f | CIMBABCACTN
EO#f | endom
EO#f | ovary
EO#m | prostate
EO%b | bladder
EO%b | colon
EO%b | gastricesoph
EO%b | kidney
EO%b | leukemia
EO%b | lung
EO%b | melanoma
EO%b | NHL
EO%b | oralpharynx
EO%b | pancreas
EO%b | rectum
EO%b | thyroid
Nominal -log10(p)
Empirical -log10(q)
All SNPs
Expected

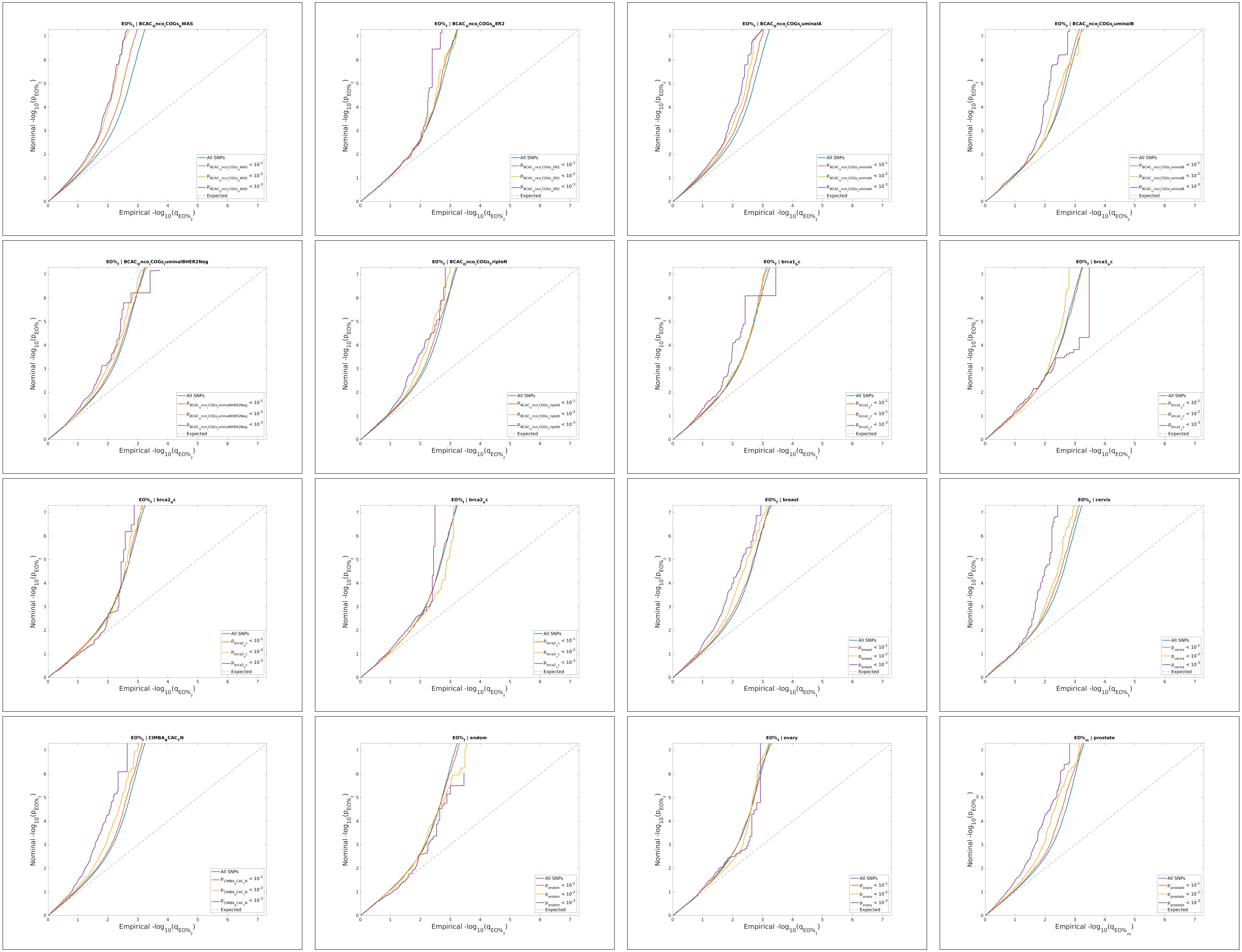
EO%f | BCACOncoiCOGsGWAS
EO%f | BCACOncoiCOGsHER2
EO%f | BCACOncoiCOGsLuminalA
EO%f | BCACOncoiCOGsLuminalB
EO%f | BCACOncoiCOGsLuminalBHER2Neg
EO%f | BCACOncoiCOGsTripleN
EO%f | brca1bc
EO%f | brca1oc
EO%f | brca2bc
EO%f | brca2oc
EO%f | breast
EO%f | cervix
EO%f | CIMBABCACTN
EO%f | endom
EO%f | ovary
EO%m | prostate
All SNPs
Expected
Nominal -log10(pEO%f)
Empirical -log10(qEO%f)
Nominal -log10(pEO%m)
Empirical -log10(qEO%m)

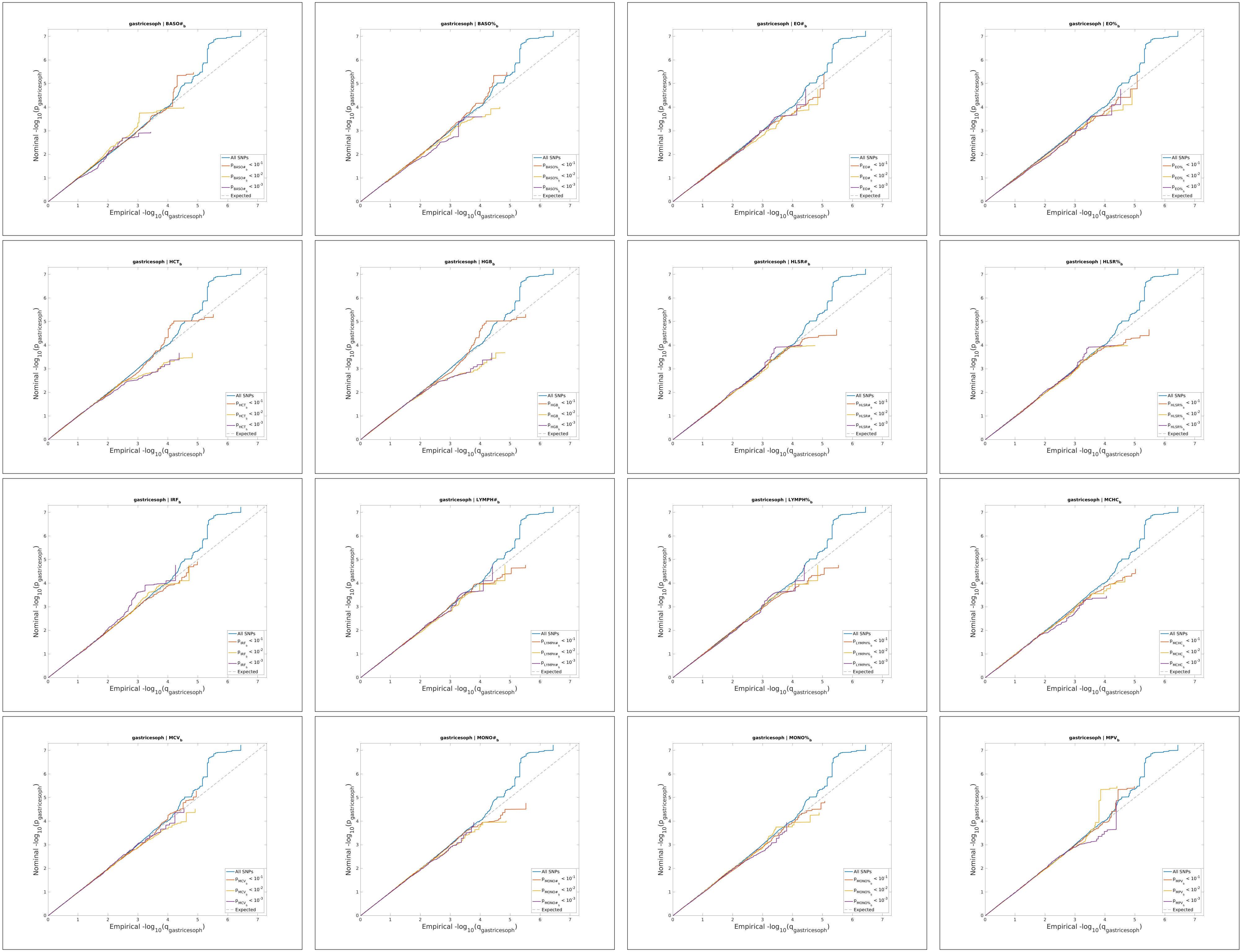

gastricesoph | BASO#b
gastricesoph | BASO%b
gastricesoph | EO#b
gastricesoph | EO%b
gastricesoph | HCTb
gastricesoph | HGBb
gastricesoph | HLSR#b
gastricesoph | HLSR%b
gastricesoph | IRFb
gastricesoph | LYMPH#b
gastricesoph | LYMPH%b
gastricesoph | MCHCb
gastricesoph | MCVb
gastricesoph | MONO#b
gastricesoph | MONO%b
gastricesoph | MPVb
Nominal -log10(pgastricesoph)
Empirical -log10(qgastricesoph)
All SNPs
Expected

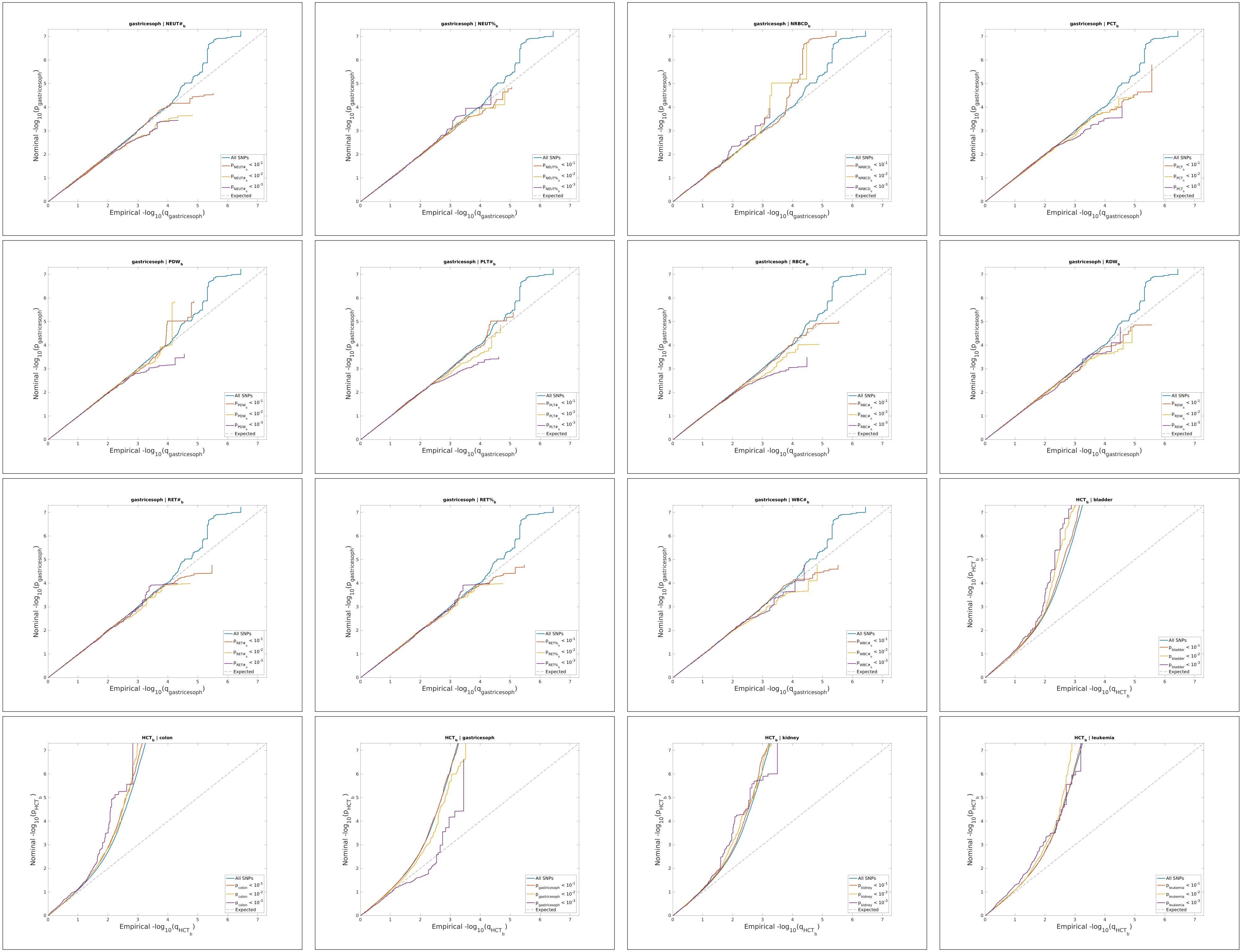
gastricesoph | NEUT#b
gastricesoph | NEUT%b
gastricesoph | NRBCDb
gastricesoph | PCTb
gastricesoph | PDWb
gastricesoph | PLT#b
gastricesoph | RBC#b
gastricesoph | RDWb
gastricesoph | RET#b
gastricesoph | RET%b
gastricesoph | WBC#b
HCTb | bladder
HCTb | colon
HCTb | gastricesoph
HCTb | kidney
HCTb | leukemia
Nominal -log10(pgastricesoph)
Empirical -log10(qgastricesoph)
Nominal -log10(pHCTb)
Empirical -log10(qHCTb)
All SNPs
Expected

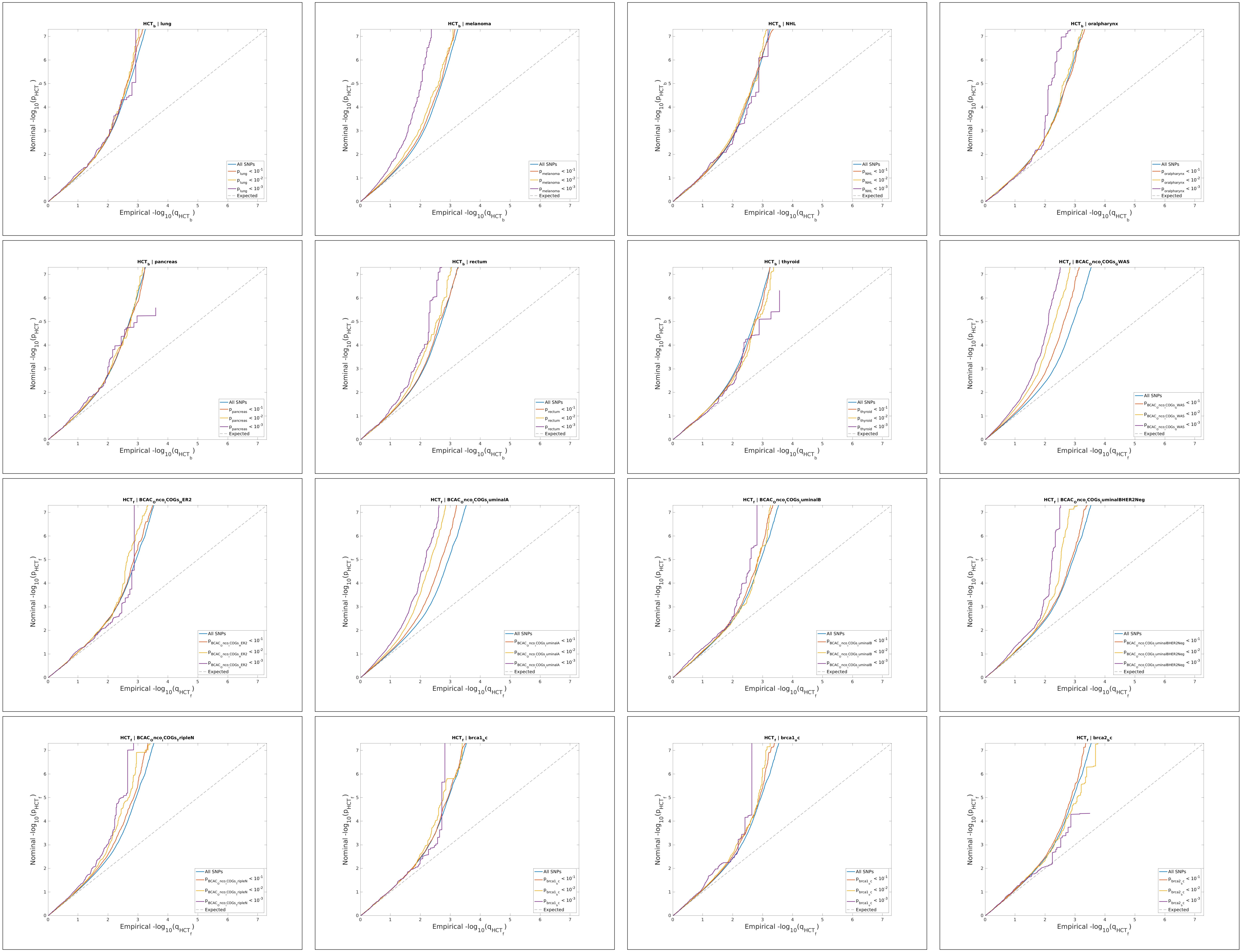
HCTb | lung
HCTb | melanoma
HCTb | NHL
HCTb | oralpharynx
HCTb | pancreas
HCTb | rectum
HCTb | thyroid
HCTf | BCAConcoCOGsGWAS
HCTf | BCAConcoCOGsHER2
HCTf | BCAConcoCOGsluminalA
HCTf | BCAConcoCOGsluminalB
HCTf | BCAConcoCOGsluminalBHER2Neg
HCTf | BCAConcoCOGstripleN
HCTf | brca1bc
HCTf | brca1oc
HCTf | brca2bc
Nominal -log10(pHCT)
Empirical -log10(qHCT)
All SNPs
Expected

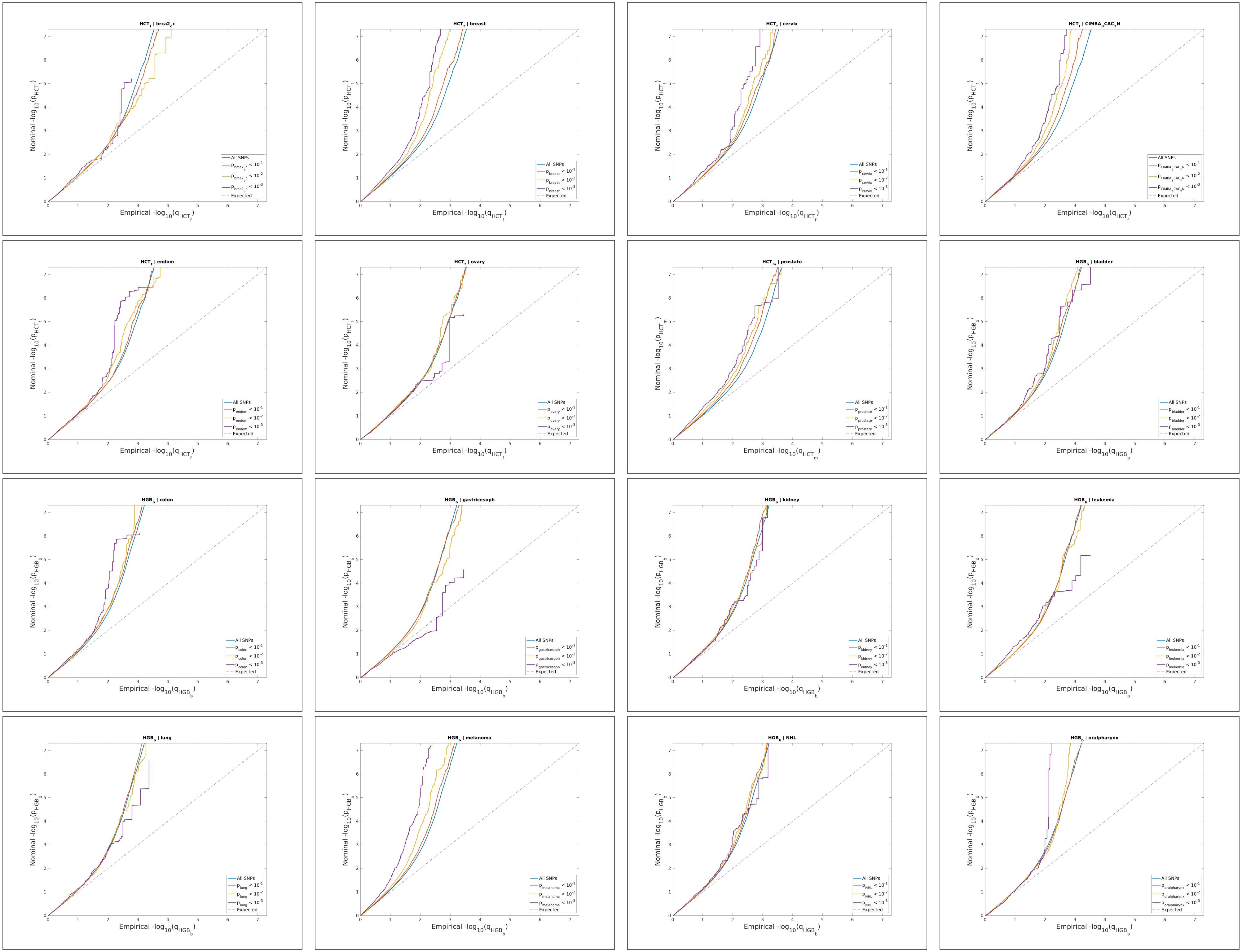
HCTf | breast
HCTf | cervix
HCTf | endom
HCTf | ovary
HCTm | prostate
HGBb | bladder
HGBb | colon
HGBb | gastricesoph
HGBb | kidney
HGBb | leukemia
HGBb | lung
HGBb | melanoma
HGBb | NHL
HGBb | oralpharynx
Nominal -log10(p)
Empirical -log10(q)
All SNPs
Expected

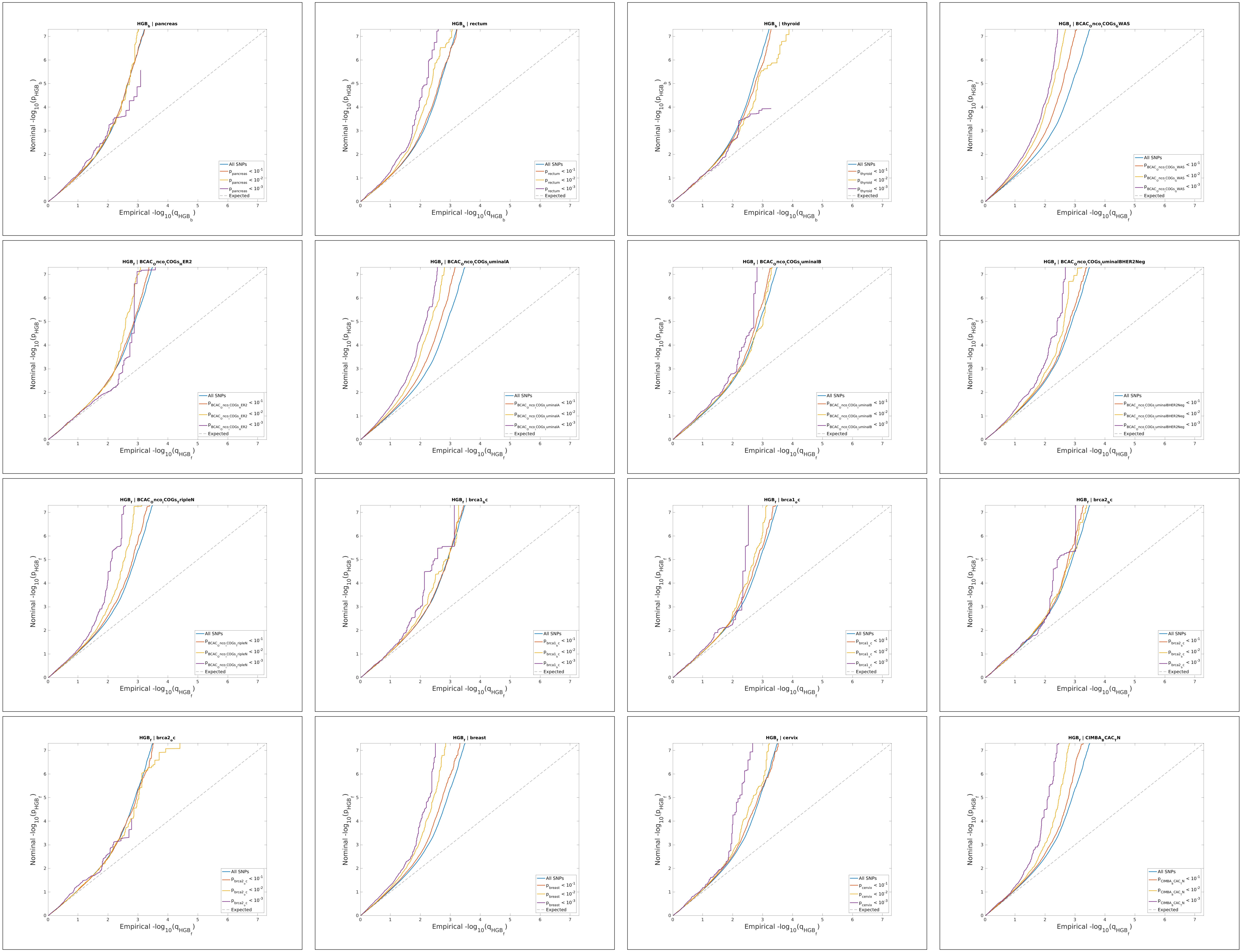
HGBb | pancreas
HGBb | rectum
HGBb | thyroid
HGBf | BCAConcoCOGsGWAS
HGBf | BCAConcoCOGsHER2
HGBf | BCAConcoCOGsluminalA
HGBf | BCAConcoCOGsluminalB
HGBf | BCAConcoCOGsluminalBHER2Neg
HGBf | BCAConcoCOGstripleN
HGBf | brca1bc
HGBf | brca1oc
HGBf | brca2bc
HGBf | brca2oc
HGBf | breast
HGBf | cervix
HGBf | CIMBABCACtN
Nominal -log10(pHGB)
Empirical -log10(qHGB)
All SNPs
Expected

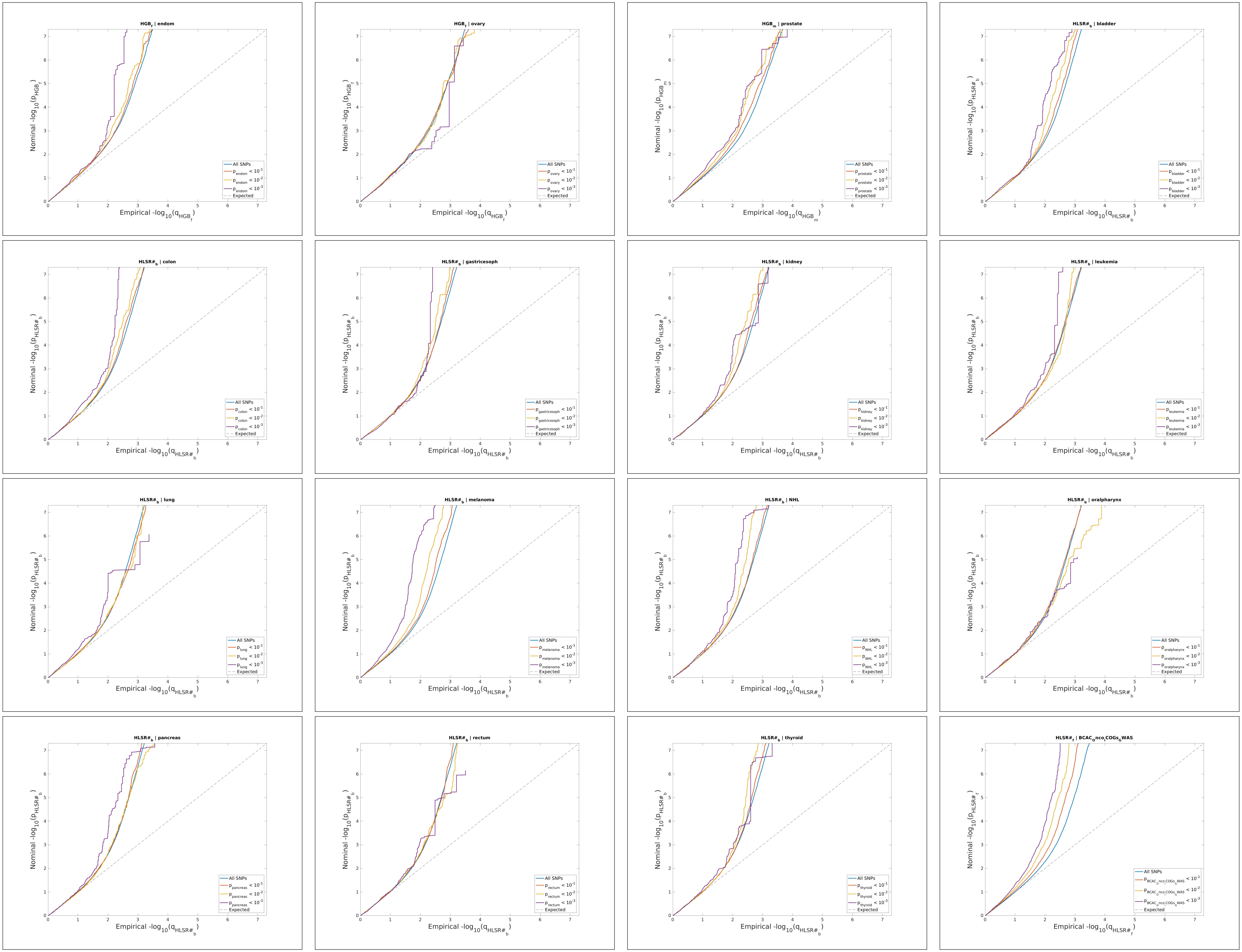

HGBf | endom
HGBf | ovary
HGBm | prostate
HLSR#b | bladder
HLSR#b | colon
HLSR#b | gastricesoph
HLSR#b | kidney
HLSR#b | leukemia
HLSR#b | lung
HLSR#b | melanoma
HLSR#b | NHL
HLSR#b | oralpharynx
HLSR#b | pancreas
HLSR#b | rectum
HLSR#b | thyroid
Nominal -log10(pHGBf)
Empirical -log10(qHGBf)
Nominal -log10(pHGBm)
Empirical -log10(qHGBm)
Nominal -log10(pHLSR#b)
Empirical -log10(qHLSR#b)
Nominal -log10(pHLSR#f)
Empirical -log10(qHLSR#f)
All SNPs
Expected

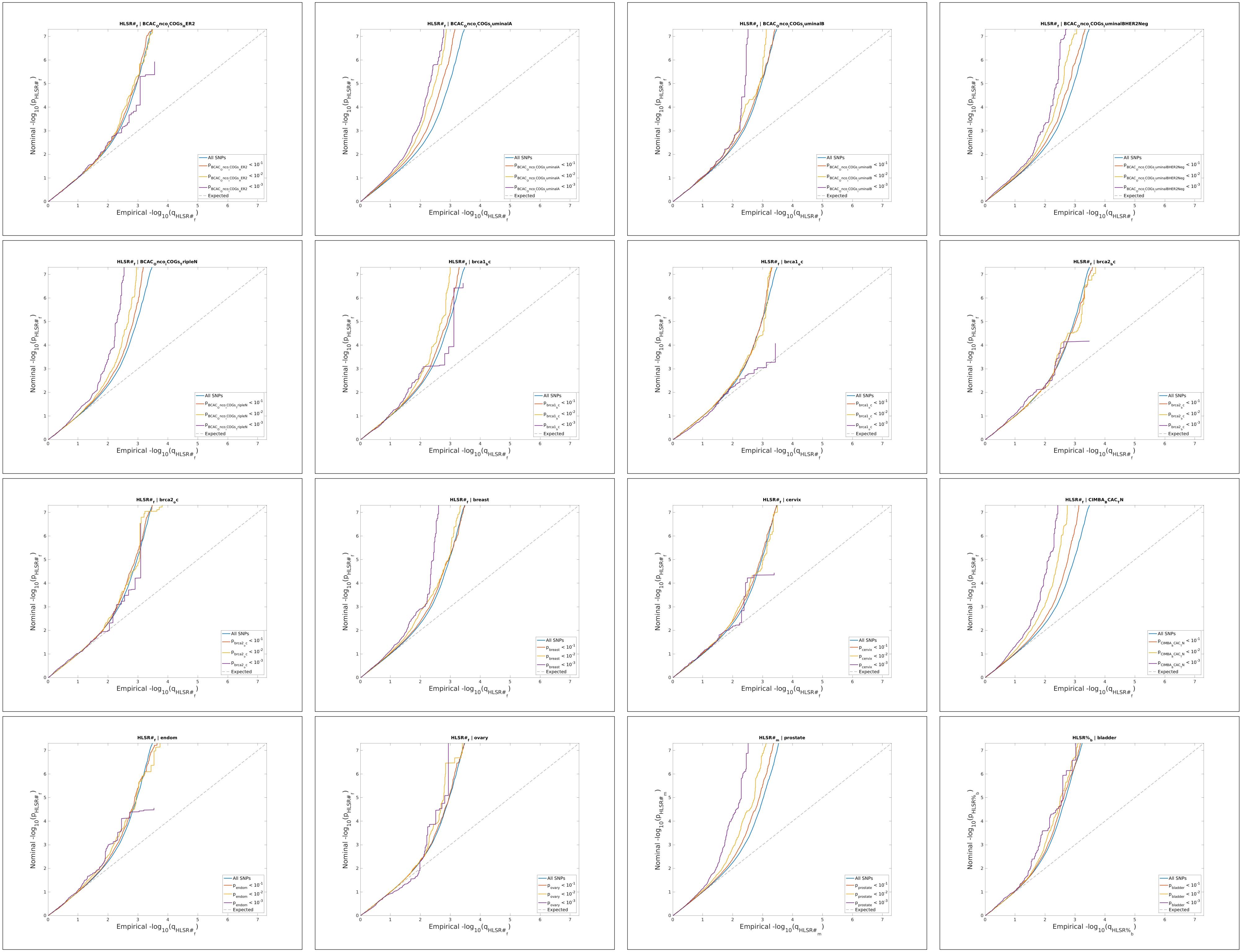

HLSR#f | BCAConcoCOGsHER2
HLSR#f | BCAConcoCOGsuminalA
HLSR#f | BCAConcoCOGsuminalB
HLSR#f | BCAConcoCOGsuminalBHER2Neg
HLSR#f | BCAConcoCOGsripleN
HLSR#f | brca1bc
HLSR#f | brca1oc
HLSR#f | brca2bc
HLSR#f | brca2oc
HLSR#f | breast
HLSR#f | cervix
HLSR#f | CIMBABCACSN
HLSR#f | endom
HLSR#f | ovary
HLSR#m | prostate
HLSR%b | bladder
Nominal -log10(pHLSR#f)
Empirical -log10(qHLSR#f)
All SNPs
Expected

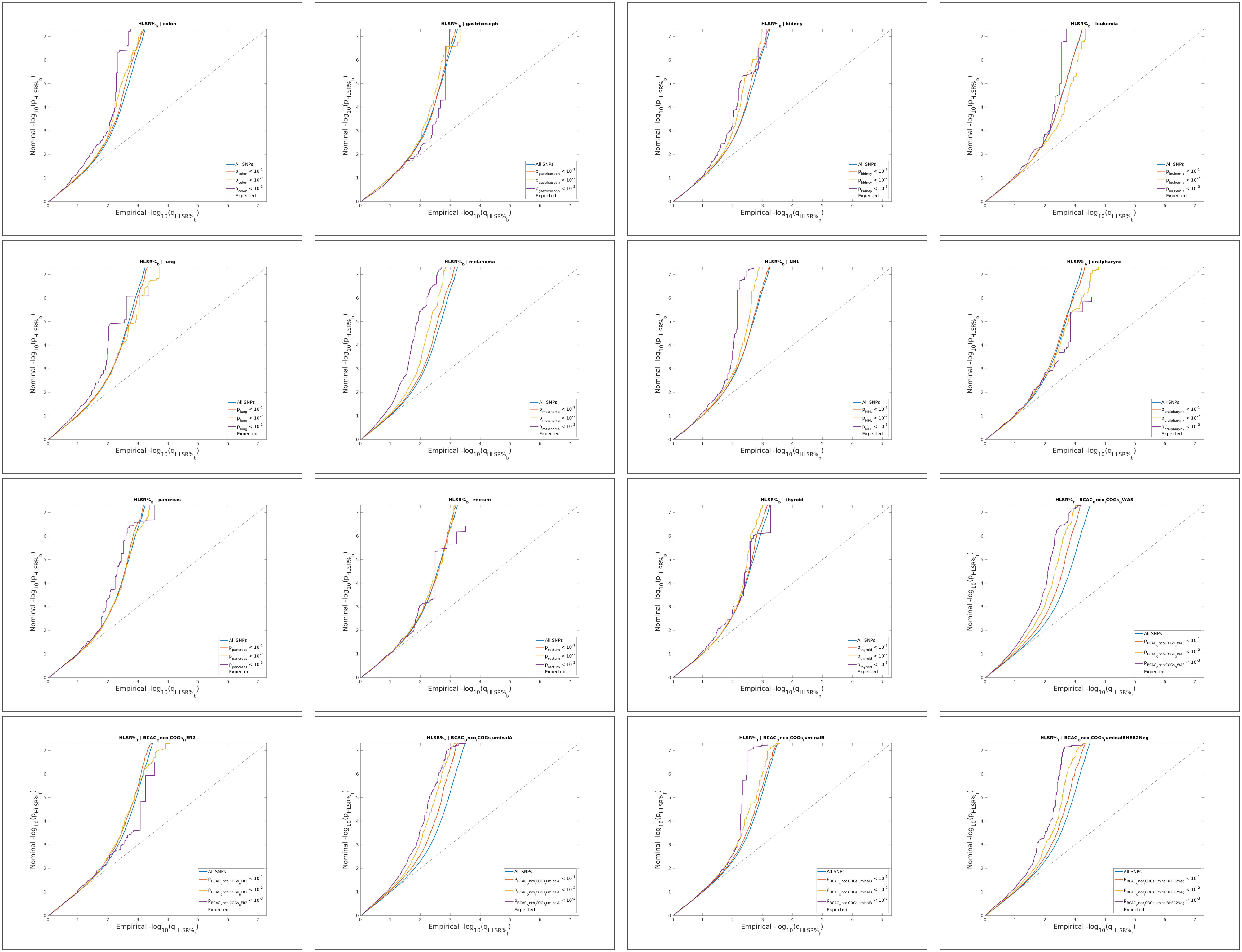

HLSR%b | colon
HLSR%b | gastricesoph
HLSR%b | kidney
HLSR%b | leukemia
HLSR%b | lung
HLSR%b | melanoma
HLSR%b | NHL
HLSR%b | oralpharynx
HLSR%b | pancreas
HLSR%b | rectum
HLSR%b | thyroid
HLSR%f | BCACOncoCOGsGWAS
HLSR%f | BCACOncoCOGsHER2
HLSR%f | BCACOncoCOGsLuminalA
HLSR%f | BCACOncoCOGsLuminalB
HLSR%f | BCACOncoCOGsLuminalBHER2Neg
Nominal -log10(p)
Empirical -log10(q)
All SNPs
Expected

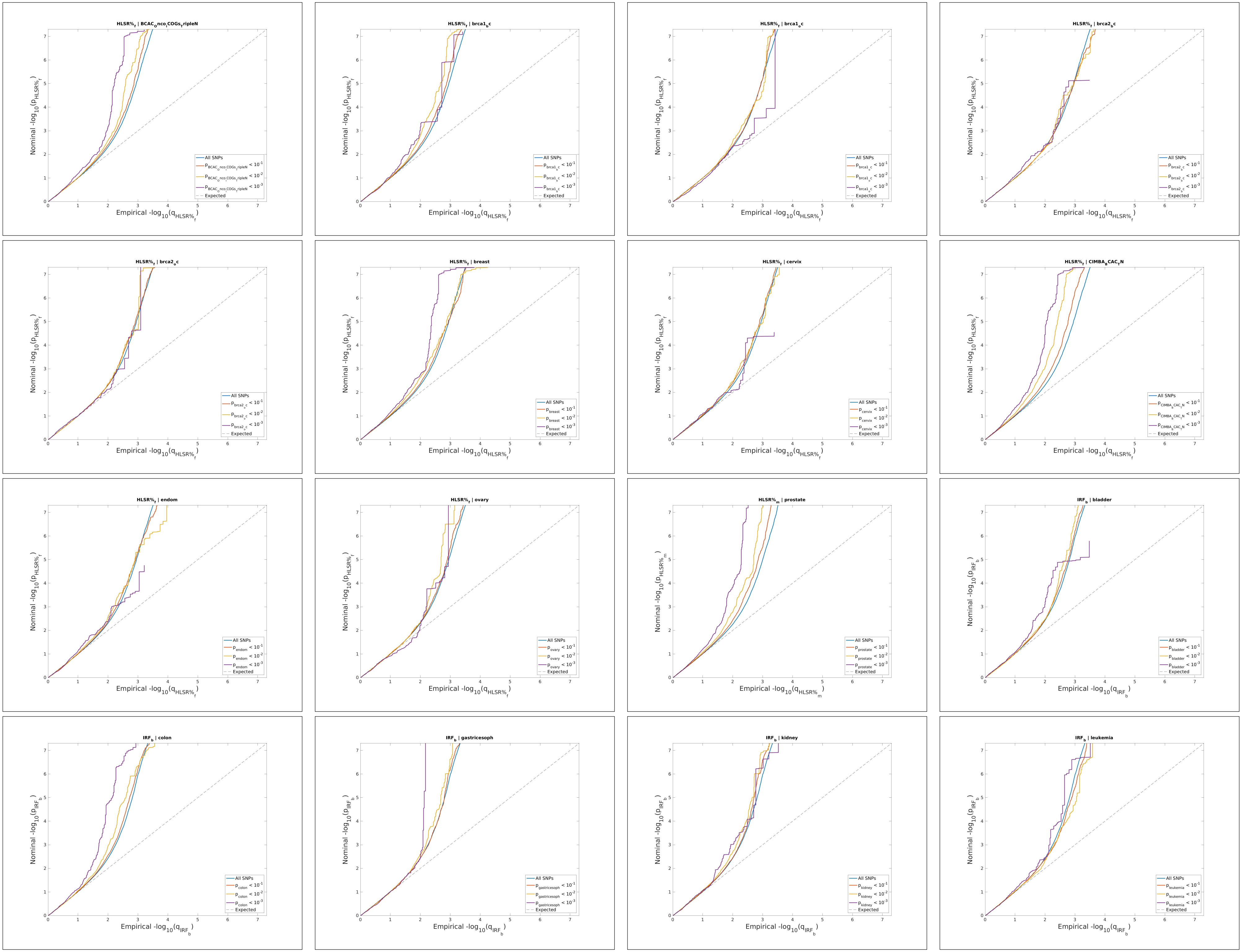

HLSR%f | BCACOncoICOGsTripleN
HLSR%f | brca1bc
HLSR%f | brca1oc
HLSR%f | brca2bc
HLSR%f | brca2oc
HLSR%f | breast
HLSR%f | cervix
HLSR%f | CIMBABCACTN
HLSR%f | endom
HLSR%f | ovary
HLSR%m | prostate
IRFb | bladder
IRFb | colon
IRFb | gastricesoph
IRFb | kidney
IRFb | leukemia
Nominal -log10(p)
Empirical -log10(q)
All SNPs
Expected

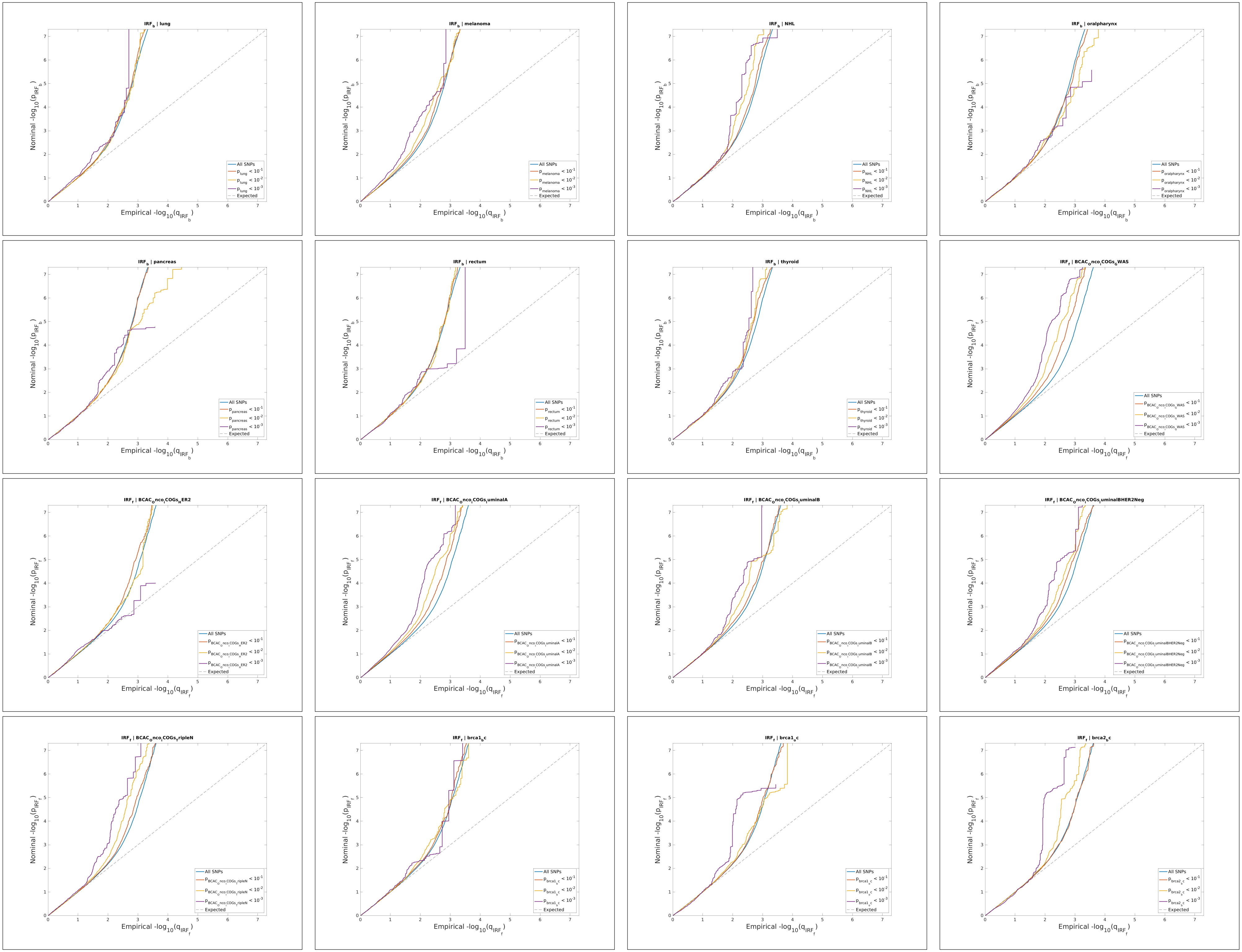
IRFb | lung
IRFb | melanoma
IRFb | NHL
IRFb | oralpharynx
IRFb | pancreas
IRFb | rectum
IRFb | thyroid
IRFf | BCAConcoCOGsGWAS
IRFf | BCAConcoCOGsHER2
IRFf | BCAConcoCOGsuminalA
IRFf | BCAConcoCOGsuminalB
IRFf | BCAConcoCOGsuminalBHER2Neg
IRFf | BCAConcoCOGsripleN
IRFf | brca1bc
IRFf | brca1oc
IRFf | brca2bc
Nominal -log10(pIRF)
Empirical -log10(qIRF)
All SNPs
Expected

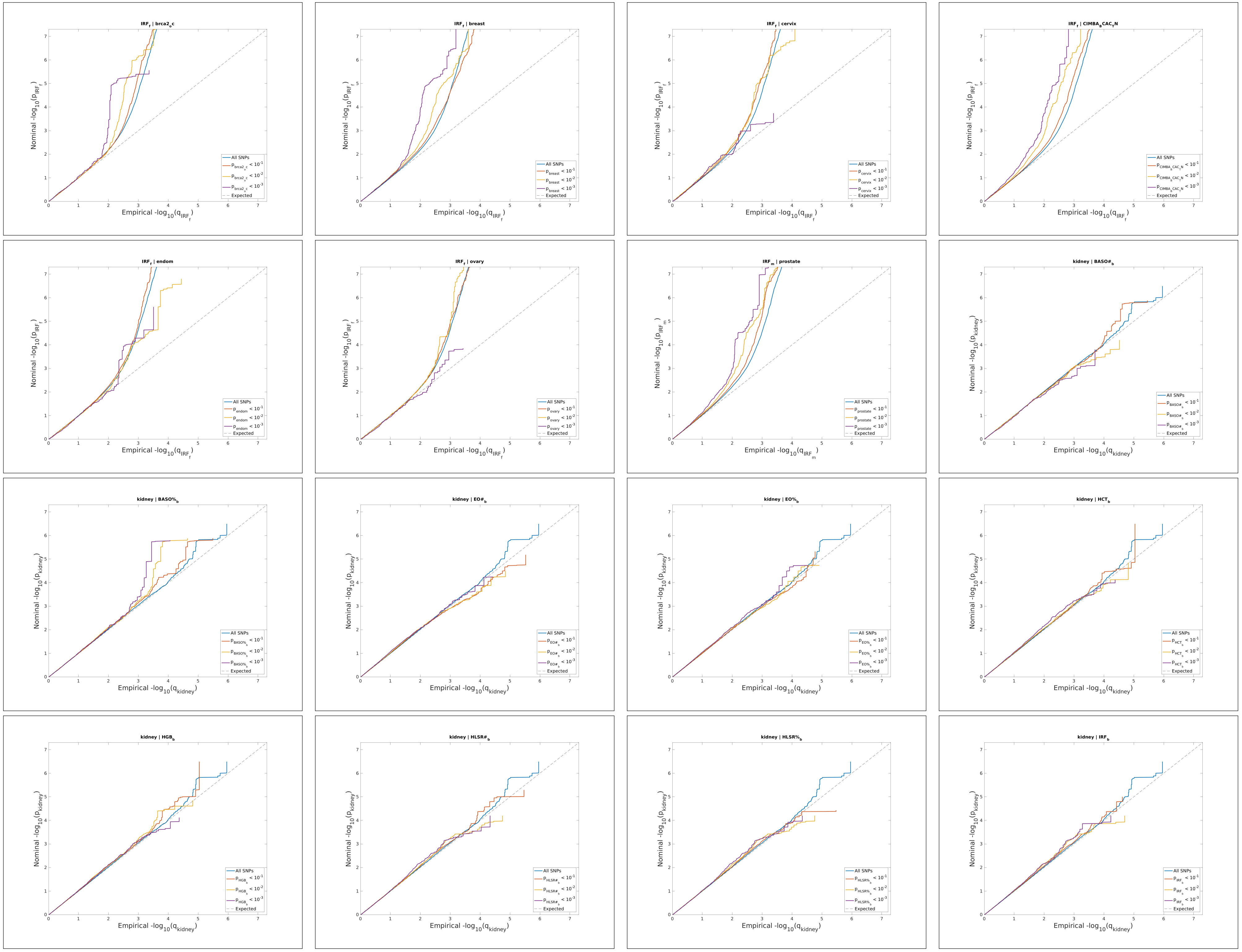
IRF_f | brca2_c
IRF_f | breast
IRF_f | cervix
IRF_f | CIMBA_BCAC_TN
IRF_f | endom
IRF_f | ovary
IRF_m | prostate
kidney | BASO#_b
kidney | BASO%_b
kidney | EO#_b
kidney | EO%_b
kidney | HCT_b
kidney | HGB_b
kidney | HLSR#_b
kidney | HLSR%_b
kidney | IRF_b
Nominal -log10(p)
Empirical -log10(q)

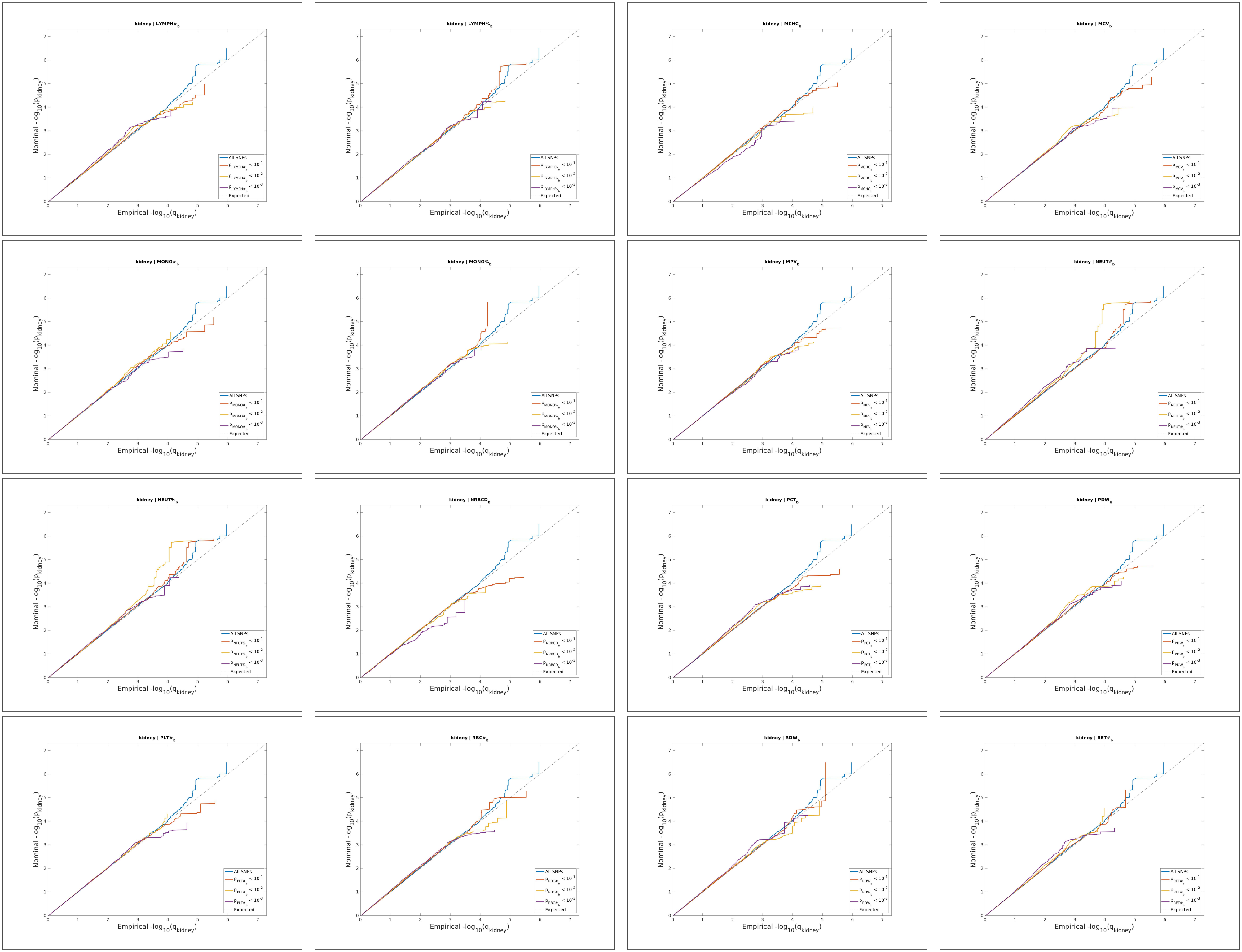

kidney | LYMPH#b
kidney | LYMPH%b
kidney | MCHCb
kidney | MCVb
kidney | MONO#b
kidney | MONO%b
kidney | MPVb
kidney | NEUT#b
kidney | NEUT%b
kidney | NRBCDb
kidney | PCTb
kidney | PDWb
kidney | PLT#b
kidney | RBC#b
kidney | RDWb
kidney | RET#b
Nominal -log10(pkidney)
Empirical -log10(qkidney)
All SNPs
Expected

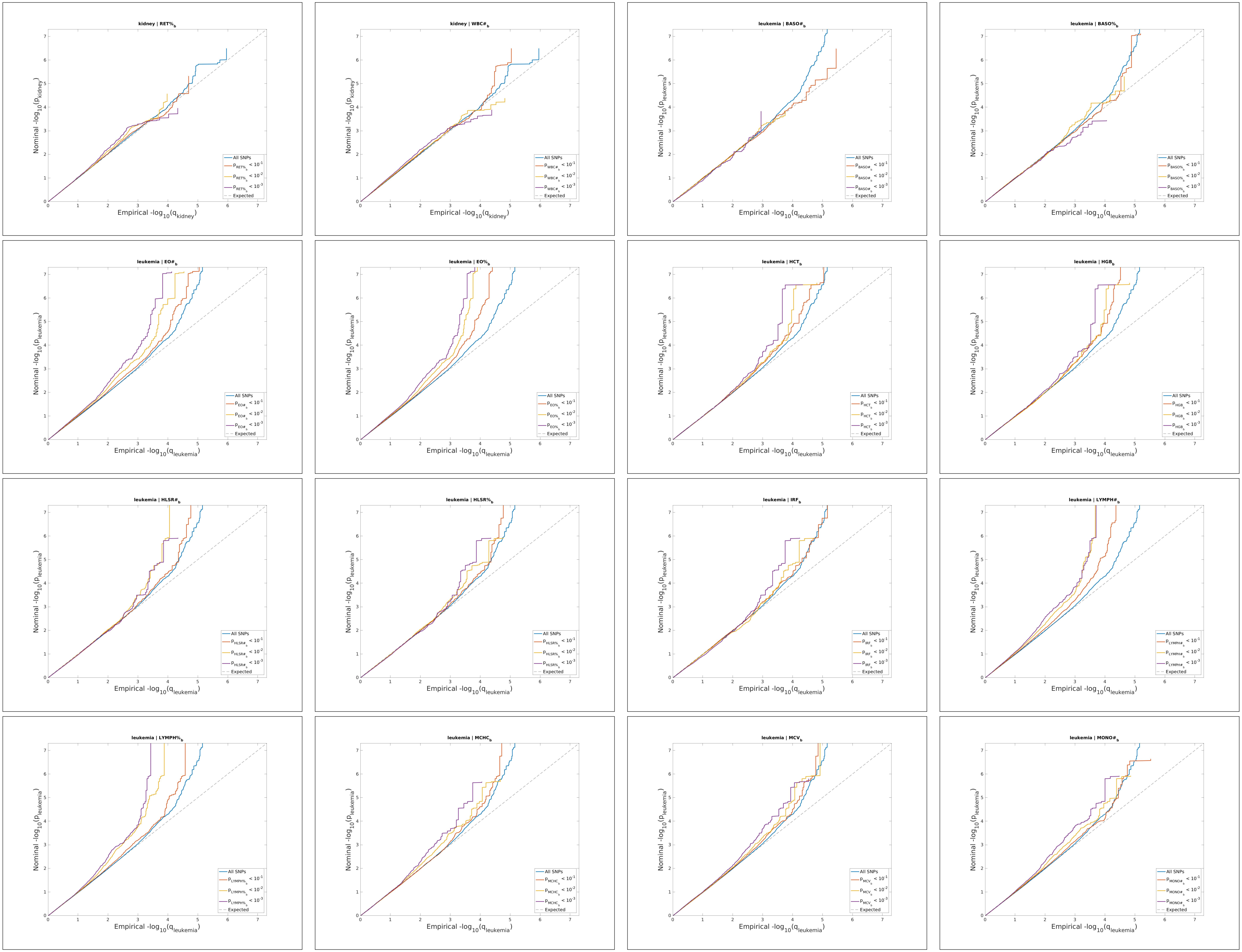

kidney | RET%b
Nominal -log10(pkidney)
Empirical -log10(qkidney)
All SNPs
pRET%b < 10-1
pRET%b < 10-2
pRET%b < 10-3
Expected
kidney | WBC#b
Nominal -log10(pkidney)
Empirical -log10(qkidney)
All SNPs
pWBC#b < 10-1
pWBC#b < 10-2
pWBC#b < 10-3
Expected
leukemia | BASO#b
Nominal -log10(pleukemia)
Empirical -log10(qleukemia)
All SNPs
pBASO#b < 10-1
pBASO#b < 10-2
pBASO#b < 10-3
Expected
leukemia | BASO%b
All SNPs
pBASO%b < 10-1
pBASO%b < 10-2
pBASO%b < 10-3
Expected
leukemia | EO#b
All SNPs
pEO#b < 10-1
pEO#b < 10-2
pEO#b < 10-3
Expected
leukemia | EO%b
All SNPs
pEO%b < 10-1
pEO%b < 10-2
pEO%b < 10-3
Expected
leukemia | HCTb
All SNPs
pHCTb < 10-1
pHCTb < 10-2
pHCTb < 10-3
Expected
leukemia | HGBb
All SNPs
pHGBb < 10-1
pHGBb < 10-2
pHGBb < 10-3
Expected
leukemia | HLSR#b
All SNPs
pHLSR#b < 10-1
pHLSR#b < 10-2
pHLSR#b < 10-3
Expected
leukemia | HLSR%b
All SNPs
pHLSR%b < 10-1
pHLSR%b < 10-2
pHLSR%b < 10-3
Expected
leukemia | IRFb
All SNPs
pIRFb < 10-1
pIRFb < 10-2
pIRFb < 10-3
Expected
leukemia | LYMPH#b
All SNPs
pLYMPH#b < 10-1
pLYMPH#b < 10-2
pLYMPH#b < 10-3
Expected
leukemia | LYMPH%b
All SNPs
pLYMPH%b < 10-1
pLYMPH%b < 10-2
pLYMPH%b < 10-3
Expected
leukemia | MCHCb
All SNPs
pMCHCb < 10-1
pMCHCb < 10-2
pMCHCb < 10-3
Expected
leukemia | MCVb
All SNPs
pMCVb < 10-1
pMCVb < 10-2
pMCVb < 10-3
Expected
leukemia | MONO#b
All SNPs
pMONO#b < 10-1
pMONO#b < 10-2
pMONO#b < 10-3
Expected

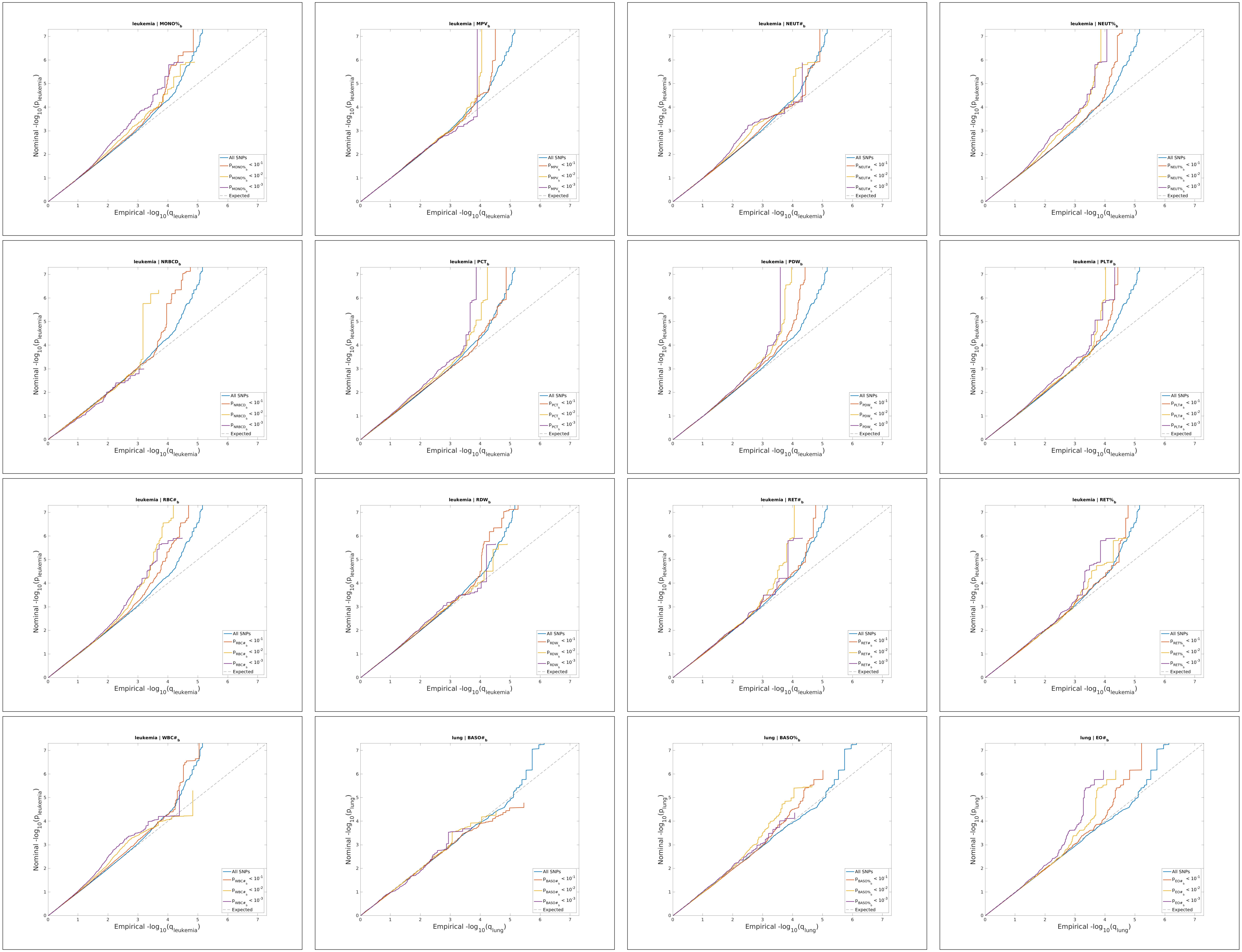

leukemia | MONO%b
leukemia | MPVb
leukemia | NEUT#b
leukemia | NEUT%b
leukemia | NRBCDb
leukemia | PCTb
leukemia | PDWb
leukemia | PLT#b
leukemia | RBC#b
leukemia | RDWb
leukemia | RET#b
leukemia | RET%b
leukemia | WBC#b
lung | BASO#b
lung | BASO%b
lung | EO#b
Nominal -log10(pleukemia)
Empirical -log10(qleukemia)
Nominal -log10(plung)
Empirical -log10(qlung)
All SNPs
< 10-1
< 10-2
< 10-3
Expected

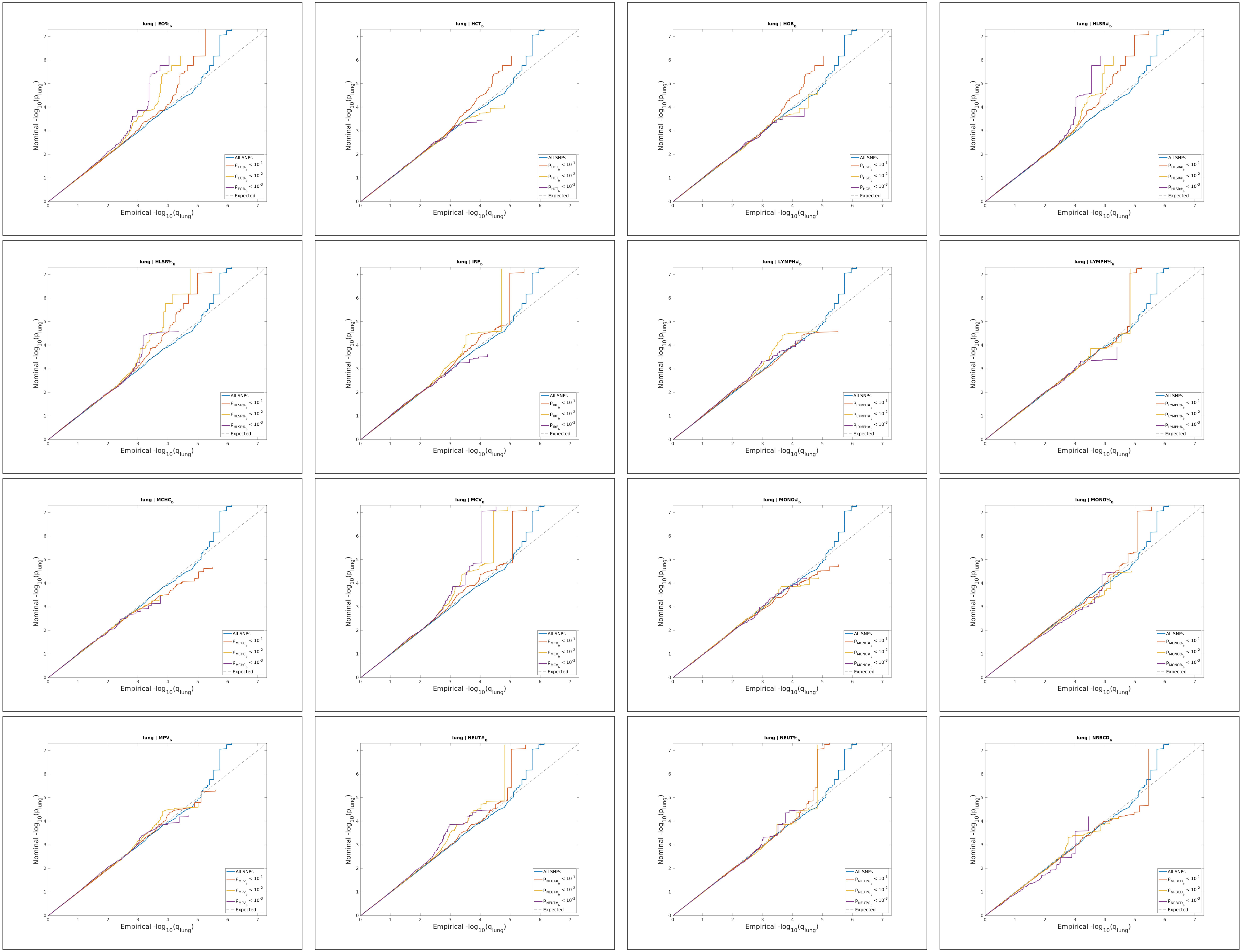

lung | EO%b
lung | HCTb
lung | HGBb
lung | HLSR#b
lung | HLSR%b
lung | IRFb
lung | LYMPH#b
lung | LYMPH%b
lung | MCHCb
lung | MCVb
lung | MONO#b
lung | MONO%b
lung | MPVb
lung | NEUT#b
lung | NEUT%b
lung | NRBCDb
Nominal -log10(plung)
Empirical -log10(qlung)
All SNPs
Expected

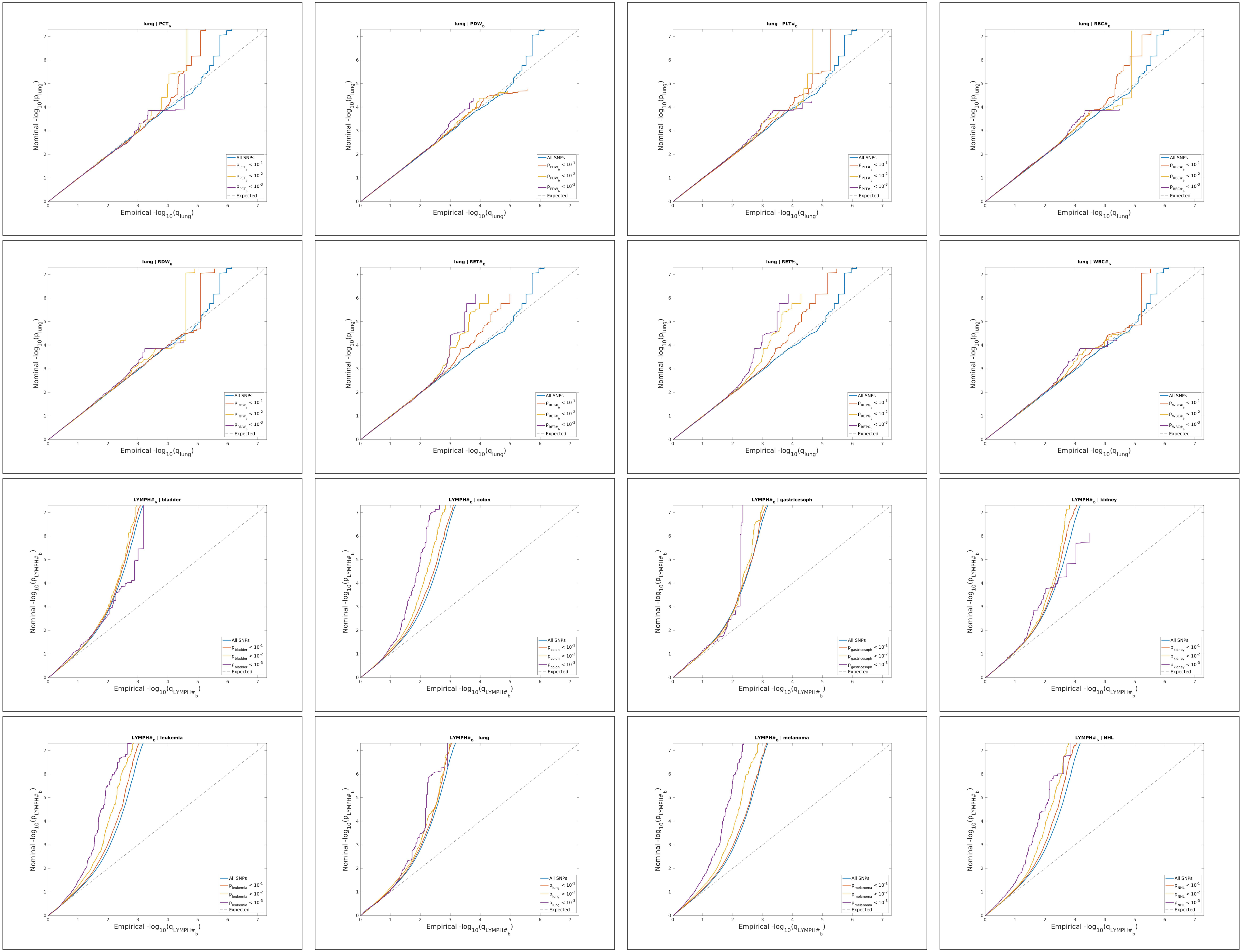

lung | PCT_b
lung | PDW_b
lung | PLT#_b
lung | RBC#_b
lung | RDW_b
lung | RET#_b
lung | RET%_b
lung | WBC#_b
LYMPH#_b | bladder
LYMPH#_b | colon
LYMPH#_b | gastricesoph
LYMPH#_b | kidney
LYMPH#_b | leukemia
LYMPH#_b | lung
LYMPH#_b | melanoma
LYMPH#_b | NHL
Nominal -log10(p_lung)
Empirical -log10(q_lung)
Nominal -log10(p_LYMPH#_b)
Empirical -log10(q_LYMPH#_b)
All SNPs
Expected

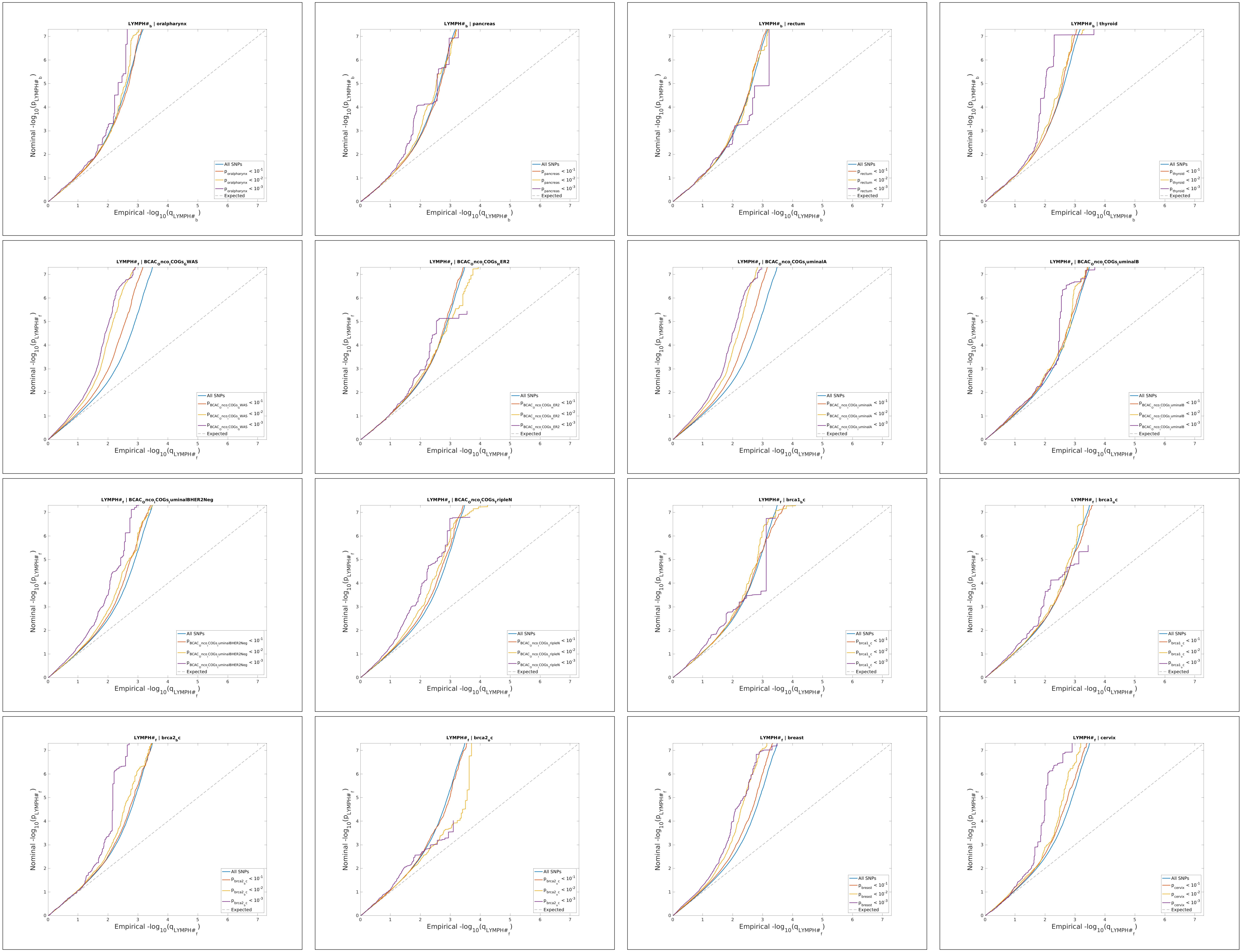
LYMPH#b | oralpharynx
LYMPH#b | pancreas
LYMPH#b | rectum
LYMPH#b | thyroid
LYMPH#f | BCAConcoCOGsGWAS
LYMPH#f | BCAConcoCOGsHER2
LYMPH#f | BCAConcoCOGsuminalA
LYMPH#f | BCAConcoCOGsuminalB
LYMPH#f | BCAConcoCOGsuminalBHER2Neg
LYMPH#f | BCAConcoCOGsripleN
LYMPH#f | brca1bc
LYMPH#f | brca1oc
LYMPH#f | brca2bc
LYMPH#f | brca2oc
LYMPH#f | breast
LYMPH#f | cervix
All SNPs
Expected
Nominal -log10(p)
Empirical -log10(q)

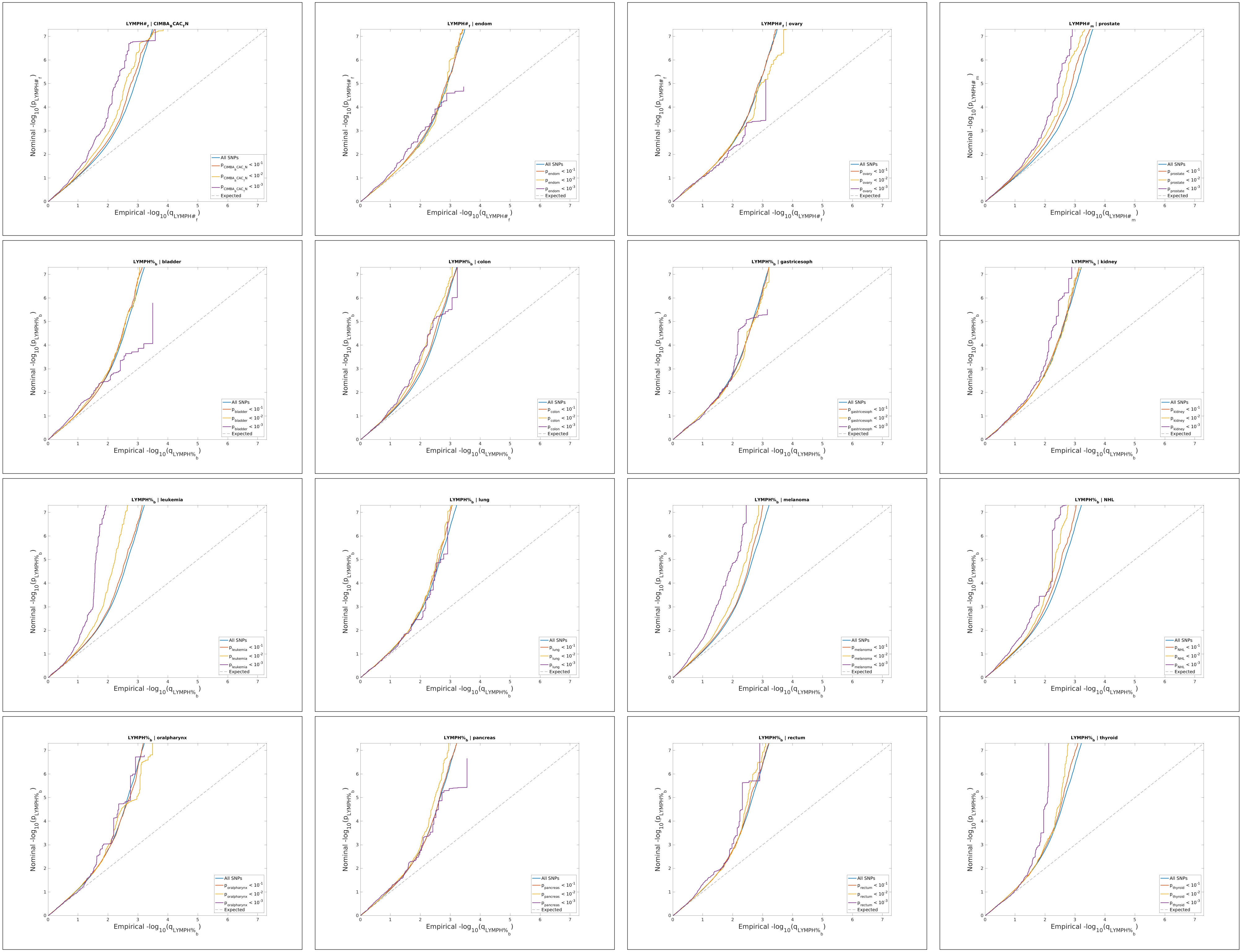

LYMPH#f | CIMBA_B CAC_T N
LYMPH#f | endom
LYMPH#f | ovary
LYMPH#m | prostate
LYMPH%b | bladder
LYMPH%b | colon
LYMPH%b | gastricesoph
LYMPH%b | kidney
LYMPH%b | leukemia
LYMPH%b | lung
LYMPH%b | melanoma
LYMPH%b | NHL
LYMPH%b | oralpharynx
LYMPH%b | pancreas
LYMPH%b | rectum
LYMPH%b | thyroid
Nominal -log10(p)
Empirical -log10(q)
All SNPs
p < 10-1
p < 10-2
p < 10-3
Expected

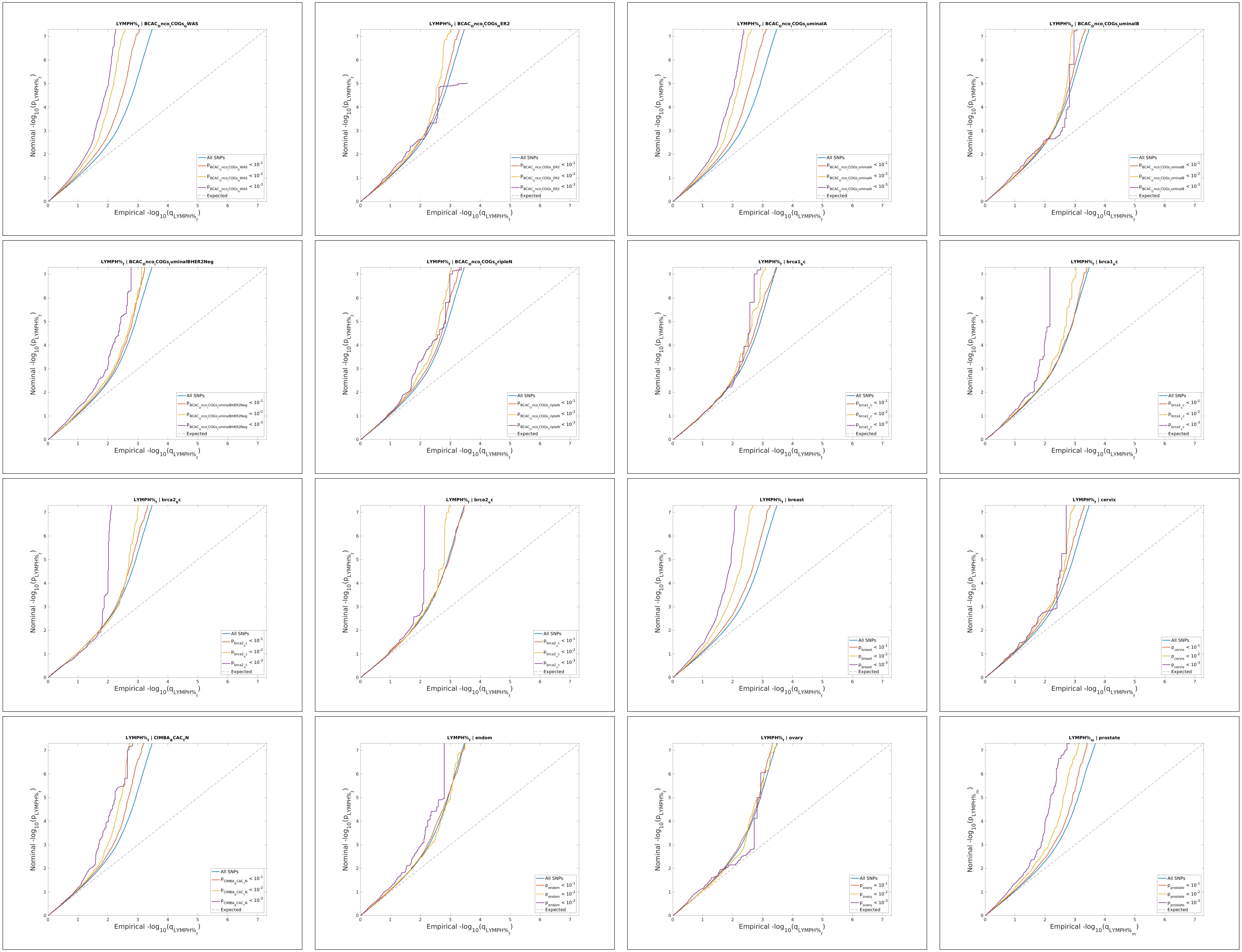
LYMPH%f | BCAConcoCOGsGWAS
LYMPH%f | BCAConcoCOGsHER2
LYMPH%f | BCAConcoCOGsLuminalA
LYMPH%f | BCAConcoCOGsLuminalB
LYMPH%f | BCAConcoCOGsLuminalBHER2Neg
LYMPH%f | BCAConcoCOGsTripleN
LYMPH%f | brca1bc
LYMPH%f | brca1oc
LYMPH%f | brca2bc
LYMPH%f | brca2oc
LYMPH%f | breast
LYMPH%f | cervix
LYMPH%f | CIMBABCACTN
LYMPH%f | endom
LYMPH%f | ovary
LYMPH%m | prostate
Nominal -log10(pLYMPH%f)
Empirical -log10(qLYMPH%f)
All SNPs
Expected

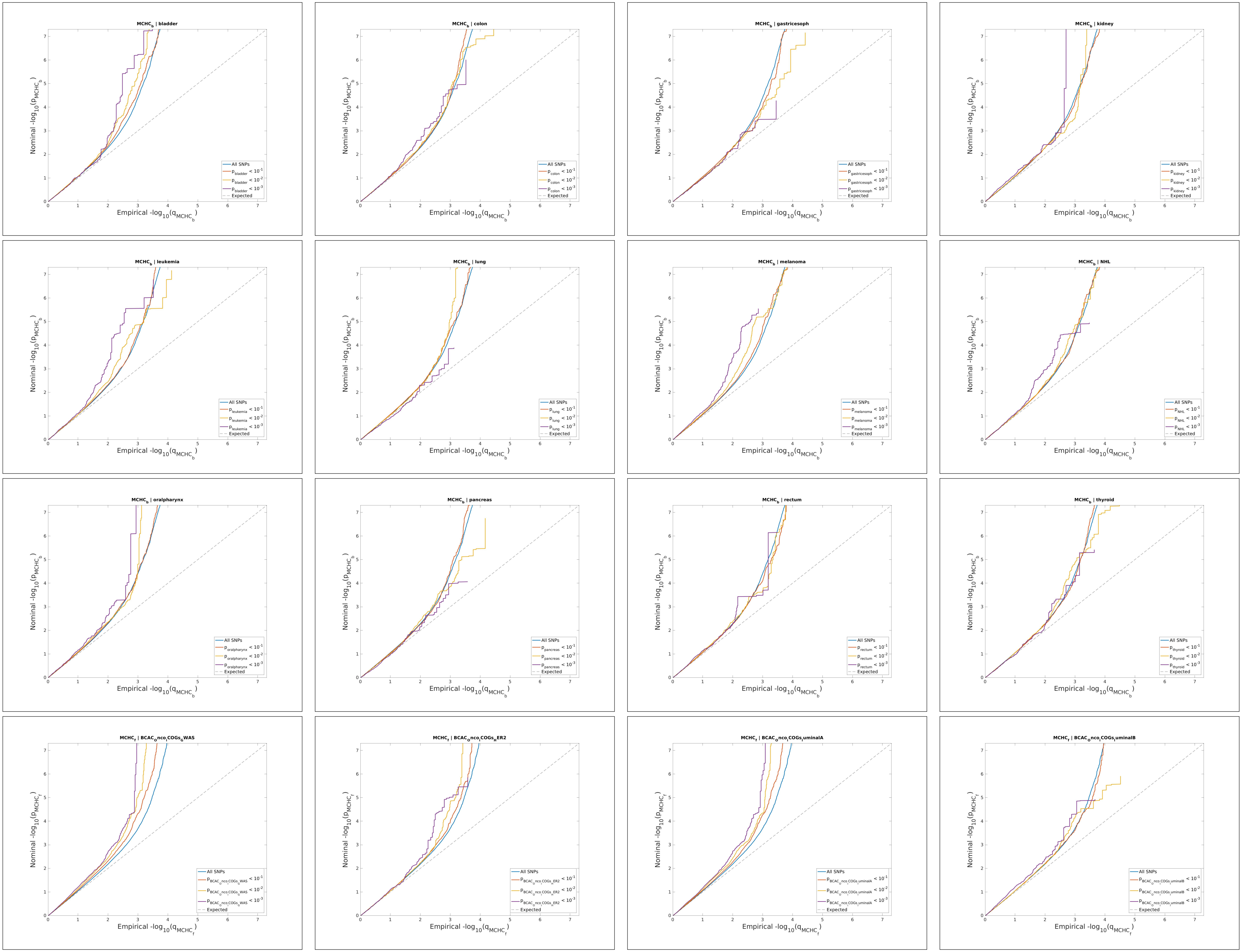

MCHC_b | bladder
MCHC_b | colon
MCHC_b | gastricesoph
MCHC_b | kidney
MCHC_b | leukemia
MCHC_b | lung
MCHC_b | melanoma
MCHC_b | NHL
MCHC_b | oralpharynx
MCHC_b | pancreas
MCHC_b | rectum
MCHC_b | thyroid
MCHC_f | BCAC_Onco_COGs_GWAS
MCHC_f | BCAC_Onco_COGs_HER2
MCHC_f | BCAC_Onco_COGs_luminalA
MCHC_f | BCAC_Onco_COGs_luminalB
Nominal -log10(p)
Empirical -log10(q)
All SNPs
Expected

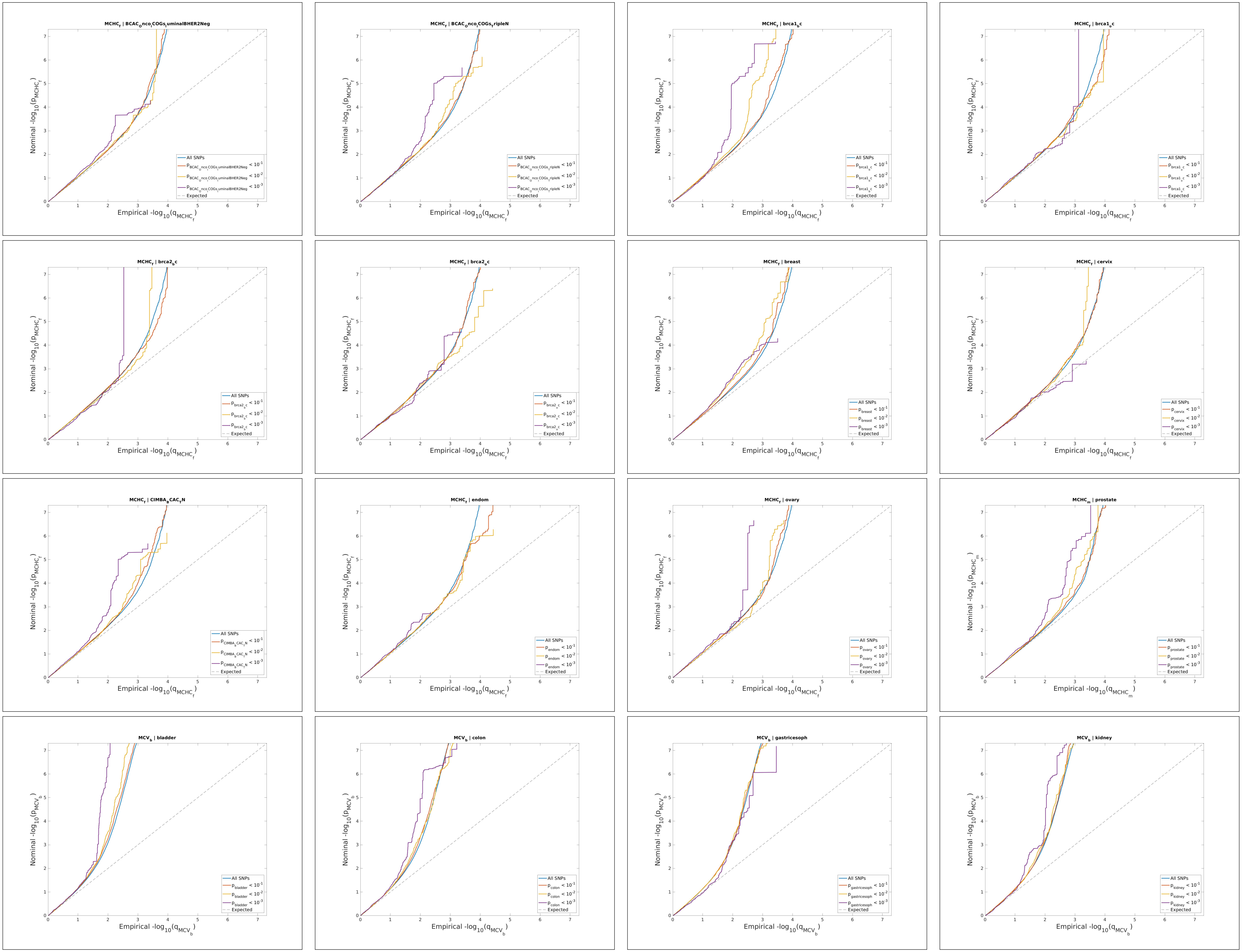

MCHCf | BCAConcoCOGsuminalBHER2Neg
MCHCf | BCAConcoCOGsripleN
MCHCf | brca1bc
MCHCf | brca1oc
MCHCf | brca2bc
MCHCf | brca2oc
MCHCf | breast
MCHCf | cervix
MCHCf | CIMBABCACrN
MCHCf | endom
MCHCf | ovary
MCHCm | prostate
MCVb | bladder
MCVb | colon
MCVb | gastricesoph
MCVb | kidney
Nominal -log10(pMCHCf)
Empirical -log10(qMCHCf)
Nominal -log10(pMCHCm)
Empirical -log10(qMCHCm)
Nominal -log10(pMCVb)
Empirical -log10(qMCVb)
All SNPs
Expected

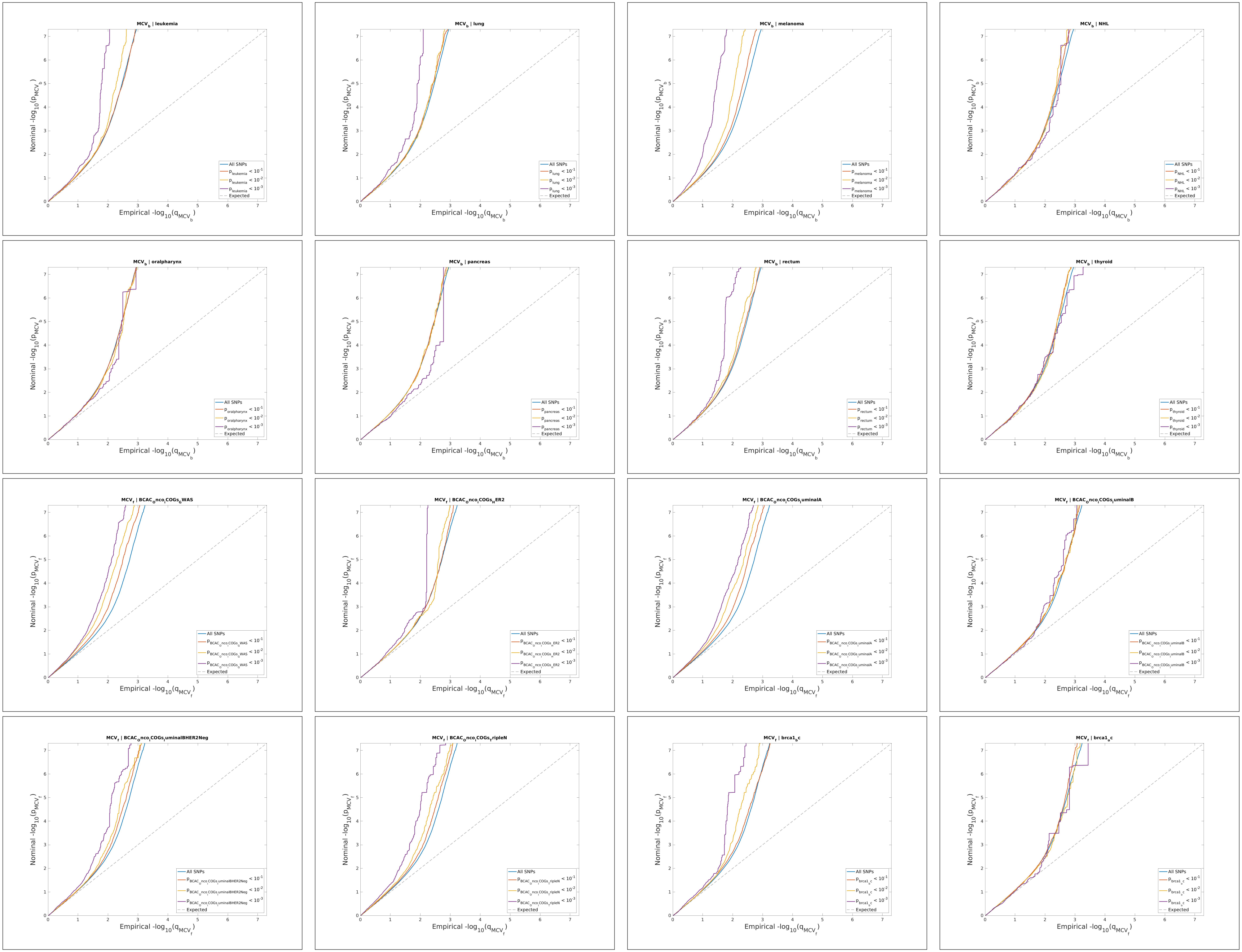

MCVb | leukemia
MCVb | lung
MCVb | melanoma
MCVb | NHL
MCVb | oralpharynx
MCVb | pancreas
MCVb | rectum
MCVb | thyroid
MCVf | BCAC_OncoCOGs_GWAS
MCVf | BCAC_OncoCOGs_HER2
MCVf | BCAC_OncoCOGs_LuminalA
MCVf | BCAC_OncoCOGs_LuminalB
MCVf | BCAC_OncoCOGs_LuminalBHER2Neg
MCVf | BCAC_OncoCOGs_TripleN
MCVf | brca1_bc
MCVf | brca1_oc
Nominal -log10(pMCV)
Empirical -log10(qMCV)
All SNPs
Expected

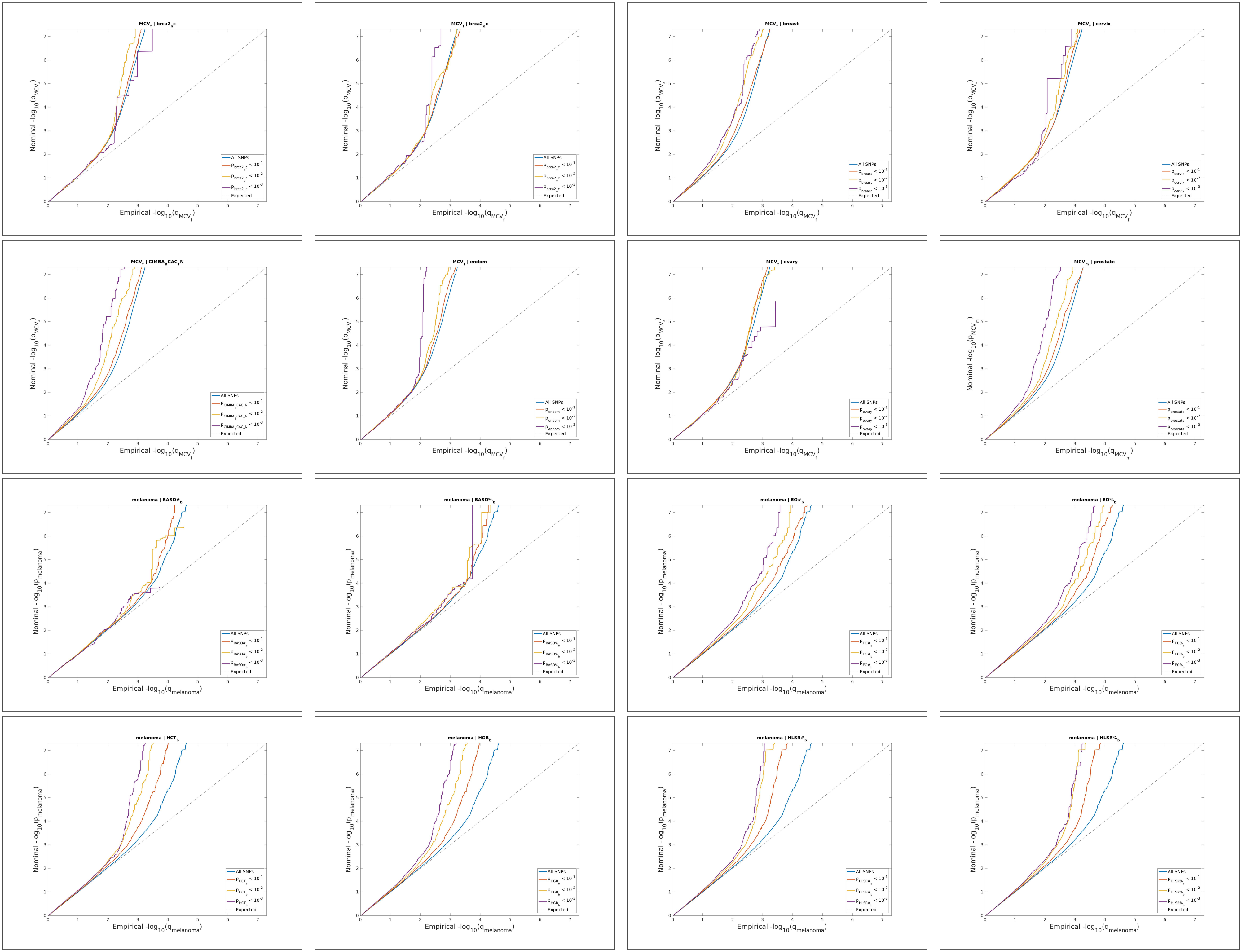

MCVf | brca2bc
MCVf | brca2oc
MCVf | breast
MCVf | cervix
MCVf | CIMBABCACTN
MCVf | endom
MCVf | ovary
MCVm | prostate
melanoma | BASO#b
melanoma | BASO%b
melanoma | EO#b
melanoma | EO%b
melanoma | HCTb
melanoma | HGBb
melanoma | HLSR#b
melanoma | HLSR%b
Nominal -log10(pMCVf)
Empirical -log10(qMCVf)
Nominal -log10(pMCVm)
Empirical -log10(qMCVm)
Nominal -log10(pmelanoma)
Empirical -log10(qmelanoma)
All SNPs
< 10-1
< 10-2
< 10-3
Expected

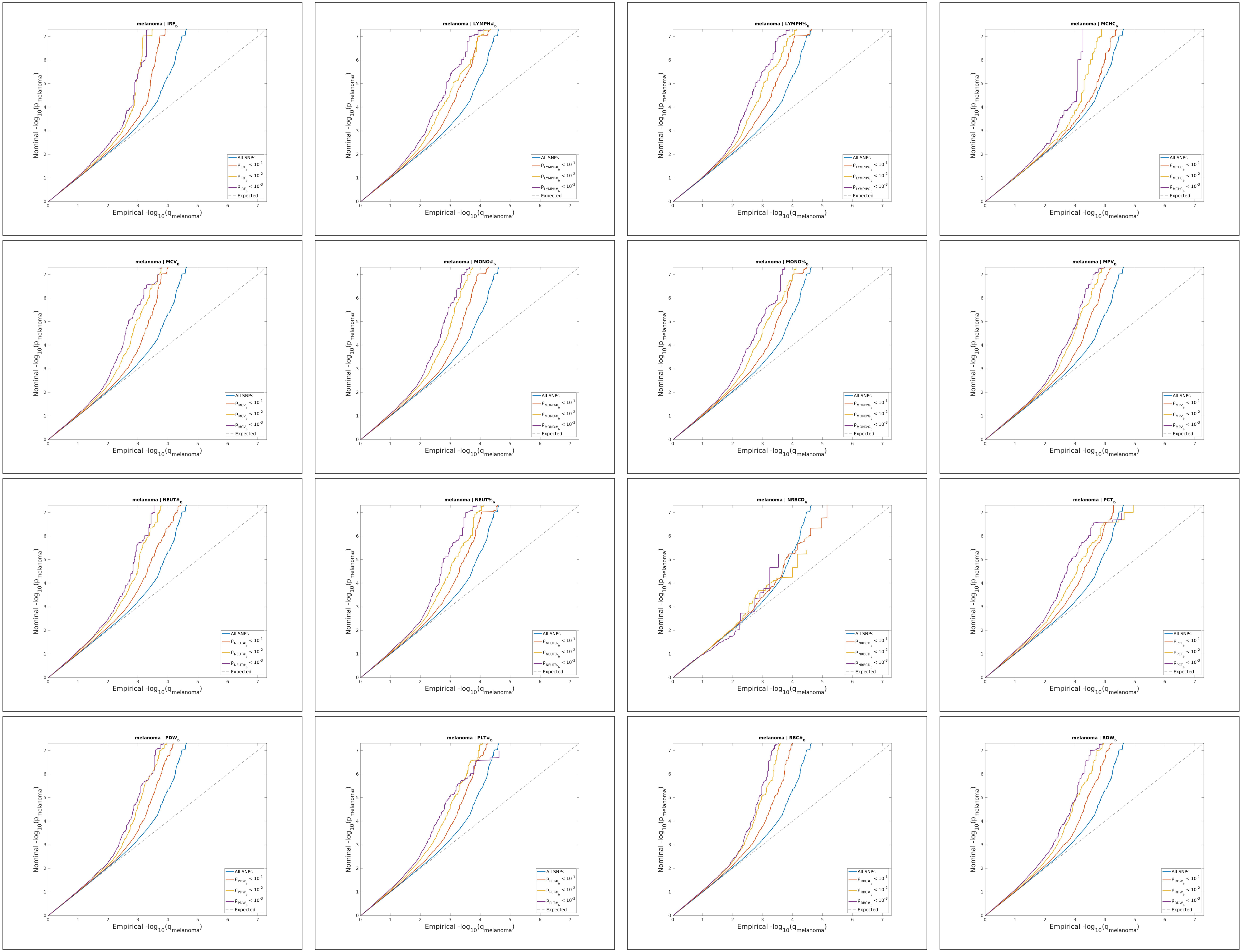
melanoma | IRFb
melanoma | LYMPH#b
melanoma | LYMPH%b
melanoma | MCHCb
melanoma | MCVb
melanoma | MONO#b
melanoma | MONO%b
melanoma | MPVb
melanoma | NEUT#b
melanoma | NEUT%b
melanoma | NRBCDb
melanoma | PCTb
melanoma | PDWb
melanoma | PLT#b
melanoma | RBC#b
melanoma | RDWb
Nominal -log10(pmelanoma)
Empirical -log10(qmelanoma)
All SNPs
Expected

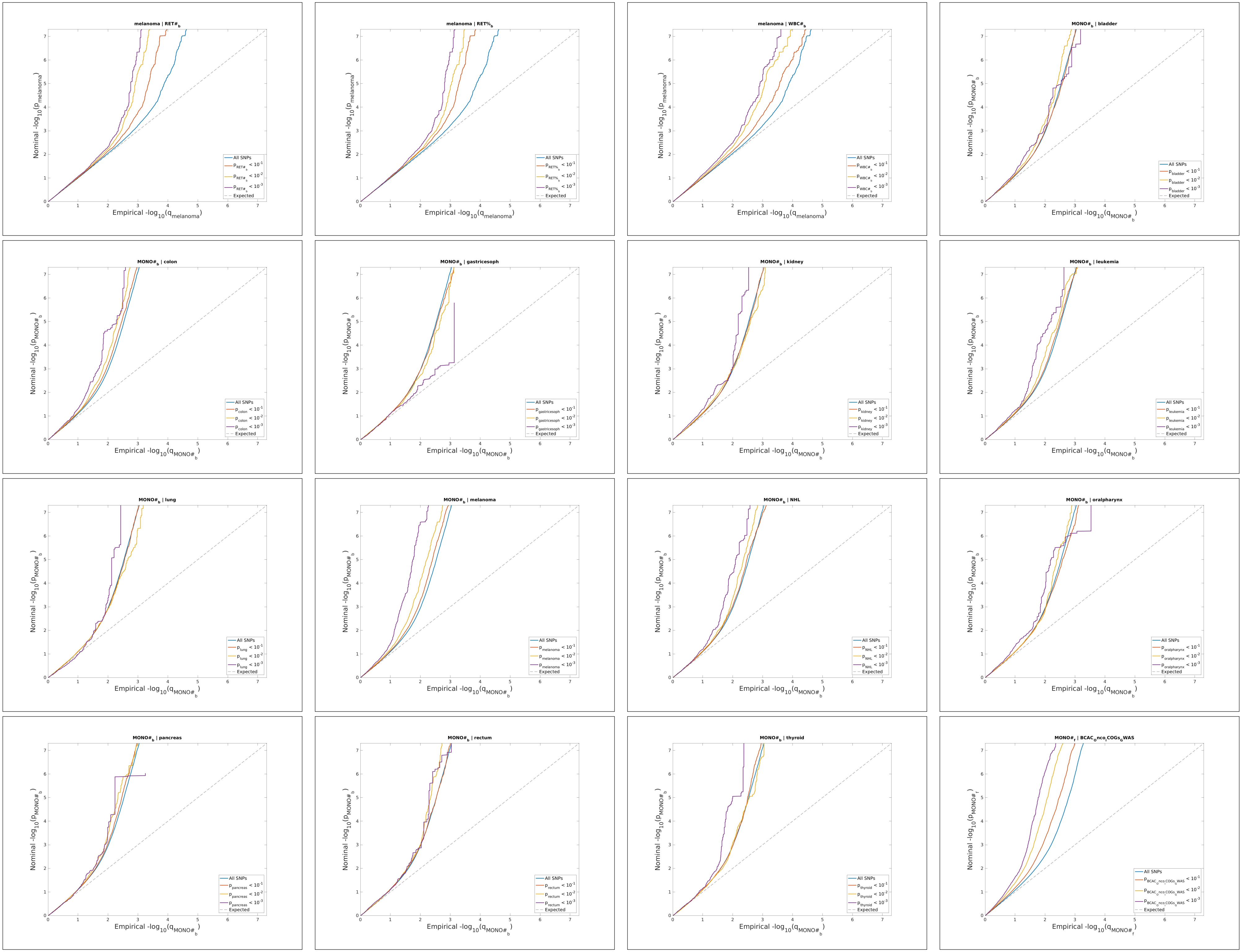
melanoma | RET#b
melanoma | RET%b
melanoma | WBC#b
MONO#b | bladder
MONO#b | colon
MONO#b | gastricesoph
MONO#b | kidney
MONO#b | leukemia
MONO#b | lung
MONO#b | melanoma
MONO#b | NHL
MONO#b | oralpharynx
MONO#b | pancreas
MONO#b | rectum
MONO#b | thyroid
MONO#f | BCAConcoCOGsGWAS
Nominal -log10(p)
Empirical -log10(q)
All SNPs
Expected

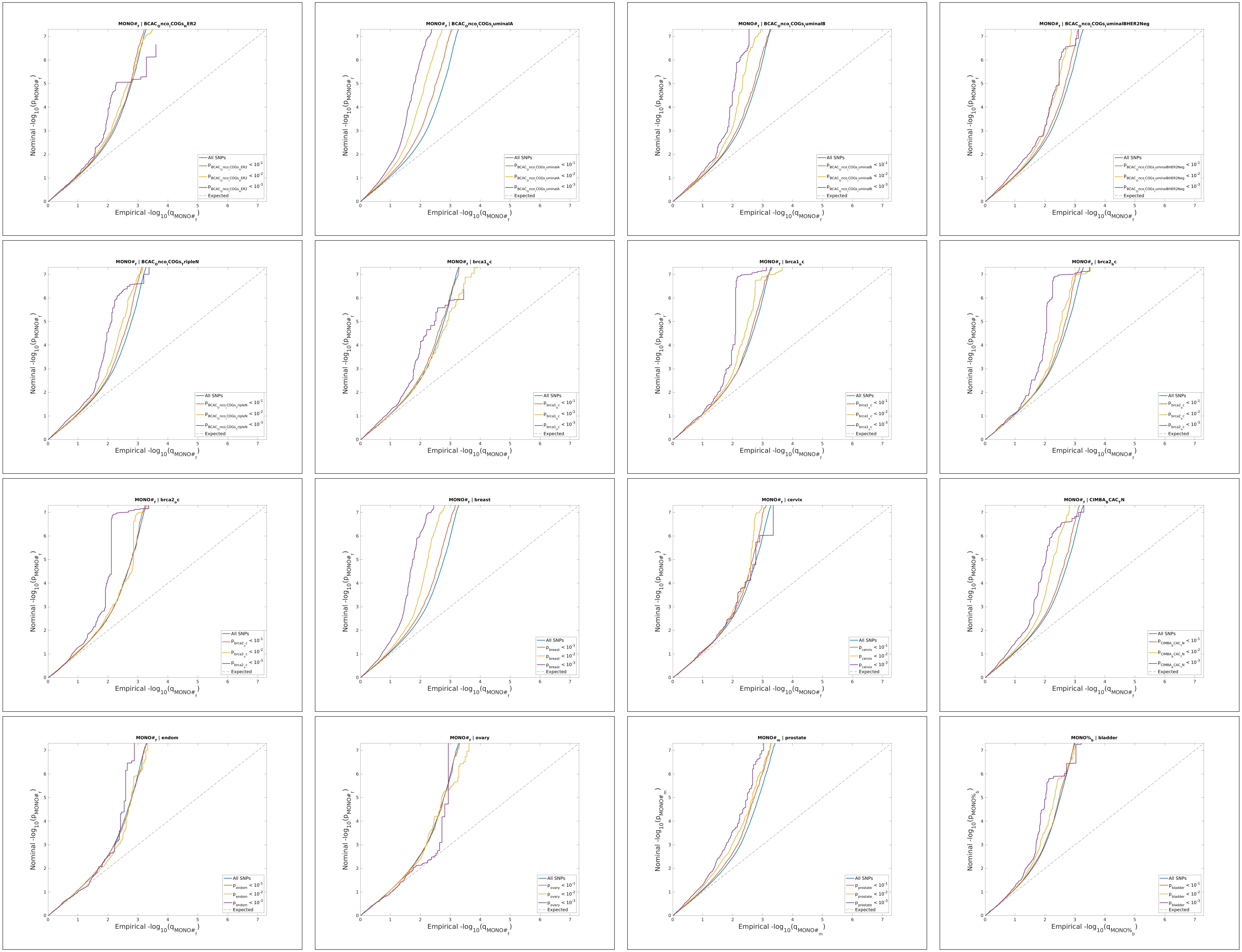
MONO#f | BCAConcoCOGsHER2
MONO#f | BCAConcoCOGsLuminalA
MONO#f | BCAConcoCOGsLuminalB
MONO#f | BCAConcoCOGsLuminalBHER2Neg
MONO#f | BCAConcoCOGsTripleN
MONO#f | brca1bc
MONO#f | brca1oc
MONO#f | brca2bc
MONO#f | brca2oc
MONO#f | breast
MONO#f | cervix
MONO#f | CIMBABCACTN
MONO#f | endom
MONO#f | ovary
MONO#m | prostate
MONO%b | bladder
Nominal -log10(p)
Empirical -log10(q)
All SNPs
Expected

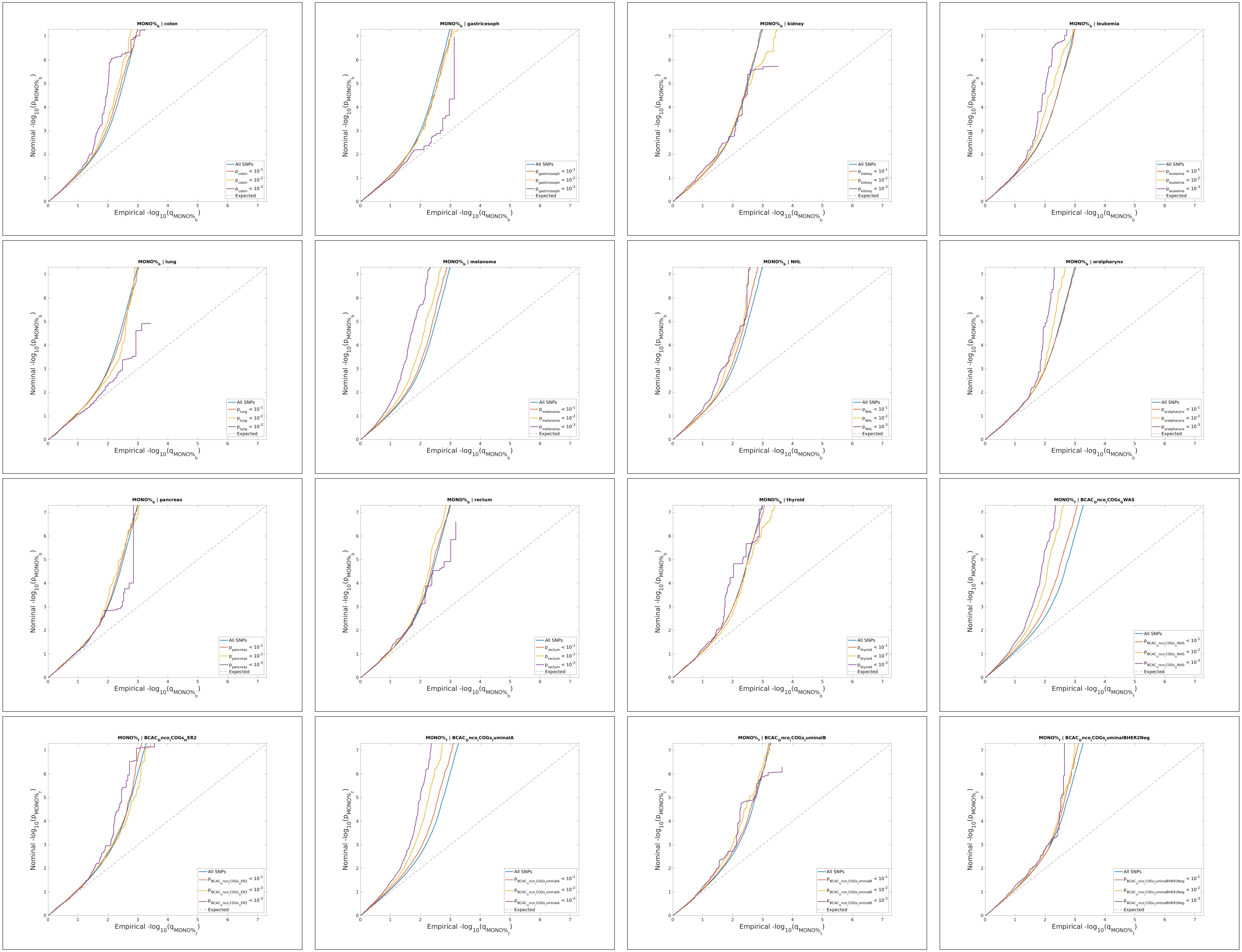
MONO%b | colon
MONO%b | gastricesoph
MONO%b | kidney
MONO%b | leukemia
MONO%b | lung
MONO%b | melanoma
MONO%b | NHL
MONO%b | oralpharynx
MONO%b | pancreas
MONO%b | rectum
MONO%b | thyroid
Nominal -log10(pMONO%b)
Empirical -log10(qMONO%b)
Nominal -log10(pMONO%f)
Empirical -log10(qMONO%f)
All SNPs
Expected

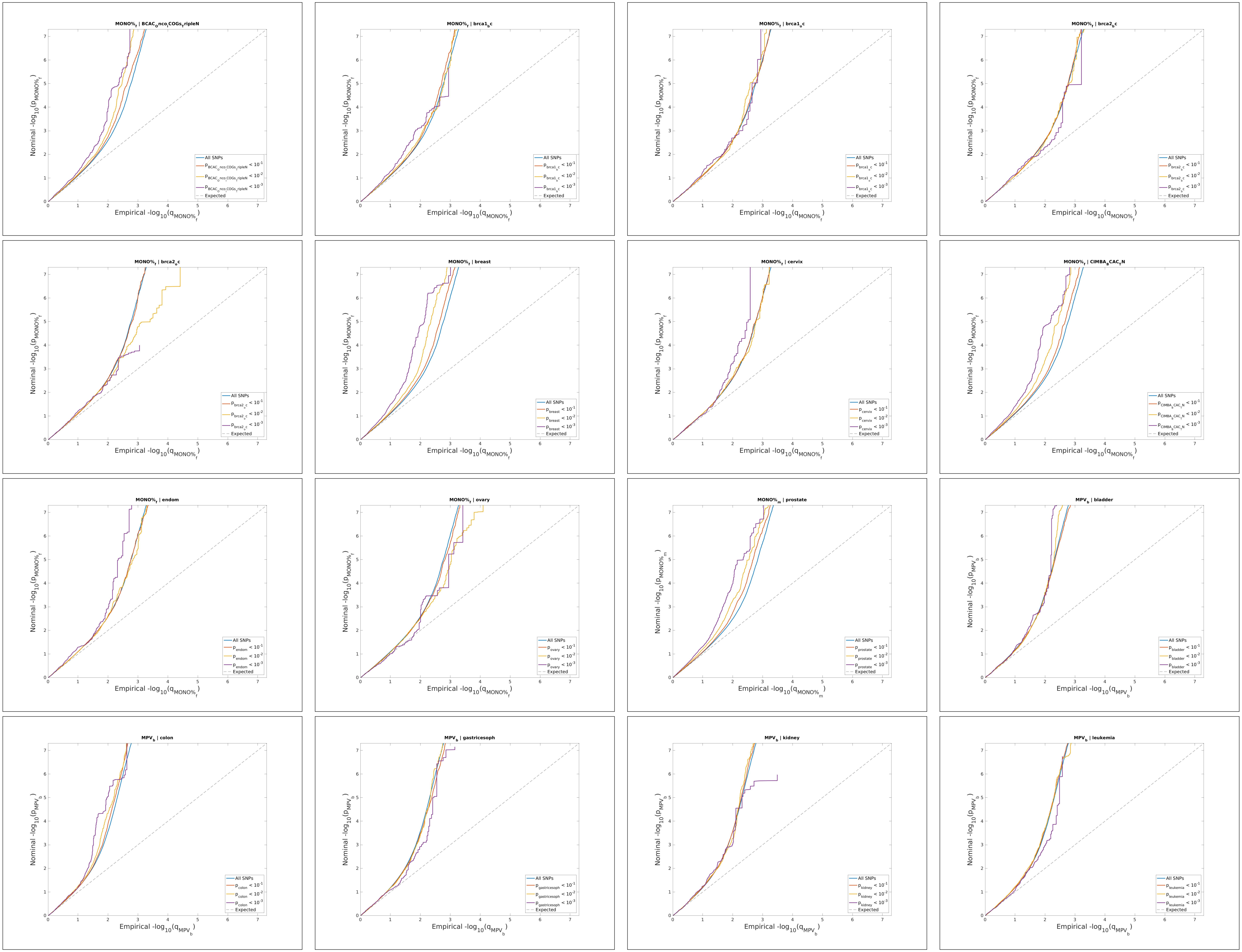

MONO%f | BCACOncoCOGsTripleN
MONO%f | brca1bc
MONO%f | brca1oc
MONO%f | brca2bc
MONO%f | brca2oc
MONO%f | breast
MONO%f | cervix
MONO%f | CIMBABCACTN
MONO%f | endom
MONO%f | ovary
MONO%m | prostate
MPVb | bladder
MPVb | colon
MPVb | gastricesoph
MPVb | kidney
MPVb | leukemia
Nominal -log10(p)
Empirical -log10(q)
All SNPs
Expected

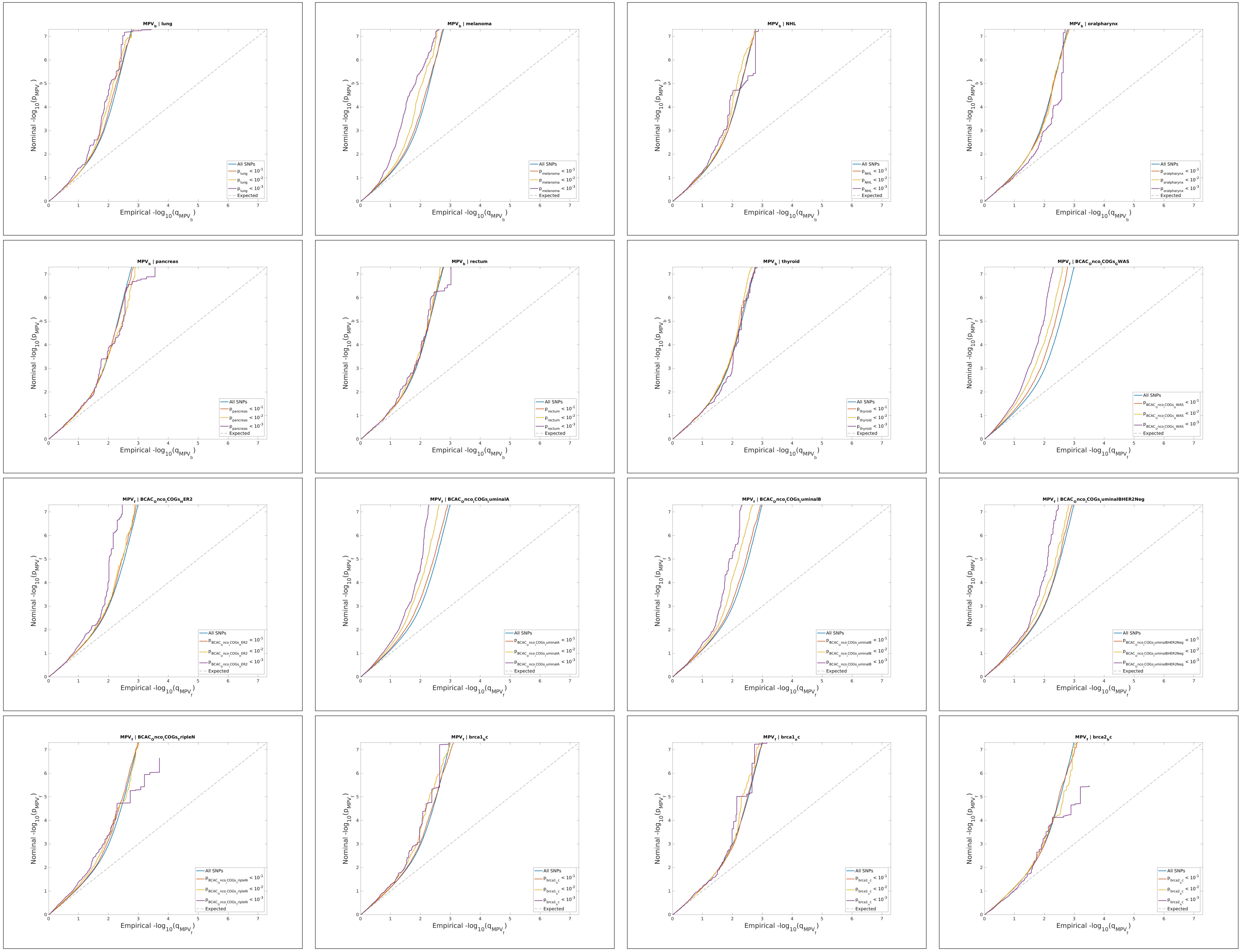
MPVb | lung
MPVb | melanoma
MPVb | NHL
MPVb | oralpharynx
MPVb | pancreas
MPVb | rectum
MPVb | thyroid
MPVf | BCAConcoCOGsGWAS
MPVf | BCAConcoCOGsHER2
MPVf | BCAConcoCOGsLuminalA
MPVf | BCAConcoCOGsLuminalB
MPVf | BCAConcoCOGsLuminalBHER2Neg
MPVf | BCAConcoCOGsTripleN
MPVf | brca1bc
MPVf | brca1oc
MPVf | brca2bc
Nominal -log10(pMPV)
Empirical -log10(qMPV)
All SNPs
Expected

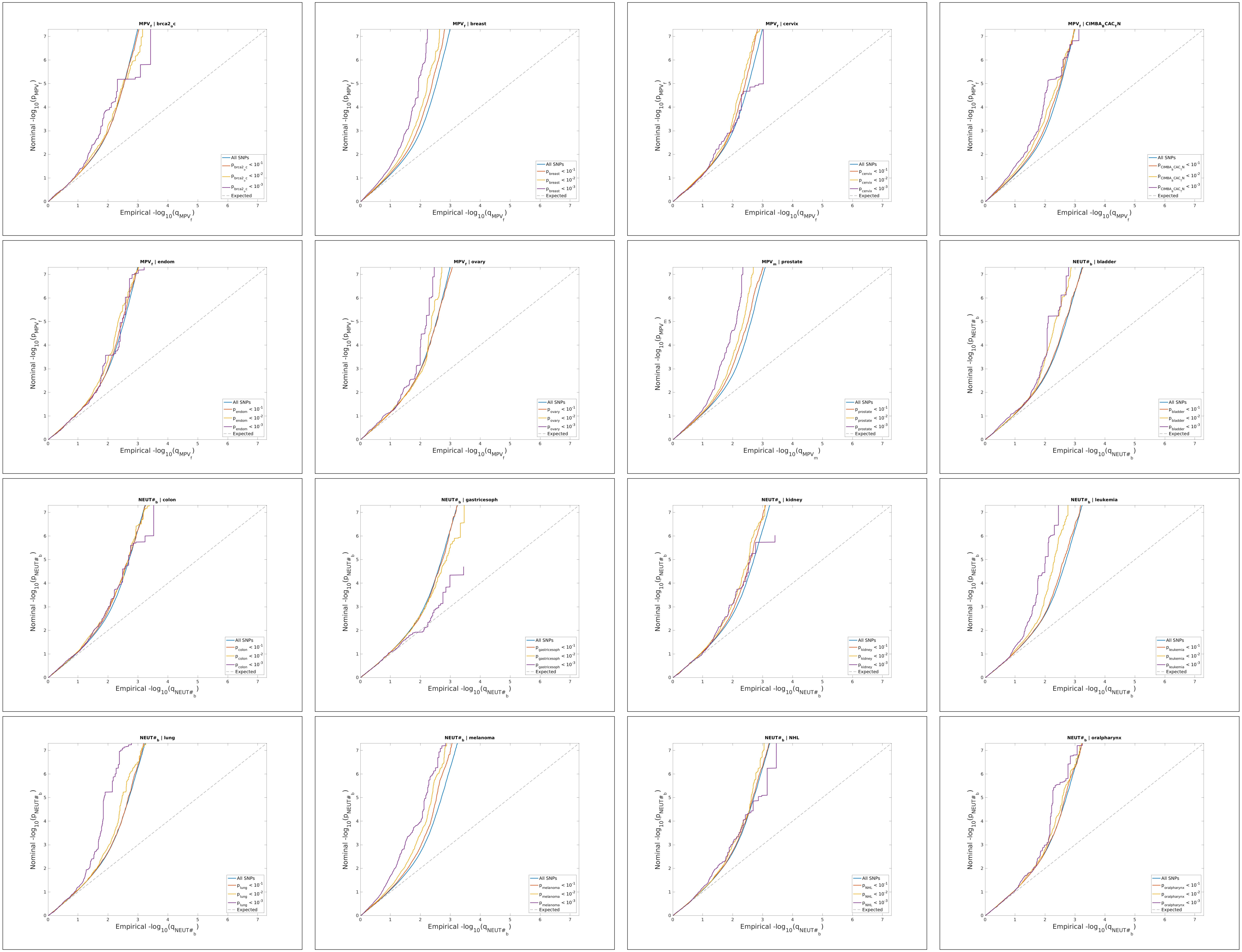

MPVf | brca2oc
Nominal -log10(pMPVf)
Empirical -log10(qMPVf)
All SNPs
pbrca2oc < 10-1
pbrca2oc < 10-2
pbrca2oc < 10-3
Expected
MPVf | breast
Nominal -log10(pMPVf)
Empirical -log10(qMPVf)
All SNPs
pbreast < 10-1
pbreast < 10-2
pbreast < 10-3
Expected
MPVf | cervix
Nominal -log10(pMPVf)
Empirical -log10(qMPVf)
All SNPs
pcervix < 10-1
pcervix < 10-2
pcervix < 10-3
Expected
MPVf | CIMBABCACrN
Nominal -log10(pMPVf)
Empirical -log10(qMPVf)
All SNPs
pCIMBABCACrN < 10-1
pCIMBABCACrN < 10-2
pCIMBABCACrN < 10-3
Expected
MPVf | endom
Nominal -log10(pMPVf)
Empirical -log10(qMPVf)
All SNPs
pendom < 10-1
pendom < 10-2
pendom < 10-3
Expected
MPVf | ovary
Nominal -log10(pMPVf)
Empirical -log10(qMPVf)
All SNPs
povary < 10-1
povary < 10-2
povary < 10-3
Expected
MPVm | prostate
Nominal -log10(pMPVm)
Empirical -log10(qMPVm)
All SNPs
pprostate < 10-1
pprostate < 10-2
pprostate < 10-3
Expected
NEUT#b | bladder
Nominal -log10(pNEUT#b)
Empirical -log10(qNEUT#b)
All SNPs
pbladder < 10-1
pbladder < 10-2
pbladder < 10-3
Expected
NEUT#b | colon
Nominal -log10(pNEUT#b)
Empirical -log10(qNEUT#b)
All SNPs
pcolon < 10-1
pcolon < 10-2
pcolon < 10-3
Expected
NEUT#b | gastricesoph
Nominal -log10(pNEUT#b)
Empirical -log10(qNEUT#b)
All SNPs
pgastricesoph < 10-1
pgastricesoph < 10-2
pgastricesoph < 10-3
Expected
NEUT#b | kidney
Nominal -log10(pNEUT#b)
Empirical -log10(qNEUT#b)
All SNPs
pkidney < 10-1
pkidney < 10-2
pkidney < 10-3
Expected
NEUT#b | leukemia
Nominal -log10(pNEUT#b)
Empirical -log10(qNEUT#b)
All SNPs
pleukemia < 10-1
pleukemia < 10-2
pleukemia < 10-3
Expected
NEUT#b | lung
Nominal -log10(pNEUT#b)
Empirical -log10(qNEUT#b)
All SNPs
plung < 10-1
plung < 10-2
plung < 10-3
Expected
NEUT#b | melanoma
Nominal -log10(pNEUT#b)
Empirical -log10(qNEUT#b)
All SNPs
pmelanoma < 10-1
pmelanoma < 10-2
pmelanoma < 10-3
Expected
NEUT#b | NHL
Nominal -log10(pNEUT#b)
Empirical -log10(qNEUT#b)
All SNPs
pNHL < 10-1
pNHL < 10-2
pNHL < 10-3
Expected
NEUT#b | oralpharynx
Nominal -log10(pNEUT#b)
Empirical -log10(qNEUT#b)
All SNPs
poralpharynx < 10-1
poralpharynx < 10-2
poralpharynx < 10-3
Expected

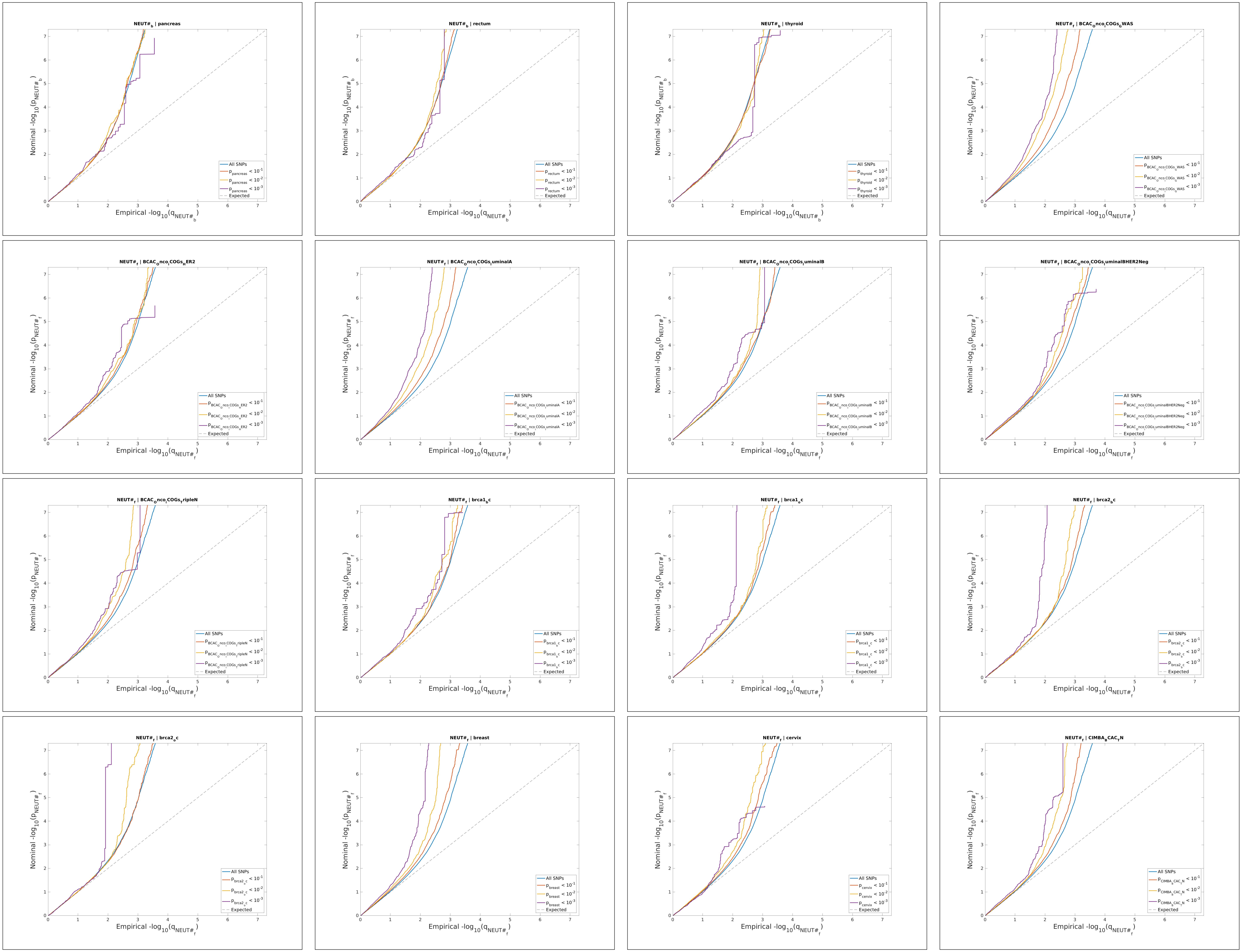

NEUT#b | pancreas
NEUT#b | rectum
NEUT#b | thyroid
NEUT#f | BCAC_OncoCOGs_GWAS
NEUT#f | BCAC_OncoCOGs_HER2
NEUT#f | BCAC_OncoCOGs_LuminalA
NEUT#f | BCAC_OncoCOGs_LuminalB
NEUT#f | BCAC_OncoCOGs_LuminalBHER2Neg
NEUT#f | BCAC_OncoCOGs_TripleN
NEUT#f | brca1_bc
NEUT#f | brca1_oc
NEUT#f | brca2_bc
NEUT#f | brca2_oc
NEUT#f | breast
NEUT#f | cervix
NEUT#f | CIMBA_BCAC_TN
Nominal -log10(p)
Empirical -log10(q)
All SNPs
Expected

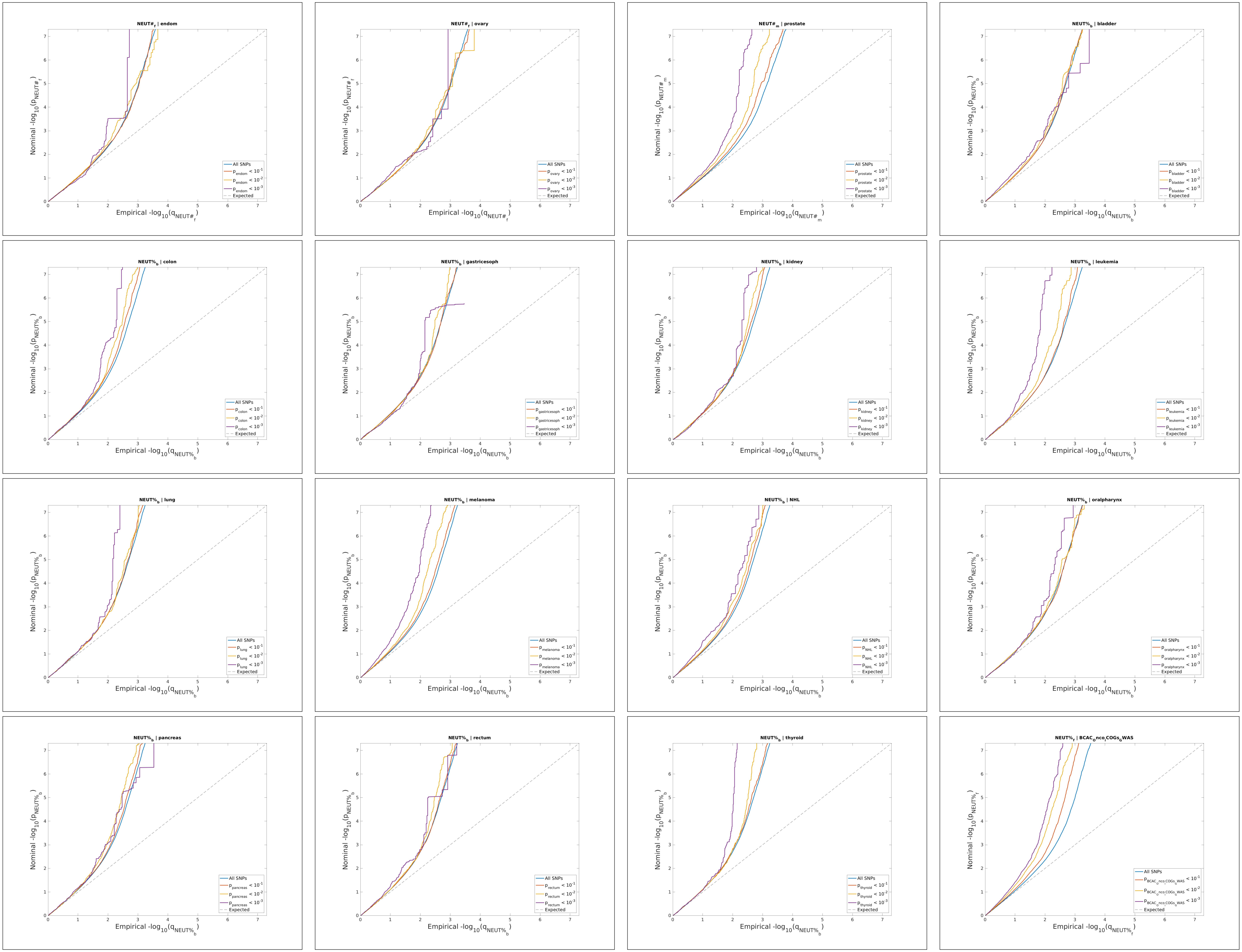

NEUT#f | endom
NEUT#f | ovary
NEUT#m | prostate
NEUT%b | bladder
NEUT%b | colon
NEUT%b | gastricesoph
NEUT%b | kidney
NEUT%b | leukemia
NEUT%b | lung
NEUT%b | melanoma
NEUT%b | NHL
NEUT%b | oralpharynx
NEUT%b | pancreas
NEUT%b | rectum
NEUT%b | thyroid
NEUT%f | BCACOncoCOGsGWAS
Nominal -log10(pNEUT#f)
Empirical -log10(qNEUT#f)
Nominal -log10(pNEUT#m)
Empirical -log10(qNEUT#m)
Nominal -log10(pNEUT%b)
Empirical -log10(qNEUT%b)
Nominal -log10(pNEUT%f)
Empirical -log10(qNEUT%f)
All SNPs
p < 10-1
p < 10-2
p < 10-3
Expected

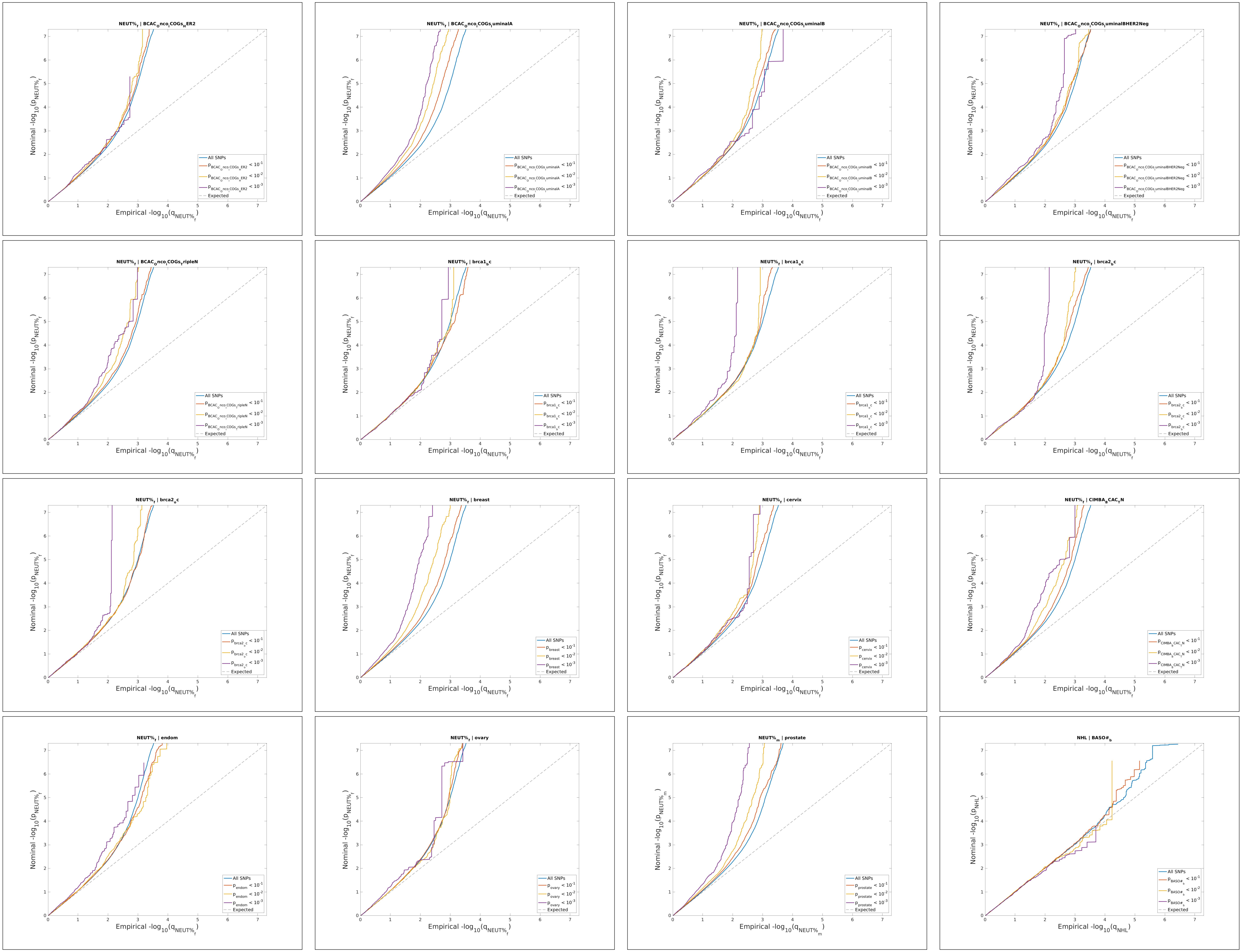
NEUT%f | BCAC_Onco_iCOGs_HER2
NEUT%f | BCAC_Onco_iCOGs_LuminalA
NEUT%f | BCAC_Onco_iCOGs_LuminalB
NEUT%f | BCAC_Onco_iCOGs_LuminalBHER2Neg
NEUT%f | BCAC_Onco_iCOGs_TripleN
NEUT%f | brca1_bc
NEUT%f | brca1_oc
NEUT%f | brca2_bc
NEUT%f | brca2_oc
NEUT%f | breast
NEUT%f | cervix
NEUT%f | CIMBA_BCAC_TN
NEUT%f | endom
NEUT%f | ovary
NEUT%m | prostate
NHL | BASO#b
Nominal -log10(p)
Empirical -log10(q)
All SNPs
< 10^-1
< 10^-2
< 10^-3
Expected

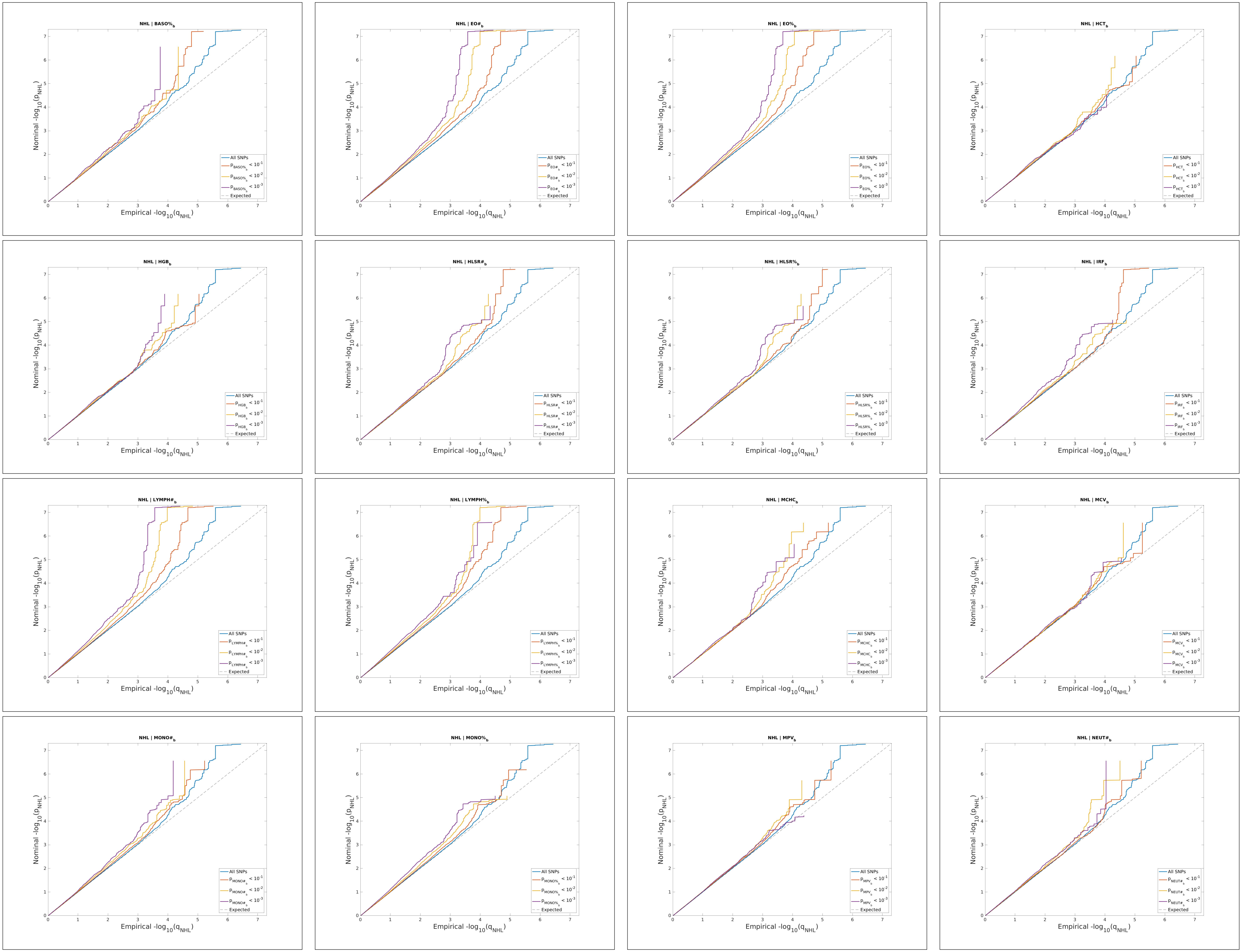
NHL | BASO%b
NHL | EO#b
NHL | EO%b
NHL | HCTb
NHL | HGBb
NHL | HLSR#b
NHL | HLSR%b
NHL | IRFb
NHL | LYMPH#b
NHL | LYMPH%b
NHL | MCHCb
NHL | MCVb
NHL | MONO#b
NHL | MONO%b
NHL | MPVb
NHL | NEUT#b
Nominal -log10(pNHL)
Empirical -log10(qNHL)
All SNPs
P < 10-1
P < 10-2
P < 10-3
Expected

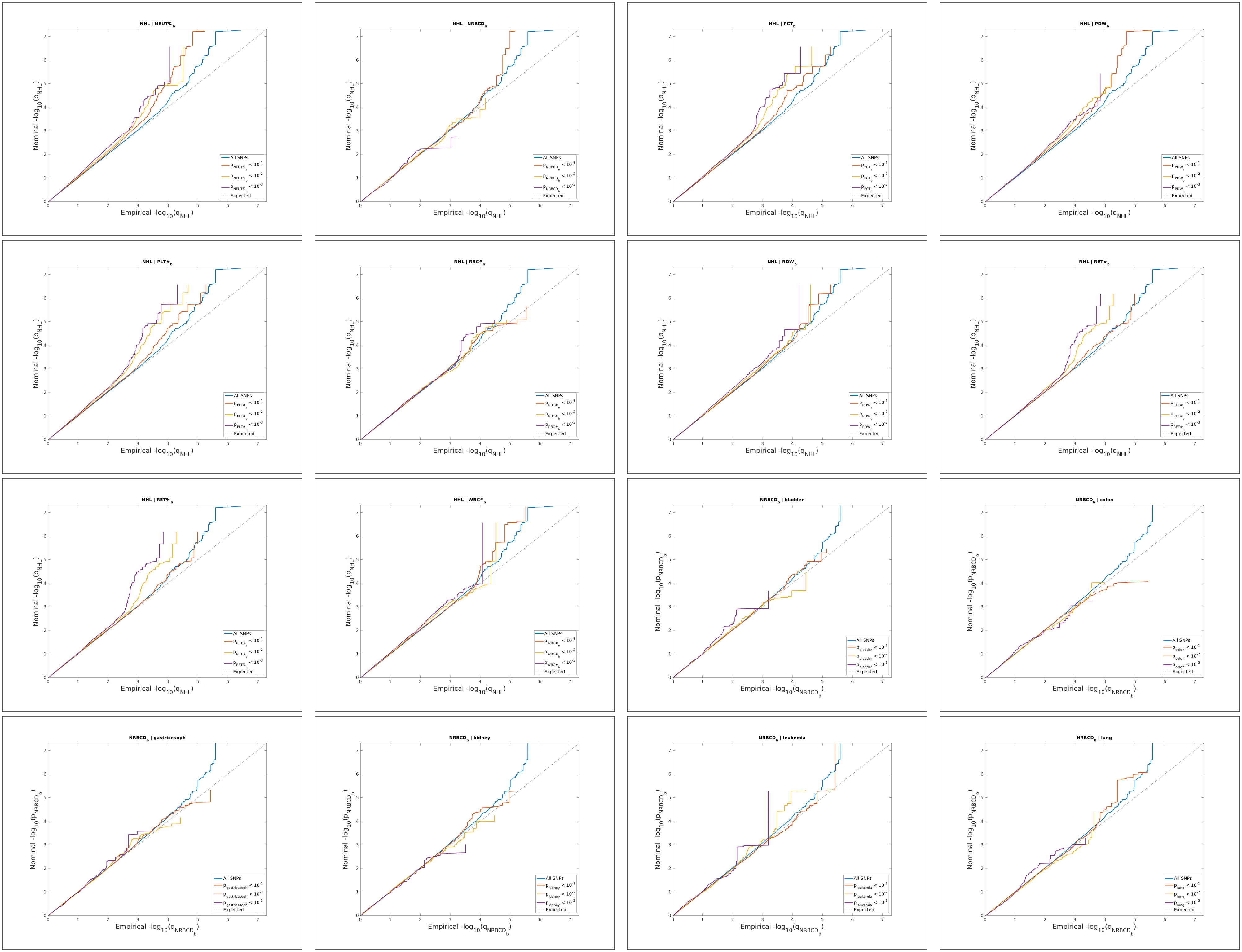

NHL | NEUT%b
NHL | NRBCDb
NHL | PCTb
NHL | PDWb
NHL | PLT#b
NHL | RBC#b
NHL | RDWb
NHL | RET#b
NHL | RET%b
NHL | WBC#b
NRBCDb | bladder
NRBCDb | colon
NRBCDb | gastricesoph
NRBCDb | kidney
NRBCDb | leukemia
NRBCDb | lung
Nominal -log10(pNHL)
Empirical -log10(qNHL)
Nominal -log10(pNRBCDb)
Empirical -log10(qNRBCDb)
All SNPs
< 10-1
< 10-2
< 10-3
Expected

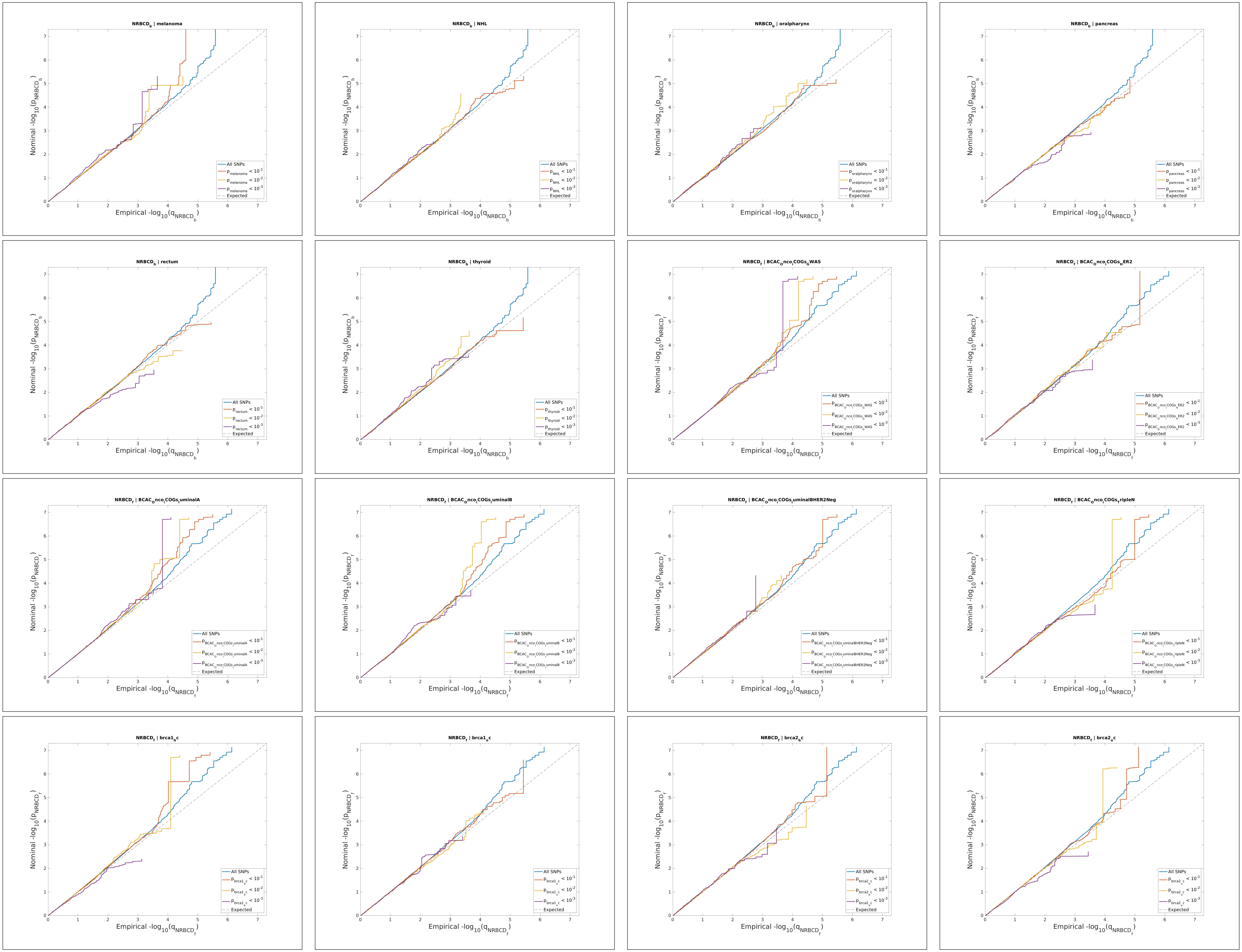

NRBCDb | melanoma
NRBCDb | NHL
NRBCDb | oralpharynx
NRBCDb | pancreas
NRBCDb | rectum
NRBCDb | thyroid
NRBCDf | BCACOncoICOGsGWAS
NRBCDf | BCACOncoICOGsHER2
NRBCDf | BCACOncoICOGsLuminalA
NRBCDf | BCACOncoICOGsLuminalB
NRBCDf | BCACOncoICOGsLuminalBHER2Neg
NRBCDf | BCACOncoICOGsTripleN
NRBCDf | brca1bc
NRBCDf | brca1oc
NRBCDf | brca2bc
NRBCDf | brca2oc
Nominal -log10(p)
Empirical -log10(q)
All SNPs
Expected

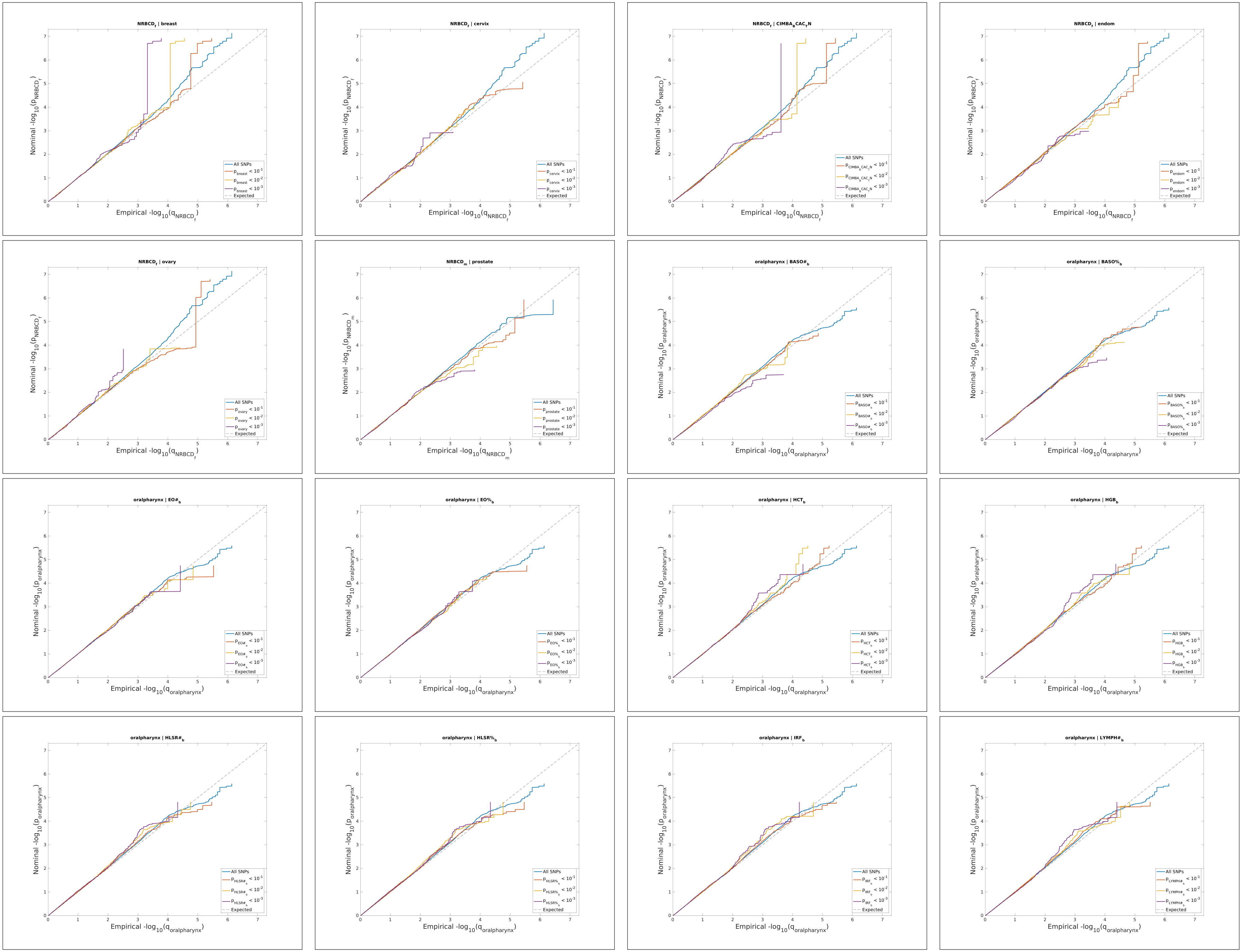

NRBCD$_f$ | breast
Nominal -log$_{10}$(p$_{NRBCD_f}$)
Empirical -log$_{10}$(q$_{NRBCD_f}$)
All SNPs
p$_{breast}$ < 10^{-1}
p$_{breast}$ < 10^{-2}
p$_{breast}$ < 10^{-3}
Expected
NRBCD$_f$ | cervix
p$_{cervix}$ < 10^{-1}
p$_{cervix}$ < 10^{-2}
p$_{cervix}$ < 10^{-3}
NRBCD$_f$ | CIMBA$_B$CAC$_T$N
p$_{CIMBA_BCAC_TN}$ < 10^{-1}
p$_{CIMBA_BCAC_TN}$ < 10^{-2}
p$_{CIMBA_BCAC_TN}$ < 10^{-3}
NRBCD$_f$ | endom
p$_{endom}$ < 10^{-1}
p$_{endom}$ < 10^{-2}
p$_{endom}$ < 10^{-3}
NRBCD$_f$ | ovary
p$_{ovary}$ < 10^{-1}
p$_{ovary}$ < 10^{-2}
p$_{ovary}$ < 10^{-3}
NRBCD$_m$ | prostate
Nominal -log$_{10}$(p$_{NRBCD_m}$)
Empirical -log$_{10}$(q$_{NRBCD_m}$)
p$_{prostate}$ < 10^{-1}
p$_{prostate}$ < 10^{-2}
p$_{prostate}$ < 10^{-3}
oralpharynx | BASO#$_b$
Nominal -log$_{10}$(p$_{oralpharynx}$)
Empirical -log$_{10}$(q$_{oralpharynx}$)
oralpharynx | BASO%$_b$
oralpharynx | EO#$_b$
oralpharynx | EO%$_b$
oralpharynx | HCT$_b$
oralpharynx | HGB$_b$
oralpharynx | HLSR#$_b$
oralpharynx | HLSR%$_b$
oralpharynx | IRF$_b$
oralpharynx | LYMPH#$_b$

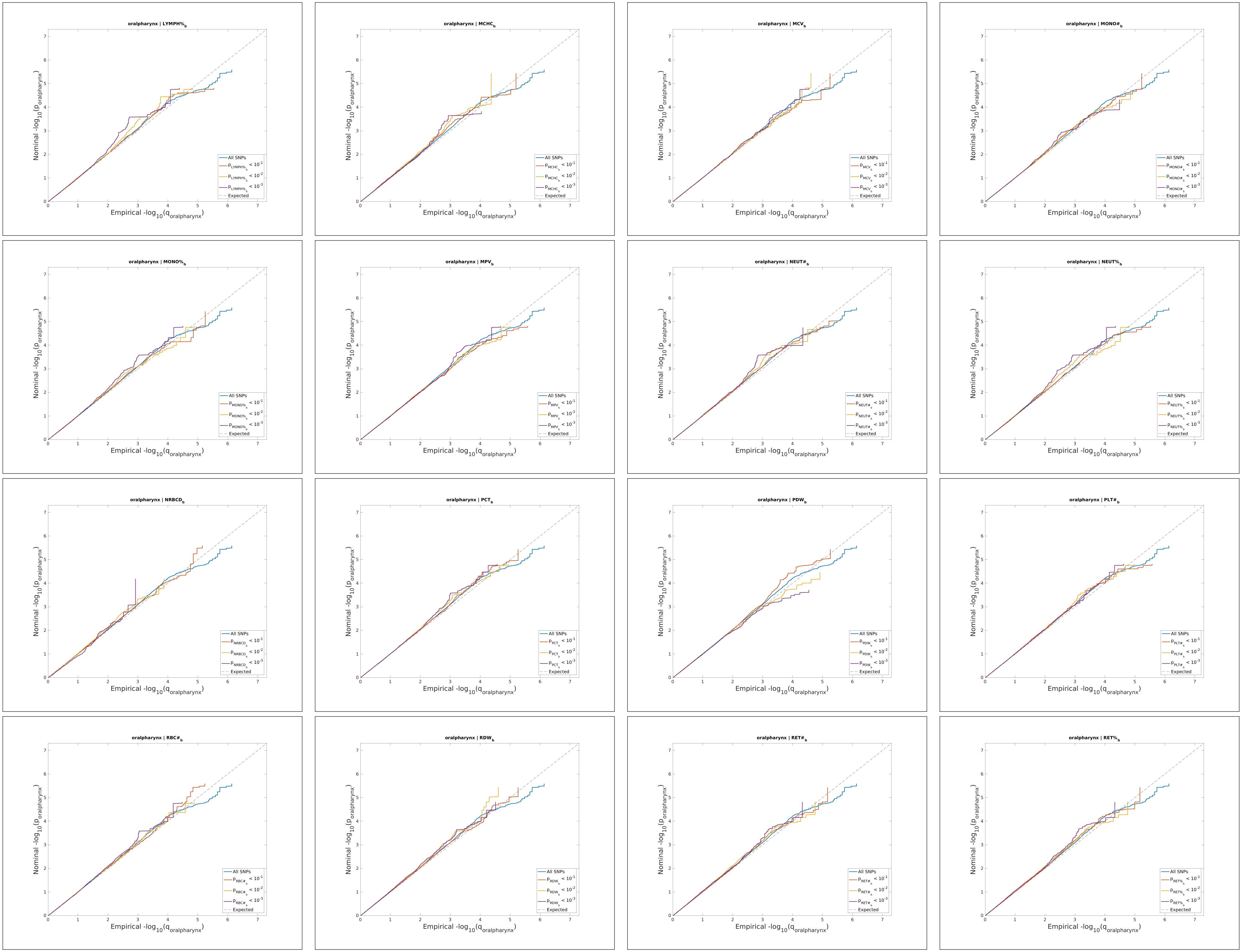
oralpharynx | LYMPH%b
oralpharynx | MCHCb
oralpharynx | MCVb
oralpharynx | MONO#b
oralpharynx | MONO%b
oralpharynx | MPVb
oralpharynx | NEUT#b
oralpharynx | NEUT%b
oralpharynx | NRBCDb
oralpharynx | PCTb
oralpharynx | PDWb
oralpharynx | PLT#b
oralpharynx | RBC#b
oralpharynx | RDWb
oralpharynx | RET#b
oralpharynx | RET%b
Nominal -log10(poralpharynx)
Empirical -log10(qoralpharynx)
All SNPs
Expected

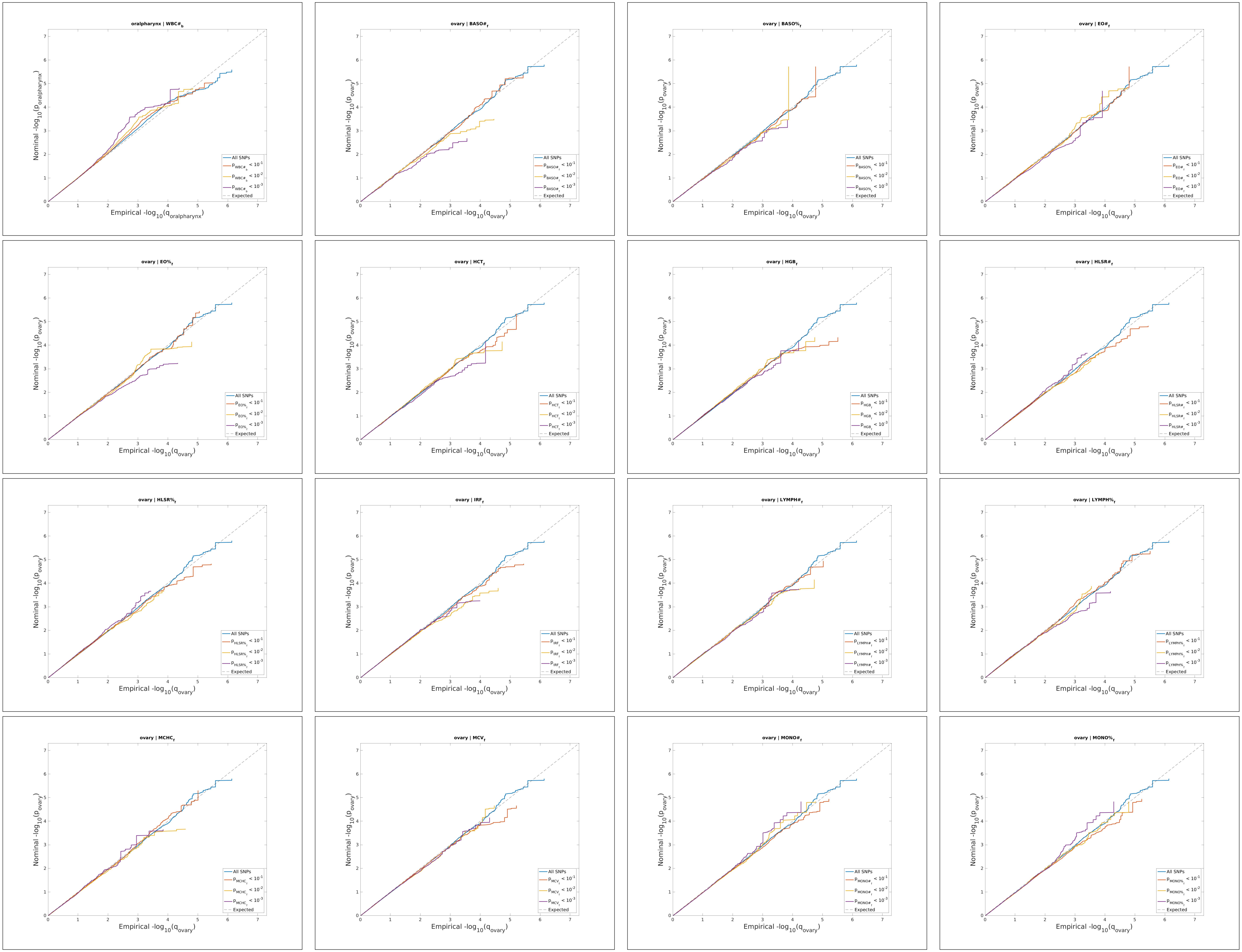

oralpharynx | WBC#b
ovary | BASO#f
ovary | BASO%f
ovary | EO#f
ovary | EO%f
ovary | HCT f
ovary | HGB f
ovary | HLSR#f
ovary | HLSR%f
ovary | IRF f
ovary | LYMPH#f
ovary | LYMPH%f
ovary | MCHC f
ovary | MCV f
ovary | MONO#f
ovary | MONO%f
Nominal -log10(p oralpharynx)
Empirical -log10(q oralpharynx)
Nominal -log10(p ovary)
Empirical -log10(q ovary)
All SNPs
< 10-1
< 10-2
< 10-3
Expected

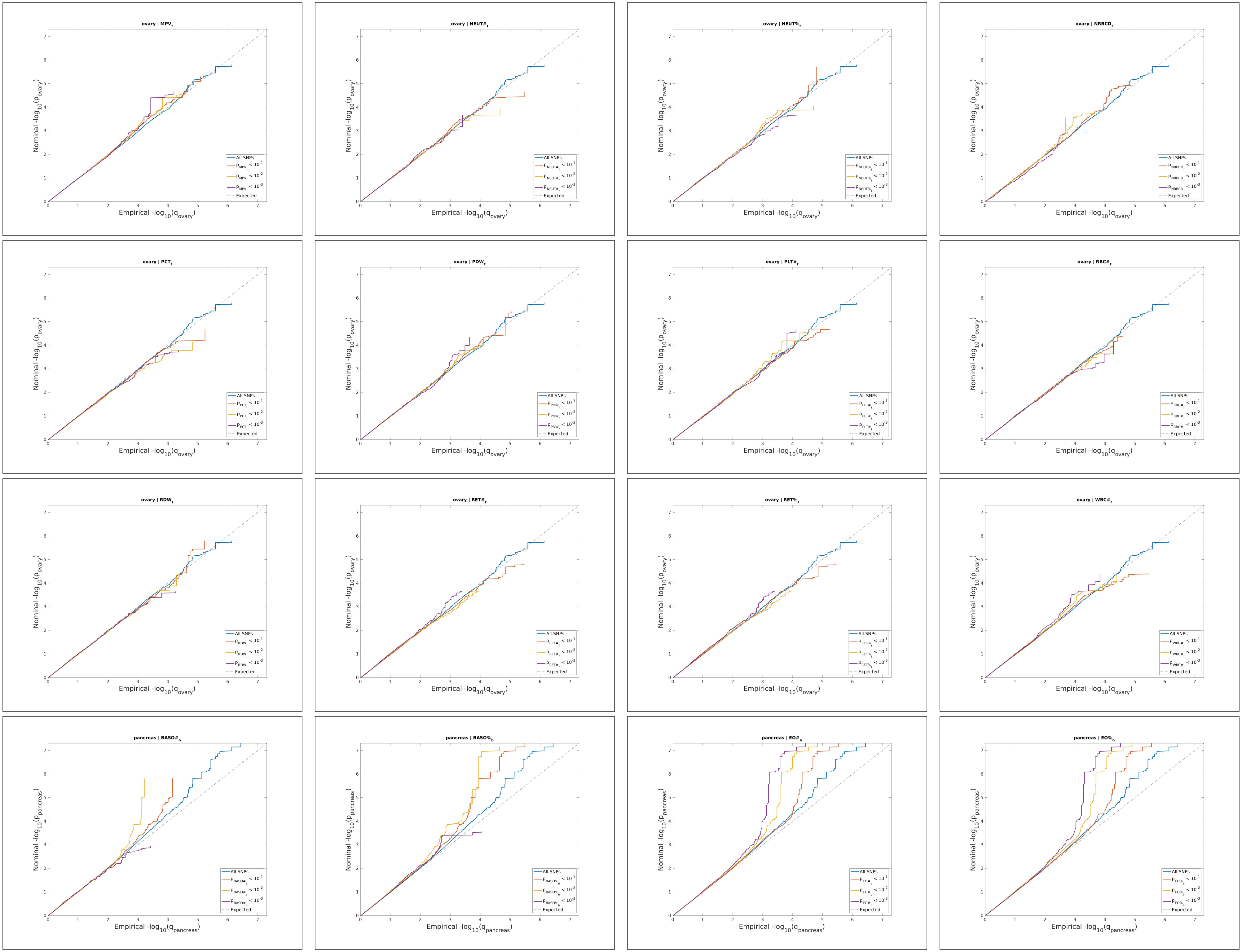
ovary | MPV_f
ovary | NEUT#_f
ovary | NEUT%_f
ovary | NRBCD_f
ovary | PCT_f
ovary | PDW_f
ovary | PLT#_f
ovary | RBC#_f
ovary | RDW_f
ovary | RET#_f
ovary | RET%_f
ovary | WBC#_f
pancreas | BASO#_b
pancreas | BASO%_b
pancreas | EO#_b
pancreas | EO%_b
Nominal -log10(p_ovary)
Empirical -log10(q_ovary)
Nominal -log10(p_pancreas)
Empirical -log10(q_pancreas)
All SNPs
< 10^-1
< 10^-2
< 10^-3
Expected

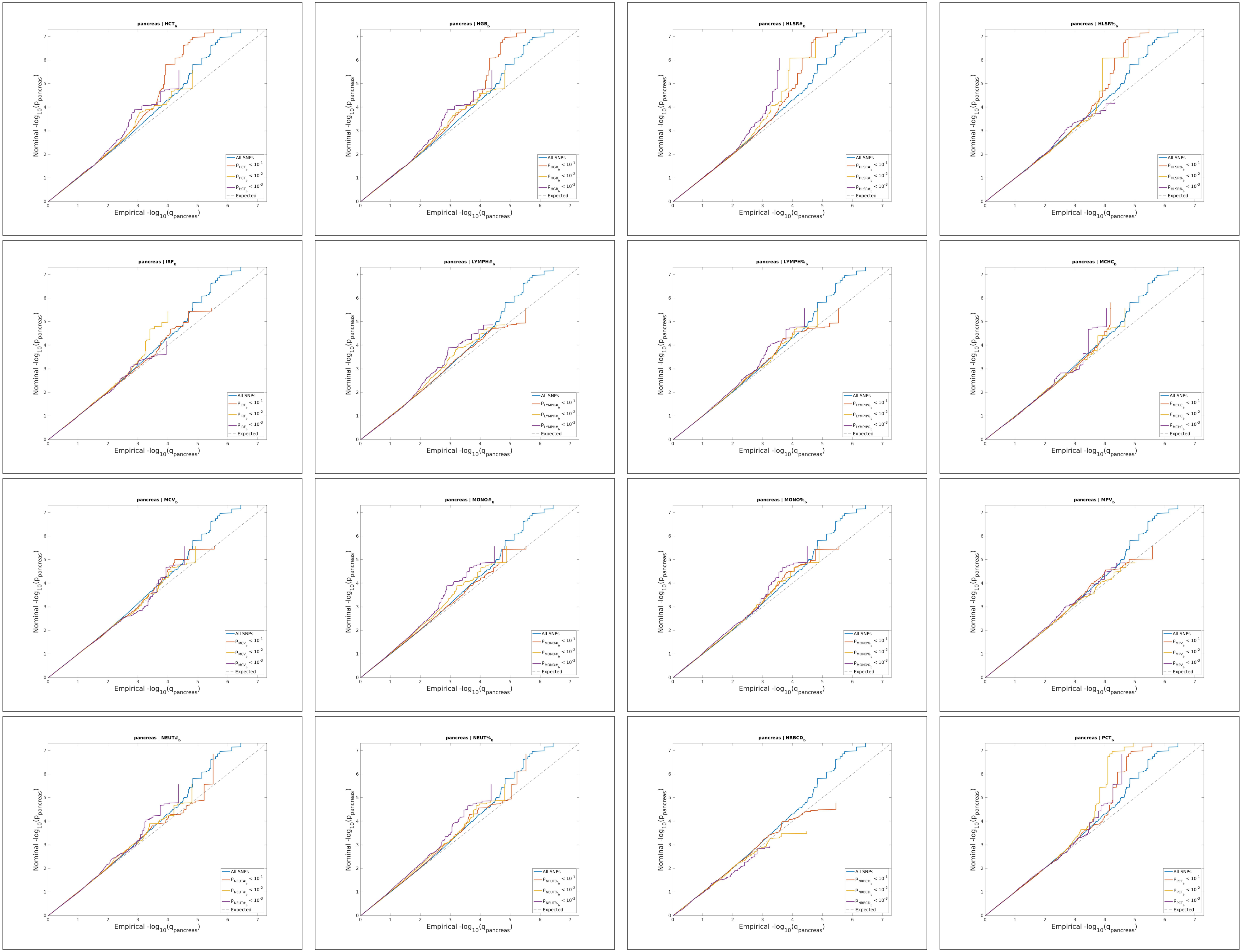

pancreas | HCT_b
pancreas | HGB_b
pancreas | HLSR#_b
pancreas | HLSR%_b
pancreas | IRF_b
pancreas | LYMPH#_b
pancreas | LYMPH%_b
pancreas | MCHC_b
pancreas | MCV_b
pancreas | MONO#_b
pancreas | MONO%_b
pancreas | MPV_b
pancreas | NEUT#_b
pancreas | NEUT%_b
pancreas | NRBCD_b
pancreas | PCT_b
Nominal -log10(p_pancreas)
Empirical -log10(q_pancreas)
All SNPs
Expected

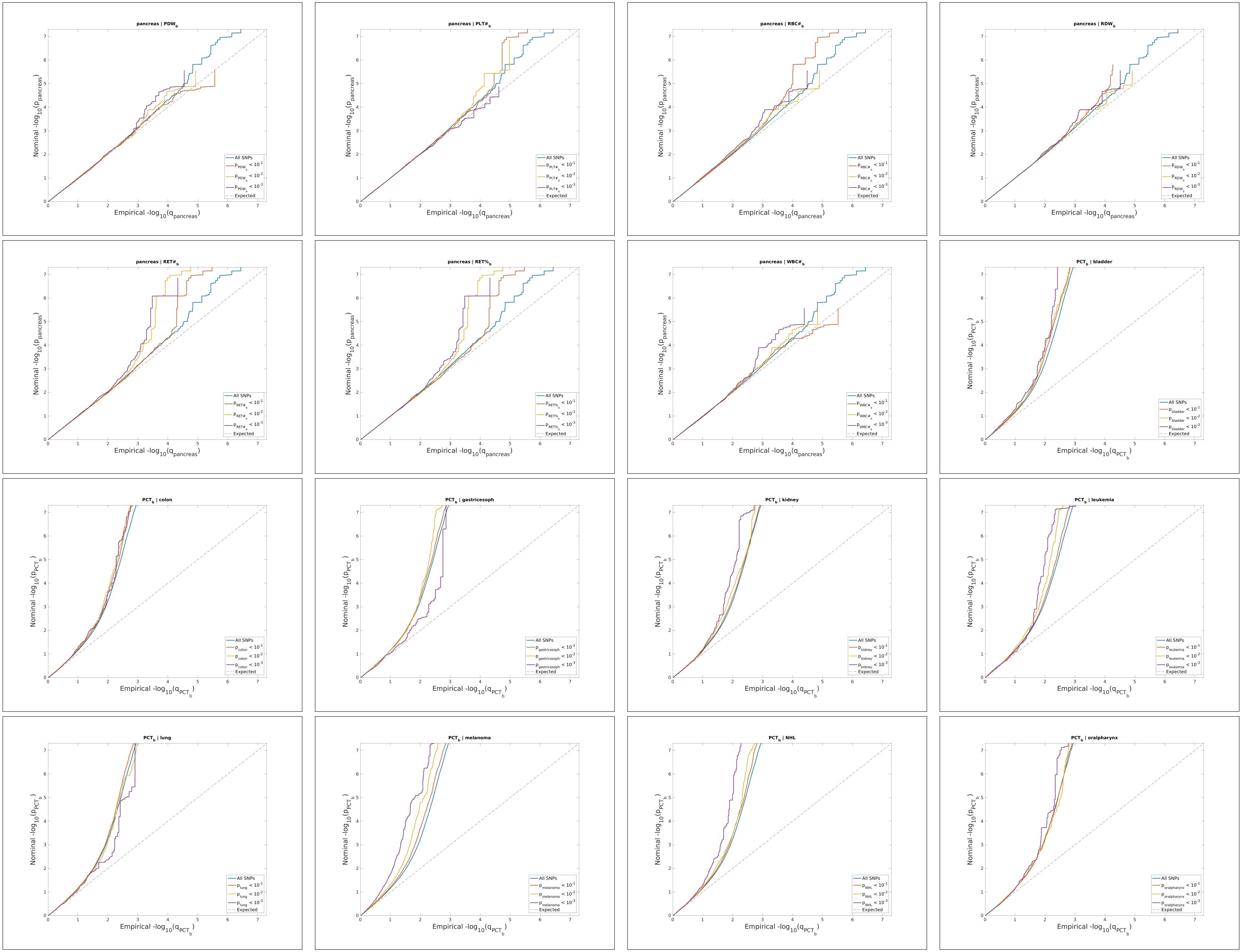

pancreas | PDW_b
pancreas | PLT#_b
pancreas | RBC#_b
pancreas | RDW_b
pancreas | RET#_b
pancreas | RET%_b
pancreas | WBC#_b
PCT_b | bladder
PCT_b | colon
PCT_b | gastricesoph
PCT_b | kidney
PCT_b | leukemia
PCT_b | lung
PCT_b | melanoma
PCT_b | NHL
PCT_b | oralpharynx
Nominal -log10(p pancreas)
Empirical -log10(q pancreas)
Nominal -log10(p PCT_b)
Empirical -log10(q PCT_b)
All SNPs
Expected

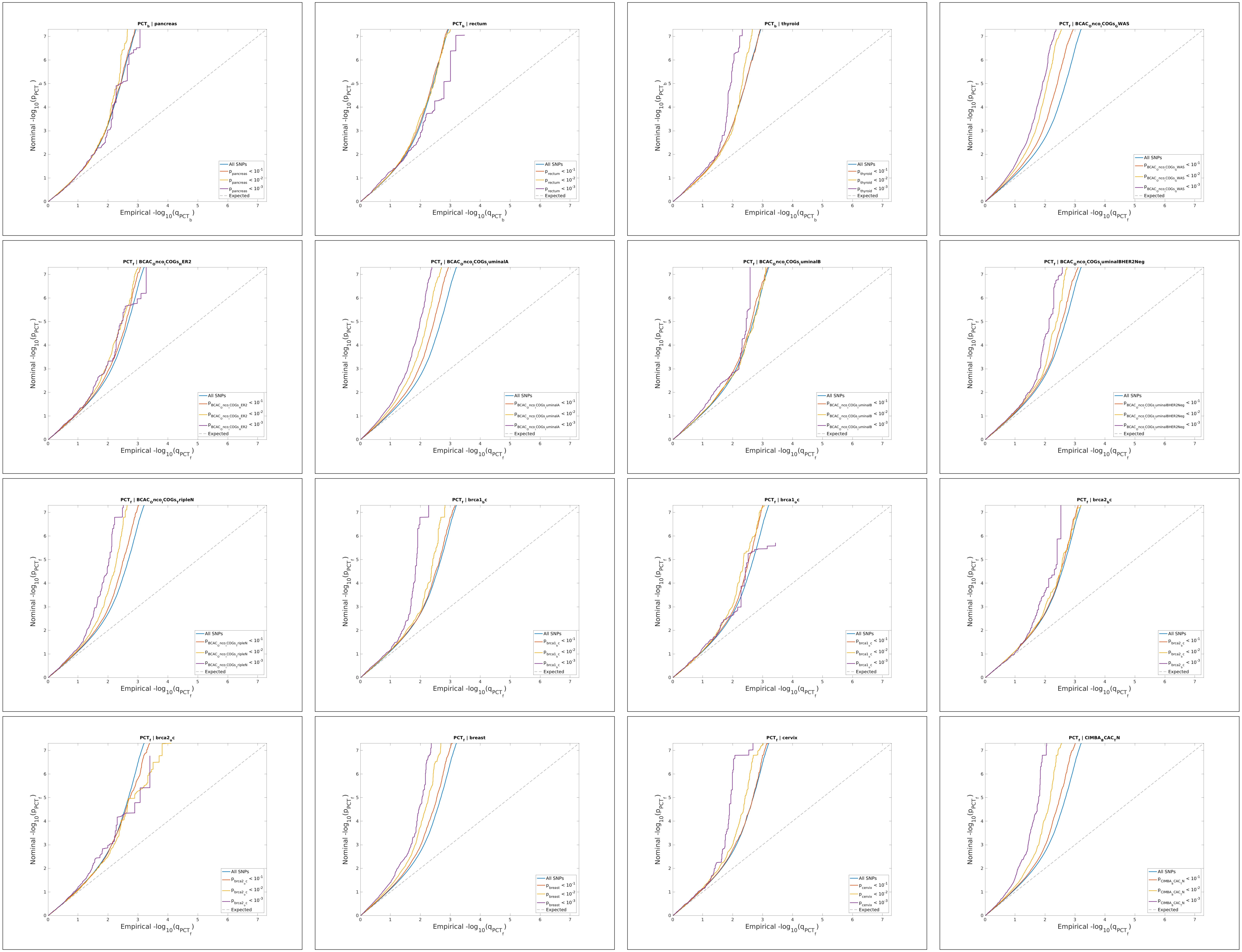

PCTb | pancreas
PCTb | rectum
PCTb | thyroid
PCTf | BCACOncoiCOGsGWAS
PCTf | BCACOncoiCOGsHER2
PCTf | BCACOncoiCOGsLuminalA
PCTf | BCACOncoiCOGsLuminalB
PCTf | BCACOncoiCOGsLuminalBHER2Neg
PCTf | BCACOncoiCOGsTripleN
PCTf | brca1bc
PCTf | brca1oc
PCTf | brca2bc
PCTf | brca2oc
PCTf | breast
PCTf | cervix
PCTf | CIMBABCACTN
Nominal -log10(pPCT)
Empirical -log10(qPCT)
All SNPs
Expected

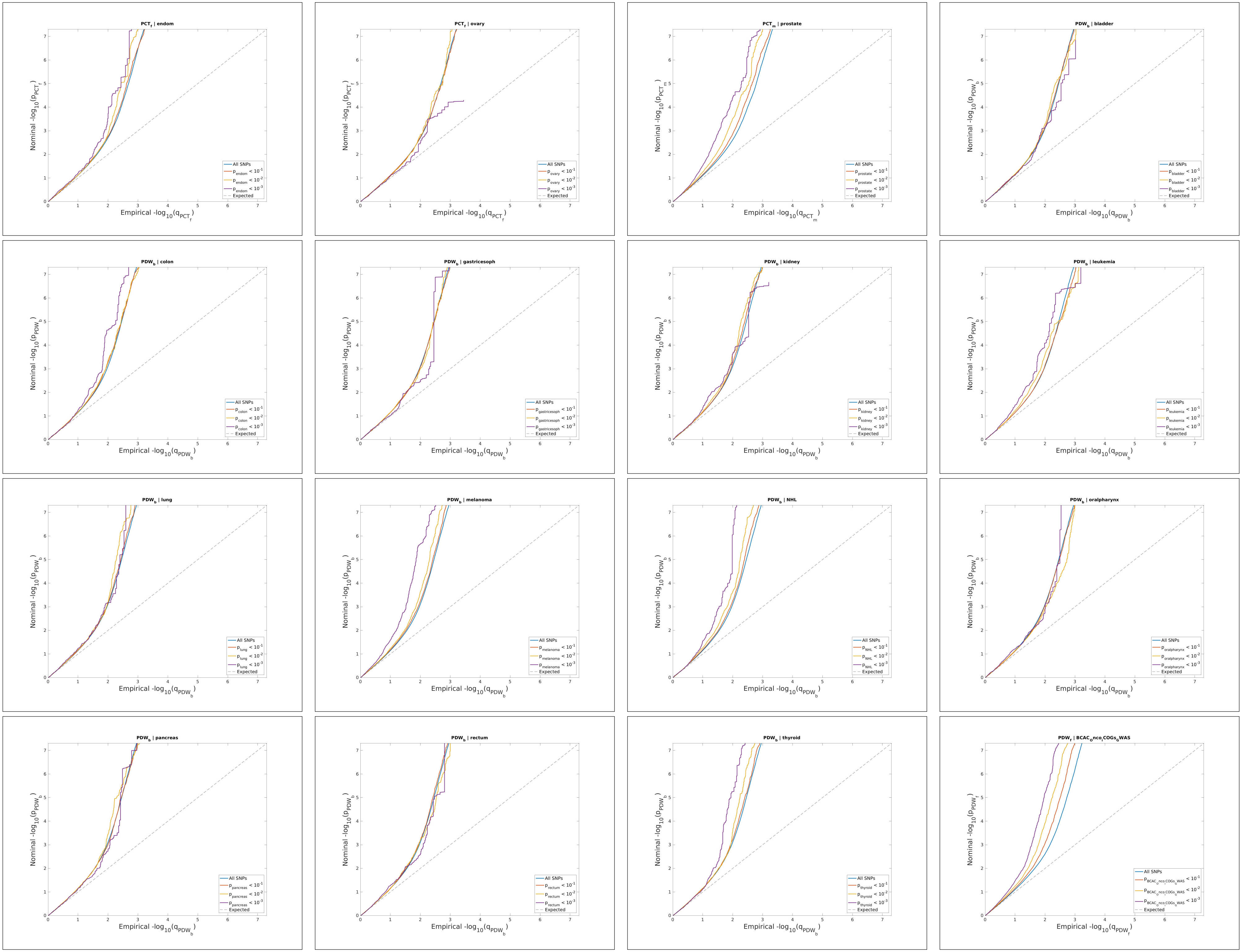
PCTf | endom
Nominal -log10(pPCTf)
Empirical -log10(qPCTf)
All SNPs
pendom < 10-1
pendom < 10-2
pendom < 10-3
Expected
PCTf | ovary
Nominal -log10(pPCTf)
Empirical -log10(qPCTf)
PCTm | prostate
Nominal -log10(pPCTm)
Empirical -log10(qPCTm)
PDWb | bladder
PDWb | colon
PDWb | gastricesoph
PDWb | kidney
PDWb | leukemia
PDWb | lung
PDWb | melanoma
PDWb | NHL
PDWb | oralpharynx
PDWb | pancreas
PDWb | rectum
PDWb | thyroid
Nominal -log10(pPDWb)
Empirical -log10(qPDWb)
PDWf | BCAConcoCOGsGWAS
Nominal -log10(pPDWf)
Empirical -log10(qPDWf)

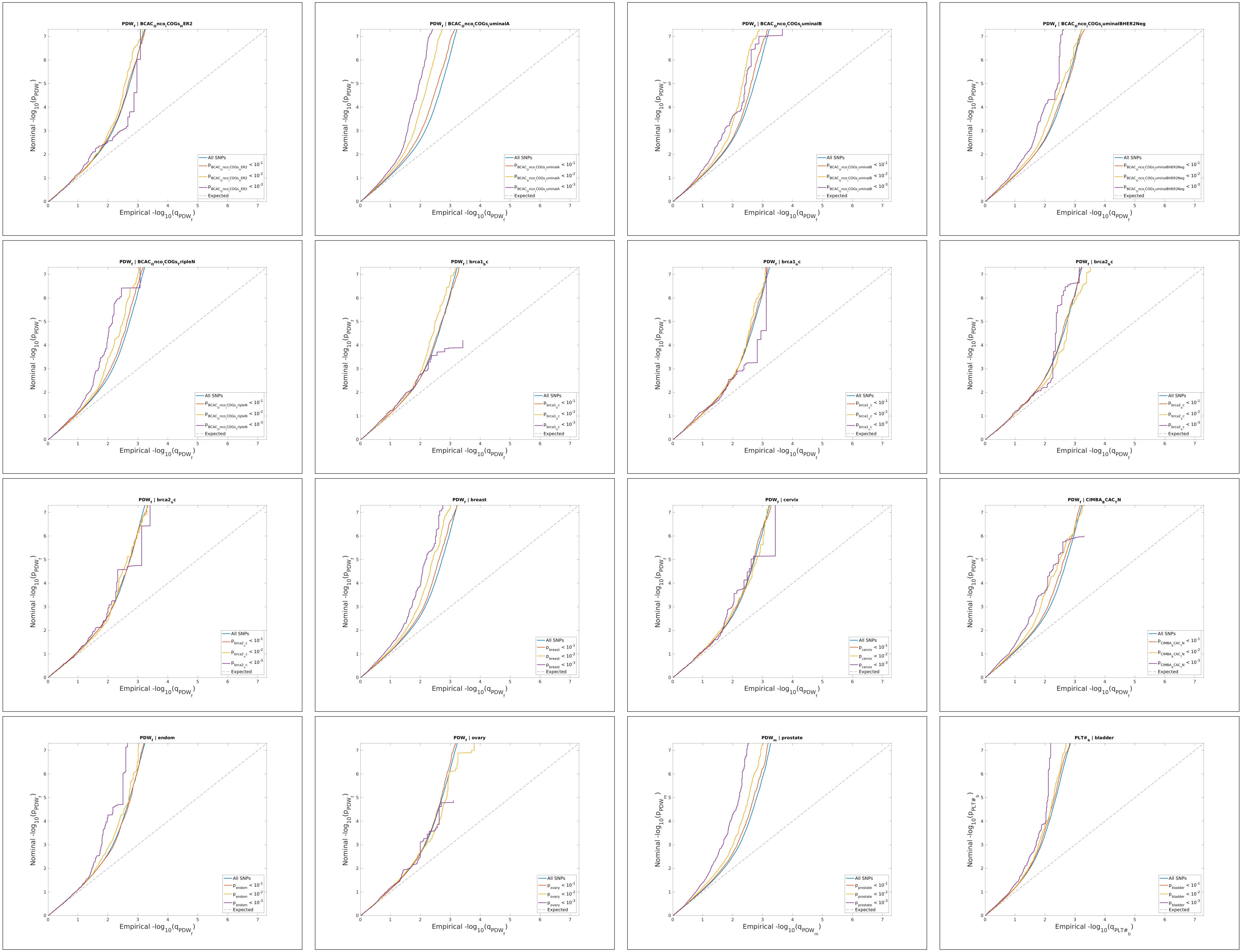

PDWf | BCAC_onco_COGs_HER2
PDWf | BCAC_onco_COGs_luminalA
PDWf | BCAC_onco_COGs_luminalB
PDWf | BCAC_onco_COGs_luminalBHER2Neg
PDWf | BCAC_onco_COGs_tripleN
PDWf | brca1_b_c
PDWf | brca1_o_c
PDWf | brca2_b_c
PDWf | brca2_o_c
PDWf | breast
PDWf | cervix
PDWf | CIMBA_BCAC_N
PDWf | endom
PDWf | ovary
PDWm | prostate
PLT#b | bladder
Nominal -log10(p)
Empirical -log10(q)
All SNPs
Expected

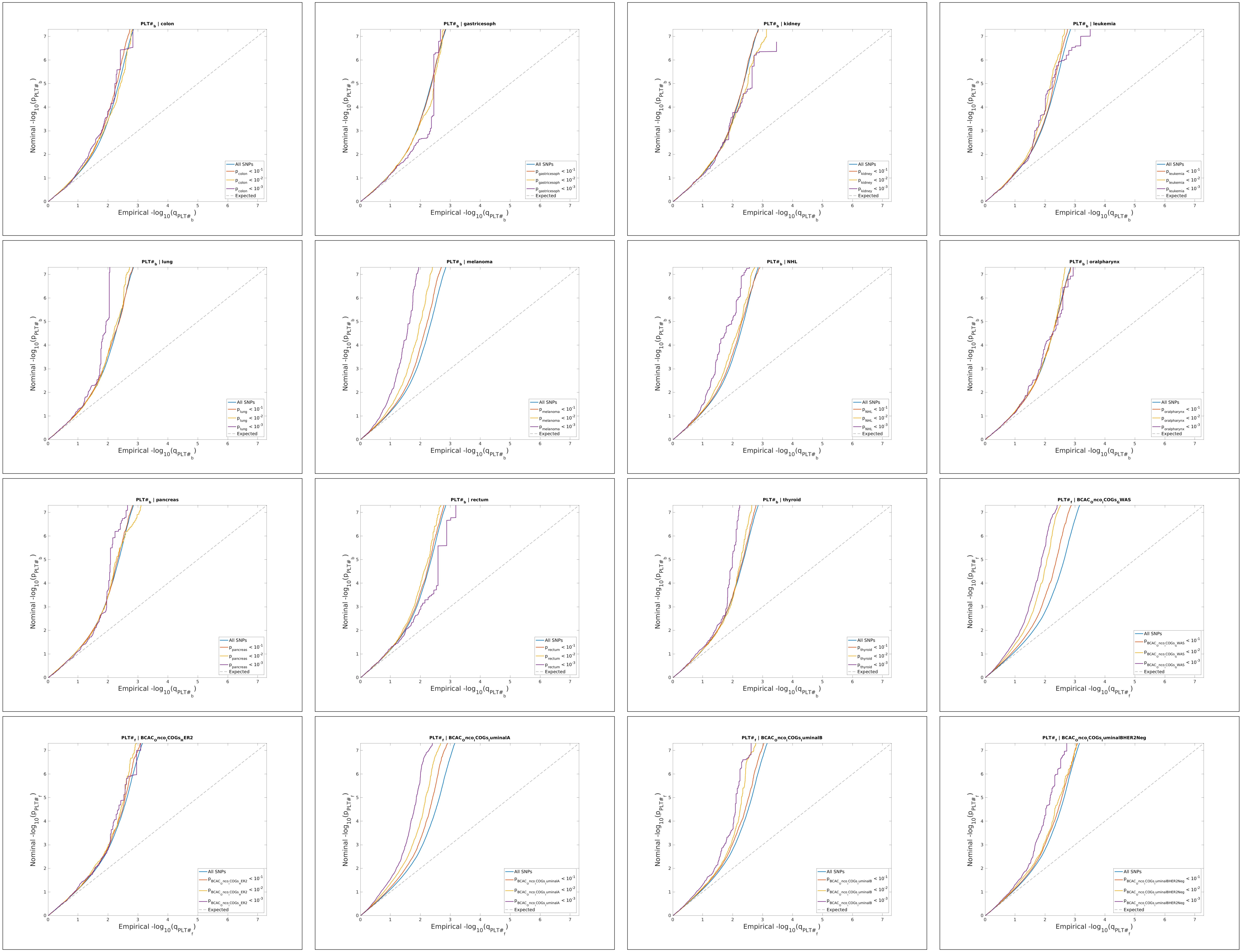

PLT#b | colon
PLT#b | gastricesoph
PLT#b | kidney
PLT#b | leukemia
PLT#b | lung
PLT#b | melanoma
PLT#b | NHL
PLT#b | oralpharynx
PLT#b | pancreas
PLT#b | rectum
PLT#b | thyroid
PLT#f | BCAConcoCOGsGWAS
PLT#f | BCAConcoCOGsHER2
PLT#f | BCAConcoCOGsLuminalA
PLT#f | BCAConcoCOGsLuminalB
PLT#f | BCAConcoCOGsLuminalBHER2Neg
Nominal -log10(pPLT#)
Empirical -log10(qPLT#)
All SNPs
Expected

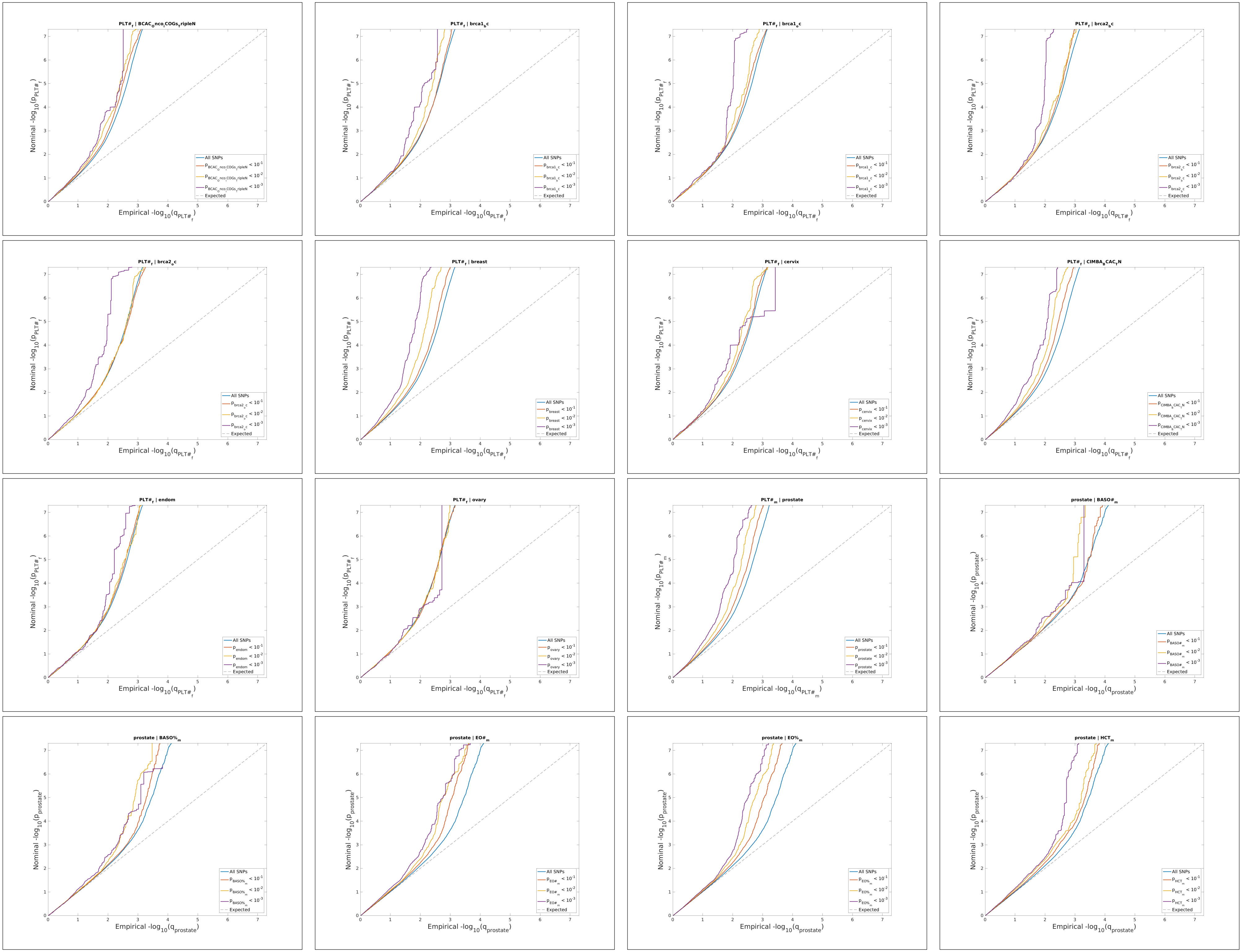

PLT#f | BCAConcoCOGsripleN
PLT#f | brca1bc
PLT#f | brca1oc
PLT#f | brca2bc
PLT#f | brca2oc
PLT#f | breast
PLT#f | cervix
PLT#f | CIMBABCACrN
PLT#f | endom
PLT#f | ovary
PLT#m | prostate
prostate | BASO#m
prostate | BASO%m
prostate | EO#m
prostate | EO%m
prostate | HCTm

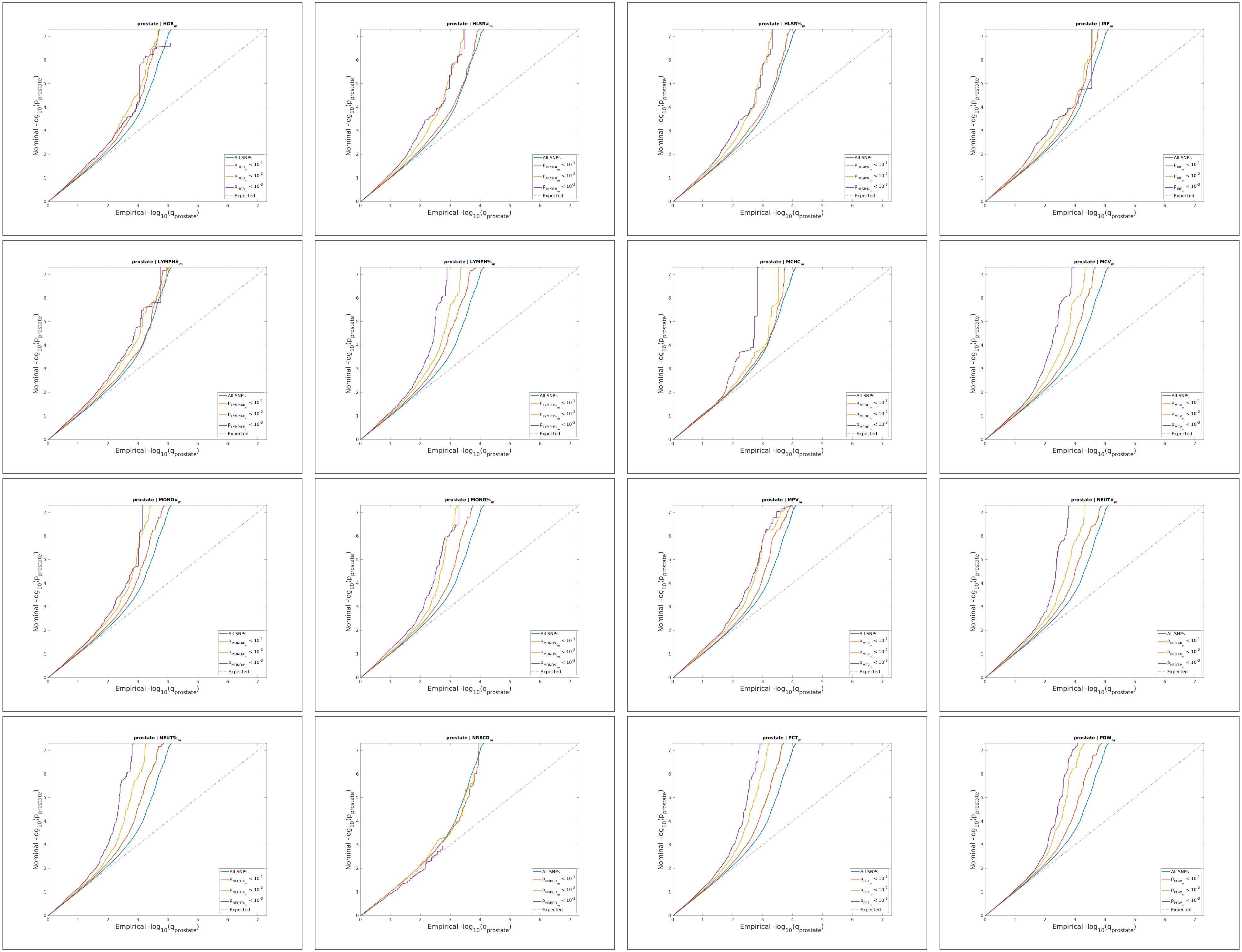
prostate | HGB_m
prostate | HLSR#_m
prostate | HLSR%_m
prostate | IRF_m
prostate | LYMPH#_m
prostate | LYMPH%_m
prostate | MCHC_m
prostate | MCV_m
prostate | MONO#_m
prostate | MONO%_m
prostate | MPV_m
prostate | NEUT#_m
prostate | NEUT%_m
prostate | NRBCD_m
prostate | PCT_m
prostate | PDW_m
Nominal -log10(p prostate)
Empirical -log10(q prostate)
All SNPs
p < 10^-1
p < 10^-2
p < 10^-3
Expected

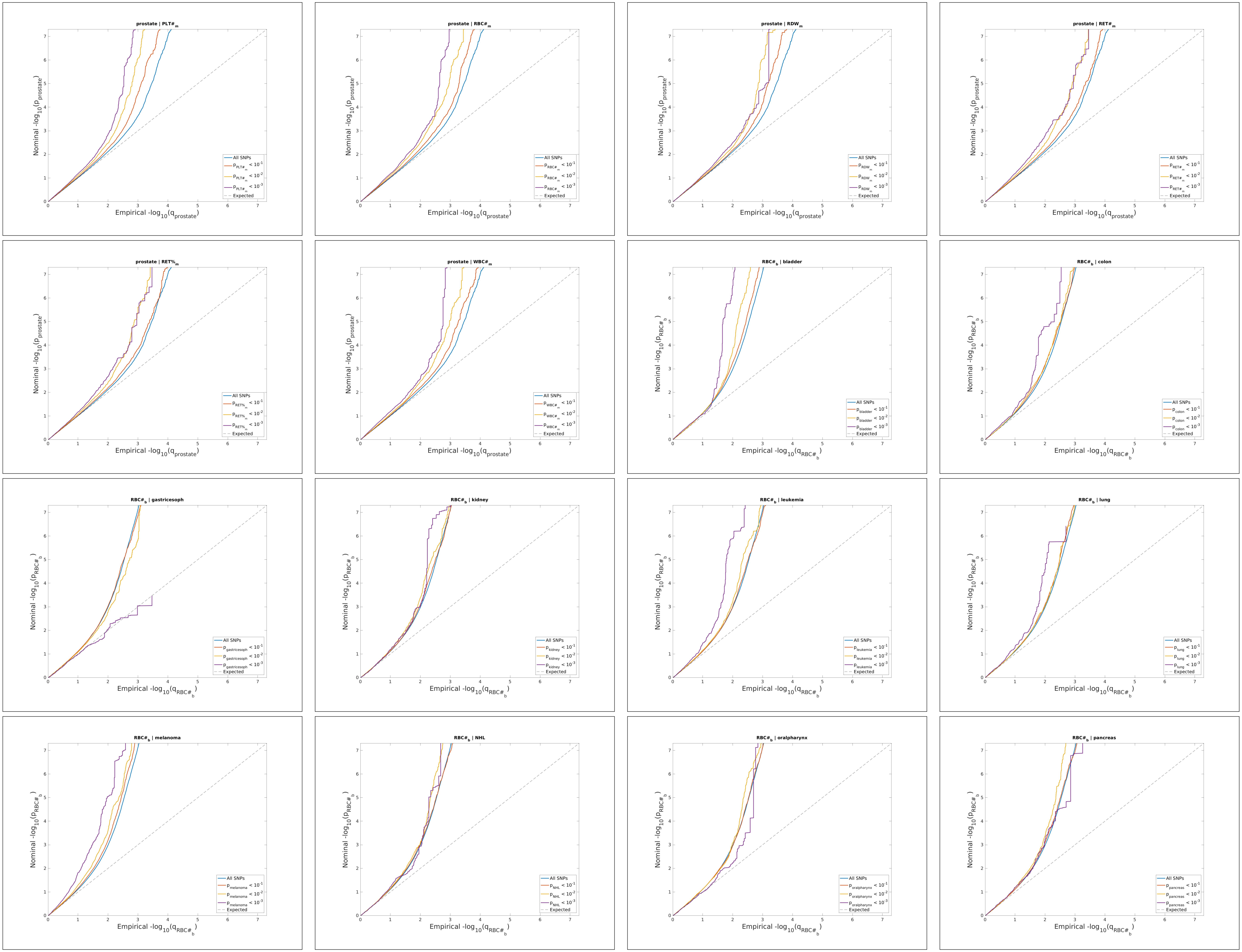
prostate | PLT$\#_m$
prostate | RBC$\#_m$
prostate | RDW$_m$
prostate | RET$\#_m$
prostate | RET%$_m$
prostate | WBC$\#_m$
RBC$\#_b$ | bladder
RBC$\#_b$ | colon
RBC$\#_b$ | gastricesoph
RBC$\#_b$ | kidney
RBC$\#_b$ | leukemia
RBC$\#_b$ | lung
RBC$\#_b$ | melanoma
RBC$\#_b$ | NHL
RBC$\#_b$ | oralpharynx
RBC$\#_b$ | pancreas
Nominal -log$_{10}$(p$_{prostate}$)
Empirical -log$_{10}$(q$_{prostate}$)
Nominal -log$_{10}$(p$_{RBC\#_b}$)
Empirical -log$_{10}$(q$_{RBC\#_b}$)
All SNPs
$p < 10^{-1}$
$p < 10^{-2}$
$p < 10^{-3}$
Expected

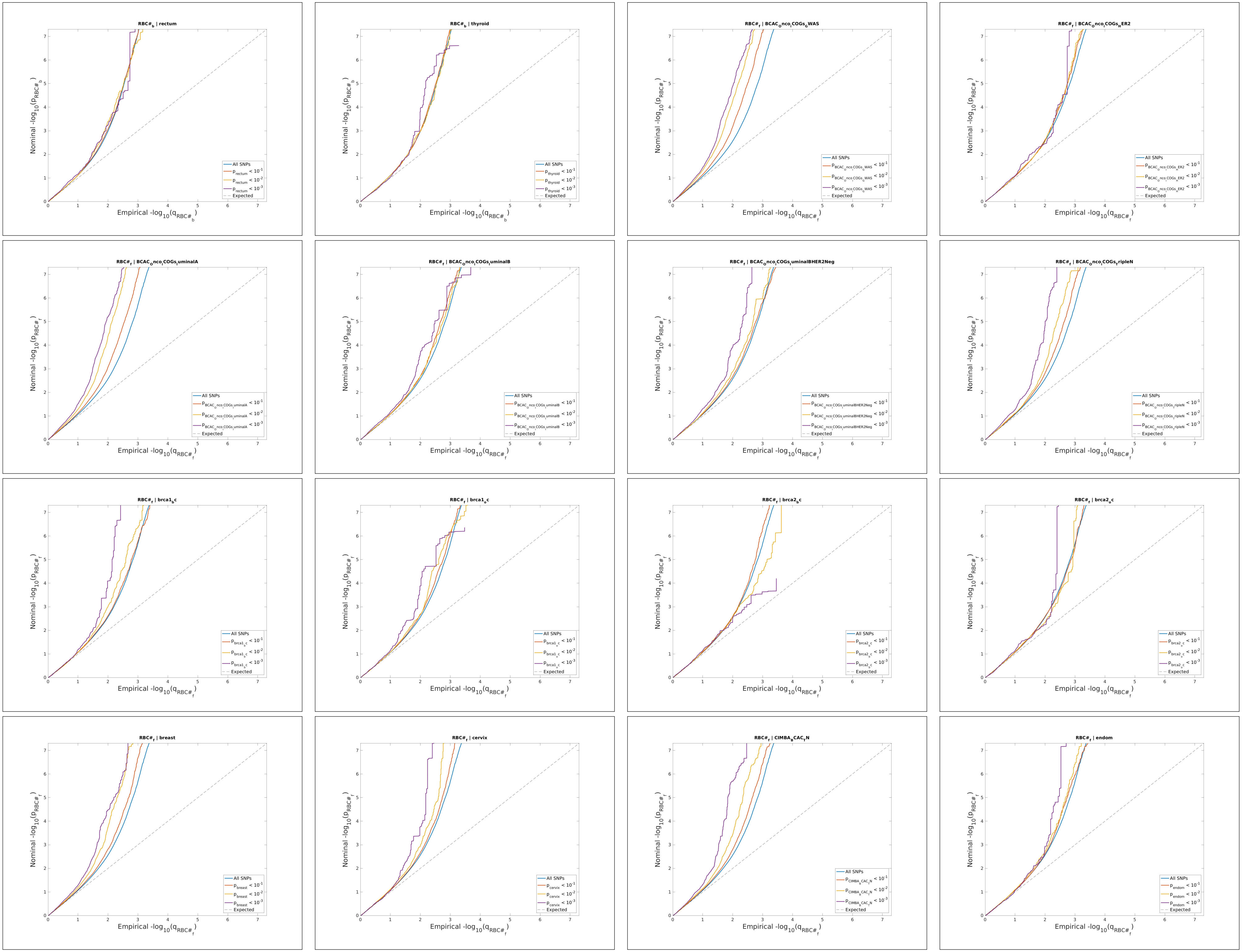

RBC#b | rectum
RBC#b | thyroid
RBC#f | BCAConcoCOGsGWAS
RBC#f | BCAConcoCOGsHER2
RBC#f | BCAConcoCOGsLuminalA
RBC#f | BCAConcoCOGsLuminalB
RBC#f | BCAConcoCOGsLuminalBHER2Neg
RBC#f | BCAConcoCOGsTripleN
RBC#f | brca1bc
RBC#f | brca1oc
RBC#f | brca2bc
RBC#f | brca2oc
RBC#f | breast
RBC#f | cervix
RBC#f | CIMBABCACTN
RBC#f | endom
Nominal -log10(pRBC#)
Empirical -log10(qRBC#)
All SNPs
Expected

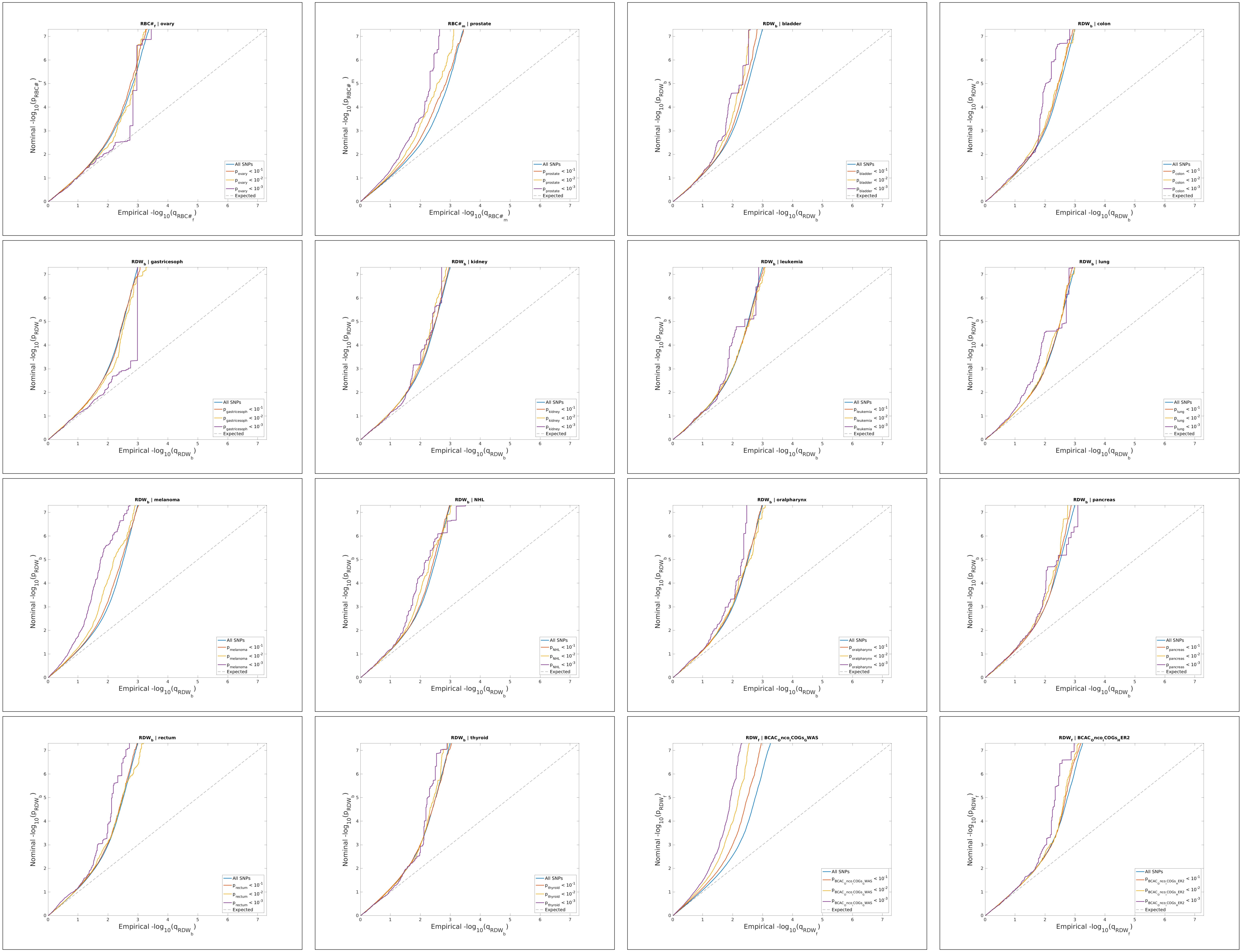
RBC#f | ovary
RBC#m | prostate
RDWb | bladder
RDWb | colon
RDWb | gastricesoph
RDWb | kidney
RDWb | leukemia
RDWb | lung
RDWb | melanoma
RDWb | NHL
RDWb | oralpharynx
RDWb | pancreas
RDWb | rectum
RDWb | thyroid
RDWf | BCAC_Onco_COGs_GWAS
RDWf | BCAC_Onco_COGs_HER2
Nominal -log10(p)
Empirical -log10(q)
All SNPs
p < 10-1
p < 10-2
p < 10-3
Expected

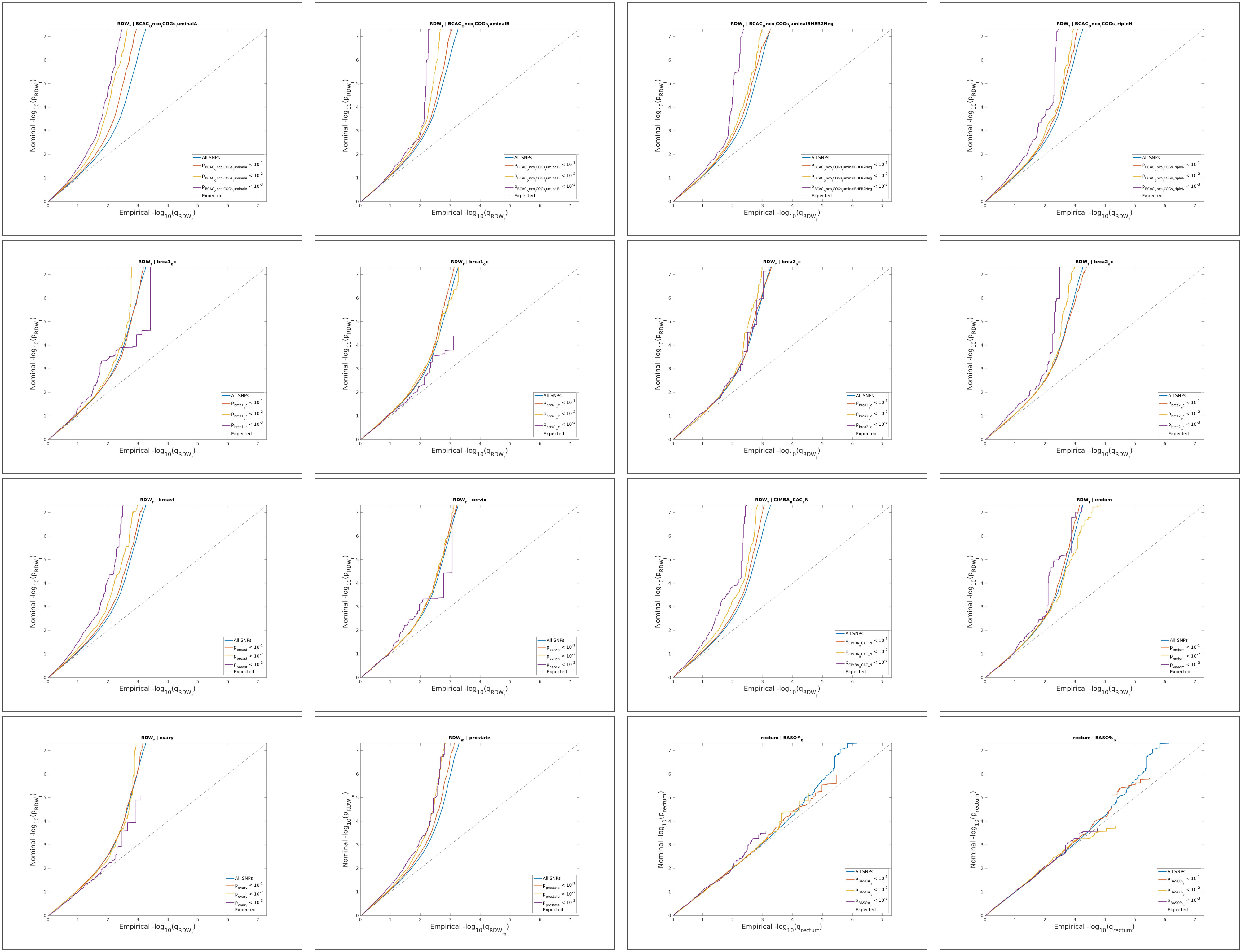

RDWf | BCAConcoCOGsluminalA
RDWf | BCAConcoCOGsluminalB
RDWf | BCAConcoCOGsluminalBHER2Neg
RDWf | BCAConcoCOGstripleN
RDWf | brca1bc
RDWf | brca1oc
RDWf | brca2bc
RDWf | brca2oc
RDWf | breast
RDWf | cervix
RDWf | CIMBABCACTN
RDWf | endom
RDWf | ovary
RDWm | prostate
rectum | BASO#b
rectum | BASO%b
Nominal -log10(pRDWf)
Empirical -log10(qRDWf)
Nominal -log10(prectum)
Empirical -log10(qrectum)
All SNPs
Expected

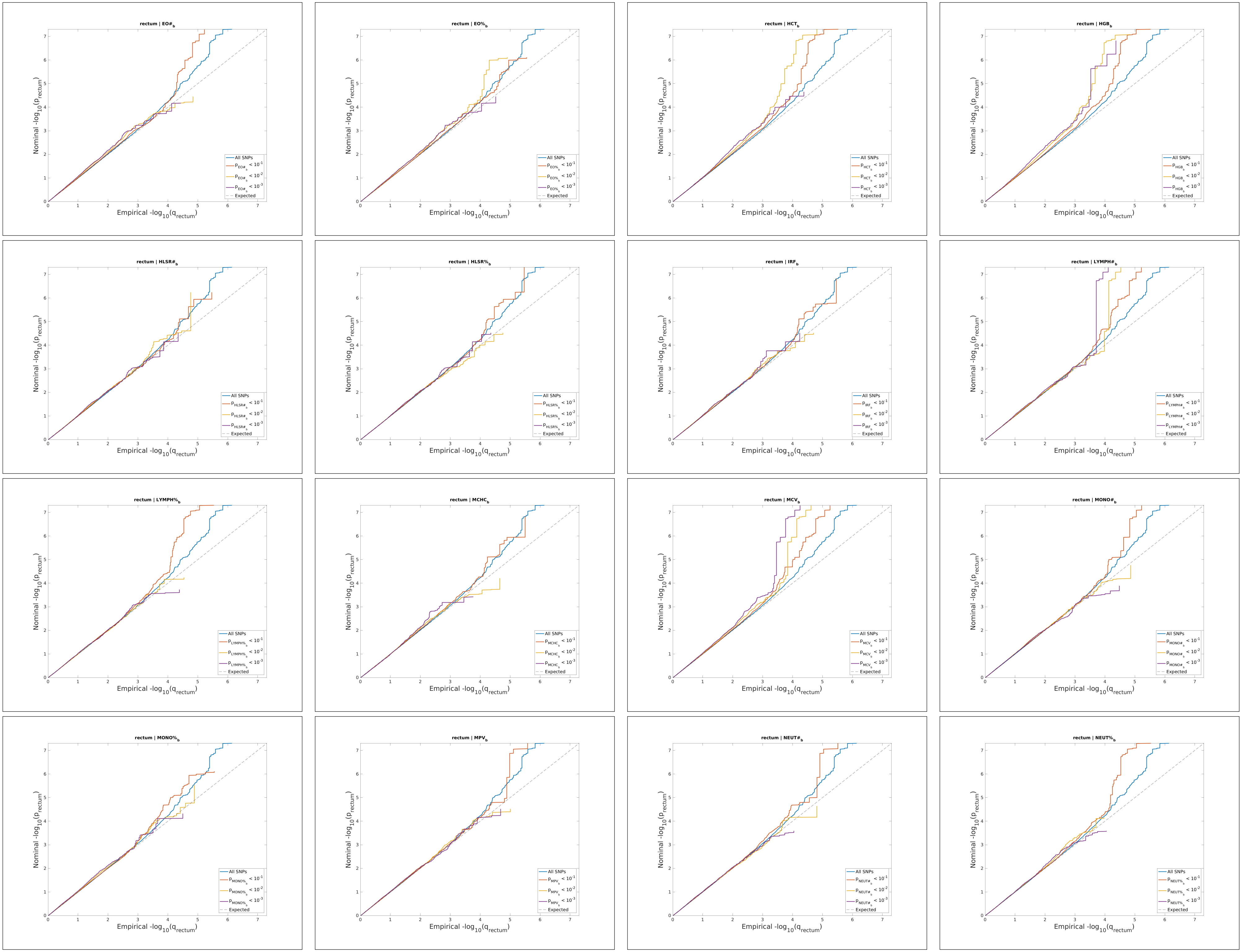
rectum | $EO\#_b$
rectum | $EO\%_b$
rectum | HCT_b
rectum | HGB_b
rectum | $HLSR\#_b$
rectum | $HLSR\%_b$
rectum | IRF_b
rectum | $LYMPH\#_b$
rectum | $LYMPH\%_b$
rectum | $MCHC_b$
rectum | MCV_b
rectum | $MONO\#_b$
rectum | $MONO\%_b$
rectum | MPV_b
rectum | $NEUT\#_b$
rectum | $NEUT\%_b$
Nominal $-\log_{10}(p_{rectum})$
Empirical $-\log_{10}(q_{rectum})$
All SNPs
$p < 10^{-1}$
$p < 10^{-2}$
$p < 10^{-3}$
Expected

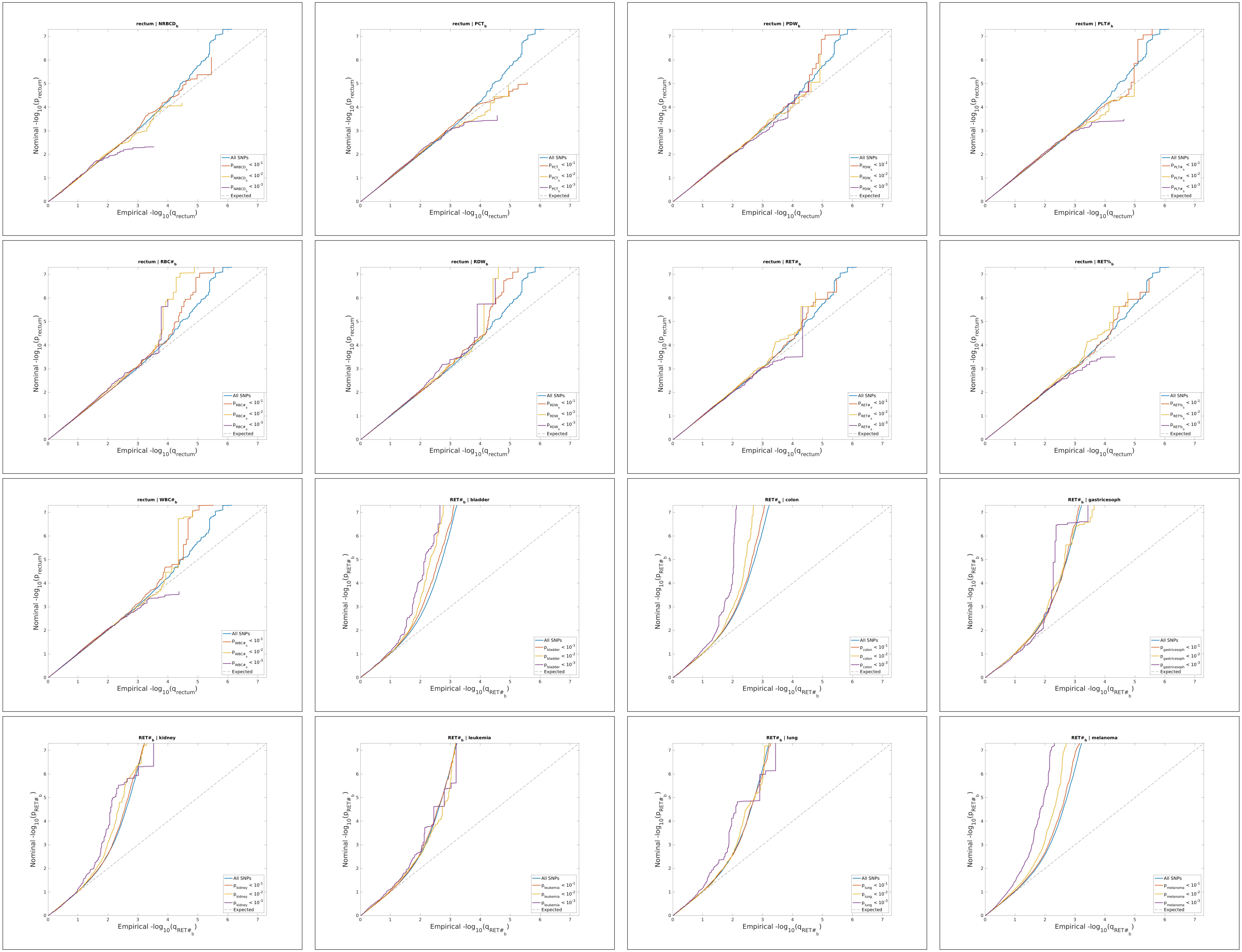

rectum | $NRBCD_b$
rectum | PCT_b
rectum | PDW_b
rectum | $PLT\#_b$
rectum | $RBC\#_b$
rectum | RDW_b
rectum | $RET\#_b$
rectum | $RET\%_b$
rectum | $WBC\#_b$
$RET\#_b$ | bladder
$RET\#_b$ | colon
$RET\#_b$ | gastricesoph
$RET\#_b$ | kidney
$RET\#_b$ | leukemia
$RET\#_b$ | lung
$RET\#_b$ | melanoma
Nominal $-\log_{10}(p_{rectum})$
Empirical $-\log_{10}(q_{rectum})$
Nominal $-\log_{10}(p_{RET\#_b})$
Empirical $-\log_{10}(q_{RET\#_b})$
All SNPs
$p < 10^{-1}$
$p < 10^{-2}$
$p < 10^{-3}$
Expected

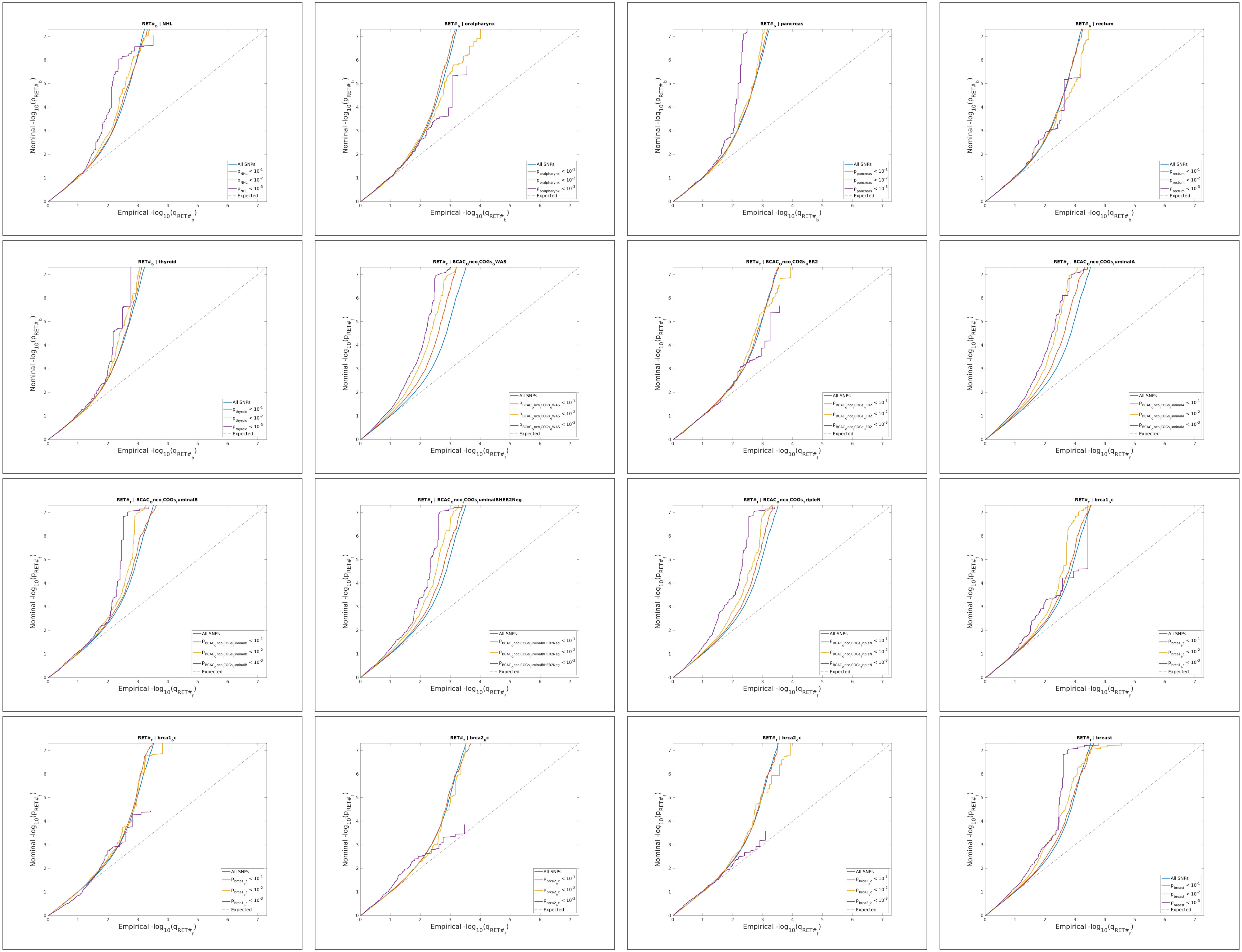
RET#b | NHL
RET#b | oralpharynx
RET#b | pancreas
RET#b | rectum
RET#b | thyroid
RET#f | BCACOncoCOGsGWAS
RET#f | BCACOncoCOGsHER2
RET#f | BCACOncoCOGsLuminalA
RET#f | BCACOncoCOGsLuminalB
RET#f | BCACOncoCOGsLuminalBHER2Neg
RET#f | BCACOncoCOGsTripleN
RET#f | brca1bc
RET#f | brca1oc
RET#f | brca2bc
RET#f | brca2oc
RET#f | breast
Nominal -log10(pRET#)
Empirical -log10(qRET#)
All SNPs
< 10-1
< 10-2
< 10-3
Expected

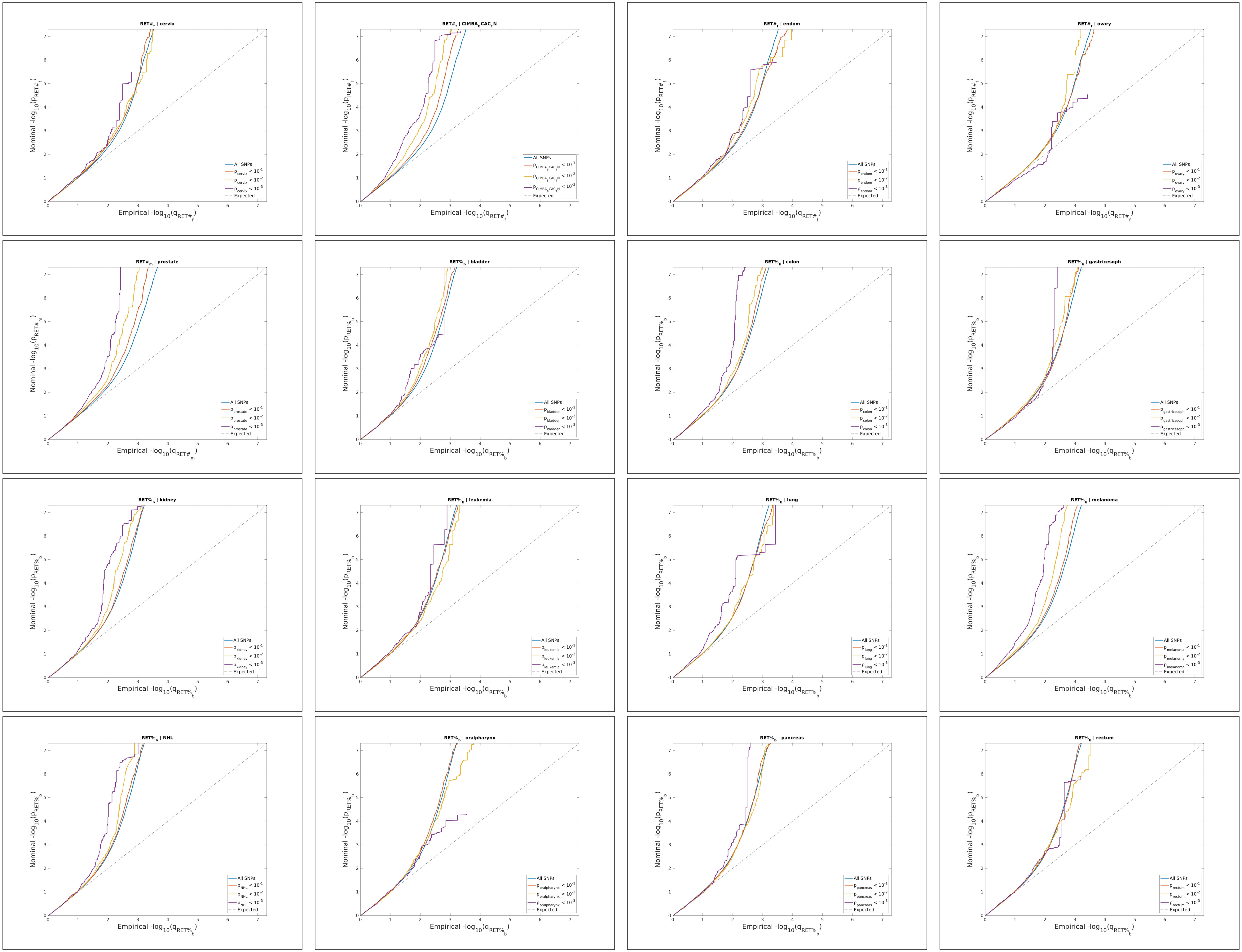
RET#f | cervix
RET#f | CIMBA_BCAC_TN
RET#f | endom
RET#f | ovary
RET#m | prostate
RET%b | bladder
RET%b | colon
RET%b | gastricesoph
RET%b | kidney
RET%b | leukemia
RET%b | lung
RET%b | melanoma
RET%b | NHL
RET%b | oralpharynx
RET%b | pancreas
RET%b | rectum
Nominal -log10(p)
Empirical -log10(q)
All SNPs
p < 10-1
p < 10-2
p < 10-3
Expected

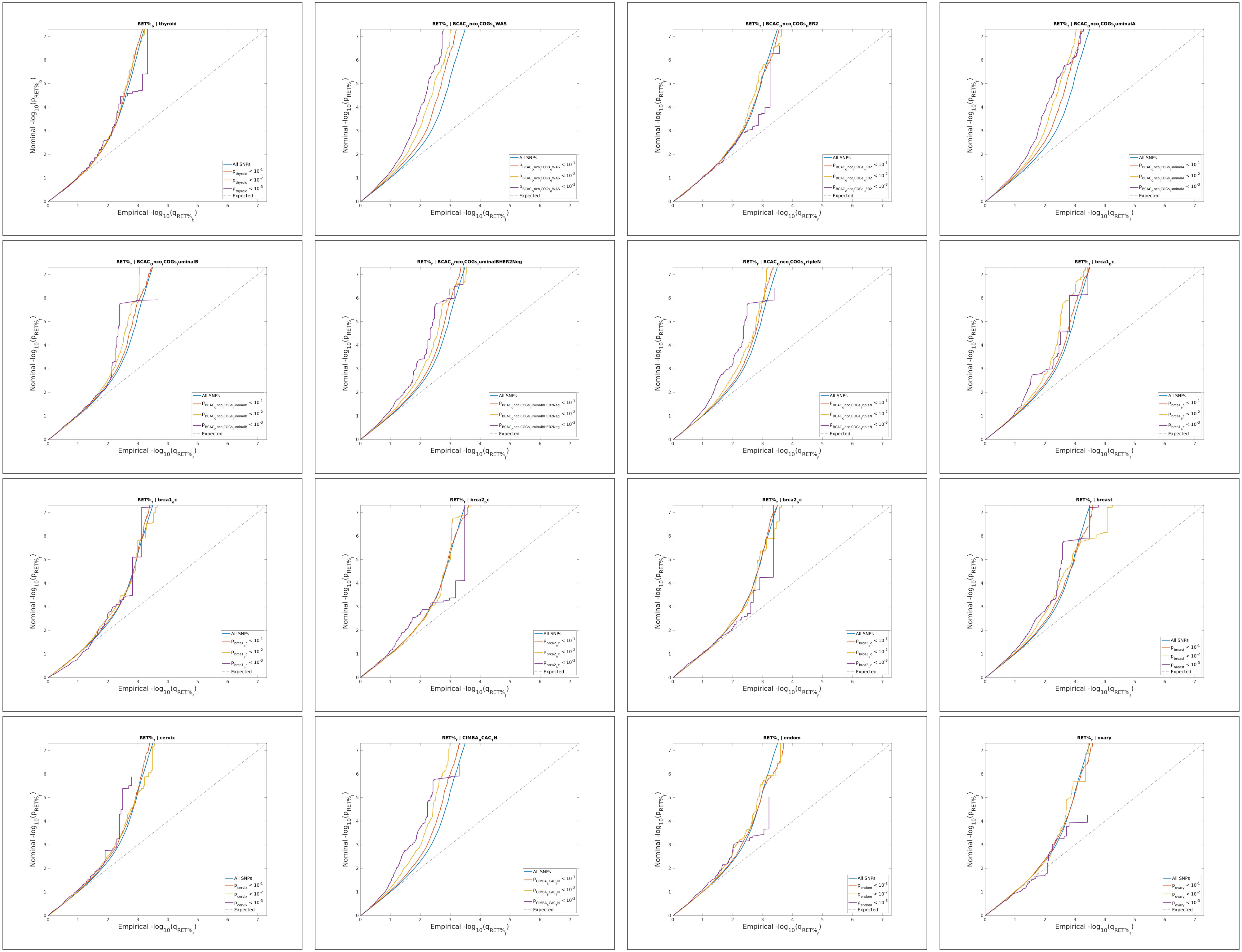
RET%b | thyroid
RET%f | BCACOncoCOGsGWAS
RET%f | BCACOncoCOGsER2
RET%f | BCACOncoCOGsLuminalA
RET%f | BCACOncoCOGsLuminalB
RET%f | BCACOncoCOGsLuminalBHER2Neg
RET%f | BCACOncoCOGsTripleN
RET%f | brca1bc
RET%f | brca1oc
RET%f | brca2bc
RET%f | brca2oc
RET%f | breast
RET%f | cervix
RET%f | CIMBABCACTN
RET%f | endom
RET%f | ovary
Nominal -log10(pRET%f)
Empirical -log10(qRET%f)
All SNPs
Expected

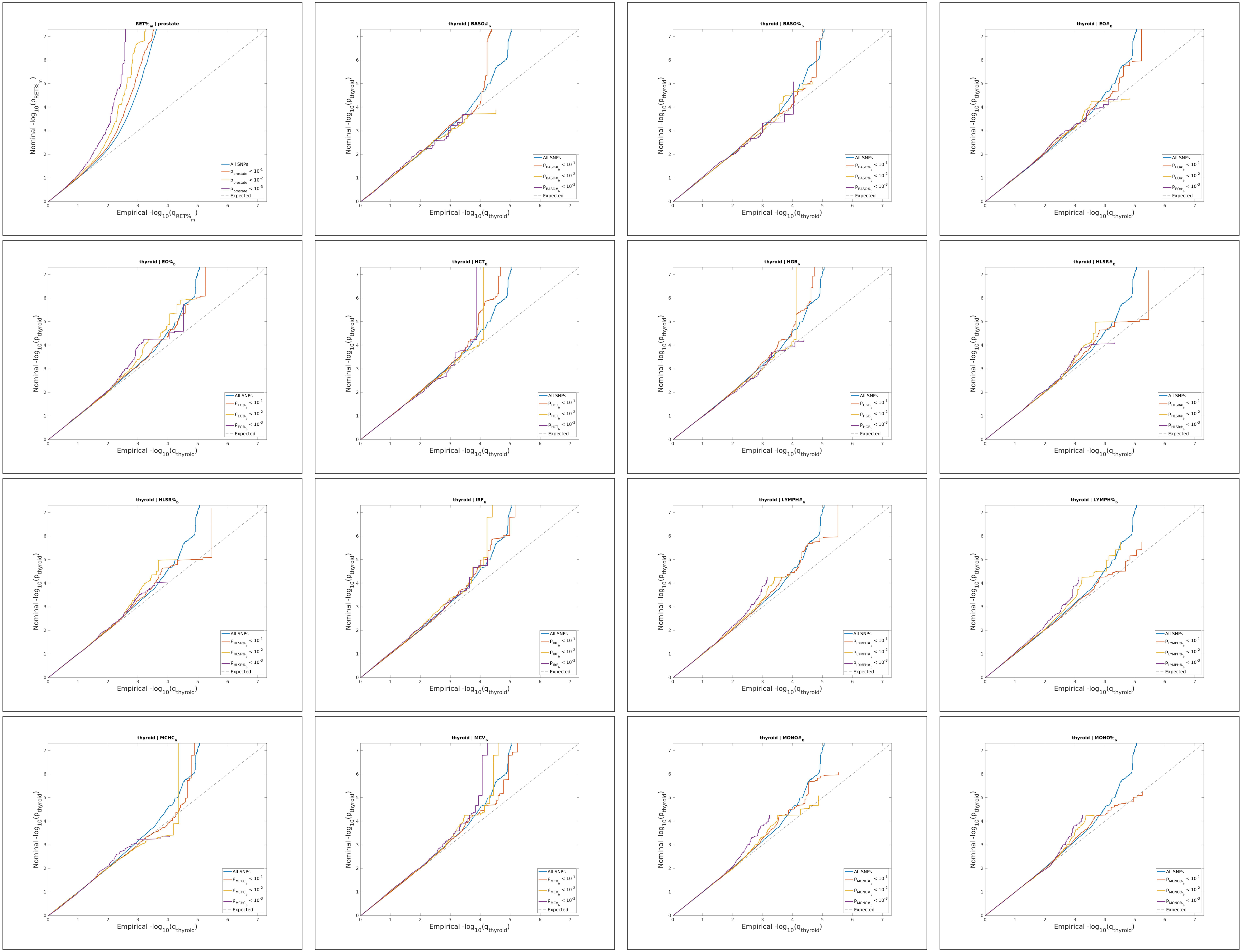
RET%m | prostate
thyroid | BASO#b
thyroid | BASO%b
thyroid | EO#b
thyroid | EO%b
thyroid | HCTb
thyroid | HGBb
thyroid | HLSR#b
thyroid | HLSR%b
thyroid | IRFb
thyroid | LYMPH#b
thyroid | LYMPH%b
thyroid | MCHCb
thyroid | MCVb
thyroid | MONO#b
thyroid | MONO%b
Nominal -log10(pthyroid)
Empirical -log10(qthyroid)
All SNPs
Expected

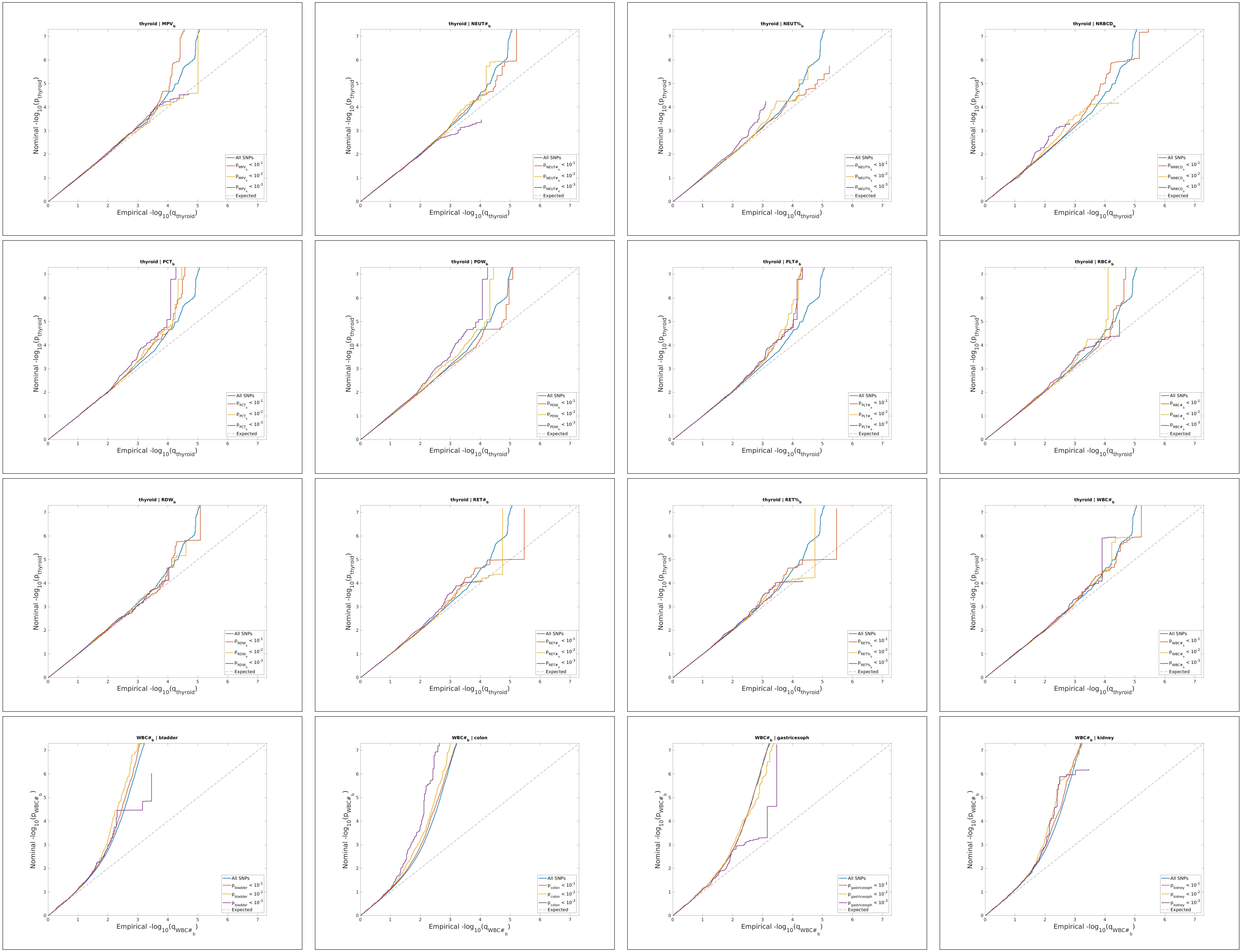

thyroid | MPV_b
thyroid | NEUT#_b
thyroid | NEUT%_b
thyroid | NRBCD_b
thyroid | PCT_b
thyroid | PDW_b
thyroid | PLT#_b
thyroid | RBC#_b
thyroid | RDW_b
thyroid | RET#_b
thyroid | RET%_b
thyroid | WBC#_b
WBC#_b | bladder
WBC#_b | colon
WBC#_b | gastricesoph
WBC#_b | kidney
Nominal -log10(p_thyroid)
Empirical -log10(q_thyroid)
Nominal -log10(p_WBC#_b)
Empirical -log10(q_WBC#_b)
All SNPs
Expected

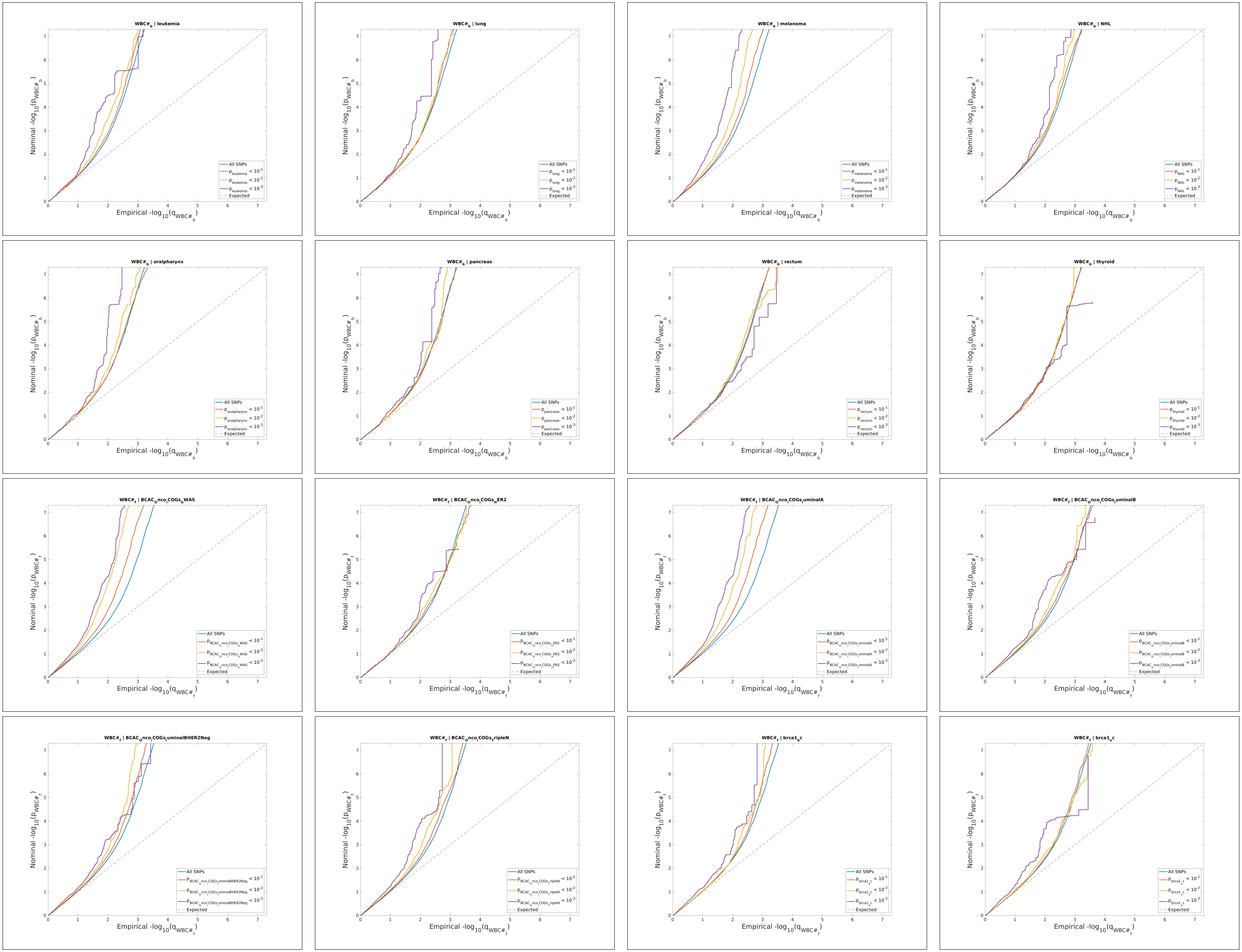
WBC#b | leukemia
WBC#b | lung
WBC#b | melanoma
WBC#b | NHL
WBC#b | oralpharynx
WBC#b | pancreas
WBC#b | rectum
WBC#b | thyroid
WBC#f | BCAC_onco_COGs_GWAS
WBC#f | BCAC_onco_COGs_HER2
WBC#f | BCAC_onco_COGs_LuminalA
WBC#f | BCAC_onco_COGs_LuminalB
WBC#f | BCAC_onco_COGs_LuminalBHER2Neg
WBC#f | BCAC_onco_COGs_TripleN
WBC#f | brca1_bc
WBC#f | brca1_oc
Nominal -log10(p)
Empirical -log10(q)
All SNPs
Expected

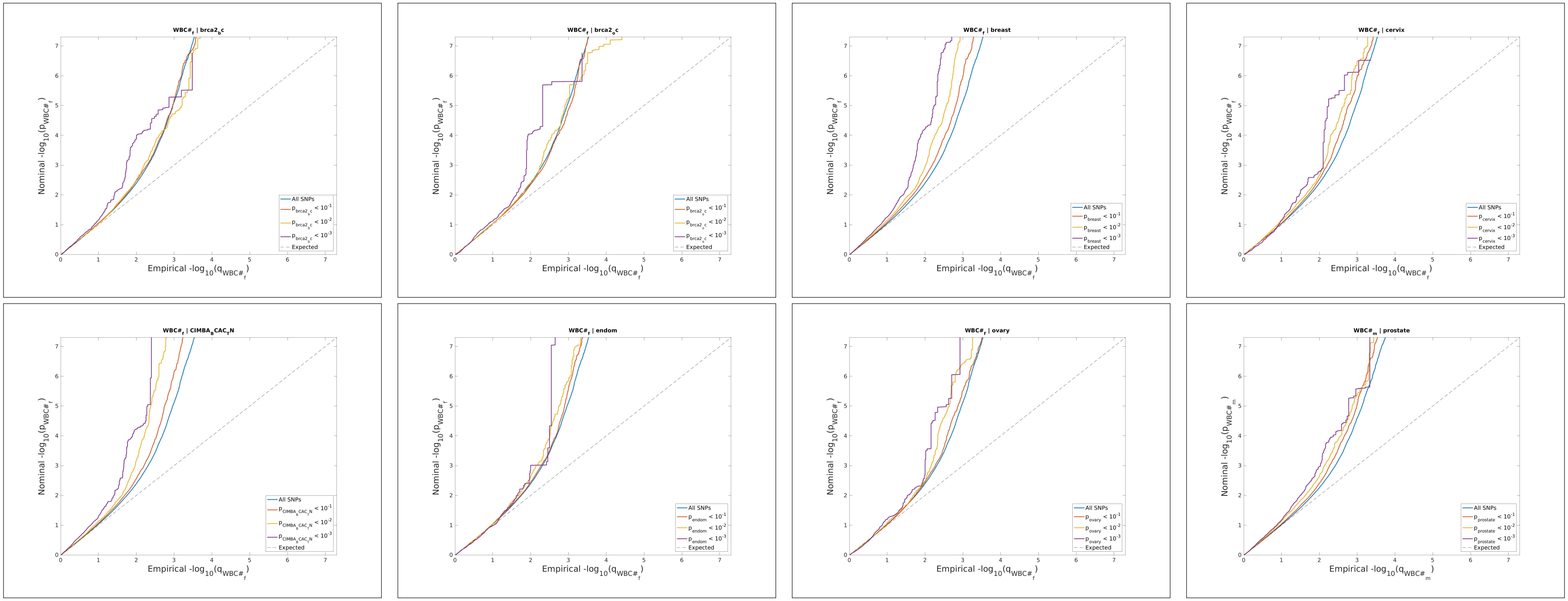
WBC#f | brca2bc
WBC#f | brca2oc
WBC#f | breast
WBC#f | cervix
WBC#f | CIMBABCACTN
WBC#f | endom
WBC#f | ovary
WBC#m | prostate
Nominal -log10(pWBC#f)
Nominal -log10(pWBC#m)
Empirical -log10(qWBC#f)
Empirical -log10(qWBC#m)
All SNPs
pbrca2bc < 10-1
pbrca2bc < 10-2
pbrca2bc < 10-3
pbrca2oc < 10-1
pbrca2oc < 10-2
pbrca2oc < 10-3
pbreast < 10-1
pbreast < 10-2
pbreast < 10-3
pcervix < 10-1
pcervix < 10-2
pcervix < 10-3
pCIMBABCACTN < 10-1
pCIMBABCACTN < 10-2
pCIMBABCACTN < 10-3
pendom < 10-1
pendom < 10-2
pendom < 10-3
povary < 10-1
povary < 10-2
povary < 10-3
pprostate < 10-1
pprostate < 10-2
pprostate < 10-3
Expected
